# Direct allylic C–H alkylation of enol silyl ethers enabled by photoredox–Brønsted base hybrid catalysis

**DOI:** 10.1038/s41467-019-10641-y

**Published:** 2019-06-20

**Authors:** Kohsuke Ohmatsu, Tsubasa Nakashima, Makoto Sato, Takashi Ooi

**Affiliations:** 1 Institute of Transformative Bio-Molecules (WPI-ITbM), and Department of Molecular and Macromolecular Chemistry, Graduate School of Engineering, Nagoya University, Nagoya, 464-8601 Japan; 2 CREST, Japan Science and Technology Agency (JST), Nagoya, 464-8601 Japan

**Keywords:** Catalytic mechanisms, Homogeneous catalysis, Photocatalysis, Synthetic chemistry methodology

## Abstract

Strategies for altering the reaction pathway of reactive intermediates are of significant importance in diversifying organic synthesis. Enol silyl ethers, versatile enolate equivalents, are known to undergo one-electron oxidation to generate the radical cations that spontaneously form electrophilic α-carbonyl radicals via elimination of the silyl groups. Here, we demonstrate that close scrutiny of the property of the radical cations as strong C–H acids enables the identification of a catalyst system consisting of an iridium-based photosensitizer and 2,4,6-collidine for the generation of nucleophilic allylic radicals from enol silyl ethers through one-electron oxidation-deprotonation sequence under light irradiation without the desilylation of the radical cation intermediates. The resultant allylic radicals engage in the addition to electron-deficient olefins, establishing the selective allylic C-H alkylation of enol silyl ethers. This strategy is broadly applicable, and the alkylated enol silyl ethers can be transformed into highly functionalized carbonyl compounds by exploiting their common polar reactivity.

## Abstract (web summary)

Allylic C–H activation of enol silyl ethers via one-electron oxidation is usually accompanied by undesired desilylation. Here, under hybrid photoredox and Brønsted base catalysis, the authors show the mild functionalization of enol silyl ethers, providing a platform to access an array of complex carbonyl compounds.

## Introduction

**Fig. 1:**
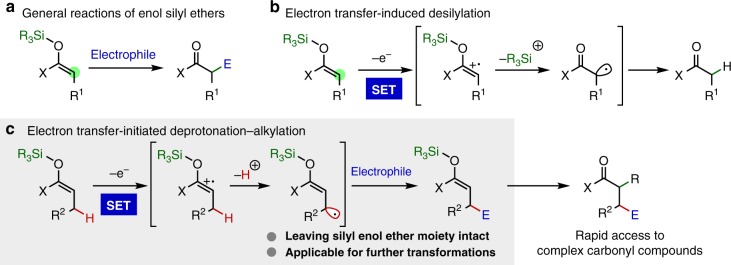
Transformations of enol silyl ethers. **a** General reaction pathway. **b** Known desilylation reaction initiated by single-electron oxidation. **c** The reaction design of allylic C–H alkylation

Enol silyl ethers and their analogs are one of the most versatile substrate classes and enjoy widespread applications in organic synthesis (Fig. 1a)^1–3^. They can be prepared from carbonyl compounds of all oxidation states, such as aldehydes, ketones, esters and amides, by reliable protocols and exhibit preeminent reactivity as enolate anion equivalents amenable to various catalysis manifolds. These distinct features render enol silyl ethers attractive yet powerful handles for selective α-functionalization of a wide array of carbonyl entities.

On the other hand, the allylic sp^3^-hybridized carbons of the enol silyl ethers are potential reaction sites as the electron-rich enol moiety contributes to decreasing bond-dissociation enthalpy of the allylic C–H bonds. In particular, selective bond formation at the allylic carbons^4^ with preservation of the enol silyl ether component offers an opportunity to harness the reactivity of the resulting functionalized enol silyl ethers for the conventional polar reactions, enabling access to α,β-difunctionalized carbonyl compounds^5–7^. However, despite their potential synthetic utility, only a few catalytic systems are available for direct allylic C–H functionalization of enol silyl ethers or their analogs, which rely on transition metal catalysis and synergistic photoredox-thiol catalysis^8,9^.

In addition to the common polar reactivity useful for a diverse set of transformations, it has long been recognized that enol silyl ethers undergo single-electron oxidation to generate the corresponding radical cations^10^. Yet, previous efforts for exploiting this radical reactivity in reaction development have been restricted to coupling with concomitantly generated heteroatom radicals^11–15^ or tethered olefins at the α-carbon of the carbonyl group; this is largely due to the strong propensity of the radical cation to undergo elimination of a silyl cation to produce parent carbonyls (Fig. 1b)^10,16–22^. Under these circumstances, we considered the intrinsic properties of this class of radical cations, specifically the presumed high acidity of allylic protons^23–27^. For instance, MacMillan^28–31^ reported that radical cations generated from aldehyde- or cyclic ketone-derived enamines underwent deprotonation to form allylic radicals that were susceptible to radical addition reactions for β-functionalizations of the parent carbonyls. With this profile in mind, we envisioned that the allylic proton of the radical cation derived from an enol silyl ether could be readily deprotonated to generate an allylic radical nucleophilic enough to be captured by an external electrophile (Fig. 1c). If the employment of an appropriate Brønsted base allows this proton abstraction event to occur predominantly over the desilylation, the single-electron oxidation–deprotonation sequence would provide a broadly applicable platform to execute bond construction exclusively at the allylic carbon, leaving the enol silyl ether component intact for further synthetic manipulations. Here, we disclose the successful implementation of this strategy through the development of a direct allylic C–H functionalization of a variety of enol silyl ethers with electron-deficient olefins under hybrid catalysis of an iridium-based photosensitizer and pyridine derivative with the irradiation of visible light. The utility of this unique C–H alkylation protocol is also demonstrated.

## Results

### Analysis of the property of enol silyl ether and its radical cation

**Fig. 2:**
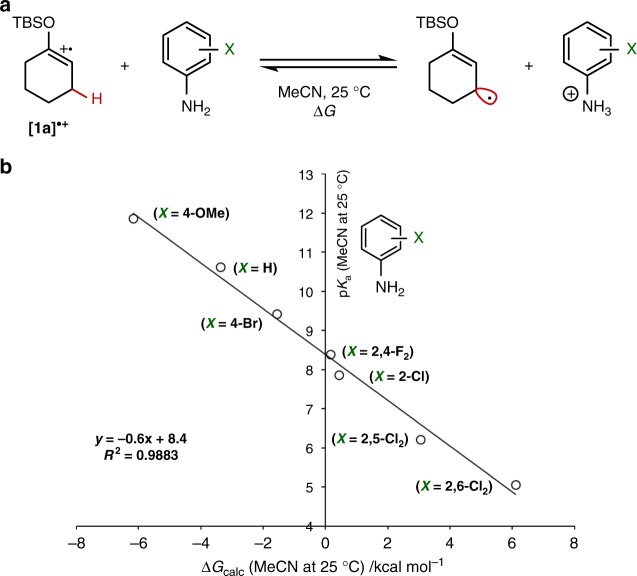
Estimation of p*K*_a_ of radical cation [**1a**]^•+^**. a** Acid–base equilibria between [**1a**]^•+^ and substituted anilines. **b** Linear free energy plot for the acid–base equilibria

We selected the cyclohexanone-derived enol silyl ether **1a** as a model substrate and investigated the physical properties requisite to establish a catalytic system for effecting the target allylic functionalization. First, the oxidation potential (*E*_ox_) of **1a** was determined to be 1.52 V vs. saturated calomel electrode (SCE) by square wave voltammetry measurement in MeCN, which led us to use a Ir^III^ photocatalyst bearing ligands suitable for imparting sufficient single-electron oxidation ability to the visible light-excited *Ir^III^ to generate a radical cation from **1a**. Second, the p*K*_a_ value of the resulting radical cation [**1a**]^•+^ in MeCN was estimated from the calculated reaction free energy (Δ*G*) of deprotonation process. Since the direct determination of p*K*_a_ from the calculated free energy is liable to give deviated value, we conducted statistical manipulation using experimentally available values. Specifically, the p*K*_a_ of [**1a**]^•+^ was obtained by considering acid–base equilibria with a series of substituted anilines (Fig. 2), whose p*K*_a_ values were experimentally determined (5–12 in MeCN)^32^. As shown in Fig. 2, the values for the reaction free energy calculated at the SMD(MeCN)-(U)CAM-B3LYP/6-311 + G(d,p) level and the experimental p*K*_a_ values of anilines showed excellent linear correlation. The intercept of this plot corresponded to the p*K*_a_ of [**1a**]^•+^ and was determined to be 8.4, which is close to that of TsOH [p*K*_a_(MeCN) = 8.6]^33^, meaning that [**1a**]^•+^ can be readily deprotonated by an appropriate Brønsted base. In view of structural tunability, we sought to employ an organic base with recognition that essential requirements for ensuring the function of the base are not only the p*K*_a_ value but also the redox potential not to be involved in the single-electron redox processes operated by the photocatalyst.

### Design of catalysis and optimization of reaction conditions

Based on these initial results and considerations, we began to evaluate the viability of our strategy in the reaction of **1a** with benzalmalononitrile (**2a**) as an acceptor in the presence of [Ir(dF(CF_3_)ppy)_2_(4,4′-dCF_3_bpy)]PF_6_ (**4a**, 2 mol%) (*E_1/2_^red^ = 1.65 V vs. SCE)^34^ and 2,4,6-collidine (1 equiv) (p*K*_a_ = 14.98 in MeCN, *E*_1/2_^ox^ =  ≥2.0 V vs. SCE)^32^ in MeCN at ambient temperature. Under the irradiation of a blue light-emitting diode (LED), bond formation occurred at the allylic carbon (β-position to the latent carbonyl) to give the alkylated enol silyl ether **3a** in 78% nuclear magnetic resonance (NMR) yield (Table 1, entry 1). The nature of the Brønsted base was of critical importance as the use of the sterically demanding 2,6-di-tert-butyl-4-methylpyridine (DTBMP) substantially decreased the yield of **3a** and inorganic bases, such as potassium phosphate, were totally ineffective (entries 2 and 3). Furthermore, attempted alkylations with Ir^III^ complexes **4b** or **4c**, having different bipyridine ligands, resulted in low conversion or no reaction, respectively, indicating that the reduction potential of the Ir^III^ complex in the excited state as well as the oxidation potential of the transient Ir^II^ complex have a significant impact on reaction efficiency (entries 4 and 5). Interestingly, solvent screening revealed that 1,2-dichloroethane (DCE) was optimal, allowing the reaction to proceed with higher efficiency under the influence of **4a** and 2,4,6-collidine to afford **3a** in 88% yield (entry 6). Although the catalytic use of the Brønsted base (10 mol%) appeared feasible, slight decrease in the product yield was inevitable because of undesired desilylated ketone formation (entry 7). This problem was overcome by performing the reaction at higher substrate and catalyst concentration; desilylation was completely suppressed and **3a** was isolated almost quantitatively (entry 8). The direct allylic C–H alkylation protocol thus developed was scalable as demonstrated by the reaction of 0.42 g of **1a** with **2a**, which proceeded smoothly with a reduced amount of **4a** (0.5 mol%) but with increased light intensity under otherwise identical conditions to yield 0.71 g of **3a** (96%) (entry 9).

**Table 1:** Optimization of conditions for reaction of enol silyl ether **1a** with benzalmalononitrile (**2a**)


Entry	4	Base (mol%)	d.r.^a^	Yield (%)^b^
1	**4a**	2,4,6-collidine (100)	1.7:1	78
2	**4a**	DTBMP (100)	1.7:1	27
3	**4a**	K_3_PO_4_ (100)	–	0
4	**4b**	2,4,6-collidine (100)	1.7:1	14
5	**4c**	2,4,6-collidine (100)	–	0
6^c^	**4a**	2,4,6-collidine (100)	2.0:1	89
7^c^	**4a**	2,4,6-collidine (10)	1.9:1	78
8^d^	**4a**	2,4,6-collidine (10)	2.0:1	95 (98)
9^e^	**4a**	2,4,6-collidine (10)	1.8:1	(96)

Unless otherwise noted, the reactions were performed with **1a** (0.1 mmol), **2a** (1.2 equiv), base and **4** (2 mol%) in MeCN (1.0 mL) at 25 °C for 12 h under argon atmosphere with light irradiation (blue LED, 750 W m^−2^)

^*1*^*H NMR* proton nuclear magnetic resonance, *d.r.* diastereomeric ratios, *LED* light-emitting diode, *DTBMP* 2,6-di-*tert*-butyl-4-methylpyridine, *TBS*  *t*-butyldimethylsilyl, *DCE* 1,2-dichloroethane

^a^Determined by ^1^H NMR from crude reaction mixture

^b^NMR yield with mesitylene as an internal standard. The value within parentheses is an isolated yield

^c^The reaction was conducted in DCE (1.0 mL)

^d^In DCE (0.5 mL)

^e^Carried out with **1a** (2.0  mmol, 0.42 g), **2a** (1.1 equiv), 2,4,6-collidine (10 mol%) and **4a** (0.5 mol%) in DCE (10 mL) at 25  °C for 18 h under argon atmosphere with light irradiation (blue LEDs, total 5000 W m^−2^)

To gain an insight into the reaction mechanism, especially the pathway for the generation of the reactive intermediate, Stern–Volmer luminescence quenching experiments were performed. Benzalmalononitrile (**2a**) and 2,4,6-collidine did not quench the excited state of photocatalyst **4a** (Supplementary Fig. 3). In contrast, increasing the concentration of enol silyl ether **1a** caused a significant decrease in emission intensity. These observations confirmed that the allylic C–H alkylation was initiated by the single-electron oxidation of **1a**.

### Investigation of substrate scope

**Fig. 3:**
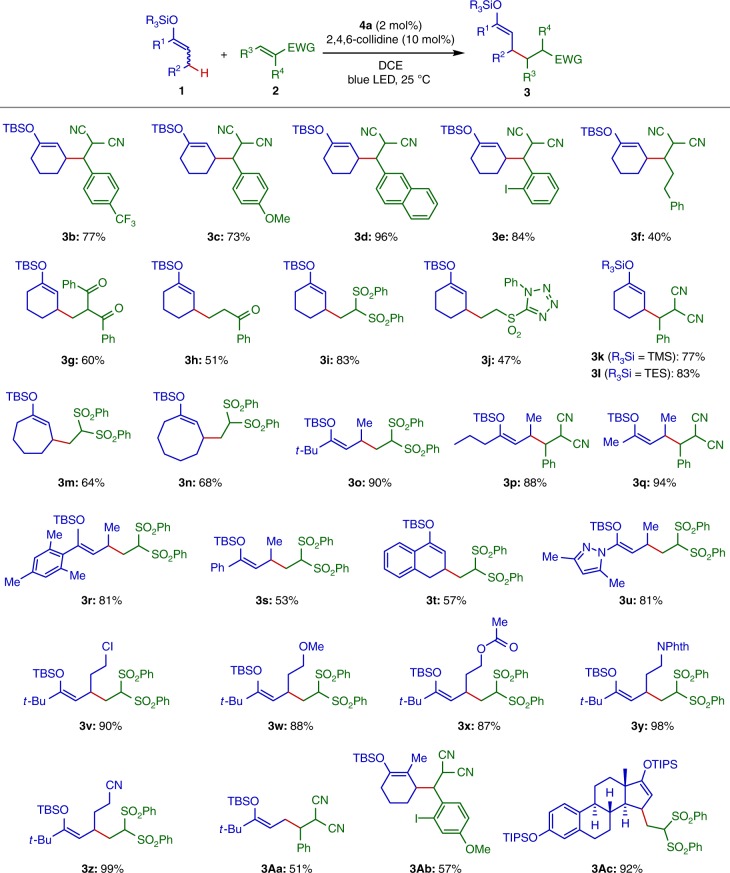
Substrate scope. Isolated yields are shown. Diastereomeric ratios (d.r.) of the products with consecutive two stereocenters were 1.5:1–2.0:1. See the Supplementary Information for details

Having established the optimized reaction conditions, we explored the scope of this alkylation under photoredox-Brønsted base hybrid catalysis (Fig. 3). As demonstrated in the reactions of **1a**, a range of arylidene malononitriles were employable as electrophilic acceptors and the corresponding alkylated enol silyl ethers **3b**–**3e** were obtained in uniformly good yield. Alkylidene malononitrile could also be coupled with **1a** to form **3f** with moderate efficiency. Furthermore, α,β-unsaturated ketones and sulfones proved viable acceptors to furnish **3g**–**3j** in moderate to good yield. With respect to enol silyl ether nucleophiles, it is important to note that trimethylsilyl and triethylsilyl derivatives underwent deprotonation–allylic alkylation to afford **3k** and **3l** in high yield, clearly indicating that the selectivity of deprotonation over desilylation does not rely on the steric demand of the silyl groups. In addition to the cyclohexanone derivatives, seven- and eight-membered cyclic enol silyl ethers smoothly reacted with 1,1-bis(phenylsulfonyl)ethylene to give good yields of **3m** and **3n**. The acyclic enol silyl ethers were also found to be suitable substrates; *primary*-alkyl ketone- and mesityl ketone-derived enol silyl ethers gave rise to the desired products **3o**–**3r** in excellent yield, whereas the phenyl ketone-derived substrate provided **3s** in lower yield (see below for theoretical investigations and associated discussion). Relatively low reactivity was also observed in the reaction of the α-tetralone derivative, resulting in the formation of **3t** in 57% yield. Notably, butyryl pyrazole-derived ketene silyl hemiaminal was amenable to alkylation and afforded **3u** in high yield. Enol silyl ethers with additional common functional groups, such as chlorine, ether, ester, imide and nitrile, were compatible with this protocol and could be converted into the corresponding alkylated enol silyl ethers **3v**–**3z** with equally high efficiency. When the ethyl *t*-butyl ketone-derived enol silyl ether was subjected to the optimized conditions, the C–C bond formation occurred at the terminal, *primary* allylic carbon to yield **3Aa**. On the other hand, 2-methylcyclohexanone-derived enol silyl ether was alkylated predominantly on the *secondary* allylic carbon to give **3Ab**. An additional noteworthy aspect of this hybrid catalytic system was that it accommodated complex substrate setting; the allylic alkylation of the estrone derivative featuring a fused polycyclic framework delivered the enol silyl ether **3Ac** in excellent yield.

### Analysis to rationalize the reactivity difference

The reactivity profile of the enol silyl ethers, particularly that observed with **1o** (R^1^ = *t-*Bu), **1r** (R^1^ = 2,4,6-Me_3_C_6_H_2_) and **1s** (R^1^ = Ph), prompted us to conduct density functional theory calculations at the CAM-B3LYP/6-311 + G(d, p) level for these substrates in order to rationalize the differences in reactivity (Table 2). The p*K*_a_ values of the corresponding radical cations in MeCN were calculated to be 8.4 (**1o**), 6.5 (**1r**) and 4.8 (**1s**), respectively (Supplementary Figs. 6–11). These values suggested that the deprotonation process to generate an allylic radical would be much easier for the **1s**-derived radical cation ([**1s**]^•+^) than for the others, albeit the reaction of **1s** provided the lowest yield (**3s**, 53%), indicating that acidity of the radical cation was not the sole factor governing the present radical addition reaction. We then calculated the singly occupied molecular orbital (SOMO) levels and spin density of **1o**-, **1r**- and **1s**-derived allylic radicals as well as the activation barrier of their additions to 1,1-bis(phenylsulfonyl)ethylene (Δ*G*^*‡*^), respectively, and the results are summarized in Table 2. According to the frontier orbital theory, the reactivity of a radical generally depends on both the SOMO level and spin density at the reactive site. Considering almost the same SOMO level (=−5.77 eV), the reactivity difference between the three allylic radicals, [**1o**]^•^, [**1r**]^•^ and [**1s**]^•^, would stem from differences in the spin density at the allylic C3 position. For [**1o**]^•^, from which the reaction proceeded with the lowest Δ*G*^*‡*^ of 21.2 kcal mol^−1^ to afford high yield of the product **3o** (90%), a relatively large spin density (=0.70) was located at the allylic position. Although the larger spin density was estimated to be at the C1 position (=0.75), alkylation at this carbon would be highly unfavorable due to steric congestion, and in fact, the corresponding product was not detected experimentally. In contrast, the electron in the radical [**1s**]^•^ can be delocalized over the phenyl group, decreasing the spin density at the C3 position (=0.51) compared to that of [**1o**]^•^. The difference in spin density was clearly reflected in the larger activation barrier (Δ*G*^*‡*^ = 23.3 kcal mol^−1^) and lower experimental yield of the product **3s** (53%). The importance of spin localization for ensuring productive bond formation was also corroborated by the similar analysis with **1r** in comparison to the outcome with **1s**. Radical conjugation with the mesityl group is partially inhibited in [**1r**]^•^ because of the torsional relationship between the allyl and mesityl moieties (Supplementary Fig. 12); this leads to a higher spin density (=0.64) at the C3 position and lower activation barrier (Δ*G*^*‡*^ = 21.6 kcal mol^−1^), thereby accounting for the experimentally observed higher efficiency in the formation of the product **3r** (81% yield).

**Table 2:** Summary of spin density and SOMO level of the radicals [**1**]^•^ and activation barrier of their addition to 1,1-bis(phenylsulfonyl)ethylene


[1]^•^	Spin density		SOMO^a^ (eV)	Δ*G*^*‡*b^ (kcal mol^−1^)	Yield (%) of the reaction
	C1	C3			
**[1o]** ^**•**^	0.75	0.70	−5.77	21.2	90
**[1r]** ^**•**^	0.62	0.64	−5.77	21.6	81
**[1s]** ^**•**^	0.58	0.51	−5.77	23.3	53

*SOMO* singly occupied molecular orbital

^a^Calculated at (U)CAM-B3LYP/6-311 + G(d, p)

^b^Values for Gibbs free energy (298.15 K and 1 atm) were calculated at SMD(DCE)-(U)CAM-B3LYP/6-311 + G(d, p)

### Transformation of alkylated enol silyl ethers

**Fig. 4:**
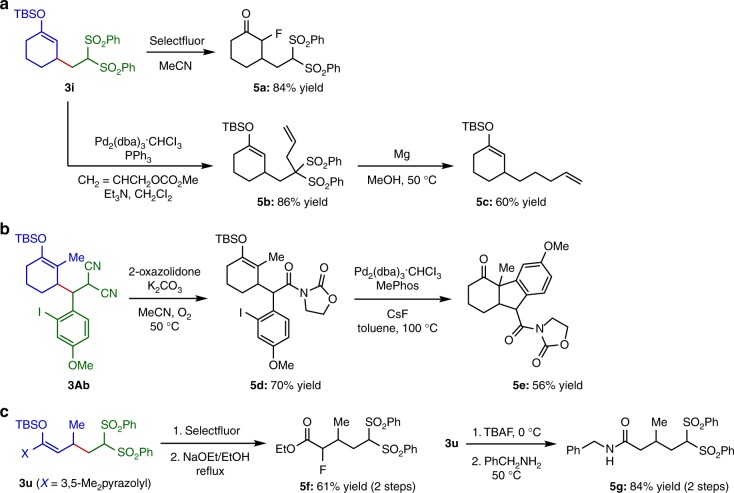
Diverse transformations of alkylated products. **a** Derivatizations of alkylated enol silyl ether **3i**. **b** Synthesis of complex fused tricyclic ketone **5e** from alkylated enol silyl ether **3Ab**. **c** Derivatizations of alkylated ketene silyl hemiaminal **3u**

The alkylation products have two different reactive sites, the enol silyl ether and active methine or methylene. This salient structural feature enables diverse transformations to access a variety of complex carbonyl compounds, providing a powerful demonstration of the utility of this method (Fig. 4). For instance, treatment of the alkylated enol silyl ether **3i** with Selectfluor facilitated smooth fluorination of the enol silyl ether component to produce the α-fluoro-β-alkylated ketone **5a**. On the other hand, selective functionalization of the bis(sulfonyl)methyl moiety of **3i** was feasible by the palladium-catalyzed allylation with allyl carbonate, affording **5b** in high yield. Subsequent exposure of **5b** to magnesium metal in MeOH effected desulfonylation to give **5c**. The malononitrile subunit of the enol silyl ether **3Ab** could be selectively converted into imide by reaction with 2-oxazolidone under oxygen atmosphere^35^. The resulting **5d** underwent intramolecular arylation under palladium catalysis to furnish the fused tricyclic ketone **5e**, of which structure is often found in diterpenoids, such as taiwaniaquinol A^36^. Moreover, functionalized esters and amides, such as **5f** and **5g**, were accessible through the common derivatizations of pyrazole-substituted ketene silyl hemiaminal **3u**; the reaction with Selectfluor followed by solvolysis in ethanol gave rise to α-fluoro-β-alkylated ester **5f**, while desilylation and subsequent condensation with benzylamine proceeded with high efficiency to yield **5g**.

We have developed a strategy for the allylic C–H alkylation of enol silyl ethers and their derivatives, which relies heavily on the combined use of appropriate photoredox and Brønsted base catalysts for the generation of requisite allylic radicals while suppressing undesired desilylation process. Under the hybrid catalysis, a series of enol silyl ethers smoothly react with electron-deficient olefins to give the corresponding functionalized enol silyl ethers. This operationally simple protocol, in concert with the ready availability of enol silyl ethers and their conventional polar reactivity, provides rapid and reliable access to an array of complex carbonyl compounds and will find widespread use among practitioners of organic synthesis.

## Methods

### Representative procedure for allylic alkylation

To a flame-dried test tube were added **2a** (18.5 mg, 0.12 mmol), [Ir(dFCF_3_ppy)_2_(4,4′-dCF_3_bpy)]PF_6_ (2.29 mg, 0.002 mmol, 2 mol%) and DCE (0.5 mL, 0.2 M). The reaction tube was sealed with a rubber septum and then evacuated in vacuo and backfilled with Ar five times. **1a** (21.2 mg, 0.1 mmol) and 2,4,6-collidine (1.3 μL, 0.01 mmol, 10 mol%) were successively introduced. The whole reaction mixture was stirred at 25 °C under the irradiation of blue LED (448 nm, 750 W m^−2^) with a fan to keep the temperature. After 12 h, the reaction mixture was evaporated. Purification of the resulting crude residue by column chromatography on silica gel (hexane 100% to hexane/EtOAc = 5:1) afforded **3a** in 98% yield.

## Supplementary information

Supplementary Information

Description of Additional Supplementary Files

Supplementary Data 1

## Data Availability

All relevant data are available on request from the authors.
