# Supplementary material for: Direct allylic C–H alkylation of enol silyl ethers enabled by photoredox–Brønsted base hybrid catalysis
Source: Nat Commun. 2019 Jun 20;10:2706. doi: 10.1038/s41467-019-10641-y (PMC6586846; doi:10.1038/s41467-019-10641-y)
Supplement: Supplementary file 1 — Supplementary Information [file 41467_2019_10641_MOESM1_ESM.pdf]

Supplementary Information

Direct Allylic C–H Alkylation of Enol Silyl Ethers Enabled by Photoredox–Brønsted Base Hybrid Catalysis

K. Ohmatsu, *et al.*

Supplementary Methods:

General Information: ^{1}H NMR spectra were recorded on a JEOL JNM-ECS400 (400 MHz) and JEOL JNM-ECA600II (600 MHz) spectrometer. Chemical shifts are reported in ppm from the tetramethylsilane (0.0 ppm) resonance as the internal standard ($CDCl_3$). Data are reported as follows: chemical shift, integration, multiplicity (s = singlet, d = doublet, t = triplet, q = quartet, quin = quintet, sext = sextet, sept =septet, m = multiplet, and br = broad) and coupling constants (Hz). ^{13}C NMR spectra were recorded on a JEOL JNM-ECA600II (151 MHz) spectrometer with complete proton decoupling. Chemical shifts are reported in ppm from the solvent resonance as the internal standard ($CDCl_3$; 77.16 ppm). The high-resolution mass spectra were conducted on Thermo Fisher Scientific Exactive Plus (ESI). Analytical thin layer chromatography (TLC) was performed on Merck precoated TLC plates (silica gel 60 GF254, 0.25 mm). Flash column chromatography was performed on Silica gel 60 N (spherical, neutral, 40~50μm; Kanto Chemical Co., Inc.).

All air- and moisture-sensitive reactions were performed under an atmosphere of argon (Ar) in dried glassware. Dichloromethane (CH_2Cl_2), 1,2-dichloroethane (DCE), diethyl ether (Et_2O), and tetrahydrofuran (THF) were supplied from Kanto Chemical Co., Inc. as “Dehydrated” and further purified by both A2 alumina and Q5 reactant using a GlassContour solvent dispensing system. The photocatalysts (**4a-4c**) and enol silyl ethers were synthesized according to the previously reported procedures.[1-4] Other simple chemicals were purchased and used as such.

Synthesis of Ketones

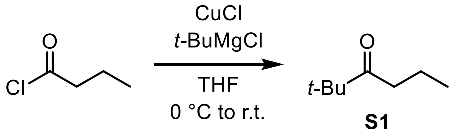

S1

To a solution of butyryl chloride (3.1 mL, 30 mmol) and CuCl (0.15 g, 1.5 mmol) in THF (60 mL, 0.5 M), a 1 M THF solution of *t*-BuMgCl (31 mL, 31 mmol) was added dropwise via syringe under Ar atmosphere. The resulting mixture was allowed to warm to room temperature and stirred overnight. The reaction was then quenched by the addition of water at 0 °C and the aqueous phase was extracted with Et_2O twice. The combined organic phases were washed with 1 N aqueous solution of HCl three times, dried over Na_2SO_4, filtered, and concentrated. The residue was purified by distillation to afford **S1** in 57% yield (2.2 g, 17 mmol). ^{1}H NMR (400 MHz, $CDCl_3$) δ 2.46 (2H, t, J = 6.8 Hz), 1.63-1.54 (2H, m), 1.13 (9H, s), 0.90 (3H, t, J = 7.2 Hz).

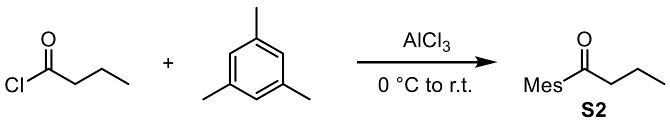

S2

To a round-bottom flask was charged with mesitylene (8.4 mL, 60 mmol) and the reaction flask was cooled to 0 °C. Anhydrous $AlCl_3$ (0.67 g, 5 mmol) was added one-portion and the mixture was stirred at the same temperature for several minutes. To this mixture was added butyryl chloride (2.1 mL, 20 mmol) dropwise over 1 h at 0 °C. The whole reaction mixture was then allowed to warm to room temperature and stirred for 6 h. The reaction was quenched by the addition of water and the extractive work-up was conducted with Et_2O. The combined organic layers were washed with saturated aqueous solution of $NaHCO_3$, dried over Na_2SO_4, filtered, and concentrated. The resulting crude material was purified by column chromatography on silica gel (hexane 100% to hexane/EtOAc = 5:1 as eluent) to afford **S2** in 99% yield (3.7 g, 19.4 mmol). ^{1}H NMR (400 MHz, $CDCl_3$) δ 6.83 (2H, s), 2.67 (2H, t, J = 7.6 Hz), 2.27 (3H, s), 2.19 (6H, s), 1.73 (2H, sext, J = 7.6 Hz), 1.00 (3H, t, J = 7.6 Hz).

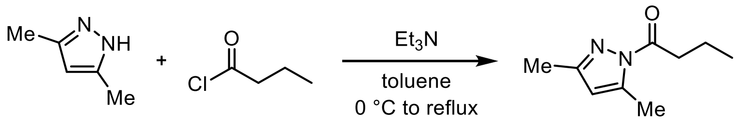

S3

To a two-necked round-bottom flask was added 3,5-dimethylpyrazole (0.96 g, 10 mmol), butyryl chloride (1.2 mL, 11 mmol), toluene (50 mL, 0.2 M) and the flask was degassed by alternating vacuum evacuation/Ar backfill. The mixture was cooled to 0 °C, and Et_3N (2.8 mL, 20 mmol) was added. The whole mixture was then heated to reflux. After stirring for 1 h, the reaction mixture was cooled to room temperature and filtered through a pad of Celite with the aid of EtOAc. The filtrates were evaporated under reduced pressure and the crude product was purified by column chromatography on silica gel (hexane/EtOAc = 10:1 as eluent) to afford **S3** quantitatively (1.7 g, 10 mmol). ^{1}H NMR (400 MHz, $CDCl_3$) δ 5.95 (1H, s), 3.08 (2H, t, J = 7.6 Hz), 2.54 (3H, s), 2.24 (3H, s), 1.77 (2H, sext, J =7.6 Hz), 1.02 (3H, t, J = 7.6 Hz).

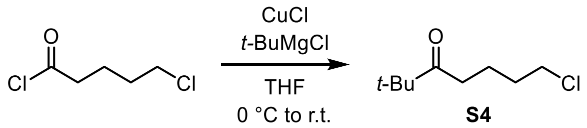

To a solution of 5-chlorovaleryl chloride (7.7 mL, 60 mmol) and CuCl (5.9 g, 60 mmol) in THF (60 mL, 1 M), a 1 M THF solution of *t*-BuMgCl (63 mL, 63 mmol) was added dropwise via syringe under Ar atmosphere at 0 °C. The reaction mixture was then allowed to warm to room temperature and stirred overnight. The reaction was quenched with ice water and filtered through a pad of Celite. The organic layer of resulting filtrates was washed with 1 N HCl aq. three times, dried, and concentrated. The crude product was purified by silica gel column chromatography (hexane/EtOAc = 15:1 as eluent) to give **S4** in 92% yield (9.75 g, 55.2 mmol). ^{1}H NMR (400 MHz, $CDCl_3$) δ 3.53 (2H, t, J = 6.4 Hz), 2.52 (2H, t, J = 6.8 Hz), 1.85-1.65 (4H, m), 1.14 (9H, s).

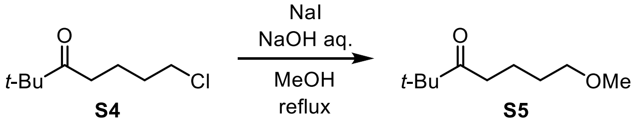

The mixture of **S4** (0.53 g, 3 mmol) and NaI (0.90 g, 6 mmol) in 1 N NaOH aq. (20 mL) and MeOH (100 mL) was refluxed with stirring for 12 h. The reaction mixture was then cooled to room temperature and evaporated to remove MeOH. The resulting mixture was extracted with EtOAc twice, and the combined organic layers were washed with brine, dried over Na_2SO_4, filtered, and concentrated. The residue was purified by column chromatography on silica gel (hexane/EtOAc = 15:1) to afford **S5** in 54% yield (0.28 g, 1.63 mmol). ^{1}H NMR (400 MHz, $CDCl_3$) δ 3.37 (2H, t, J = 6.4 Hz), 3.32 (3H, s), 2.51 (2H, t, J = 7.0 Hz), 1.66-1.50 (4H, m), 1.13 (9H, s).

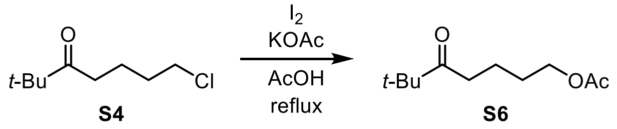

The solution of **S4** (2.6 g, 15 mmol), KOAc (2.9 g, 30 mmol), and I_2 (31 mg, 0.12 mmol) in AcOH (150 mL, 0.1 M) was refluxed with stirring overnight. The reaction mixture was allowed to cool to room temperature and acetic acid was removed by rotary evaporator. The residue was diluted with water and extracted with EtOAc. The combined organic layers were washed with brine, dried over Na_2SO_4, filtered, and concentrated. The crude material was purified by column chromatography on silica gel (hexane/EtOAc = 10:1) to afford **S6** in 60% yield (1.8 g, 9.0 mmol). ^{1}H NMR (400 MHz, $CDCl_3$) δ 4.06 (2H, t, J = 6.2 Hz), 2.52 (2H, t, J = 7.0 Hz), 2.05 (3H, s), 1.62 (4H, m), 1.14 (9H, s).

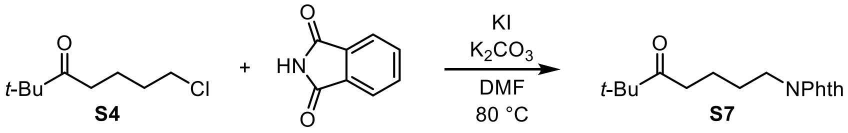

The suspension of **S4** (0.88 g, 5 mmol), phthalimide (0.88 g, 6 mmol), K_2CO_3 (1.38 g, 10 mmol), and KI (0.33 g, 2 mmol) in DMF (20 mL) was stirred at 80 °C overnight. After cooling to room temperature, the reaction

mixture was diluted with water and the extractive work-up was performed with EtOAc. The combined organic layers were washed with water three times, dried over Na_2SO_4, and concentrated. The resulting crude residue was purified by column chromatography on silica gel (hexane/EtOAc = 3:1) to afford **S7** in 90% yield (1.3 g, 4.5 mmol). ^{1}H NMR (400 MHz, $CDCl_3$) δ 7.87-7,81 (2H, m), 7.74-7.68 (2H, m), 3.69 (2H, t, J = 7.2 Hz), 2.54 (2H, t, J = 7.2 Hz), 1.74-1.55 (4H, m), 1.13 (9H, s).

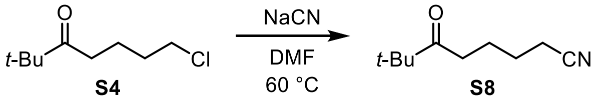

The mixture of **S4** (0.49 g, 2.8 mmol) and NaCN (0.41 g, 8.3 mmol) in DMF (3 mL) was vigorously stirred at 60 °C for 10 h. After cooling to room temperature, the reaction mixture was diluted with water and the extractive work-up was conducted with EtOAc. The combined organic layers were washed with water three times, dried over Na_2SO_4, filtered, and concentrated. The crude material was purified by column chromatography on silica gel (hexane/EtOAc = 10:1 to 4:1) to furnish **S8** quantitatively (0.47 g, 2.8 mmol). ^{1}H NMR (400 MHz, $CDCl_3$) δ 2.54 (2H, t, J = 6.8 Hz), 2.35 (2H, t, J = 7.2 Hz), 1.79-1.59 (4H, m), 1.14 (9H, s).

Characterization of Enol Silyl Ethers

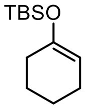

^{1}H NMR (400 MHz, $CDCl_3$) δ 4.89-4.84 (1H, brm), 2.03-1.96 (4H, m), 1.69-1.61 (2H, m), 1.53-1.47 (2H, m), 0.91 (9H, s), 0.12 (6H, s); ^{13}C NMR (151 MHz, $CDCl_3$) δ 150.6, 104.5, 30.0, 25.9, 24.0, 23.3, 22.5, 18.2, –4.2.

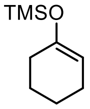

^{1}H NMR (400 MHz, $CDCl_3$) δ 4.88-4.85 (1H, m), 2.03-1.96 (4H, m), 1.68-1.62 (2H, m), 1.53-1.48 (2H, m), 0.18 (9H, s); ^{13}C NMR (151 MHz, $CDCl_3$) δ 150.4, 104.4, 30.0, 24.0, 23.3, 22.5, 0.5.

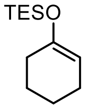

^{1}H NMR (400 MHz, $CDCl_3$) δ 4.89-4.83 (1H, m), 2.04-1.96 (4H, m), 1.68-1.60 (2H, m), 1.54-1.46 (2H, m), 0.97 (9H, t, J = 8.0 Hz), 0.64 (6H, q, J = 8.0 Hz); ^{13}C NMR (151 MHz, $CDCl_3$) δ 150.6, 104.1, 30.0, 24.0, 23.4, 22.5, 6.9, 5.2.

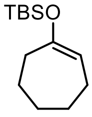

^{1}H NMR (400 MHz, $CDCl_3$) δ 5.01 (1H, t, J = 6.4 Hz), 2.26-2.19 (2H, brm), 1.98 (2H, dd, J = 11.6, 6.4 Hz), 1.71-1.63 (2H, m), 1.61-1.48 (4H, m), 0.91 (9H, s), 0.11 (6H, s); ^{13}C NMR (151 MHz, $CDCl_3$) δ 156.3, 108.7, 35.7, 31.6, 27.9, 25.9, 25.5, 25.4, 18.1, –4.3.

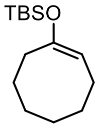

^{1}H NMR (400 MHz, $CDCl_3$) δ 4.74-4.66 (1H, m), 2.20-2.14 (2H, brm), 2.04-1.96 (2H, br), 1.61-1.45 (8H, brm), 0.91 (9H, s), 0.14 (6H, s); ^{13}C NMR (151 MHz, $CDCl_3$) δ 153.4, 105.0, 31.1(9), 31.1(5), 27.9, 26.5(2), 26.5(0), 25.9, 25.7, 18.2, –4.2.

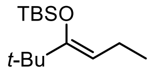

^{1}H NMR (400 MHz, $CDCl_3$) δ 4.44 (1H, t, J = 7.2 Hz), 1.99 (2H, quin, J = 7.2 Hz), 1.06 (9H, s), 0.98 (9H, s), 0.93 (3H, t, J = 7.2 Hz), 0.17 (6H, s); ^{13}C NMR (151 MHz, $CDCl_3$) δ 157.3, 105.5, 29.1, 26.7, 25.9, 19.5, 19.4, 14.9, –2.9.

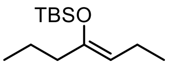

^{1}H NMR (400 MHz, $CDCl_3$) δ 4.40 (1H, t, J = 7.4 Hz), 2.06-1.90 (4H, m), 1.52-1.46 (2H, m), 0.95 (9H, s), 0.93-0.88 (6H, m), 0.12 (6H, s); ^{13}C NMR (151 MHz, $CDCl_3$) δ 149.7, 110.0, 38.8, 26.0, 20.5, 18.7, 18.4, 14.7, 13.8, –3.9.

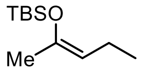

This compound was synthesized by the literature method.[5]

^{1}H NMR (400 MHz, $CDCl_3$) δ 4.93 (1H, t, J = 7.2 Hz), 1.97-2.04 (2H, m), 1.76 (3H, s), 0.94 (9H, s), 0.91 (3H, t, J = 7.8 Hz), 0.13 (6H, s); ^{13}C NMR (151 MHz, $CDCl_3$) δ 146.2, 110.7, 25.9, 22.9, 18.8, 18.4, 14.6, –3.8.

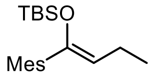

^{1}H NMR (400 MHz, $CDCl_3$) δ 6.81 (2H, s), 4.52 (1H, t, J = 7.2 Hz), 2.28 (6H, s), 2.25 (3H, s), 2.21 (2H, quin, J = 7.2 Hz), 1.01 (3H, t, J = 7.2 Hz), 0.91 (9H, s), –0.21 (6H, s); ^{13}C NMR (151 MHz, $CDCl_3$) δ 145.8, 136.9, 136.5, 136.4, 128.2, 115.0, 25.8, 21.2, 20.4, 18.8, 18.4, 14.5, –4.4.

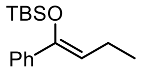

^{1}H NMR (400 MHz, $CDCl_3$) δ 7.43 (2H, d, J = 6.8 Hz), 7.31-7.25 (3H, m), 5.09 (1H, t, J = 7.6 Hz), 2.22 (2H, quin, J = 7.6 Hz), 1.03 (3H, t, J = 7.6 Hz), 0.98 (9H, s), –0.05 (6H, s); ^{13}C NMR (151 MHz, $CDCl_3$) δ 148.9, 140.0, 128.0, 127.4, 126.0, 114.0, 26.0, 19.6, 18.5, 14.4, –3.9.

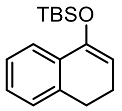

^{1}H NMR (400 MHz, $CDCl_3$) δ 7.46 (1H, d, J = 7.2 Hz), 7.22-7.08 (3H, m), 5.17 (1H, t, J = 4.6 Hz), 2.76 (2H, t, J = 8.0 Hz), 2.35-2.27 (2H, m), 1.01 (9H, s), 0.20 (6H, s); ^{13}C NMR (151 MHz, $CDCl_3$) δ 148.4, 137.3, 133.8, 127.4, 127.1, 126.3, 122.0, 105.1, 28.4, 26.1, 22.4, 18.5, –2.8.

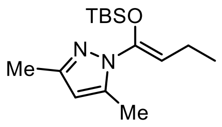

^{1}H NMR (400 MHz, $CDCl_3$) δ 5.79 (1H, s), 4.76 (1H, t, J = 7.6 Hz), 2.23 (3H, s), 2.21 (3H, s), 2.18 (2H, quin, J = 7.6 Hz), 1.03 (3H, t, J = 7.6 Hz), 0.92 (9H, s), –0.08 (6H, s); ^{13}C NMR (151 MHz, $CDCl_3$) 148.1, 141.3, 139.8, 109.3, 105.7, 25.7, 18.9, 18.2, 14.1, 13.6, 11.6, –5.2.

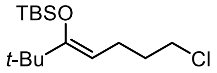

^{1}H NMR (400 MHz, $CDCl_3$) δ 4.43 (1H, t, J = 7.2 Hz), 3.53 (2H, t, J = 7.2 Hz), 2.13 (2H, q, J = 7.2 Hz), 1.79 (2H, quin, J = 7.2 Hz), 1.06 (9H, s), 0.98 (9H, s), 0.18 (6H, s); ^{13}C NMR (151 MHz, $CDCl_3$) δ 159.1, 101.7, 45.0, 36.7, 33.3, 29.1, 26.7, 23.8, 19.4, –2.7.

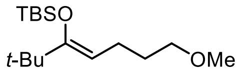

^{1}H NMR (400 MHz, $CDCl_3$) δ 4.45 (1H, t, J = 7.0 Hz), 3.36 (2H, t, J = 7.0 Hz), 3.32 (3H, s), 2.03 (2H, q, J = 7.0 Hz), 1.59 (2H, quin, J = 7.0 Hz), 1.05 (9H, s), 0.98 (9H, s), 0.17 (6H, s); ^{13}C NMR (151 MHz, $CDCl_3$) δ 158.3, 102.9, 72.7, 66.0, 58.7, 30.3, 29.1, 26.7, 22.7, 19.4, –2.8.

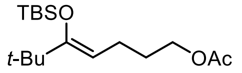

^{1}H NMR (400 MHz, $CDCl_3$) δ 4.44 (1H, t, J = 7.0 Hz), 4.06 (2H, t, J = 6.6 Hz), 2.07 (2H, q, J = 7.0 Hz), 2.04 (3H, s), 1.70-1.60 (2H, m), 1.05 (9H, s), 0.98 (9H, s), 0.18 (6H, s); ^{13}C NMR (151 MHz, $CDCl_3$) δ 171.4, 158.7, 102.3, 64.5, 36.7, 29.3, 29.0, 26.7, 22.8, 21.2, 19.4, –2.8.

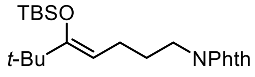

^{1}H NMR (400 MHz, $CDCl_3$) δ 7.86-7.80 (2H, m), 7.73-7.67 (2H, m), 4.47 (1H, brt, J = 6.6 Hz), 3.69 (2H, t, J = 7.6 Hz), 2.04 (2H, q, J = 7.6 Hz), 1.71 (2H, quin, J = 7.6 Hz), 1.04 (9H, s), 0.94 (9H, s), 0.15 (6H, s); ^{13}C NMR (151 MHz, $CDCl_3$) δ 168.5, 158.7, 134.0, 132.4, 123.3, 102.2, 37.9, 36.7, 29.2, 29.0, 26.6, 23.7, 19.3, –2.8.

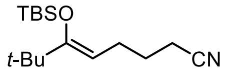

^{1}H NMR (400 MHz, $CDCl_3$) δ 4.41 (1H, t, J = 7.2 Hz), 2.32 (2H, t, J = 7.2 Hz), 2.13 (2H, q, J = 7.2 Hz), 1.69 (1H, quin, J = 7.2 Hz), 1.06 (9H, s), 0.98 (9H, s), 0.18 (6H, s); ^{13}C NMR (151 MHz, $CDCl_3$) δ 159.9, 120.0, 101.0, 36.8, 29.0, 26.6, 26.1, 25.2, 19.3, 16.7, –2.7.

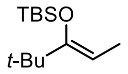

^{1}H NMR (400 MHz, $CDCl_3$) δ 4.57 (1H, q, J = 7.2 Hz), 1.53 (3H, d, J = 7.2 Hz), 1.06 (9H, s), 0.99 (9H, s), 0.18 (6H, s); ^{13}C NMR (151 MHz, $CDCl_3$) δ 159.1, 97.0, 31.7, 29.0, 26.7, 25.9, 12.0, –2.7.

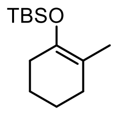

^{1}H NMR (600 MHz, $CDCl_3$) δ 2.04-1.99 (2H, m), 1.97-1.92 (2H, m), 1.67-1.61 (2H, m), 1.57 (3H, s), 1.56-1.51 (2H, m), 0.95 (9H, s), 0.11 (6H, s); ^{13}C NMR (151 MHz, $CDCl_3$) δ 143.1, 111.7, 30.5(4), 30.4(5), 26.3, 24.0, 23.2, 18.3, 16.6, –3.7.

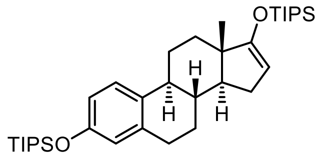

^{1}H NMR (400 MHz, $CDCl_3$) δ 7.08 (1H, d, J = 8.4 Hz), 6.64 (1H, d, J = 8.4 Hz), 6.59 (1H, s), 4.42 (1H, s), 2.90-2.74 (2H, m), 2.34-2.18 (2H, m), 2.21-2.03 (1H, m), 1.94-1.78 (3H, m), 1.64-1.31 (5H, m), 1.31-1.14 (6H, m), 1.10 (36H, br), 0.90 (3H, s); ^{13}C NMR (151 MHz, $CDCl_3$) δ 165.4, 153.8, 138.0, 133.4, 125.8, 119.9, 116.9, 97.4, 54.3, 45.3, 44.8, 37.3, 33.8, 29.7, 28.3, 27.3, 26.4, 18.2(3), 18.1(6), 15.7, 12.9, 12.7.

General Experimental Procedure for Allylic Alkylation of Enol Silyl Ethers

To a flame-dried test tube were added electron-deficient olefin (0.12 mmol, 1.2 equiv), [Ir(dFCF$_3$ppy)$_2$(4,4′-dCF$_3$bpy)]PF$_6$ (2.29 mg, 0.002 mmol, 2 mol%) and DCE (0.5 mL, 0.2 M). The reaction tube was sealed with a rubber septum and then evacuated in *vacuo* and backfilled with Ar five times. Enol silyl ether (0.1 mmol, 1 equiv) and 2,4,6-collidine (1.3 μL, 0.01 mmol, 10 mol%) were successively introduced. The whole reaction mixture was stirred at 25 °C under the irradiation of blue LED (448 nm, 750 W/m^2) with a fan to keep the temperature. After 12 h, the reaction mixture was evaporated. Purification of the resulting crude residue by column chromatography on silica gel (hexane 100% to hexane/EtOAc = 5:1) afforded the corresponding alkylated enol silyl ether.

Proposed Catalytic Cycle of Allylic C–H Alkylation

While it is difficult to precisely establish the entire reaction mechanism, particularly the closing step of the catalytic cycle, we illustrate the plausible mechanism of the present allylic alkylation of enol silyl ethers in Supplementary Figure 1.

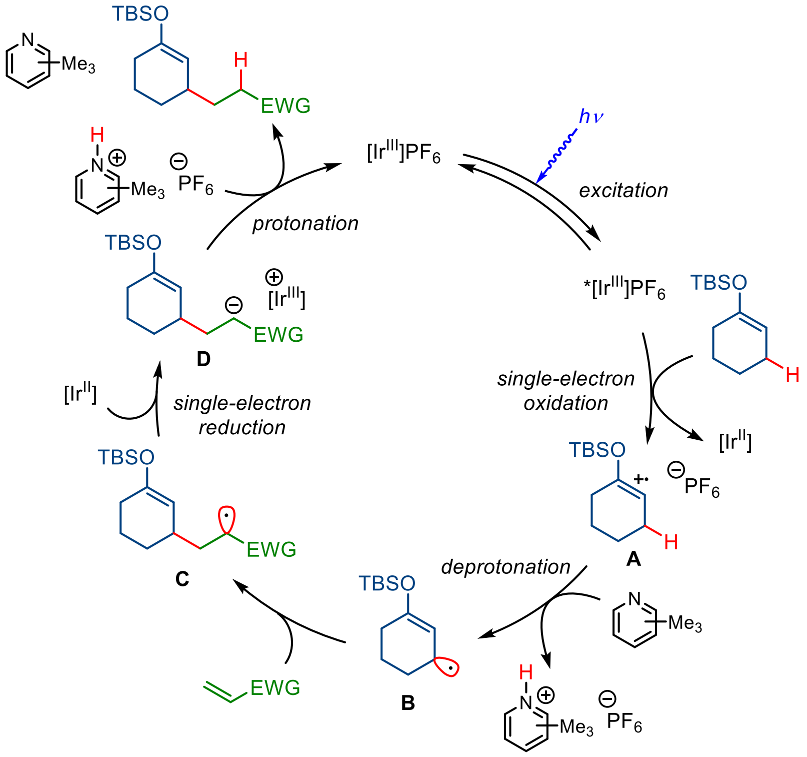

Supplementary Figure 1. Proposed catalytic cycle. The light-excited Ir^{III} complex would engage in the oxidation of enol silyl ether to generate radical cation intermediate **A** and the reduced Ir^{II} complex. This oxidation process would then be relayed to the deprotonation of the radical cation by 2,4,6-collidine to furnish nucleophilic allylic radical **B**. The subsequent radical addition to electron-deficient olefin could form a new carbon–carbon bond with the generation of electrophilic radical **C**. The single-electron reduction of **C** by Ir^{II} complex would give the carbanion intermediate **D**, which could be protonated by collidinium salt to afford the alkylated product and return both catalyst species back to the initial forms.

Characterization of Alkylated Enol Silyl Ethers

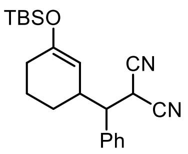

3a: ^{1}H NMR (600 MHz, $CDCl_3$) (for major diastereomer) δ 7.45-7.32 (5H, m), 4.35 (1H, br), 4.16 (1H, d, J = 4.8 Hz), 2.99 (1H, dd, J = 10.2, 4.8 Hz), 2.91-2.85 (1H, m), 2.02-1.94 (3H, m), 1.88-1.82 (1H, m), 1.70-1.61 (1H, m), 1.28-1.22 (1H, m), 0.82 (9H, s), –0.06 (3H, s), –0.07 (3H, s), (for minor diastereomer) δ 7.45-7.32 (5H, m), 4.94 (1H, d, J = 4.2 Hz), 4.24 (1H, d, J = 4.8 Hz), 3.00-2.94 (1H, m), 2.91-2.85 (1H, m), 2.05-2.01 (2H, m), 1.71-1.61 (1H, m), 1.61-1.55 (1H, m), 1.49-1.42 (1H, m), 1.22-1.17 (1H, m), 0.94 (9H, s), 0.17 (6H, s); ^{13}C NMR (151 MHz, $CDCl_3$) (for major diastereomer) δ 153.4, 136.6, 129.3, 128.7, 112.1, 111.9, 103.8, 52.1, 37.1, 29.6, 27.7, 27.6, 21.6, 18.1, –4.3, –4.5, (for minor diastereomer) δ 155.3, 136.7, 129.0, 128.4, 112.4, 112.0, 103.0, 52.0, 36.1, 30.0, 27.9, 25.8, 19.6, 18.2, –4.0, –4.2; HRMS (ESI) Calcd for $C_{22}H_{29}ON_2Si^-$ ([M−H]$^-$) 365.2044. Found 365.2052.

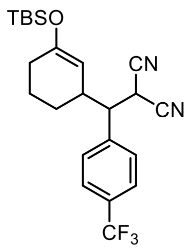

3b (dr = 1.8:1): ^{1}H NMR (600 MHz, $CDCl_3$) (for major diastereomer) *δ* 7.69 (2H, d, *J* = 8.4 Hz), 7.49 (2H, d, *J* = 8.4 Hz), 4.27 (1H, br), 4.18 (1H, d, *J* = 3.6 Hz), 3.02-2.95 (2H, m), 2.02-1.94 (3H, m), 1.89-1.82 (1H, m), 1.72-1.63 (1H, m), 1.29-1.21 (1H, m), 0.82 (9H, s), –0.07 (6H, s), (for minor diastereomer) *δ* 7.69 (2H, d, *J* = 7.5 Hz), 7.51 (2H, d, *J* = 7.5 Hz), 4.92 (1H, d, *J* = 4.2 Hz), 4.27 (1H, d, *J* = 4.8 Hz), 3.06 (1H, dd, *J* = 10.5, 4.8 Hz), 2.93-2.86 (1H, m), 2.04 (2H, t, *J* = 6.0 Hz), 1.70-1.56 (2H, m), 1.51-1.42 (1H, m), 1.18-1.12 (1H, m), 0.94 (9H, s), 0.17 (6H, s); ^{13}C NMR (151 MHz, $CDCl_3$) (for major diastereomer) *δ* 154.0, 140.5, 131.4 (q, $J_{C\text{-}F}$ = 32.8 Hz), 129.3, 126.3 (q, $J_{C\text{-}F}$ = 4.4 Hz), 123.9 (q, $J_{C\text{-}F}$ = 273.0 Hz), 111.7, 111.4, 103.1, 51.8, 37.0, 29.5, 27.5, 27.2, 25.7, 21.5, 18.1, –4.3, –4.5, (for minor diastereomer) *δ* 155.8, 140.6, 131.3 (q, $J_{C\text{-}F}$ = 33.2 Hz), 128.9, 126.3, 123.9 (q, $J_{C\text{-}F}$ = 272.0 Hz), 112.0, 111.5, 102.3, 51.7, 36.0, 29.9, 25.7, 25.5, 19.4, 18.2, –4.0, –4.2; HRMS (ESI) Calcd for $C_{23}H_{28}ON_2F_3Si^-$ ($[M-H]^-$) 433.1918. Found 433.1917.

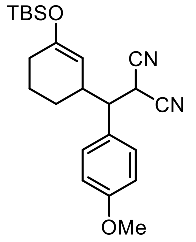

3c (dr = 1.9:1): The reaction of **1a** (0.1 mmol) with electron-deficient olefin (0.2 mmol, 2 equiv) was carried out for 24 h.

^{1}H NMR (600 MHz, $CDCl_3$) (for major diastereomer) *δ* 7.26 (2H, d, *J* = 9.0 Hz), 6.93 (2H, d, *J* = 9.0 Hz), 4.39 (1H, brs), 4.12 (1H, d, *J* = 4.8 Hz), 3.83 (3H, s), 2.96-2.89 (1H, m), 2.84 (1H, dd, *J* = 10.2, 4.8 Hz), 2.04-1.91 (3H, m), 1.88-1.81 (1H, m), 1.70-1.61 (1H, m), 1.27-1.18 (1H, m), 0.83 (9H, s), –0.04 (6H, s), (for minor diastereomer) *δ* 7.28 (2H, d, *J* = 7.8 Hz), 6.93 (2H, d, *J* = 7.8 Hz), 4.91 (1H, d, *J* = 4.2 Hz), 4.21 (1H, d, *J* = 4.8 Hz), 3.82 (3H, s), 2.94 (1H, dd, *J* = 10.8, 4.8 Hz), 2.86-2.80 (1H, m), 2.02 (2H, t, *J* = 6.3 Hz), 1.68-1.55 (2H, m), 1.48-1.42 (1H, m), 1.24-1.18 (1H, m), 0.94 (9H, s), 0.16 (6H, s); ^{13}C NMR (151 MHz, $CDCl_3$) (for major diastereomer) *δ* 160.0, 153.3, 129.8, 128.5, 114.6, 112.3, 111.9, 104.0, 55.5, 51.5, 37.1, 29.6, 27.8(3), 27.7(6), 25.8, 21.7, 18.2, –4.3, –4.5, (for minor diastereomer) *δ* 160.0, 155.2, 129.5, 128.6, 114.7, 112.6, 112.0, 103.1, 55.4, 51.4, 36.2, 30.0, 28.1, 25.8, 25.5, 19.5, 18.2, –4.0, –4.2; HRMS (ESI) Calcd for $C_{23}H_{32}O_2N_2NaSi^+$ ($[M+Na]^+$) 419.2125. Found 419.2139.

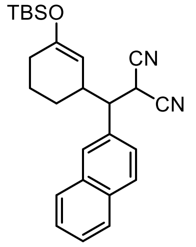

3d (dr = 1.8:1): ^{1}H NMR (600 MHz, $CDCl_3$) (for major diastereomer) *δ* 7.90 (1H, d, *J* = 8.4 Hz), 7.88-7.82 (2H, m), 7.80 (1H, s), 7.52 (1H, d, *J* = 7.2 Hz), 7.52 (1H, t, *J* = 5.4 Hz), 7.46 (1H, d, *J* = 7.8 Hz), 4.41 (1H, brs), 4.23 (1H, brd), 3.13-3.06 (2H, m), 2.05-1.95 (3H, m), 1.90-1.83 (1H, m), 1.74-1.65 (1H, m), 1.34-1.24 (1H, m), 0.79 (9H, s), –0.10 (3H, s), –0.11 (3H, s), (for minor diastereomer) *δ* 7.90 (1H, d, *J* = 8.4 Hz), 7.88-7.84 (2H, m), 7.82 (1H, s), 7.55-7.51 (2H, m), 7.49 (1H, d, *J* = 9.0 Hz), 5.00 (1H, d, *J* = 4.2 Hz), 4.32 (1H, d, *J* = 4.8 Hz), 3.17 (1H, dd, *J* = 10.2, 4.8 Hz), 3.04-2.98 (1H, m), 2.04 (2H, t, *J* = 6.3 Hz), 1.72-1.64 (1H, m), 1.63-1.56 (1H, m), 1.50-1.44 (1H, m), 1.27-1.19 (1H, m), 0.95 (9H, s), 0.18 (6H, s); ^{13}C NMR (151 MHz, $CDCl_3$) (for major isomer) *δ* 153.5, 134.0, 133.4(9), 133.4(5), 129.2, 128.4, 128.2, 127.9, 126.8, 126.7, 125.6, 112.1, 112.0, 103.9, 52.2, 37.3, 29.6, 27.8, 27.6, 25.7, 21.6, 18.1, –4.3, –4.5, (for minor diastereomer) *δ* 155.4, 134.1, 133.5(4), 133.4(6), 129.3, 128.3, 128.2, 127.9, 126.8, 126.7, 125.3, 112.4, 112.0, 103.0, 52.2, 36.2, 30.0, 27.9, 25.8, 25.7, 19.6, 18.2, –4.0, –4.2; HRMS (ESI) Calcd for $C_{26}H_{31}ON_2Si^-$ ($[M-H]^-$) 415.2200. Found 415.2210.

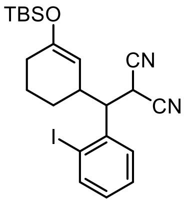

3e (dr = 1.6:1): ^{1}H NMR (400 MHz, $CDCl_3$) (for major diastereomer) δ 7.97-7.93 (1H, m), 7.49 (1H, d, *J* = 2.1 Hz), 7.46-7.42 (1H, m), 7.07 (1H, t, *J* = 2.0 Hz), 4.26 (1H, brs), 4.05 (1H, d, *J* = 1.2 Hz), 3.64 (1H, dd, *J* = 2.6, 1.2 Hz), 3.02-2.97 (1H, m), 2.09-1.96 (3H, m), 1.94-1.88 (1H, m), 1.72-1.60 (1H, m), 1.42-1.35 (1H, m), 0.83 (9H, m), –0.01 (3H, s), –0.05 (3H, s), (for minor diasteromer) δ 7.97-7.93 (1H, m), 7.54 (1H, d, *J* = 2.0 Hz), 7.46-7.42 (1H, m), 7.07 (1H, t, *J* = 1.9 Hz), 4.98 (1H, d, *J* = 1.2 Hz), 4.18 (1H, d, *J* = 1.4 Hz), 3.74 (1H, dd, *J* = 2.6, 1.3 Hz), 2.90-2.84 (1H, m), 2.09-1.96 (2H, m), 1.88-1.79 (1H, m), 1.72-1.60 (1H, m), 1.45-1.40 (1H, m), 1.21-1.15 (1H, m), 0.95 (9H, s), 0.19 (3H, s), 0.18 (3H, s); ^{13}C NMR (151 MHz, $CDCl_3$) (for major diastereomer) δ 171.5, 152.2, 141.1, 139.5, 129.7, 129.0, 104.4, 103.0, 67.0, 56.1, 47.0, 43.0, 39.9, 29.9, 25.8, 21.4, 18.1, –4.1(8), –4.2(3), (for minor diastereomer) δ 171.5, 152.6, 141.6, 139.7, 129.6, 129.1, 107.4, 102.3, 67.0, 56.0, 47.1, 42.9, 40.7, 30.2, 28.1, 21.8, 18.2, –4.0, –4.1; HRMS (ESI) Calcd for $C_{22}H_{29}ON_2INaSi^+$ ($[M+Na]^+$) 515.0986. Found 515.0983.

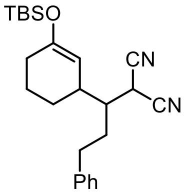

3f (dr = 1.6:1): ^{1}H NMR (400 MHz, $CDCl_3$) (for major diastereomer) δ 7.35-7.30 (2H, m), 7.26-7.20 (3H, m), 4.62 (1H, brs), 3.89 (1H, d, *J* = 2.4 Hz), 2.88-2.68 (2H, m), 2.68-2.60 (1H, m), 2.14-1.68 (5H, m), 1.64-1.49 (2H, m), 1.33-1.18 (2H, m), 0.92 (9H, s), 0.15 (6H, s), (for minor diastereomer) δ 7.35-7.28 (2H, m), 7.26-7.20 (3H, m), 4.62 (1H, brs), 3.79 (1H, d, *J* = 4.4 Hz), 2.88-2.68 (2H, m), 2.68-2.60 (1H, m), 2.14-1.68 (7H, m), 1.64-1.49 (2H, m), 0.92 (9H, s), 0.15 (6H, s); ^{13}C NMR (151 MHz, $CDCl_3$) (for major diastereomer) δ 155.1, 140.5, 128.9, 128.4, 126.6, 113.5, 112.6, 104.6, 45.2, 37.9, 33.7, 31.5, 30.0, 25.8, 25.2, 24.2, 22.2, 18.2, –4.1(1), –4.1(4), (for minor diastereomer) δ 154.5, 140.4, 128.9, 128.4, 126.7, 113.2, 112.4, 103.9, 44.1, 37.8, 33.9, 31.2, 29.9, 25.8, 25.1, 24.7, 21.7, 18.1, –4.1; HRMS (ESI) Calcd for $C_{24}H_{34}ON_2NaSi^+$ ($[M+Na]^+$) 417.2333. Found 417.2346.

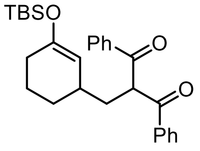

3g: ^{1}H NMR (600 MHz, $CDCl_3$) δ 7.97 (4H, d, *J* = 7.2 Hz), 7.56 (2H, t, *J* = 7.2 Hz), 7.48-7.43 (4H, m), 5.34 (1H, t, *J* = 6.6 Hz), 4.83 (1H, brs), 2.31 (1H, brs), 2.17-2.06 (2H, m), 2.04-1.92 (2H, m), 1.80-1.72 (2H, m), 1.58-1.49 (1H, m), 1.22-1.17 (1H, m), 0.91 (9H, s), 0.12 (6H, s); ^{13}C NMR (600 MHz, $CDCl_3$) δ 196.1, 196.0, 152.0, 136.2(2), 136.1(7), 133.6, 129.0, 128.8, 128.7, 108.0, 55.0, 36.3, 33.5, 30.1, 29.0, 25.8, 21.5, 18.2, –4.2, –4.3; HRMS_(ESI) Calcd for $C_{28}H_{36}O_3NaSi^+$ ($[M+Na]^+$) 471.2326. Found 471.2324.

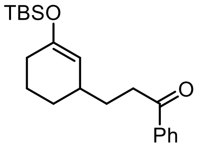

3h: The reaction was conducted with vinyl phenyl ketone (0.3 mmol, 3 equiv). ^{1}H NMR (600 MHz, $CDCl_3$) δ 7.96 (2H, d, *J* = 7.8 Hz), 7.56 (1H, t, *J* = 7.8 Hz), 7.46 (2H, t, *J* = 7.8 Hz), 4.82 (1H, brs), 2.99 (1H, t, *J* = 7.8 Hz), 2.28-2.22 (1H, m), 2.06-1.94 (2H, m), 1.82-1.67 (4H, m), 1.61-1.52 (1H, m), 1.20-1.13 (1H, m), 0.92 (9H, s), 0.13 (6H, s); ^{13}C NMR (151 MHz, $CDCl_3$) δ 200.8, 151.5, 137.2, 133.1, 128.7, 128.2, 108.8, 36.2, 34.5, 31.4, 30.1, 28.8, 25.9, 21.8, 18.2, –4.1, –4.3; HRMS (ESI) Calcd for $C_{21}H_{32}O_2NaSi^+$ ($[M+Na]^+$) 367.2064. Found 367.2063.

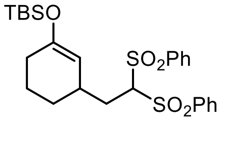

3i: ^{1}H NMR (600 MHz, $CDCl_3$) δ 7.97 (2H, d, *J* = 8.4 Hz), 7.95 (2H, d, *J* = 8.4 Hz), 7.72-7.70 (2H, m), 7.61-7.57 (4H, m), 4.64 (1H, brd), 4.46 (1H, t, *J* = 5.4 Hz), 2.46 (1H, br),

2.12-2.02 (2H, m), 1.96-1.91 (2H, m), 1.66-1.58 (2H, m), 1.52-1.46 (1H, m), 1.06-0.99 (1H, m), 0.91 (9H, s), 0.11 (6H, s); ^{13}C NMR (151 MHz, $CDCl_3$) *δ* 152.6, 138.1, 137.9, 134.7, 129.9, 129.8, 129.3, 106.6, 81.9, 32.8, 32.2, 29.9, 27.6, 25.8, 20.7, 18.2, –4.2; HRMS (ESI) Calcd for $C_{26}H_{36}O_5NaS_2Si^+$ ($[M+Na]^+$) 543.1666. Found 543.1667.

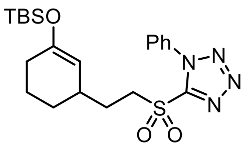

3j: The reaction was performed for 24 h. ^{1}H NMR (600 MHz, $CDCl_3$) *δ* 7.70 (2H, d, *J* = 7.8 Hz), 7.65-7.58 (3H, m), 4.72 (1H, brs), 3.80-3.69 (2H, m), 2.42-2.36 (1H, m), 2.09-1.86 (4H, m), 1.82-1.72 (2H, m), 1.63-1.56 (1H, m), 1.22-1.14 (1H, m), 0.92 (9H, s), 0.14 (6H, s); ^{13}C NMR (151 MHz, $CDCl_3$) *δ* 153.6, 152.9, 133.2, 131.6, 129.9, 125.2, 106.6, 54.2, 33.7, 29.9, 28.5, 28.1, 25.8, 21.5, 18.2, –4.1(6), –4.2(3); HRMS (ESI) Calcd for $C_{21}H_{32}O_3N_4NaSSi^+$ ($[M+Na]^+$) 471.1857. Found 471.1855.

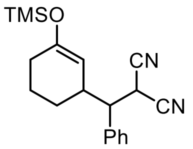

3k (dr = 2.0:1): ^{1}H NMR (600 MHz, $CDCl_3$) (for major diastereomer) *δ* 7.43-7.33 (5H, m), 4.36 (1H, brs), 4.15 (1H, d, *J* = 4.8 Hz), 3.01-2.95 (1H, m), 2.91-2.87 (1H, m), 2.02 (1H, t, *J* = 6.3 Hz), 2.01-1.94 (2H, m), 1.88-1.82 (1H, m), 1.71-1.62 (1H, m), 1.29-1.23 (1H, m), 0.02 (9H, s), (for minor diastereomer) *δ* 7.43-7.33 (5H, m), 4.95 (1H, d, *J* = 4.2 Hz), 4.24 (1H, d, *J* = 4.8 Hz), 3.01-2.95 (1H, m), 2.91-2.87 (1H, m), 2.01-1.94 (2H, m), 1.71-1.62 (1H, m), 1.62-1.56 (1H, m), 1.49-1.43 (1H, m), 1.22-1.17 (1H, m), 0.22 (9H, s); ^{13}C NMR (151 MHz, $CDCl_3$) (for major diastereomer) *δ* 153.2, 136.6, 129.3, 128.7, 112.1, 111.9, 103.9, 52.1, 37.1, 29.6, 27.6(4), 27.5(5), 21.6, 0.3, (for minor diastereomer) *δ* 155.0, 136.7, 129.0, 128.4, 112.4, 111.9, 103.1, 52.0, 36.1, 30.0, 27.8, 25.5, 19.6, 0.6; HRMS (ESI) Calcd for $C_{19}H_{23}ON_2Si^-$ ($[M-H]^-$) 323.1574. Found 323.1580.

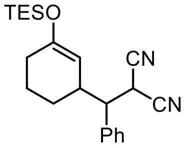

3l (dr = 2.0:1): ^{1}H NMR (600 MHz, $CDCl_3$) (for major diastereomer) *δ* 7.43-7.33 (5H, m), 4.34 (1H, brs), 4.15 (1H, d, *J* = 5.4 Hz), 3.01-2.95 (1H, m), 2.91-2.85 (1H, m), 2.05 (1H, t, *J* = 6.3 Hz), 2.02-1.95 (2H, m), 1.89-1.83 (1H, m), 1.71-1.63 (1H, m), 1.29-1.22 (1H, m), 0.83 (9H, t, *J* = 7.8 Hz), 0.47 (6H, q, *J* = 7.8 Hz), (for minor diastereomer) *δ* 7.43-7.33 (5H, m), 4.94 (1H, d, *J* = 4.2 Hz), 4.23 (1H, d, *J* = 4.8 Hz), 3.01-2.95 (1H, m), 2.91-2.85 (1H, m), 2.02-1.95 (2H, m), 1.71-1.63 (1H, m), 1.63-1.56 (1H, m), 1.49-1.43 (1H, m), 1.22-1.17 (1H, m), 1.00 (9H, t, *J* = 8.0 Hz), 0.69 (6H, q, *J* = 8.0 Hz): ^{13}C NMR (151 MHz, $CDCl_3$) (for major diastereomer) *δ* 153.3, 136.6, 129.3, 128.7, 112.1, 111.8, 103.4, 52.1, 37.1, 29.5, 27.7, 27.6, 21.6, 6.7, 5.0, (for minor diastereomer) *δ* 155.3, 136.7, 129.0, 128.4, 112.4, 111.9, 102.6, 52.0, 36.1, 29.9, 27.8, 25.5, 19.6, 6.9, 5.3; HRMS (ESI) Calcd for $C_{22}H_{29}ON_2Si^-$ ($[M-H]^-$) 365.2044. Found 365.2048.

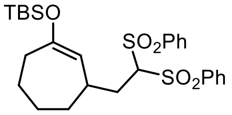

3m: ^{1}H NMR (600 MHz, $CDCl_3$) *δ* 7.96 (2H, d, *J* = 8.4 Hz), 7.93 (2H, d, *J* = 7.8 Hz), 7.72-7.68 (2H, m), 7.58 (4H, t, *J* = 7.8 Hz), 4.61 (1H, d, *J* = 4.2 Hz), 4.43 (1H, d, *J* = 5.4 Hz), 2.28-2.16 (3H, m), 2.09-2.03 (1H, m), 2.00 (1H, dd, *J* =15.9, 7.5 Hz), 1.84-1.77 (1H, m), 1.61-1.52 (1H, m), 1.52-1.46 (1H, m), 1.45-1.34 (1H, m), 1.28-1.21 (1H, m), 0.92 (9H, s), 0.12 (6H, s); ^{13}C NMR (151 MHz, $CDCl_3$) *δ* 157.7, 138.4, 137.9, 134.7(1), 134.6(7), 129.9, 129.6, 129.3, 129.2, 109.8, 82.0, 35.3,

34.7, 33.8, 32.2. 29.7, 25.9, 24.8, 18.2, –4.2, –4.3; HRMS (ESI) Calcd for $C_{27}H_{38}O_5NaS_2Si^+$ ([M+Na]$^+$) 557.1822. Found 557.1823.

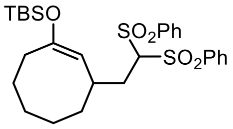

3n: ^{1}H NMR (600 MHz, $CDCl_3$) δ 7.96 (2H, d, J = 7.8 Hz), 7.91 (2H, d, J = 7.8 Hz), 7.70 (1H, t, J = 7.8 Hz), 7.69 (1H, t, J = 7.8 Hz), 7.58 (2H, t, J = 7.8 Hz), 7.57 (2H, t, J = 7.8 Hz), 4.55 (1H, dd, J = 8.7, 1.5 Hz), 4.13 (1H, d, J = 9.0 Hz), 2.42-2.34 (1H, m), 2.22-2.14 (2H, m), 2.06 (1H, ddd, J = 15.0, 8.7, 3.9 Hz), 1.87 (1H, dt, J = 13.8, 3.9 Hz), 1.72-1.59 (2H, m), 1.52-1.38 (4H, m), 1.24-1.10 (2H, m), 0.93 (9H, s), 0.17 (3H, s), 0.15 (3H, s); ^{13}C NMR (151 MHz, $CDCl_3$) δ 156.3, 138.5, 138.2, 134.6, 129.9, 129.6, 129.3, 129.2, 105.7, 82.3, 38.1, 35.3, 32.8, 32.0, 28.5, 27.0, 26.0, 25.8, 18.2, –4.0, –4.4; HRMS (ESI) Calcd for $C_{28}H_{40}O_5NaS_2Si^+$ ([M+Na]$^+$) 571.1979. Found 571.1976.

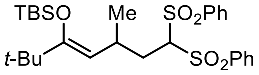

3o (Z/E = >20:1): ^{1}H NMR (600 MHz, $CDCl_3$) δ 7.87(9) (2H, d, J = 7.8 Hz), 7.87(6) (2H, d, J = 8.4 Hz), 7.64 (1H, t, J = 7.8 Hz), 7.61 (1H, t, J = 8.4 Hz), 7.50 (2H, t, J = 8.4 Hz), 7.48 (2H, t, J = 7.8 Hz), 4.59 (1H, d, J = 8.4 Hz), 4.22 (1H, d, J = 10.8 Hz), 3.23-3.14 (1H, m), 2.26 (1H, ddd, J = 15.0, 9.0, 4.2 Hz), 1.94-1.87 (1H, m), 1.06 (9H, s), 1.04 (9H, s), 0.33 (3H, s), 0.17 (3H, s); ^{13}C NMR (151 MHz, $CDCl_3$) δ 161.1, 139.6, 137.3, 134.5, 134.0, 130.3, 129.1, 129.0, 108.3, 81.6, 37.1, 33.0, 29.1, 26.9, 21.9, 19.5, –1.8, –4.1; HRMS (ESI) Calcd for $C_{28}H_{42}O_5NaS_2Si^+$ ([M+Na]$^+$) 573.2135. Found 573.2155.

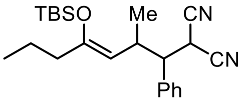

3p (dr = 1.6:1): ^{1}H NMR (600 MHz, $CDCl_3$) (for major isomer) δ 7.45-7.38 (5H, m), 4.37 (1H, d, J = 10.2 Hz), 4.34 (1H, d, J = 4.2 Hz), 2.99-2.91 (1H, m), 2.78 (1H, dd, J = 10.2, 4.2 Hz), 2.27 (1H, dt, J = 13.8, 8.4 Hz), 2.20 (1H, dt, J = 13.8, 7.2 Hz), 1.61-1.54 (2H, m), 0.97 (3H, t, J = 6.6 Hz), 0.95 (9H, s), 0.86 (3H, d, J = 6.6 Hz), 0.19 (3H, s), 0.17 (3H, s), (for minor isomer) δ 7.44-7.33 (5H, m), 4.30 (1H, d, J = 4.2 Hz), 4.27 (1H, d, J = 9.6 Hz), 3.33-3.25 (1H, m), 2.78 (1H, dd, J = 11.1, 4.2 Hz), 2.11 (1H, ddd, J = 15.0, 9.0, 5.7 Hz), 2.04 (1H, ddd, J = 15.0, 9.0, 6.8 Hz), 1.63-1.48 (2H, m), 1.01 (9H, s), 0.96 (3H, t, J = 7.2 Hz), 0.81 (3H, d, J = 6.8 Hz), 0.21 (6H, s); ^{13}C NMR (151 MHz, $CDCl_3$) (for major isomer) δ 155.7, 135.8, 129.3, 129.1, 128.8, 112.7, 112.1, 109.5, 53.6, 34.7, 34.1, 28.8, 25.8, 20.8, 20.5, 18.3, 14.0, –3.8, –4.4, (for minor isomer) δ 154.6, 136.1, 129.2, 128.9, 113.2, 112.0, 109.7, 53.7, 38.7, 32.2, 28.5, 26.0, 20.6, 19.5, 18.5, 13.9, –3.5, –3.6; HRMS (ESI) Calcd for $C_{23}H_{34}ON_2NaSi^+$ ([M+Na]$^+$) 405.2333. Found 405.2348.

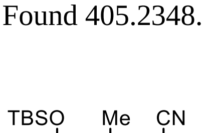

3q (dr = 1.5:1): ^{1}H NMR (400 MHz, $CDCl_3$) (for major diastereomer) δ 7.45-7.33 (5H, m), 4.32 (1H, d, J = 4.0 Hz), 4.24 (1H, d, J = 9.6 Hz), 3.34-3.22 (1H, m), 2.79 (1H, dd, J = 11.4, 4.0 Hz), 1.88 (3H, s), 1.01 (9H, s), 0.81 (3H, d, J = 7.2 Hz), 0.22 (6H, s), (for minor diastereomer) δ 7.40-7.31 (3H, m), 7.20-7.14 (2H, m), 4.14 (1H, d, J = 9.2 Hz), 4.00 (1H, d, J = 10.0 Hz), 3.39-3.29 (1H, m), 3.19 (1H, dd, J = 9.2, 5.6 Hz), 1.77 (3H, s), 0.98 (9H, s), 0.98 (3H, d, J = 7.2 Hz), 0.20 (3H, s), 0.18 (3H, s); ^{13}C NMR (151 MHz, $CDCl_3$) (for major diastereomer) δ 150.8, 136.2, 129.2, 128.9, 113.2, 112.1, 110.3, 53.6, 32.4, 28.6, 25.9, 23.1, 19.4, 18.5, –3.4, –3.5, (for minor diastereomer) δ 149.8, 135.2, 129.0, 128.6,

113.3, 112.5, 107.6, 52.8, 32.0, 27.3, 26.0, 23.1, 19.9, 18.5, –3.2, –3.6; HRMS (ESI) Calcd for $C_{21}H_{30}ON_2NaSi^+$ ($[M+Na]^+$) 377.2020. Found 377.2021.

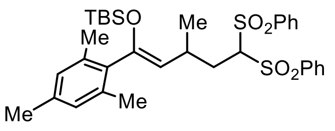

3r (*Z*/*E* = 5:1): ^{1}H NMR (600 MHz, $CDCl_3$) (for *Z* isomer) δ 8.04 (2H, d, *J* = 7.8 Hz), 7.78 (2H, d, *J* = 7.8 Hz), 7.70 (1H, t, *J* = 7.8 Hz), 7.57 (2H, t, *J* = 7.8 Hz), 7.49 (1H, t, *J* = 7.8 Hz), 7.30 (2H, t, *J* = 7.8 Hz), 6.82 (2H, s), 4.57 (1H, dd, *J* = 7.5, 2.1 Hz), 3.64 (1H, d, *J* = 9.6 Hz), 3.36-3.28 (1H, m), 2.34-2.28 (1H, m), 2.29 (3H, s), 2.24 (3H, s), 2.20 (3H, s), 1.94-1.88 (1H, m), 1.08 (3H, d, *J* = 6.6 Hz), 0.95 (9H, s), –0.13 (3H, s), –0.25 (3H, s): ^{13}C NMR (151 MHz, $CDCl_3$) (for *Z* isomer) δ 149.2, 139.2, 137.4(2), 137.4(0), 136.1, 135.7, 135.6, 134.4, 134.3, 129.8, 129.6, 129.0(2), 128.9(8), 128.5, 128.4, 115.7, 82.0, 32.5, 29.6, 25.8, 21.2, 21.0, 20.5, 18.4, –4.3; HRMS (ESI) Calcd for $C_{33}H_{44}O_5NaS_2Si^+$ ($[M+Na]^+$) 635.2292. Found 635.2305.

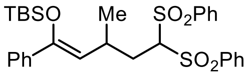

3s (*Z*/*E* = 6.7:1): ^{1}H NMR (600 MHz, $CDCl_3$) (for *Z* isomer) δ 8.12 (2H, d, *J* = 7.2 Hz), 7.71 (1H, d, *J* = 7.2 Hz), 7.60 (2H, t, *J* = 7.2 Hz), 7.57 (2H, d, *J* = 8.4 Hz), 7.40-7.26 (5H, m), 7.34 (1H, t, *J* = 7.2 Hz), 7.05 (2H, dd, *J* = 8.4, 7.2 Hz), 4.50 (1H, d, *J* = 8.4 Hz), 3.92 (1H, d, *J* = 10.2 Hz), 3.32-3.24 (1H, m), 2.46-2.42 (1H, m), 1.81-1.75 (1H, m), 1.06 (3H, d, *J* = 6.6 Hz), 0.98 (9H, s), 0.06 (3H, s), –0.11 (3H, s): ^{13}C NMR (151 MHz, $CDCl_3$) (for *Z* isomer) δ 152.1, 139.7, 139.6, 137.5, 134.5, 134.2, 129.8, 129.3, 129.1, 129.0, 128.3, 126.5, 114.7, 81.3, 33.0, 30.5, 26.1, 21.4, 18.6, –3.7, –3.9; HRMS (ESI) Calcd for $C_{30}H_{38}O_5NaS_2Si^+$ ($[M+Na]^+$) 593.1822. Found 593.1819.

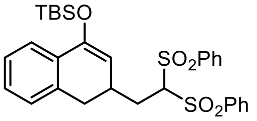

3t: ^{1}H NMR (600 MHz, $CDCl_3$) δ 7.92 (2H, d, *J* = 8.4 Hz), 7.80 (2H, d, *J* = 8.4 Hz), 7.69 (1H, t, *J* = 7.2 Hz), 7.64 (1H, t, *J* = 7.2 Hz), 7.55 (2H, dd, *J* = 8.4, 7.2 Hz), 7.48 (2H, dd, *J* = 8.4, 7.2 Hz), 7.33 (1H, d, *J* = 7.2 Hz), 7.17 (1H, t, *J* = 7.2 Hz), 7.14 (1H, t, *J* = 7.2 Hz), 7.00 (1H, d, *J* = 7.2 Hz), 4.97 (1H, d, *J* = 5.4 Hz), 4.40 (1H, t, *J* = 5.4 Hz), 2.92 (1H, dd, *J* = 15.0, 6.2 Hz), 2.90-2.83 (1H, m), 2.50 (1H, dd, 15.0, 5.4 Hz), 2.14-2.04 (2H, m), 1.00 (9H, s), 0.19 (3H, s), 0.16 (3H, s); ^{13}C NMR (151 MHz, $CDCl_3$) δ 149.5, 137.9, 137.8, 134.8, 134.6, 132.6, 129.8, 129.5, 129.3, 128.1, 126.6, 122.4, 105.6, 81.7, 33.6, 31.1, 29.7, 26.0, 18.4, –4.2, –4.4; HRMS (ESI) Calcd for $C_{30}H_{36}O_5NaS_2Si^+$ ($[M+Na]^+$) 591.1666. Found 591.1664.

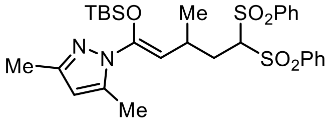

3u (*Z*/*E* = 1.7:1): ^{1}H NMR (600 MHz, $CDCl_3$) (for *Z* isomer) δ 8.03 (2H, d, *J* = 7.8 Hz), 7.83 (2H, d, *J* = 7.8 Hz), 7.69 (1H, t, *J* = 7.8 Hz), 7.56 (2H, t, *J* = 7.8 Hz), 7.53 (1H, t, *J* = 7.8 Hz), 7.36 (2H, t, *J* = 7.8 Hz), 5.83 (1H, s), 4.54 (1H, dd, *J* = 7.5, 2.4 Hz), 3.83 (1H, d, *J* = 11.0 Hz), 3.21-3.12 (1H, m), 2.35 (1H, ddd, *J* = 11.0, 7.5, 4.2 Hz), 2.24 (3H, s), 2.17 (3H, s), 1.93 (1H, ddd, *J* = 15.3, 11.0, 2.4 Hz), 1.09 (3H, d, *J* = 7.5 Hz), 0.94 (9H, s), –0.06 (6H, s), (for *E* isomer) δ 8.04 (2H, d, *J* = 7.8 Hz), 7.93 (2H, d, *J* = 7.8 Hz), 7.65 (1H, t, *J* = 7.8 Hz), 7.61 (1H, t, *J* = 7.8 Hz), 7.54 (2H, t, *J* = 7.8 Hz), 7.46 (2H, t, *J* = 7.8 Hz), 5.86 (1H, s), 5.58 (1H, dd, *J* = 6.6, 1.8 Hz), 4.61 (1H, d, *J* = 10.8 Hz), 2.21-2.16 (1H, m), 2.19 (3H, s), 2.18 (3H, s), 2.10-2.01 (2H, m), 0.95 (3H, d, *J* = 6.6 Hz), 0.89 (9H, s), 0.17 (3H, s), 0.00(4) (3H, s); ^{13}C NMR (151 MHz, $CDCl_3$) (for *Z* isomer) δ 148.4, 143.4, 139.4, 138.9, 137.4,

134.5, 134.3, 129.7(4), 129.6(5), 129.1(0), 129.0(8), 109.0, 106.3, 81.5, 32.4, 30.2, 25.7, 21.2, 18.3, 13.6, 11.9, –5.0, –5.3, (for *E* isomer) δ 148.9, 143.2, 140.0, 138.5, 137.9, 134.2(0), 134.1(8), 129.9, 129.7, 128.8(9), 128.8(6), 111.0, 106.0, 80.0, 31.7, 31.6, 25.6, 22.4, 18.1, 13.7, 11.1, –5.0, –5.1; HRMS (ESI): Calcd for $C_{29}H_{40}O_5N_2NaS_2Si^+$ ($[M+Na]^+$) 611.2040. Found 611.2035.

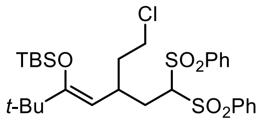

3v (*Z*/*E* = >20:1): ^{1}H NMR (600 MHz, $CDCl_3$) δ 7.89 (2H, d, *J* = 7.8 Hz), 7.87 (2H, d, *J* = 7.8 Hz), 7.66 (1H, t, *J* = 7.8 Hz), 7.64 (1H, t, *J* = 7.8 Hz), 7.52 (2H, t, *J* = 7.8 Hz), 7.50 (2H, t, *J* = 7.8 Hz), 4.57 (1H, d, *J* = 8.4 Hz), 4.21 (1H, d, *J* = 10.2 Hz), 3.60-3.49 (2H, m), 3.26-3.17 (1H, m), 2.36-2.30 (1H, m), 1.99-1.93 (1H, m), 1.93-1.82 (2H, m), 1.08 (9H, s), 1.04 (9H, s), 0.34 (3H, s), 0.22 (3H, s); ^{13}C NMR (151 MHz, $CDCl_3$) δ 162.4, 138.8, 137.3, 134.7, 134.3, 130.1, 129.3, 129.2, 129.1, 105.6, 81.1, 41.9, 39.5, 37.3, 33.0, 30.4, 29.1, 26.9, 19.6, –1.8, –3.5; HRMS (ESI) Calcd for $C_{29}H_{43}O_5ClNaS_2Si^+$ ($[M+Na]^+$) 621.1902. Found 621.1909.

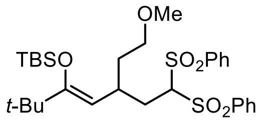

3w (*Z*/*E* = >20:1): ^{1}H NMR (600 MHz, $CDCl_3$) δ 7.90 (2H, d, *J* = 8.4 Hz), 7.87 (2H, d, *J* = 7.8 Hz), 7.65 (1H, t, *J* = 7.2 Hz), 7.62 (1H, t, *J* = 7.2 Hz), 7.51 (2H, dd, *J* = 7.8, 7.2 Hz), 7.49 (2H, dd, *J* = 8.4, 7.2 Hz), 4.61 (1H, d, *J* = 6.6 Hz), 4.24 (1H, d, *J* = 11.4 Hz), 3.49-3.40 (2H, m), 3.32 (3H, s), 3.22-3.15 (1H, m), 2.36 (1H, ddd, *J* = 15.9, 8.7, 4.2 Hz), 1.97 (1H, dd, *J* = 13.8, 11.4 Hz), 1.71-1.61 (2H, m), 1.07 (9H, s), 1.04 (9H, s), 0.35 (3H, s), 0.20 (3H, s); ^{13}C NMR (151 MHz, $CDCl_3$) δ 161.4, 139.2, 137.4, 134.5, 134.1, 130.2, 129.3, 129.1, 129.0, 106.6, 81.3, 70.5, 58.8, 37.3, 35.9, 32.1, 30.7, 29.2, 26.9, 19.6, –1.9, –3.8; HRMS (ESI) Calcd for $C_{30}H_{46}O_6NaS_2Si^+$ ($[M+Na]^+$) 617.2397. Found 617.2408.

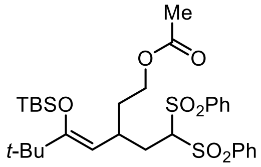

3x (*Z*/*E* = >20:1): ^{1}H NMR (600 MHz, $CDCl_3$) δ 7.89 (2H, d, *J* = 8.4 Hz), 7.87 (2H, d, *J* = 8.4 Hz), 7.65 (1H, t, *J* = 7.8 Hz), 7.63 (1H, t, *J* = 7.8 Hz), 7.50 (2H, dd, *J* = 8.4, 7.8 Hz), 7.49 (2H, dd, *J* = 8.4, 7.8 Hz), 4.61 (1H, d, *J* = 8.4 Hz), 4.24 (1H, d, *J* = 10.2 Hz), 4.18-4.08 (2H, m), 3.30-3.21 (1H, m), 2.40-2.33 (1H, m), 2.04 (3H, s), 1.92 (1H, t, *J* = 13.2 Hz), 1.81-1.66 (2H, m), 1.08 (9H, s), 1.04 (9H, s), 0.37 (3H, s), 0.21 (3H, s); ^{13}C NMR (151 MHz, $CDCl_3$) δ 171.2, 162.0, 139.1, 137.3, 134.6, 134.2, 130.2, 129.2, 129.1, 129.0, 106.2, 81.1, 62.1, 37.3, 34.8, 31.8, 30.4, 29.1, 26.9, 21.2, 19.6, –1.8, –3.8; HRMS (ESI) Calcd for $C_{31}H_{46}O_7NaS_2Si^+$ ($[M+Na^+]$) 645.2346. Found 645.2352.

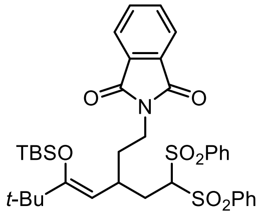

3y (*Z*/*E* = >20:1): ^{1}H NMR (600 MHz, $CDCl_3$) δ 7.94 (2H, d, *J* = 7.8 Hz), 7.91 (2H, d, *J* = 7.8 Hz), 7.87-7.82 (2H, m), 7.74-7.69 (2H, m), 7.67 (1H, t, *J* = 7.8 Hz), 7.64 (1H, t, *J* = 7.8 Hz), 7.54 (2H, t, *J* = 7.8 Hz), 7.51 (2H, t, *J* = 7.8 Hz), 4.62 (1H, d, *J* = 8.4 Hz), 4.24 (1H, d, *J* = 10.8 Hz), 3.80-3.67 (2H, m), 3.30-3.22 (1H, m), 2.42 (1H, ddd, *J* = 14.7, 8.4, 3.6 Hz), 2.06-1.98 (1H, m), 1.82-1.78 (1H, m), 1.77-1.69 (1H, m), 1.06 (9H, s), 1.05 (9H, s), 0.41 (3H, s), 0.25 (3H, s); ^{13}C NMR (151 MHz, $CDCl_3$) δ 168.2, 162.2, 139.0, 137.3, 134.6, 134.2, 134.1, 132.4, 130.2, 129.4, 129.2, 129.1, 123.3, 105.7, 81.2, 37.3, 35.6, 34.6, 32.5,

30.4, 29.1, 26.9, 19.6, –1.8, –3.6; HRMS (ESI) Calcd for $C_{37}H_{47}O_7NNaS_2Si^+$ ($[M+Na]^+$) 732.2455. Found 732.2424.

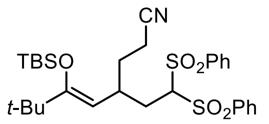

3z (*Z*/*E* = >20:1): ^{1}H NMR (600 MHz, $CDCl_3$) δ 7.88 (2H, d, J = 8.4 Hz), 7.85 (2H, d, J = 7.8 Hz), 7.67 (1H, t, J = 8.4 Hz), 7.65 (1H, t, J = 7.8 Hz), 7.55-7.48 (4H, m), 4.51 (1H, d, J = 8.4 Hz), 4.18 (1H, d, J = 10.2 Hz), 3.21-3.12 (1H, m), 2.41-2.31 (2H, m), 2.30-2.24 (1H, m), 1.96 (1H, t, J = 12.6 Hz), 1.88-1.80 (1H, m), 1.74-1.65 (1H, m), 1.08 (9H, s), 1.03 (9H, s), 0.31 (3H, s), 0.24 (3H, s); ^{13}C NMR (151 MHz, $CDCl_3$) δ 163.3, 138.6, 137.2, 134.8, 134.4, 130.0, 129.3, 129.2, 129.1, 119.9, 104.6, 81.0, 37.4, 33.8, 31.9, 30.3, 29.1, 26.8, 19.5, 14.4, –1.8, –3.3; HRMS (ESI) Calcd for $C_{30}H_{43}O_5NNaS_2Si^+$ ($[M+Na]^+$) 612.2244. Found 612.2258.

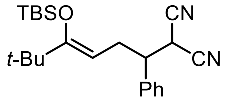

3Aa (*Z*/*E* = >20:1): ^{1}H NMR (600 MHz, $CDCl_3$) δ 7.42-7.34 (3H, m), 7.33-7.30 (2H, m), 4.33 (1H, t, J = 6.6 Hz), 3.98 (1H, d, J = 5.4 Hz), 3.21 (1H, td, J = 7.8, 5.4 Hz), 2.78 (1H, ddd, J = 14.8, 7.8, 6.6 Hz), 2.60 (1H, ddd, J = 14.8, 7.8, 6.6 Hz), 1.01 (9H, s), 1.00 (9H, s), 0.24 (3H, s), 0.21 (3H, s); ^{13}C NMR (151 MHz, $CDCl_3$) δ 162.0, 137.1, 129.2, 128.9, 128.1, 112.3, 111.9, 98.5, 47.2, 37.0, 29.2, 29.0, 28.9, 26.6, 19.3, –2.3, –2.6; HRMS (ESI): Calcd for $C_{23}H_{33}ON_2Si^-$ ($[M-H]^-$) 381.2357. Found 381. 2356.

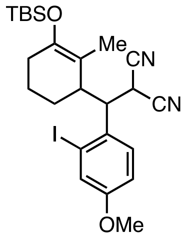

3Ab (dr = 2.0:1): ^{1}H NMR (600 MHz, $CDCl_3$) (for major diastereomer) δ 7.49 (1H, d, J = 8.4 Hz), 7.44 (1H, d, J = 3.0 Hz), 6.97 (1H, dd, J = 8.4, 3.0 Hz), 4.03 (1H, d, J = 6.0 Hz), 3.98 (1H, dd, J = 8.1, 6.0 Hz), 3.81 (3H, s), 2.78-2.72 (1H, m), 2.18-2.04 (2H, m), 1.79-1.64 (4H, m), 1.31 (3H, s), 0.92 (9H, s), 0.13 (3H, s), 0.11 (3H, s); ^{13}C NMR (151 MHz, $CDCl_3$) (for major diastereomer) δ 159.7, 147.9, 132.0, 128.2, 125.6, 114.9, 112.6, 111.9, 111.1, 103.3, 55.7, 50.5, 42.6, 30.0, 26.2, 25.9, 25.4, 19.9, 18.3, 16.6, –3.2, –3.3; HRMS (ESI) Calcd for $C_{24}H_{33}O_2N_2INaSi^+$ ($[M+Na]^+$) 559.1248. Found 559.1255.

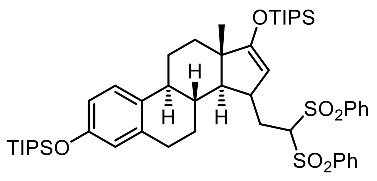

3Ac (dr = 9.2:1): ^{1}H NMR (600 MHz, $CDCl_3$) (for major isomer) δ 8.06 (2H, d, J = 7.8 Hz), 7.88 (2H, d, J = 7.8 Hz), 7.73 (1H, t, J = 7.8 Hz), 7.66 (1H, t, J = 7.2 Hz), 7.61 (2H, t, J = 7.8 Hz), 7.54 (2H, t, J = 7.8 Hz), 7.05 (1H, d, J = 9.0 Hz), 6.65 (1H, dd, J = 8.1, 2.4 Hz), 6.61 (1H, s), 4.50 (1H, d, J = 3.0 Hz), 4.47 (1H, dd, J = 10.2, 2.4 Hz), 3.00-2.94 (1H, m), 2.80-2.78 (2H, m), 2.32-2.21 (3H, m), 2.15 (1H, ddd, J = 14.7, 10.2, 4.8 Hz), 1.85 (1H, dd, J = 11.7, 7.2 Hz), 1.82-1.74 (2H, m), 1.60-1.37 (4H, m), 1.30-1.18 (6H, m), 1.12 (18H, br), 1.10 (18H, br), 0.91 (3H, s); ^{13}C NMR (151 MHz, $CDCl_3$) (for major isomer) δ 167.7, 154.0, 138.2, 138.1, 137.8, 134.7, 134.6, 133.2, 129.9, 129.7, 129.2(2), 129.1(8), 125.4, 119.8, 116.9, 98.6, 83.5, 55.1, 45.4, 45.3, 38.6, 35.9, 35.1, 29.2, 27.5, 26.2, 25.6, 21.5, 18.1, 12.9, 12.7; HRMS (ESI) Calcd for $C_{50}H_{73}O_6S_2Si_2^-$ ($[M-H]^-$) 889.4382. Found 889.4387.

Derivatization of Alkylated Enol Silyl Ethers

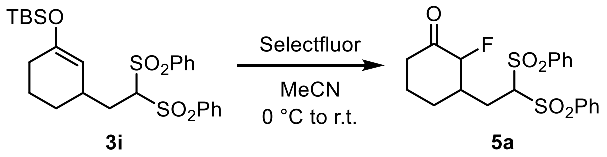

To a solution of **3i** (30.7 mg, 0.059 mmol) in MeCN (0.6 mL, 0.1 M) was added Selectfluor (23.0 mg, 0.065 mmol) at 0 °C, and the resulting reaction mixture was allowed to warm to room temperature. After stirring for 3 h, the solvent was removed under reduced pressure. The crude residue was purified by column chromatography on silica gel (hexane/EtOAc = 1:1 as eluent) to afford **5a** in 84% yield (21.1 mg, 0.050 mmol, dr = 1.4:1). ^{1}H NMR (400 MHz, $CDCl_3$) (for major diastereomer) δ 7.93 (2H, dd, J = 8.4, 1.2 Hz), 7.89 (2H, dd, J = 8.8, 1.2 Hz), 7.70 (1H, t, J = 7.6 Hz), 7.68 (1H, t, J = 8.0 Hz), 7.62-7.52 (4H, m), 4.82-4.77 (1H, m), 4.57 (1H, dd, $J_{H–F}$ = 49.8 Hz, $J_{H–H}$ = 11.0 Hz), 2.57-2.48 (2H, m), 2.46-2.24 (3H, m), 2.10-1.98 (2H, m), 1.69-1.44 (2H, m), (for minor diastereomer) δ 7.97 (2H, dd, J = 8.4, 1.0 Hz), 7.92 (2H, dd, J = 8.8, 1.6 Hz), 7.76-7.70 (2H, m), 7.64-7.57 (4H, m), 4.70 (1H, dd, $J_{H–F}$ = 50.6 Hz, $J_{H–H}$ = 3.8 Hz), 4.45 (1H, t, J = 5.8 Hz), 2.76-2.63 (1H, m), 2,63-2,53 (1H, m), 2.36-2.16 (3H, m), 1.94-1.68 (4H, m); ^{13}C NMR (151 MHz, $CDCl_3$) (for major diastereomer) δ 203.2 (d, $J_{C–F}$ = 14.3 Hz), 138.1, 137.1, 134.9, 134.8, 129.8, 129.6, 129.4, 129.3, 97.6 (d, $J_{C–F}$ = 192.4 Hz), 80.9 (d, $J_{C–F}$ = 4.2 Hz), 43.4 (d, $J_{C–F}$ = 16.0 Hz), 39.8, 29.9, 29.8 (d, $J_{C–F}$ = 8.8 Hz), 24.7, (for minor isomer) δ 205.0 (d, $J_{C–F}$ = 18.7 Hz), 137.8, 137.1, 135.0(4), 134.9(8), 129.8, 129.4(4), 129.4(0), 94.1 (d, $J_{C–F}$ = 188.0 Hz), 81.3, 41.6 (d, $J_{C–F}$ = 18.7 Hz), 38.8, 25.9 (d, $J_{C–F}$ = 5.9 Hz), 25.3, 23.5; HRMS (ESI) Calcd for $C_{20}H_{20}O_5FNaS_2^+$ ($[M+Na]^+$) 447.0707. Found 447.0703.

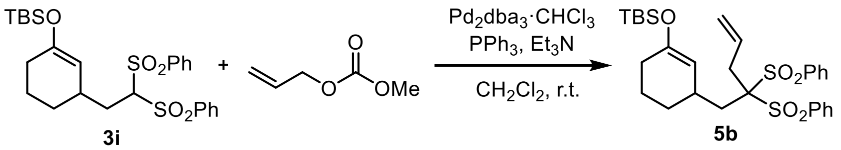

To a flame-dried Schlenk tube were added **3i** (72.9 mg, 0.14 mmol, 1 equiv), $Pd_2dba_3·CHCl_3$ (4.35 mg, 0.0042 mmol, [Pd] 6 mol%) and PPh_3 (5.51 mg, 0.021 mmol, 15 mol%) and the tube was degassed by alternating vacuum evacuation/Ar backfill. Then, CH_2Cl_2 (0.7 mL, 0.2 M) was introduced via syringe and the mixture was stirred for 10 min. After the addition of a solution of allyl methyl carbonate (19.1 μL, 0.17 mmol, 1.2 equiv) and Et_3N (21.5 μL, 0.15 mmol, 1.1 equiv) in CH_2Cl_2 (0.7 mL, 0.2 M), the whole reaction mixture was stirred at room temperature for 24 h and then diluted with CH_2Cl_2 and brine. The extractive work-up was carried out with CH_2Cl_2 and the combined organic layers were dried over Na_2SO_4, filtered, and concentrated. The crude product was purified by column chromatography on silica gel (hexane/EtOAc = 5:1 as eluent) to afford **5b** in 86% yield (67.3 mg, 0.12 mmol, 86%): ^{1}H NMR (600 MHz, $CDCl_3$) δ 8.06-8.02 (4H, m), 7.70 (2H, t, J = 7.8 Hz), 7.57 (4H, t, J = 7.8 Hz), 6.05-5.97 (1H, m), 5.22 (1H, br), 5.20-5.18 (1H, brm), 4.78 (1H, br), 3.21-3.12 (2H, m), 2.91-2.85 (1H, m), 2.26 (1H, dd, J = 15.6, 5.1 Hz), 2.18 (1H, dd, J = 15.6, 5.1 Hz), 2.00-1.88 (2H, m), 1.82-1.75 (1H, m), 1.69-1.62 (1H, m), 1.60-1.51 (1H, m), 1.27-1.19 (1H, m), 0.90 (9H, s), 0.09 (6H, s); ^{13}C NMR (151 MHz, $CDCl_3$) δ 151.6, 137.6, 137.5, 134.6, 131.6, 131.1, 128.6(8), 128.6(5), 120.0, 109.7, 92.5, 37.3, 34.4,

31.0, 29.9, 29.6, 25.8, 21.2, 18.2, –4.2, –4.3; HRMS (ESI) Calcd for $C_{29}H_{40}O_5NaS_2Si^+$ ([M+Na]$^+$) 583.1979. Found 583.1974.

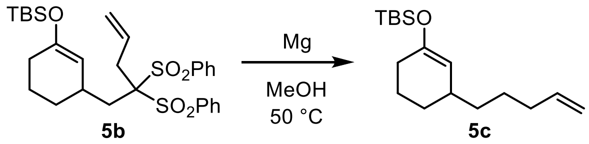

To a flame-dried test tube were added **5b** (26.4 mg, 0.047 mmol, 1 equiv) and anhydrous MeOH (1.2 mL, 0.04 M), and the reaction mixture was heated to 50 °C. To this solution was added Mg (22.9 mg, 0.94 mmol, 20 equiv) in three portions over 3 h at the same temperature. After stirring for additional 4 h, the reaction mixture was cooled to room temperature, filtered through a pad of Celite, and evaporated to remove the solvent. The crude residue was diluted with CH_2Cl_2 and brine, and then extractive work-up was carried out with CH_2Cl_2. The combined organic layers were dried over Na_2SO_4, filtered, and concentrated. The crude product was purified by silica gel column chromatography (hexane/EtOAc = 5:1 as eluent) to afford **5c** in 60% yield (7.9 mg, 0.028 mmol). ^{1}H NMR (400 MHz, $CDCl_3$) δ 5.88-5.76 (1H, m), 5.04-4.91 (2H, m), 4.79 (1H, brs), 2.16-1.89 (5H, m), 1.80-1.64 (2H, m), 1.61-1.48 (1H, m), 1.41 (2H, quin, J = 7.6 Hz), 1.34-1.20 (2H, m) 1.14-1.03 (1H, m), 0.92 (9H, s), 0.12 (6H, s); ^{13}C NMR (151 MHz, $CDCl_3$) δ 150.7, 139.3, 114.4, 109.8, 36.7, 34.7, 34.2, 30.2, 29.0, 26.5, 25.9, 21.9, 18.2, –4.2, –4.3.

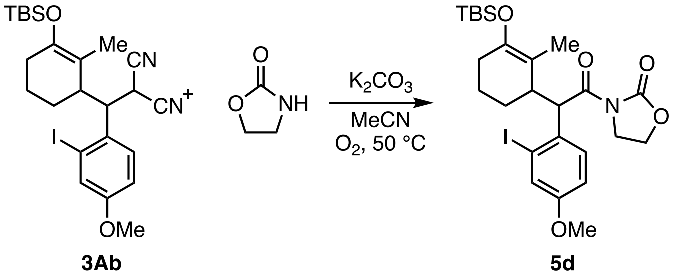

To a round-bottom flask were added **3Ab** (107.3 mg, 0.20 mmol, 1 equiv), K_2CO_3 (110.6 mg, 0.80 mmol, 4 equiv), and MeCN (2.0 mL, 0.1 M). The flask was then degassed and backfilled with O_2. To this mixture was added 2-oxazolidone (69.7 mg, 0.80 mmol, 4 equiv). The reaction mixture was heated to 50 °C and stirred there for 24 h under O_2 atmosphere (balloon), and then diluted with CH_2Cl_2 and filtered through a pad of Celite. The filtrates were concentrated and the crude material was purified by column chromatography on silica gel (hexane/EtOAc = 5:1) to afford **5d** in 70% yield (82.5 mg, 0.14 mmol). ^{1}H NMR (600 MHz, $CDCl_3$) δ 7.41 (1H, d, J = 3.0 Hz), 7.21 (1H, d, J = 8.7 Hz), 6.81 (1H, dd, J = 8.7, 3.0 Hz), 5.53 (1H, d, J = 6.6 Hz), 4.38-4.33 (1H, m), 4.31-4.26 (1H, m), 4.05-3.99 (1H, m), 3.97-3.92 (1H, m), 3.76 (3H, s), 2.92-2.85 (1H, m), 2.16-2.09 (1H, m), 2.03-1.96 (1H, m), 1.92-1.85 (1H, m), 1.81-1.75 (1H, m), 1.54-1.47 (1H, m), 1.39-1.32 (1H, m), 1.30 (3H, s), 0.92 (9H, s), 0.12 (3H, s), 0.10 (3H, s); ^{13}C NMR (151 MHz, $CDCl_3$) δ 173.0, 158.8, 152.7, 146.8, 131.9, 129.5, 125.5, 114.1, 111.9, 102.5, 61.7, 55.6, 53.7, 43.3, 43.2, 30.6, 26.6, 26.0, 21.2, 18.3, 15.6, –3.3, –3.4; HRMS (ESI) Calcd for $C_{25}H_{36}O_5NINaSi^+$ ([M+Na]$^+$) 608.1300. Found 608.1294.

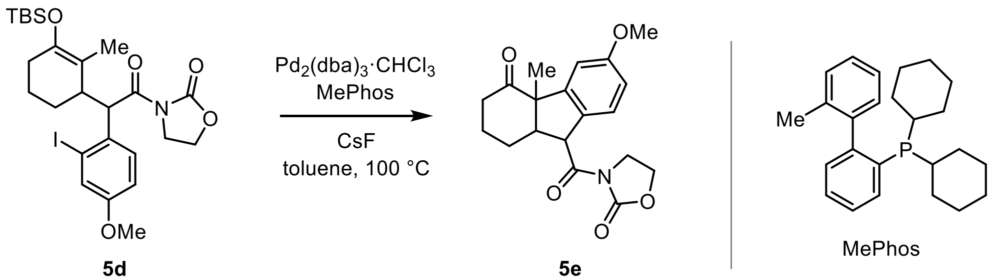

5d **5e** MePhos

To a flame-dried Schlenk were added **5d** (82.0 mg, 0.14 mmol, 1 equiv), $Pd_2dba_3{\cdot}CHCl_3$ (7.25 mg, 0.007 mmol, [Pd] 10 mol%), MePhos (6.12 mg, 0.017 mmol, 12 mol%), and CsF (33.0 mg, 0.21 mmol, 1.5 equiv). The Schlenk was then elaborately degassed and backfilled with Ar. Toluene (2.8 mL, 0.05 M) was added via syringe, and the reaction mixture was heated to 100 °C. After stirring overnight, the reaction mixture was cooled to room temperature and diluted with CH_2Cl_2. The mixture was filtered through a short pad of silica gel with the aid of EtOAc, and the filtrates were concentrated. The crude material was purified by column chromatography on silica gel (hexane/EtOAc = 3:1 to 1:3 as eluent) to afford **5e** in 56% yield (27.0 mg, 0.078 mmol, dr = 4.2:1). ^{1}H NMR (400 MHz, $CDCl_3$) (for major diastereomer) δ 7.02 (1H, d, J = 8.7 Hz), 6.79 (1H, dd, J = 8.7, 2.4 Hz), 6.75 (1H, d, J = 2.4 Hz), 5.14 (1H, d, J = 6.0 Hz), 4.48 (2H, t, J = 8.0 Hz), 4.10 (2H, t, J = 8.0 Hz), 3.79 (3H, s), 2.97 (1H, q, J = 6.0 Hz), 2.46 (1H, ddd, J = 16.0, 9.0, 6.0 Hz), 2.31 (1H, dt, J = 16.0, 6.0 Hz), 2.18-2.11 (1H, m), 1.99-1.91 (1H, m), 1.89-1.81 (1H, m), 1.75-1.68 (1H, m), 1.50 (3H, s), (for minor diastereomer) δ 7.21 (1H, d, J = 8.4 Hz), 6.82 (1H, dd, J = 8.4, 1.8 Hz), 6.53 (1H, d, J = 1.8 Hz), 5.36 (1H, d, J = 6.0 Hz), 4.54-4.45 (2H, m), 4.23-4.17 (1H, m), 4.10-4.05 (1H, m), 3.76 (3H, s), 3.15 (1H, dt, J = 11.4, 6.0 Hz), 2.45-2.40 (1H, m), 2.23 (1H, ddd, J = 15.6, 13.8, 5.4 Hz), 1.92-1.86 (1H, m), 1.68-1.59 (1H, m), 1.53-1.42 (2H, m), 1.39 (3H, s); ^{13}C NMR (151 MHz, $CDCl_3$) (for major diastereomer) δ 212.7, 174.4, 160.0, 153.7, 147.6, 131.7, 125.6, 114.5, 110.1, 62.2, 59.9, 55.6, 54.0, 52.2, 43.3, 38.7, 27.1, 25.2, 22.0, (for minor diastereomer) δ 211.2, 172.1, 160.0, 153.4, 147.8, 130.1, 127.9, 114.0, 108.0, 62.3, 59.6, 55.6, 52.6, 52.1, 42.9, 39.5, 25.4, 23.5, 23.2; HRMS (ESI) Calcd for Calcd for $C_{19}H_{21}O_5NNa^+$ ([M+Na]$^+$) 366.1312. Found 366.1317.

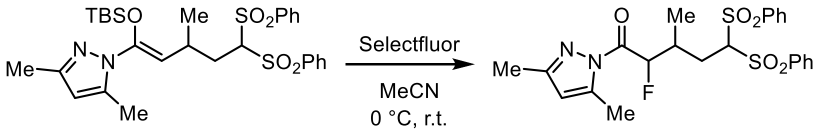

3u **S9**

To a solution of **3u** (58.9 mg, 0.10 mmol, 1 equiv) in MeCN (1.0 mL, 0.1 M) was added Selectfluor (35.4 mg, 0.10 mmol, 1 equiv) at 0 °C, and the reaction mixture was allowed to warm to room temperature. After stirring for 24 h, the solvent was removed under reduced pressure. The crude product was purified by column chromatography on silica gel (hexane/EtOAc = 3:1 as eluent) to afford **S9** in 68% yield (33.3 mg, 0.068 mmol, dr = 2.5:1). ^{1}H NMR (400 MHz, $CDCl_3$) (for major diastereomer) δ 7.93 (2H, d, J = 7.6 Hz), 7.88 (2H, d, J = 7.2 Hz), 7.76-7.64 (2H, m), 7.64-7.51 (4H, m), 6.01 (1H, s), 5.92 (1H, dd, $J_{H\text{–}F}$ = 50.0 Hz, $J_{H\text{–}H}$ = 3.2 Hz), 4.57 (1H, dd, J = 7.6, 4.4 Hz), 2.86-2.65 (1H, m), 2.56 (3H, s), 2.37 (1H, ddd, J = 16.0, 7.2, 5.3 Hz), 2.23 (3H, s), 2.10 (1H, ddd, J = 16.0, 8.9, 4.5 Hz), 1.03 (3H, d, J = 7.2 Hz), (for minor diastereomer) δ 8.05 (2H, d, J = 7.6 Hz), 8.01 (2H, d, J = 7.2 Hz), 7.76-7.64 (2H, m), 7.64-7.51 (4H, m), 6.01 (1H, s), 5.75 (1H, brd, $J_{H\text{–}F}$ = 50.4

Hz), 4.97 (1H, dd, J = 6.0, 4.4 Hz), 2.67-2.58 (1H, m), 2.55 (3H, s), 2.42 (1H, ddd, J = 15.2, 8.4, 4.0 Hz), 2.28-2.16 (1H, m), 2.24 (3H, s), 0.94 (3H, d, J = 7.6 Hz); ^{13}C NMR (151 MHz, $CDCl_3$) (for major diastereomer) δ 168.7 (d, $J_{C–F}$ = 24.5 Hz), 153.5, 144.9, 137.8, 137.6, 134.8, 134.7, 129.8, 129.7, 129.3, 129.2, 111.9, 91.9 (d, J_{C-F} = 182.3 Hz), 81.5, 34.7 (d, $J_{C–F}$ = 18.7 Hz), 27.2 (d, $J_{C–F}$ = 5.9 Hz), 16.7, 14.3, 14.0, (for minor diastereomer) δ 167.9 (d, $J_{C–F}$ = 23.1 Hz), 153.6, 145.0, 137.9, 137.3, 135.0, 134.9, 130.0, 129.9, 129.4, 129.3, 111.7, 89.1 (d, $J_{C–F}$ = 185.0 Hz), 81.4, 35.3 (d, $J_{C–F}$ = 20.2 Hz), 29.6, 14.2, 14.1, 12.9 (d, $J_{C–F}$ = 8.8 Hz); HRMS (ESI) Calcd for $C_{23}H_{25}O_5N_2FNaS_2^+$ ([M+Na]$^+$) 515.1081. Found 515.1087.

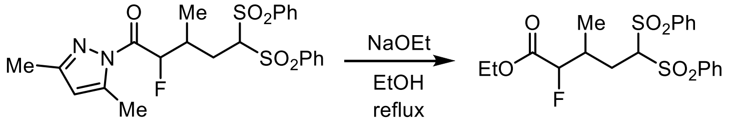

S9 **5f**

To a flame-dried test tube were added **S9** (49.3 mg, 0.10 mmol, 1 equiv) and anhydrous EtOH (1.0 mL, 0.1 M), and the test tube was degassed and backfilled with Ar. NaOEt (15.2 mg, 0.22 mmol, 2.2 equiv) was added at 0 °C, and the reaction mixture was refluxed with stirring overnight. After cooling to room temperature, the reaction was quenched with aqueous solution of 1 N HCl. The extractive work-up was carried out with EtOAc and the combined organic layers were dried over Na_2SO_4, filtered, and concentrated. The crude residue was purified by column chromatography on silica gel (hexane/EtOAc = 3:1) to afford **5g** in 90% yield (39.8 mg, 0.090 mmol). ^{1}H NMR (400 MHz, $CDCl_3$) (for major diastereomer) δ 7.99-7.91 (4H, m), 7.75-7.67 (2H, m), 7.63-7.56 (4H, m), 4.72 (1H, dd, $J_{H–F}$ = 48.8 Hz, $J_{H–H}$ = 4.4 Hz), 4.55 (1H, dd, J = 7.4, 4.6 Hz), 4.27 (2H, q, J = 7.2 Hz), 2.69-2.50 (1H, m), 2.38-2.27 (1H, m), 2.11 (1H, ddd, J = 15.8, 8.8, 4.8 Hz), 1.30 (3H, t, J = 7.2 Hz), 0.98 (3H, d, J = 6.8 Hz), (for minor diastereomer) δ 7.99-7.91 (4H, m), 7.75-7.67 (2H, m), 7.63-7.56 (4H, m), 4.80 (1H, dd, $J_{H–F}$ = 49.2 Hz, $J_{H–H}$ = 2.8 Hz), 4.49 (1H, t, J = 6.0 Hz), 4.26 (2H, q, J = 7.2 Hz), 2.69-2.50 (1H, m), 2.38-2.27 (1H, m), 2.24-2.16 (1H, m), 1.30 (3H, t, J = 7.2 Hz), 0.89 (3H, d, J = 6.8 Hz); ^{13}C NMR (151 MHz, $CDCl_3$) (for major diastereomer) δ 168.7 (d, $J_{C–F}$ = 24.6 Hz), 137.7, 137.6, 134.9, 129.9, 129.8, 129.3(7), 129.3(5), 92.2 (d, $J_{C–F}$ = 187.2 Hz), 81.4, 62.0, 34.5, 27.6 (d, $J_{C–F}$ = 5.7 Hz), 15.58 (d, $J_{C–F}$ = 2.9 Hz), 14.2, (for minor diastereomer) δ 168.5 (d, $J_{C–F}$ = 24.6 Hz), 137.7, 137.5, 135.0, 134.9, 129.9, 129.8, 129.3, 90.7 (d, $J_{C–F}$ = 188.0 Hz), 81.5, 61.9, 34.6, 28.8, 14.3, 13.2 (d, $J_{C–F}$ = 4.4 Hz); HRMS (ESI) Calcd for $C_{20}H_{23}O_6FNaS_2^+$ ([M+Na]$^+$) 465.0812. Found 465.0820.

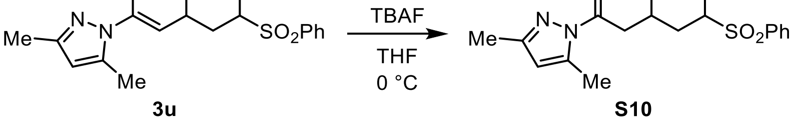

3u **S10**

To a solution of **3u** (41.2 mg, 0.07 mmol, 1 equiv) in THF (0.7 mL, 0.1 M) was added a 1 M THF solution of TBAF (70 µL, 0.07 mmol, 1 equiv) dropwise at 0 °C. The reaction mixture was stirred at 0 °C for 1 h and

then quenched with saturated aqueous solution of $NaHCO_3$. The extractive work-up was performed with EtOAc. The combined organic layers were dried over Na_2SO_4, filtered, and concentrated. The crude material was purified by column chromatography on silica gel (hexane/EtOAc = 3:1 as eluent) to afford **S10** in 96% yield (31.9 mg, 0.067 mmol). ^1H NMR (400 MHz, $CDCl_3$) δ 8.01 (2H, d, J = 7.2 Hz), 7.97 (2H, d, J = 7.2 Hz), 7.69 (1H, t, J = 7.6 Hz), 7.67 (1H, t, J = 7.2 Hz), 7.60-7.52 (4H, m), 5.96 (1H, s), 4.95 (1H, t, J = 5.4 Hz), 3.10 (1H, dd, J = 16.2, 4.8 Hz), 2.78 (1H, dd, J = 16.2, 7.4 Hz), 2.52 (3H, s), 2.40-2.31 (1H, m), 2.27-2.10 (2H, m), 2.21 (3H, s), 0.99 (3H, d, J = 6.8 Hz). ^{13}C NMR (151 MHz, $CDCl_3$) δ 172.4, 152.2, 144.3, 138.1, 137.7, 134.7, 130.0, 129.8, 129.2(0), 129.1(5), 111.4, 81.5, 41.8, 32.5, 29.2, 20.2, 14.7, 14.0; HRMS (ESI) Calcd for $C_{23}H_{26}O_5N_2NaS_2^+$ ($[M+Na]^+$) 497.1175. Found 497.1170.

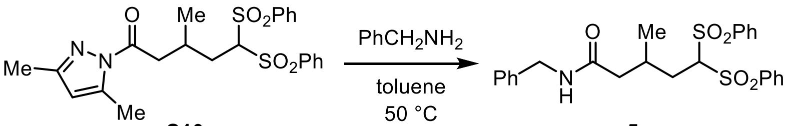

S10 **5g**

The solution of **S10** (47.5 mg, 0.10 mmol, 1 equiv) and benzylamine (22 μL, 0.20 mmol, 2 equiv) in toluene (0.4 mL, 0.25 M) was stirred at 50 °C for 3 h. After cooling to room temperature, the reaction was quenched with 1 N aqueous solution of HCl and extractive work-up was carried out with CH_2Cl_2. The combined organic layers were washed with saturated aqueous solution of $NaHCO_3$ and dried over Na_2SO_4, filtered, and concentrated. The crude product was purified by column chromatography on silica gel (hexane/EtOAc = 1:1) to afford **5g** in 88% yield (42.8 mg, 0.088 mmol). ^1H NMR (400 MHz, $CDCl_3$) δ 7.95-7.92 (4H, m), 7.69-7.65 (2H, m), 7.57-7.52 (4H, m), 7.34-7.30 (2H, m), 7.29-7.25 (3H, m), 5.93 (1H, brs), 4.86 (1H, t, J = 3.4 Hz), 4.45 (1H, dd, J = 15.2, 6.3 Hz), 4.41 (1H, dd, J = 15.2, 6.3 Hz), 2.30-2.17 (3H, m), 2.15-2.07 (2H, m), 0.92 (3H, d, J = 7.2 Hz); ^{13}C NMR (151 MHz, $CDCl_3$) δ 171.3, 138.2, 138.0, 137.7, 134.7(0), 134.6(7), 129.9, 129.6, 129.3, 129.2, 128.9, 128.0, 127.7, 81.4, 43.8, 43.5, 32.3, 29.5, 20.4; HRMS (ESI) Calcd for $C_{25}H_{26}O_5NS_2^-$ ($[M-H]^-$) 484.1247. Found 484.1251.

Square Wave Voltammetry

Square wave voltammetry (SWV) measurements of representative enol silyl ether **1a**, electron deficient olefin **2a**, and alkylated enol silyl ether **3a** were performed on ALS/CH Instruments Electrochemical Analyzer using a glassy carbon working electrode, a Pt wire counter electrode, and a ferrocene/ferrocenium reference electrode. Voltammograms were taken at room temperature in a 100 mM MeCN solution of tetrabutylammonium perchlorate ($Bu_4N{\cdot}ClO_4$) containing 1 mM of the designated substance. For conversion to the SCE couple, it is known that Fc/Fc^+ is 380 mV more positive than SCE in MeCN; this value was added from obtained potentials in Fc/Fc^+ to determine potentials against SCE.

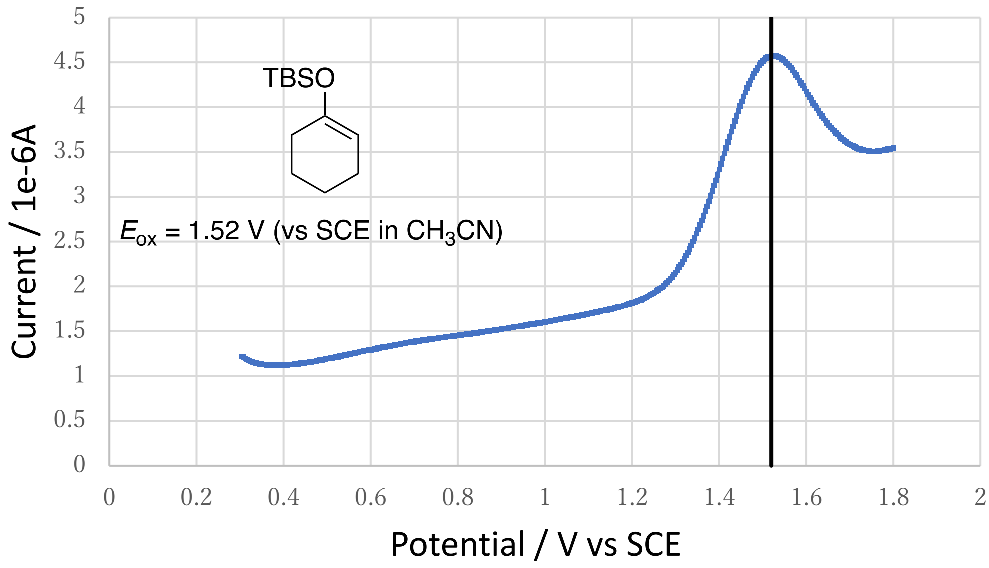

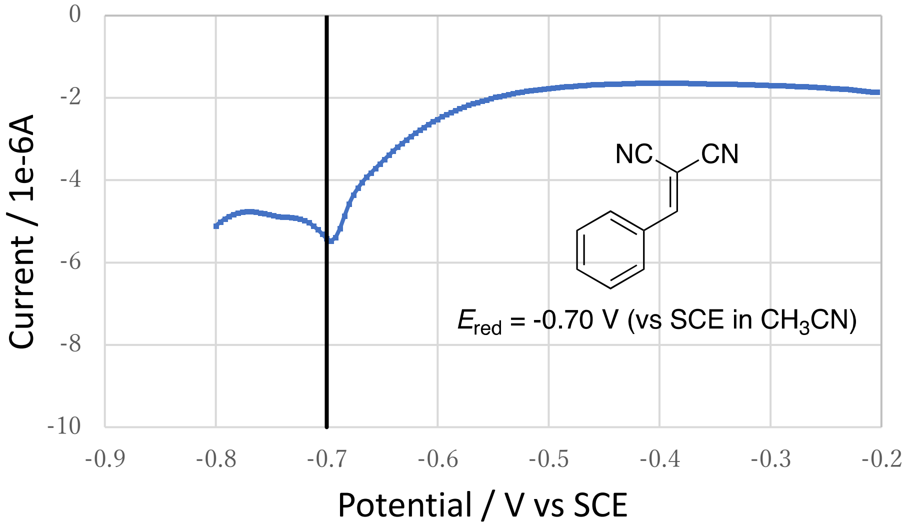

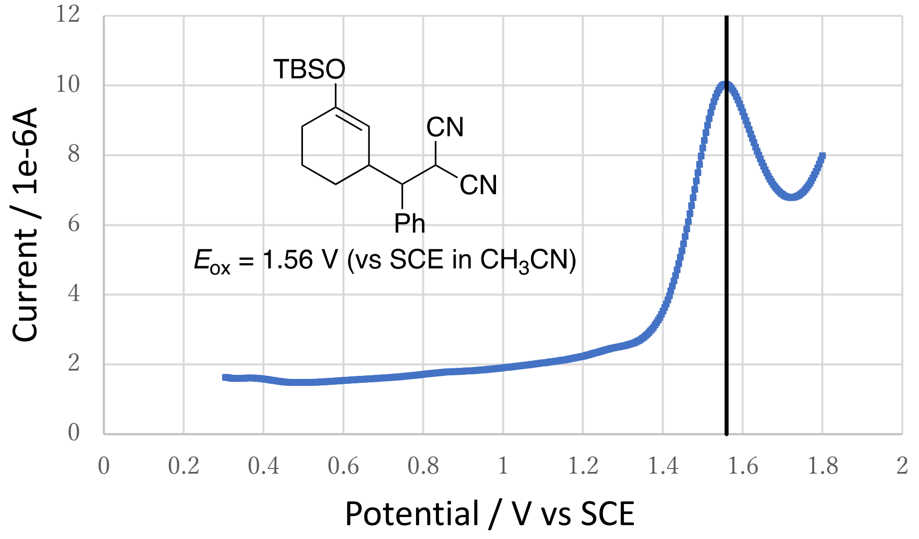

Supplementary Figure 2. SWV plots.

Stern-Volmer Experiments

Emission intensities were recorded on a HORIBA FluoroMax-4P spectrometer. The solutions were excited at 420 nm and the luminescence was measured at 564 nm. Stern-Volmer luminescence quenching experiments were run with freshly prepared a 0.1 mM solutions of [Ir(dF(CF_3)ppy)$_2$(4,4′-dCF_3bpy)]PF_6 in dry dichloroethane (DCE) at room temperature under N_2 atmosphere. In a typical experiment, the solution of Ir complex was added to an appropriate amount of quencher in 5 mL volumetric flask under N_2. The solution was transferred to 1 cm^2 quartz cell and the emission spectrum of the sample was collected. The data show that enol silyl ether **1a** and alkylated enol silyl ether **3a** are competent at quenching the excited state of the photocatalyst. On the other hand, benzalmalononitrile (**2a**) and 2,4,6-collidine are shown to be unable to quench this excited state.

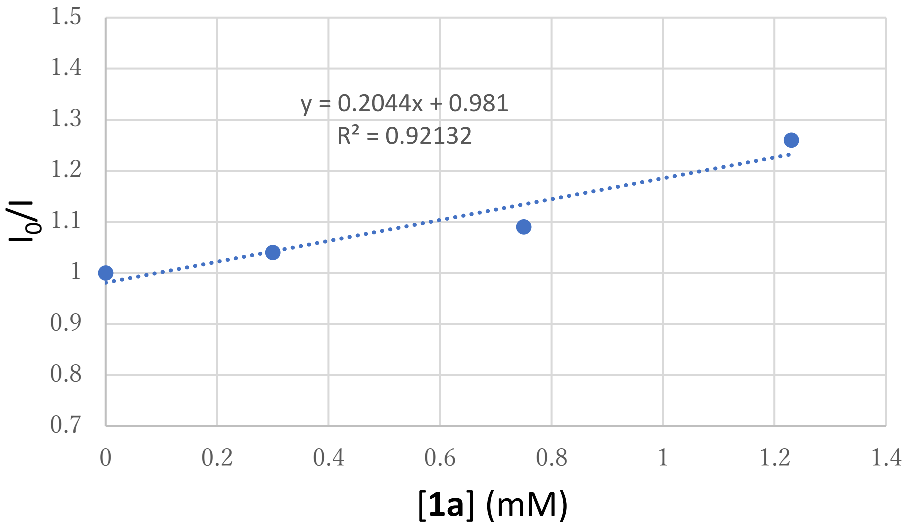

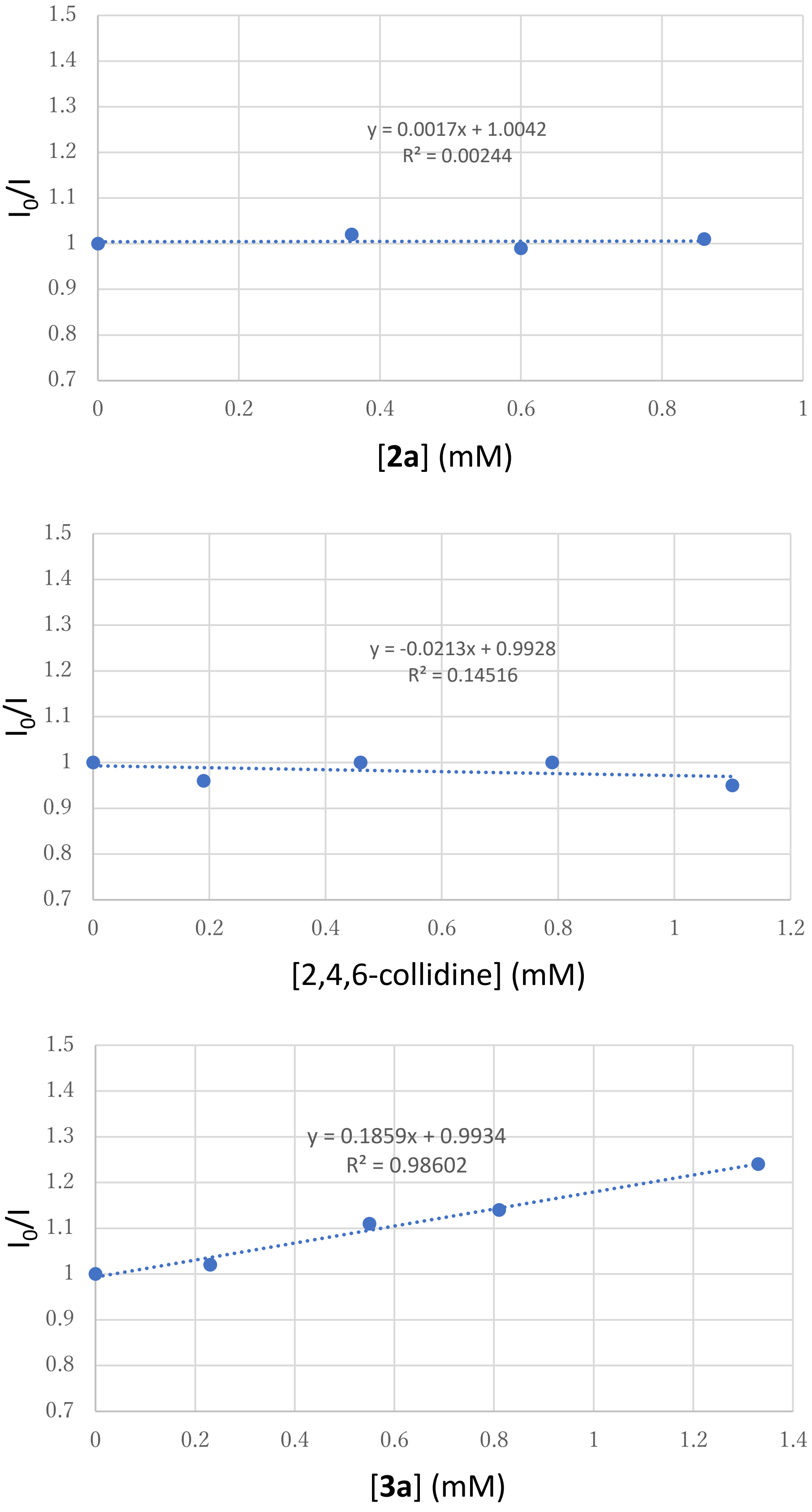

Supplementary Figure 3. Stern-Volmer Experiments.

Computational Details

The Gaussian 09 Revision D.01 program was used for all calculations.[6] The 3D molecular structures were depicted by using the CYLview v1.0.561 β.[7] Local minima and transition states (TS) were located by using the (U)CAM-B3LYP functional[8] with the 6-311+G(d,p) basis set in the dichloroethane (DCE) continuum solvent of the solvation model based on density (SMD)[9] and "int=ultrafine" option to aid in better description of a DFT grid. As discussed in the main text, we need to estimate Kohn-Sham SOMO levels of TBS enol ether radicals for comparing the radical reactivity in the addition to olefins.

Since no global functional to treat radical cations properly was reported so far, in addition to CAM-B3LYP, B3LYP[10] (one of the most popular functional for calculating organic molecules) and LC-BLYP[11] (used in predicting several orbital properties of radical cationic systems[12]) were also examined for comparison. Harmonic frequency analyses were also performed to identify the stationary points (no/one imaginary frequency for local minima/TSs) and to estimate thermodynamic parameters at 298.15 K and 1 atm. Intrinsic reaction coordinate (IRC) analyses were conducted on the representative TS structures to confirm that the desired chemical transformations occurred through the TSs. The Cartesian coordinates and energies of optimized structures are summarized in the Supplementary Data 1.

Estimation of pK_a of Enol Silyl Ether Radical Cations

It has been recognized that direct determination of pK_a of radical cations, based on thermochemical cycle, deviated by several unit, while relative tendency was reproduced. Therefore, statistical manipulation is required to obtain the pK_a values comparable with the experimentally determined values.[13,14]

Here, the pK_a values of radical cation [**1a**]$^{•+}$ of cyclohexanone-derived enol silyl ether was simply estimated by considering acid-base equilibria with various substituted anilines **B(X)**, whose pK_a were experimentally determined (range of –6–16 in MeCN), and tetracyanopropenes **TCNP(X)** (range of –3-5 in MeCN). A linear free energy plot was shown in Supplementary Figure 4 and 5, where the x-axis is the calculated reaction free energy (ΔG) of the equilibrium eq. 1 or eq. 2 with MeCN of the SMD solvent model and the y-axis is experimental pK_a values of substituted anilines or tetracyanopropenes. Both plots show excellent linear correlations: R^2 = 0.988 with a slope = –0.6 and intercept = 8.4 for eq. 1 and R^2 = 0.997 with a slope = –0.7 and intercept = 8.4 for eq. 2. According to the equilibrium eq. 1 and 2, the intercept of the plot corresponds to pK_a of [**1a**]$^{•+}$: pK_a (MeCN) = 8.4.

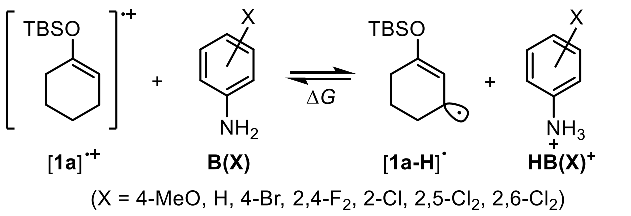

(1)

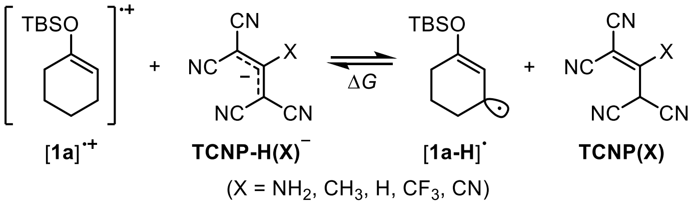

(2)

These analyses were also applied to linear TBS enol ethers **1o** (R = *t*Bu), **1r** (R = Mes), and **1s** (R = Ph), and their pK_a were determined to be 8.4 (**1o**), 6.5 (**1r**), and 4.8 (**1s**), respectively (eq. 3, Supplementary Table 1-6). B3LYP and LC-BLYP always give higher and lower pK_a values compared with CAM-B3LYP, and the difference becomes larger for aryl substituted systems ([**1r**]$^{•+}$ and [**1s**]$^{•+}$). While the absolute pK_a values depend on the functionals and the reference systems, the acidity order of the radical cations ([**1a**]$^{•+}$ ≈ [**1o**]$^{•+}$ < [**1r**]$^{•+}$ < [**1s**]$^{•+}$) is not changed. Therefore, the conclusion that [**1s**]$^{•+}$ is the most acidic of all calculated radical cations would be acceptable.

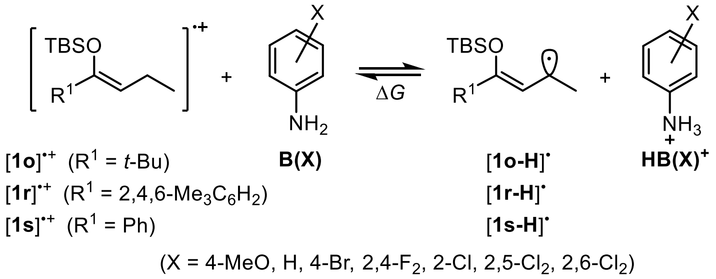

(3)

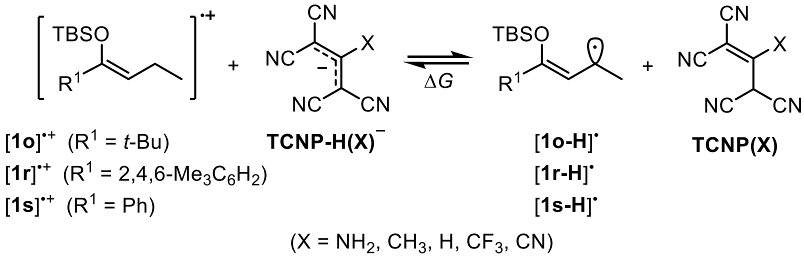

(4)

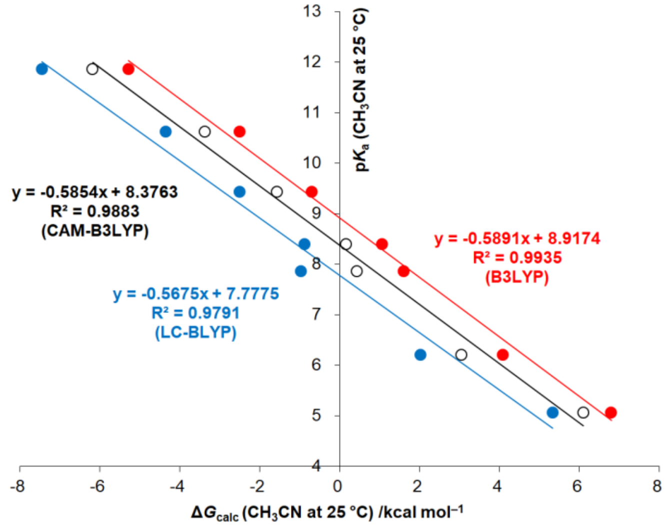

Supplementary Figure 4. Linear free energy plot of eq. 1.

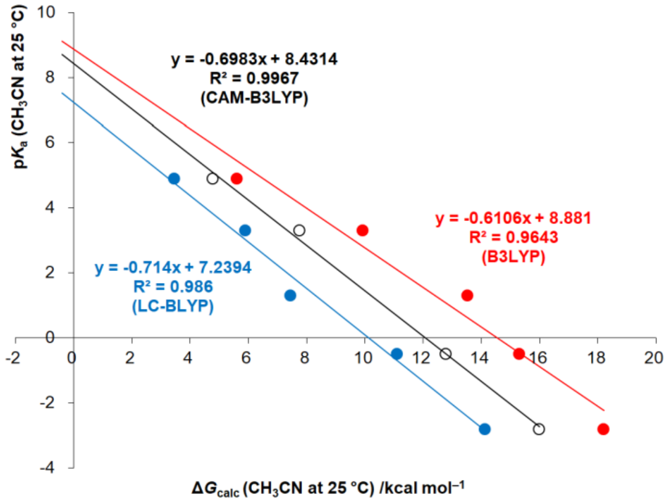

Supplementary Figure 5. Linear free energy plot of eq. 2.

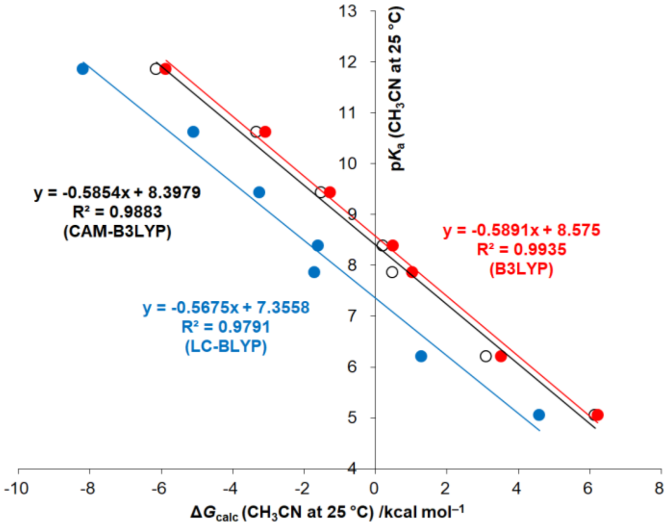

Supplementary Figure 6. Linear free energy plot of eq. 3 (R^1 = *t*-Bu).

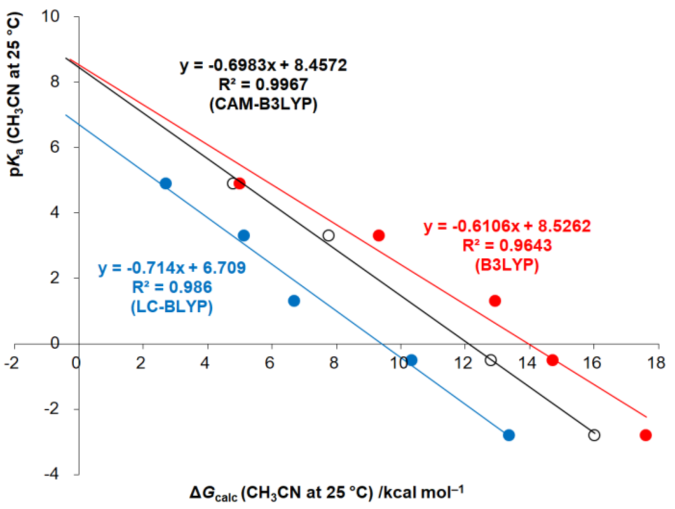

Supplementary Figure 7. Linear free energy plot of eq. 4 (R^1 = *t*-Bu).

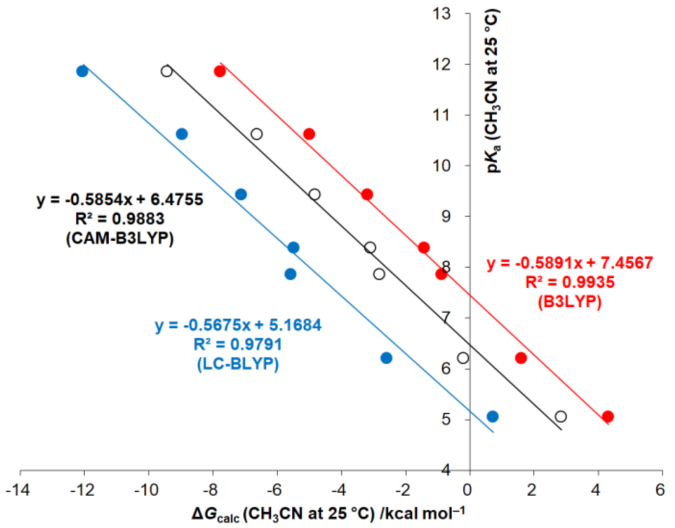

Supplementary Figure 8. Linear free energy plot of eq. 3 (R^1 = Mes).

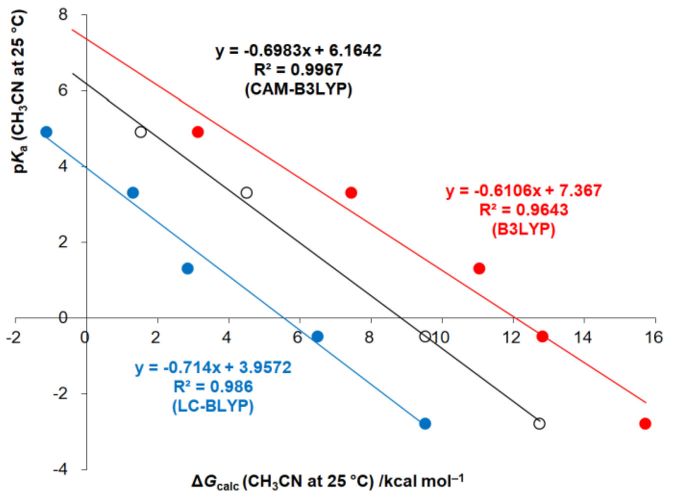

Supplementary Figure 9. Linear free energy plot of eq. 4 (R^1 = Mes).

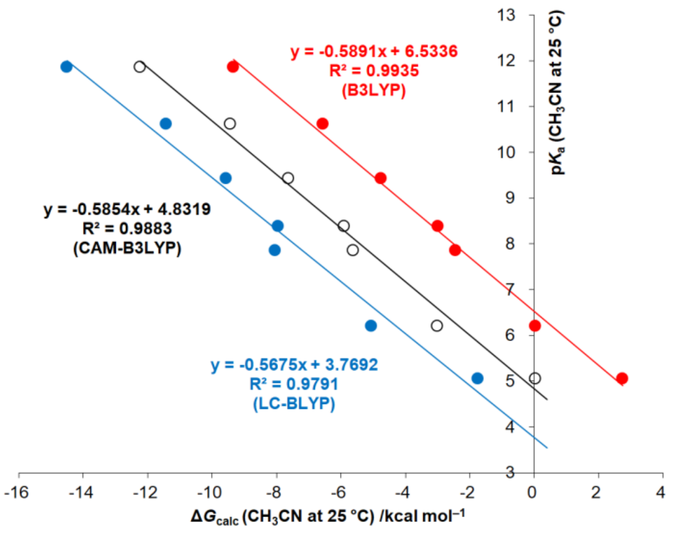

Supplementary Figure 10. Linear free energy plot of eq. 3 (R^1 = Ph).

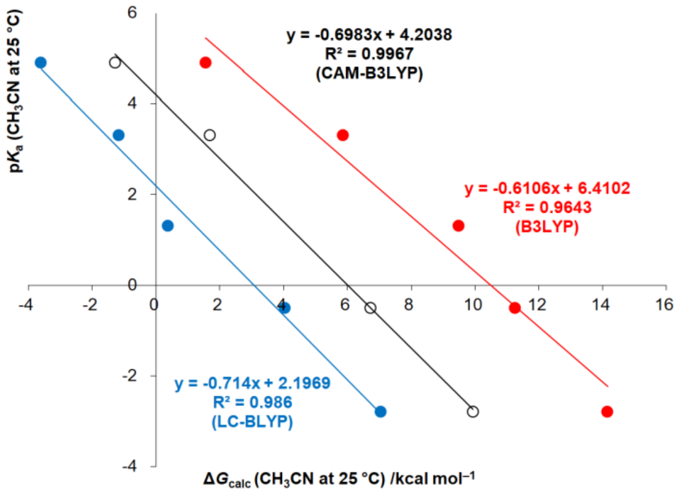

Supplementary Figure 11. Linear free energy plot of eq. 4 (R^1 = Ph).

Supplementary Table 1. Raw Gibbs free energy at SMD(MeCN)-CAM-B3LYP/6-311+G(d,p) and Gibbs free energy change in the equilibrium of eq. 1 and 3

	G /au		G /au	ΔG /kcal mol^{-1}				pK_a(CH_3CN)^a
				[**1a**]$^{\bullet+}$	[**1o**]$^{\bullet+}$	[**1r**]$^{\bullet+}$	[**1s**]$^{\bullet+}$	
TBS enol ether		***anilines***						
[**1a**]$^{\bullet+}$	-835.887634	**B(4-MeO)**	-401.928893	-6.18	-6.14	-9.43	-12.23	11.86
[**1a-H**]$^{\bullet}$	-835.461696	**HB(4-MeO)$^+$**	-402.364678					
[**1o**]$^{\bullet+}$	-915.608993	**B(H)**	-287.447382	-3.37	-3.33	-6.62	-9.42	10.62
[**1o-H**]$^{\bullet}$	-915.182996	**HB(H)$^+$**	-287.878690					
[**1r**]$^{\bullet+}$	-1107.248251	**B(4-Br)**	-2861.107170	-1.56	-1.52	-4.81	-7.62	9.43
[**1r-H**]$^{\bullet}$	-1106.827487	**HB(4-Br)$^+$**	-2861.535596					
[**1s**]$^{\bullet+}$	-989.422853	**B(2,4-F_2)**	-485.966607	0.16	0.20	-3.08	-5.89	8.39
[**1s-H**]$^{\bullet}$	-989.006563	**HB(2,4-F_2)$^+$**	-486.392287					
		**B(2-Cl)**	-747.085815	0.44	0.47	-2.81	-5.62	7.86
		**HB(2-Cl)$^+$**	-747.511057					
		**B(2,5-Cl_2)**	-1206.724615	3.05	3.09	-0.20	-3.00	6.21
		**HB(2,5-Cl_2)$^+$**	-1207.145693					
		**B(2,6-Cl_2)**	-1206.722090	6.10	6.14	2.86	0.05	5.06
		**HB(2,6-Cl_2)$^+$**	-1207.138304					

^{a} See ref 11

Supplementary Table 2. Raw Gibbs free energy at SMD(MeCN)-CAM-B3LYP/6-311+G(d,p) and Gibbs free energy change in the equilibrium of eq. 2 and 4

	G /au		G /au	ΔG /kcal mol^{-1}				pK_a(CH_3CN)^a
				[**1a**]$^{\bullet+}$	[**1o**]$^{\bullet+}$	[**1r**]$^{\bullet+}$	[**1s**]$^{\bullet+}$	
TBS enol ether		***tetracyanopropene***						
[**1a**]$^{\bullet+}$	-835.887634	**TCNP(NH_2)**	-542.072517	4.77	4.81	1.52	-1.28	4.90
[**1a-H**]$^{\bullet}$	-835.461696	**TCNP-H(NH_2)$^-$**	-541.654181					
[**1o**]$^{\bullet+}$	-915.608993	**TCNP(Me)**	-525.990880	7.75	7.78	4.50	1.69	3.30
[**1o-H**]$^{\bullet}$	-915.182996	**TCNP-H(Me)$^-$**	-525.577285					
[**1r**]$^{\bullet+}$	-1107.248251	**TCNP(H)**	*nd*^{b}					1.30
[**1r-H**]$^{\bullet}$	-1106.827487	**TCNP-H(N)$^-$**	-486.300151					
[**1s**]$^{\bullet+}$	-989.422853	**TCNP(CF_3)**	-823.758622	12.77	12.81	9.53	6.72	-0.50
[**1s-H**]$^{\bullet}$	-989.006563	**TCNP-H(CF_3)$^-$**	-823.353040					
		**TCNP(CN)**	-578.917068	15.99	16.03	12.74	9.94	-2.80
		**TCNP-H(CN)$^-$**	-578.516613					

^{a} See ref 12

^{b} Converged to 1st order saddle point and not used for the LFER plot

Supplementary Table 3. Raw Gibbs free energy at SMD(MeCN)-(U)B3LYP/6-311+G(d,p) and Gibbs free energy change in the equilibrium of eq. 1 and 3

				ΔG /kcal mol^{-1}				pK_a(CH_3CN)[a]
	G /au		*G* /au	[**1a**]$^{\cdot+}$	[**1o**]$^{\cdot+}$	[**1r**]$^{\cdot+}$	[**1s**]$^{\cdot+}$	
TBS enol ether		***anilines***						
[**1a**]$^{\cdot+}$	-836.226460	**B(4-MeO)**	-402.141285	-5.29	-5.87	-7.77	-9.33	11.86
[**1a-H**]$^{\cdot}$	-835.797546	**HB(4-MeO)$^+$**	-402.578624					
[**1o**]$^{\cdot+}$	-916.002889	**B(H)**	-287.614588	-2.51	-3.09	-4.98	-6.55	10.62
[**1o-H**]$^{\cdot}$	-915.574901	**HB(H)$^+$**	-288.047495					
[**1r**]$^{\cdot+}$	-1107.754289	**B(4-Br)**	-2861.173071	-0.70	-1.28	-3.18	-4.74	9.43
[**1r-H**]$^{\cdot}$	-1107.329326	**HB(4-Br)$^+$**	-2861.603094					
[**1s**]$^{\cdot+}$	-989.857161	**B(2,4-F_2)**	-486.166904	1.06	0.48	-1.42	-2.99	8.39
[**1s-H**]$^{\cdot}$	-989.434695	**HB(2,4-F_2)$^+$**	-486.594127					
		**B(2-Cl)**	-747.249464	1.61	1.03	-0.87	-2.43	7.86
		**HB(2-Cl)$^+$**	-747.675809					
		**B(2,5-Cl_2)**	-1206.884794	4.09	3.51	1.61	0.05	6.21
		**HB(2,5-Cl_2)$^+$**	-1207.307185					
		**B(2,6-Cl_2)**	-1206.882174	6.80	6.22	4.32	2.75	5.06
		**HB(2,6-Cl_2)$^+$**	-1207.300252					

[a] See ref 11

Supplementary Table 4. Raw Gibbs free energy at SMD(MeCN)-(U)B3LYP/6-311+G(d,p) and Gibbs free energy change in the equilibrium of eq. 2 and 4

				ΔG /kcal mol^{-1}				pK_a(CH_3CN)[a]
	G /au		*G* /au	[**1a**]$^{\cdot+}$	[**1o**]$^{\cdot+}$	[**1r**]$^{\cdot+}$	[**1s**]$^{\cdot+}$	
TBS enol ether		***tetracyanopropene***						
[**1a**]$^{\cdot+}$	-836.226460	**TCNP(NH_2)**	-542.338339	5.61	5.03	3.13	1.56	11.86
[**1a-H**]$^{\cdot}$	-835.797546	**TCNP-H(NH_2)$^-$**	-541.918360					
[**1o**]$^{\cdot+}$	-916.002889	**TCNP(Me)**	-526.258520	9.92	9.34	7.44	5.87	10.62
[**1o-H**]$^{\cdot}$	-915.574901	**TCNP-H(Me)$^-$**	-525.845410					
[**1r**]$^{\cdot+}$	-1107.754289	**TCNP(H)**	-486.952191	13.53	12.95	11.05	9.48	9.43
[**1r-H**]$^{\cdot}$	-1107.329326	**TCNP-H(N)$^-$**	-486.544838					
[**1s**]$^{\cdot+}$	-989.857161	**TCNP(CF_3)**	-824.074635	15.31	14.73	12.83	11.26	8.39
[**1s-H**]$^{\cdot}$	-989.434695	**TCNP-H(CF_3)$^-$**	-823.670116					
		**TCNP(CN)**	-579.201573	18.20	17.62	15.72	14.16	7.86
		**TCNP-H(CN)$^-$**	-578.801669					

[a] See ref 12

Supplementary Table 5. Raw Gibbs free energy at SMD(MeCN)-(U)LC-BLYP/6-311+G(d,p) and Gibbs free energy change in the equilibrium of eq. 1 and 3

	G /au		G /au	ΔG /kcal mol^{-1}				pK_a(CH$_3$CN)^a
				**[1a]**$^{•+}$	**[1o]**$^{•+}$	**[1r]**$^{•+}$	**[1s]**$^{•+}$	
TBS enol ether		***anilines***						
**[1a]**$^{•+}$	-834.331589	**B(4-MeO)**	-401.015234	-7.44	-8.19	-12.04	-14.51	11.86
**[1a-H]**$^{•}$	-833.910699	**HB(4-MeO)**$^{+}$	-401.447988					
**[1o]**$^{•+}$	-913.840835	**B(H)**	-286.745068	-4.36	-5.10	-8.95	-11.42	10.62
**[1o-H]**$^{•}$	-913.421129	**HB(H)**$^{+}$	-287.172899					
**[1r]**$^{•+}$	-1104.996066	**B(4-Br)**	-2859.902665	-2.50	-3.25	-7.10	-9.57	9.43
**[1r-H]**$^{•}$	-1104.582502	**HB(4-Br)**$^{+}$	-2860.327543					
**[1s]**$^{•+}$	-987.472908	**B(2,4-F$_2$)**	-485.028459	-0.87	-1.61	-5.47	-7.93	8.39
**[1s-H]**$^{•}$	-987.063273	**HB(2,4-F$_2$)**$^{+}$	-485.450734					
		**B(2-Cl)**	-746.120811	-0.97	-1.71	-5.57	-8.03	7.86
		**HB(2-Cl)**$^{+}$	-746.543245					
		**B(2,5-Cl$_2$)**	-1205.496649	2.02	1.28	-2.57	-5.04	6.21
		**HB(2,5-Cl$_2$)**$^{+}$	-1205.914312					
		**B(2,6-Cl$_2$)**	-1205.494467	5.33	4.58	0.73	-1.74	5.06
		**HB(2,6-Cl$_2$)**$^{+}$	-1205.906867					

[a] See ref 11

Supplementary Table 6. Raw Gibbs free energy at SMD(MeCN)-(U)LC-BLYP/6-311+G(d,p) and Gibbs free energy change in the equilibrium of eq. 2 and 4

	G /au		G /au	ΔG /kcal mol^{-1}				pK_a(CH$_3$CN)^a
				**[1a]**$^{•+}$	**[1o]**$^{•+}$	**[1r]**$^{•+}$	**[1s]**$^{•+}$	
TBS enol ether		***tetracyanopropene***						
**[1a]**$^{•+}$	-834.331589	**TCNP(NH$_2$)**	-540.871671	3.46	2.72	-1.14	-3.60	11.86
**[1a-H]**$^{•}$	-833.910699	**TCNP-H(NH$_2$)**$^{-}$	-540.456294					
**[1o]**$^{•+}$	-913.840835	**TCNP(Me)**	-524.793872	5.90	5.15	1.30	-1.16	10.62
**[1o-H]**$^{•}$	-913.421129	**TCNP-H(Me)**$^{-}$	-524.382381					
**[1r]**$^{•+}$	-1104.996066	**TCNP(H)**	-485.613673	7.44	6.70	2.85	0.38	9.43
**[1r-H]**$^{•}$	-1104.582502	**TCNP-H(N)**$^{-}$	-485.204643					
**[1s]**$^{•+}$	-987.472908	**TCNP(CF$_3$)**	-822.207167	11.10	10.35	6.50	4.03	8.39
**[1s-H]**$^{•}$	-987.063273	**TCNP-H(CF$_3$)**$^{-}$	-821.803961					
		**TCNP(CN)**	-577.622417	14.12	13.37	9.52	7.06	7.86
		**TCNP-H(CN)**$^{-}$	-577.224025					

[a] See ref 12

Calculation for Radical Addition

(TBS enol ether radical to 1,1-bis(phenylsulfonyl)ethylene)

The reactivity of enol silyl ether-derived allylic radicals was examined by calculating frontier orbital properties and TS of radical addition to 1,1-bis(phenylsulfonyl)ethylene (eq. 5).

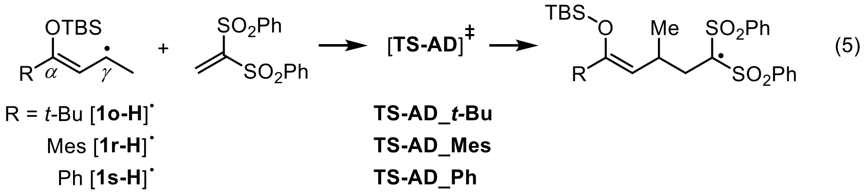

Optimized structures of these radicals at UCAM-B3LYP/6-311+G(d,p) level were summarized in Supplementary Figure 12. As described in the main manuscript, spin density at the allylic carbon of each radical correlates with the experimental yield: 0.70 ([**1o-H**]•), 0.64 ([**1r-H**]•), and 0.51 ([**1s-H**]•). For [**1s-H**]•, the spin is localized to the Ph group, which is reflected in the bond distance between the oxygen-bearing carbon and the carbon of the Ph group (***a*** in Supplementary Figure 12). The C–C distance changes from 1.52 Å to 1.45 Å by substituting *t*-Bu to Ph, indicating the existence of radical delocalization toward an aromatic Ph ring. When Mes is introduced instead of Ph, ***a*** is elongated again from 1.45 Å to 1.48 Å, and the spin density at the γ-carbon increases from 0.51 to 0.64. The Mes group cannot adopt a coplanar orientation with the olefin moiety due to the steric repulsion caused by the two *o*-Me groups (the dihedral angle of $C_{ortho}C_{ipso}$-$C_{\alpha}C_{\beta}$ is 60.1°; highlighted in green in Supplementary Figure 12). Since radical conjugation with the Mes group is partially inhibited in [**1r-H**]•, its spin density at the allylic position and reactivity are comparable with those of [**1o-H**]•.

In exploring the most stable TS for the radical addition, 8 kinds of conformers were examined: different orientations of 1) radical and olefin fragments about the forming C–C bond and 2) SO_2Ph groups in olefin about the approaching radical (Supplementary Figure 13-15, Supplementary Table 7). The lowest values of $\Delta G^{\ddagger}$ for each substituent (21.2 kcal mol^{-1} (**TS-AD_*t*-Bu_1b**), 21.6 kcal mol^{-1} (**TS-AD_Mes_2b**), and 23.3 kcal mol^{-1} (**TS-AD_Ph_2b**)) are reported in Table 2 in the main text. These values correlate with the spin density of the C3-carbon and the experimental yield (90% (**1o**), 81% (**1r**), 53% (**1s**)). The proper balance of the radical cation acidity and the activation barrier of the radical addition may be a source of the superior reactivity of **1r** in the C–H alkylation.

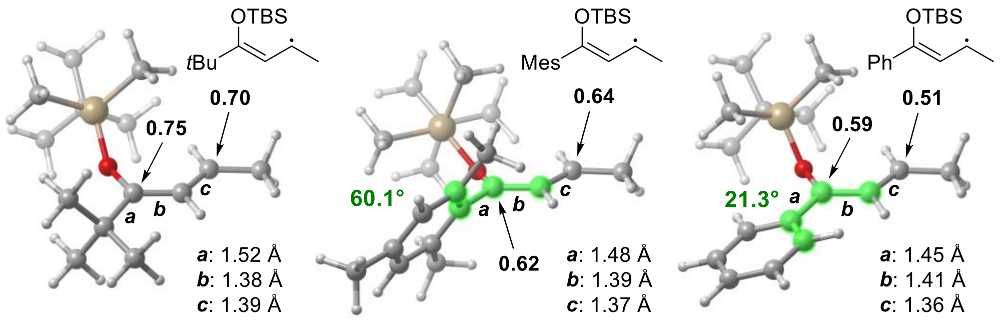

Supplementary Figure 12. Optimized structures of [**1o-H**]$^{\bullet}$, [**1r-H**]$^{\bullet}$, and [**1s-H**]$^{\bullet}$ at UCAM-B3LYP/6-311+G(d,p). Representative bond distances, spin densities, and dihedral angles between an aromatic ring and an olefin moiety (green) are also shown for [**1r-H**]$^{\bullet}$ and [**1s-H**]$^{\bullet}$.

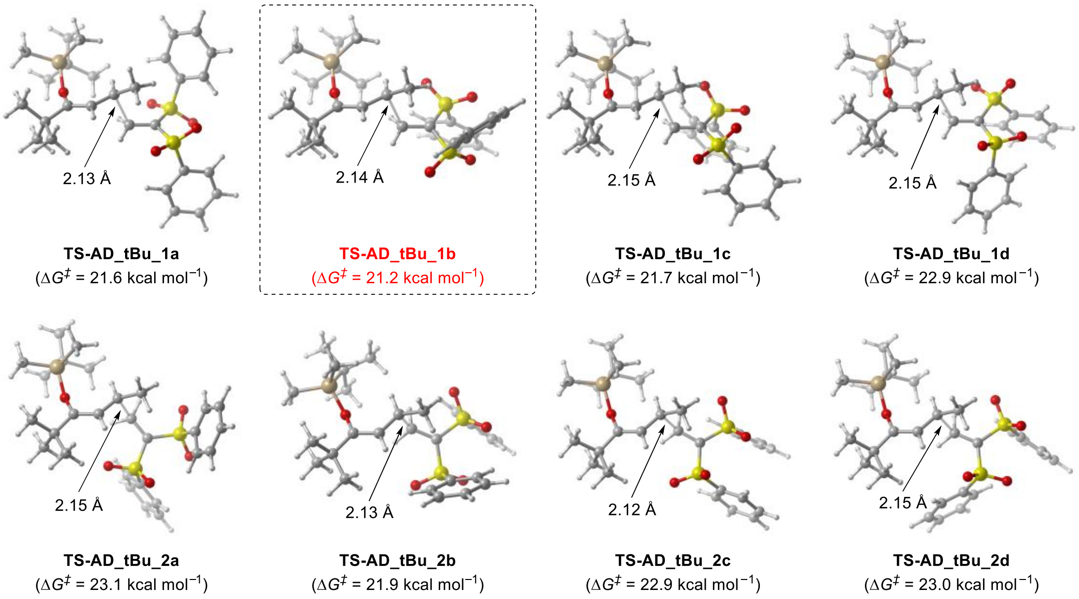

Supplementary Figure 13. Optimized TS structures of radical addition of [**1o-H**]$^{\bullet}$ to 1,1-bis(phenylsulfonyl)ethylene.

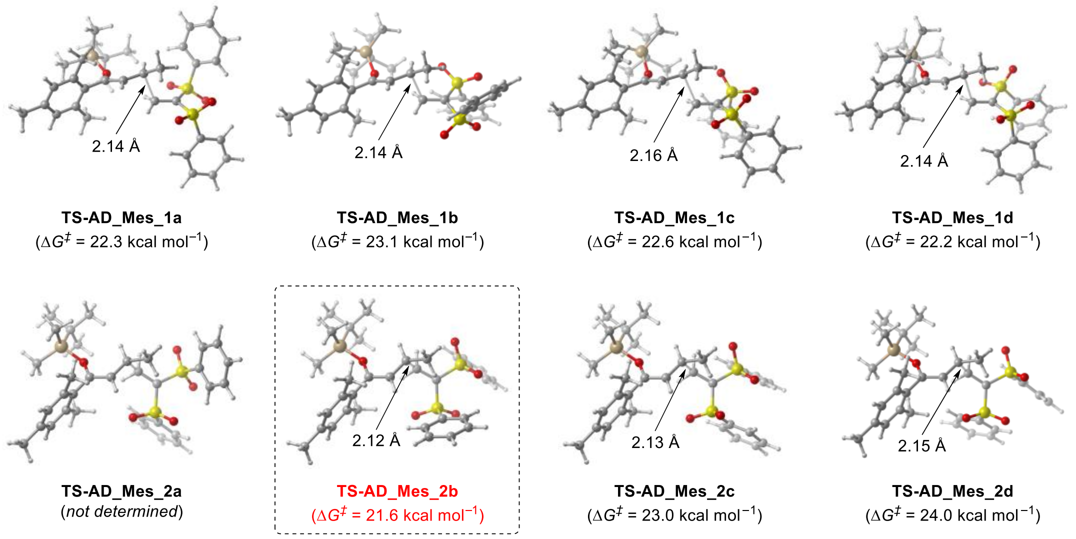

Supplementary Figure 14. Optimized TS structures of radical addition of [**1r-H**]$^{\bullet}$ to 1,1-bis(phenylsulfonyl)ethylene.

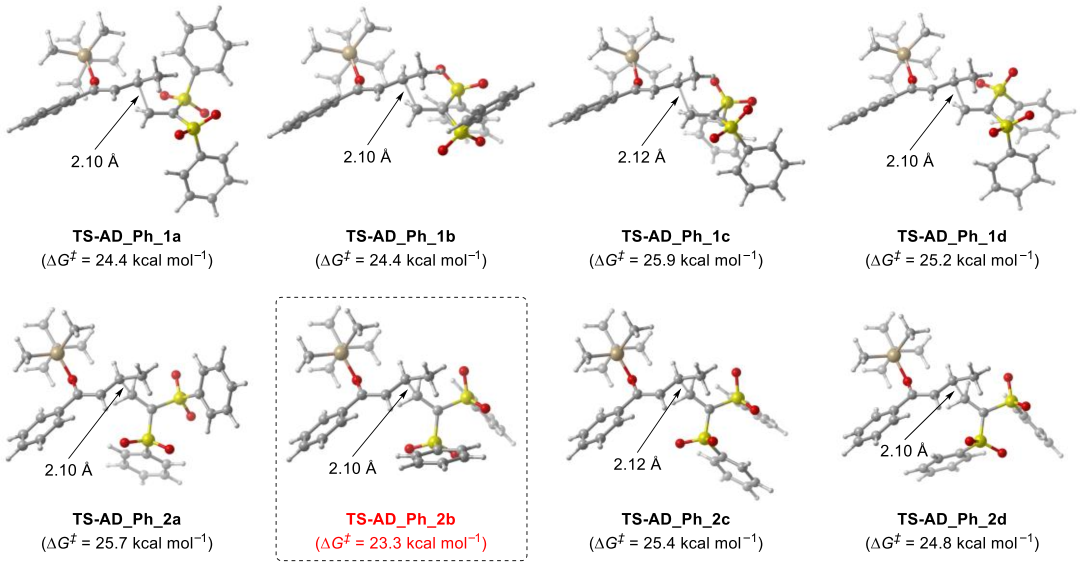

Supplementary Figure 15. Optimized TS structures of radical addition of [**1s-H**]$^{\bullet}$ to 1,1-bis(phenylsulfonyl)ethylene.

Supplementary Table 7. Electron energy, enthalpy, entropy, Gibbs free energy, relative electron energy and relative Gibbs free energy of eq. S5 at SMD(CH_3CN)-(U)CAM-B3LYP/6-311+G(d,p)

(R^1, R^2)	E /au	H /au	S /eu	G /au	ΔE /kcal mol^{-1}	ΔG /kcal mol^{-1}
1,1-bis(phenylsulfonyl)ethylene	-1637.714023	-1637.458874	139.527	-1637.525168		
[**1o-H**]$^•$	-915.532276	-915.108818	156.782	-915.183310		
**TS-AD_tBu_1a**	-2553.236443	-2552.555665	249.277	-2552.674104	6.18	21.57
**TS-AD_tBu_1b**[a]	-2553.235171	-2552.554233	253.409	-2552.674636	6.98	21.24
**TS-AD_tBu_1c**	-2553.233933	-2552.553140	254.256	-2552.673945	7.76	21.67
**TS-AD_tBu_1d**	-2553.233498	-2552.552567	251.254	-2552.671946	8.03	22.92
**TS-AD_tBu_2a**	-2553.233131	-2552.552260	251.276	-2552.671650	8.26	23.11
**TS-AD_tBu_2b**	-2553.234706	-2552.553594	252.479	-2552.673555	7.27	21.91
**TS-AD_tBu_2c**	-2553.233127	-2552.552212	252.024	-2552.671957	8.27	22.92
**TS-AD_tBu_2d**	-2553.233387	-2552.552397	251.324	-2552.671809	8.10	23.01
[**1r-H**]$^•$	-1107.221441	-1106.742045	180.532	-1106.827822		
**TS-AD_Mes_1a**	-2744.925381	-2744.188832	270.677	-2744.317439	6.33	22.31
**TS-AD_Mes_1b**	-2744.923264	-2744.186774	272.275	-2744.316140	7.66	23.12
**TS-AD_Mes_1c**	-2744.921565	-2744.185228	277.444	-2744.317050	8.72	22.55
**TS-AD_Mes_1d**	-2744.921742	-2744.185555	277.911	-2744.317599	8.61	22.21
**TS-AD_Mes_2a**	=> converged to **TS-AD_Mes_1a**					
**TS-AD_Mes_2b**[a]	-2744.923877	-2744.187270	276.201	-2744.318502	7.27	21.64
**TS-AD_Mes_2c**	-2744.920545	-2744.184310	278.007	-2744.316400	9.36	22.96
**TS-AD_Mes_2d**	-2744.919603	-2744.183163	277.032	-2744.314790	9.95	23.97
[**1s-H**]$^•$	-989.323605	-988.932114	156.857	-989.006642		
**TS-AD_Ph_1a**	-2627.023676	-2626.374740	248.702	-2626.492907	8.76	24.41
**TS-AD_Ph_1b**	-2627.022089	-2626.373270	251.686	-2626.492854	9.75	24.45
**TS-AD_Ph_1c**	-2627.021267	-2626.372448	248.423	-2626.490482	10.27	25.93
**TS-AD_Ph_1d**	-2627.020483	-2626.371708	252.369	-2626.491617	10.76	25.22
**TS-AD_Ph_2a**	-2627.018974	-2626.370125	253.966	-2626.490792	11.71	25.74
**TS-AD_Ph_2b**[a]	-2627.021894	-2626.373039	255.865	-2626.494609	9.87	23.34
**TS-AD_Ph_2c**	-2627.018844	-2626.370112	255.047	-2626.491293	11.79	25.42
**TS-AD_Ph_2d**	-2627.020143	-2626.371438	254.258	-2626.492244	10.97	24.83

[a] Reported in the main text (Table 3).

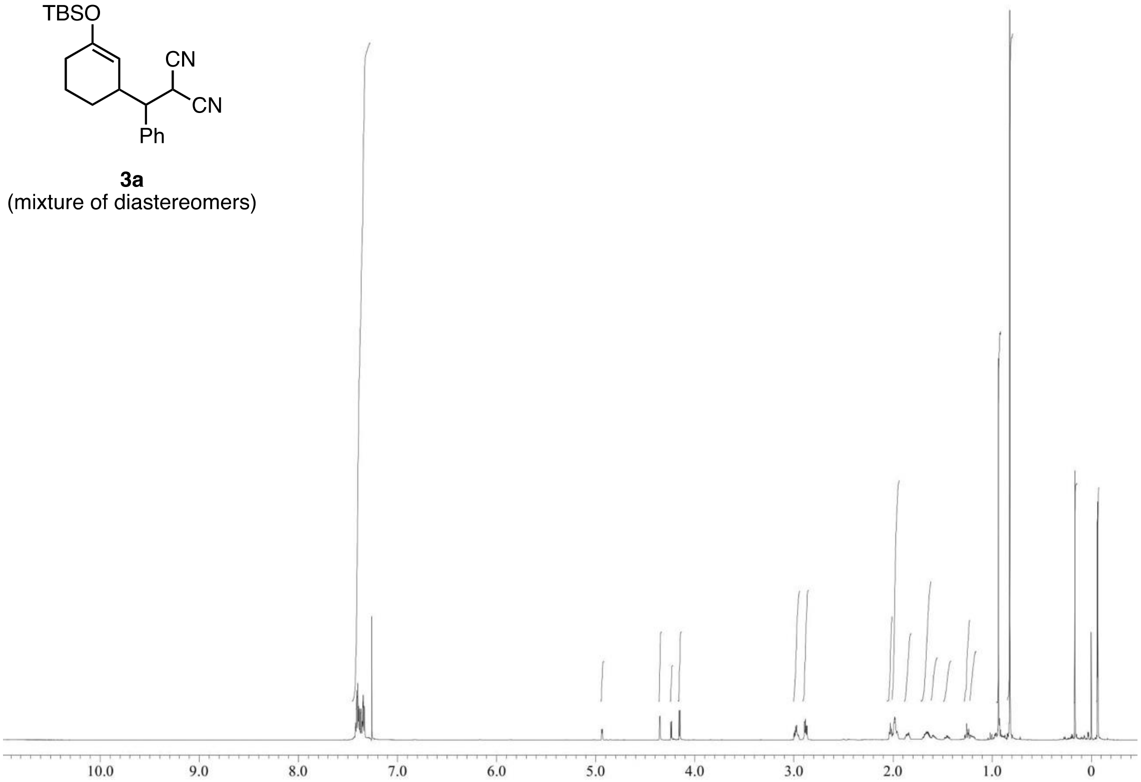

Supplementary Figure 16. ^{1}H NMR spectra of **3a** (mixture of diastereomers).

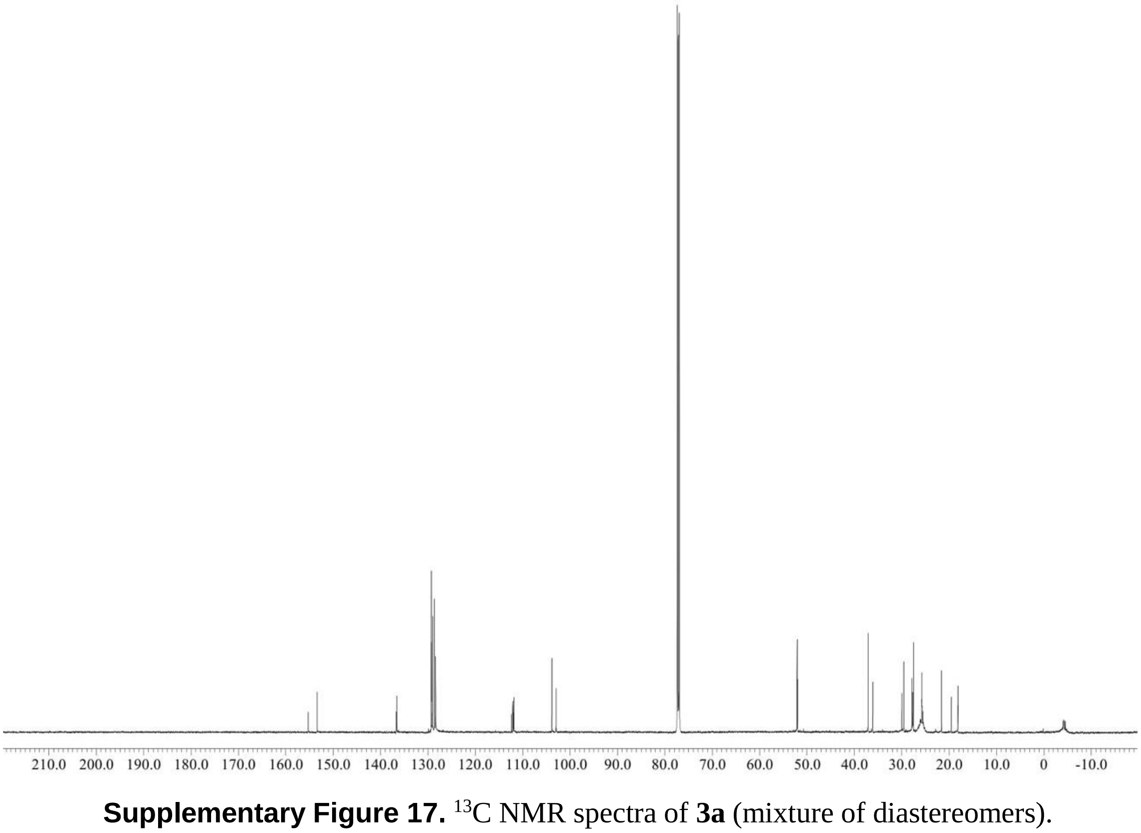

Supplementary Figure 17. ^{13}C NMR spectra of **3a** (mixture of diastereomers).

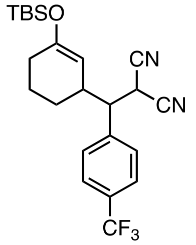

3b
(major diastereomer)

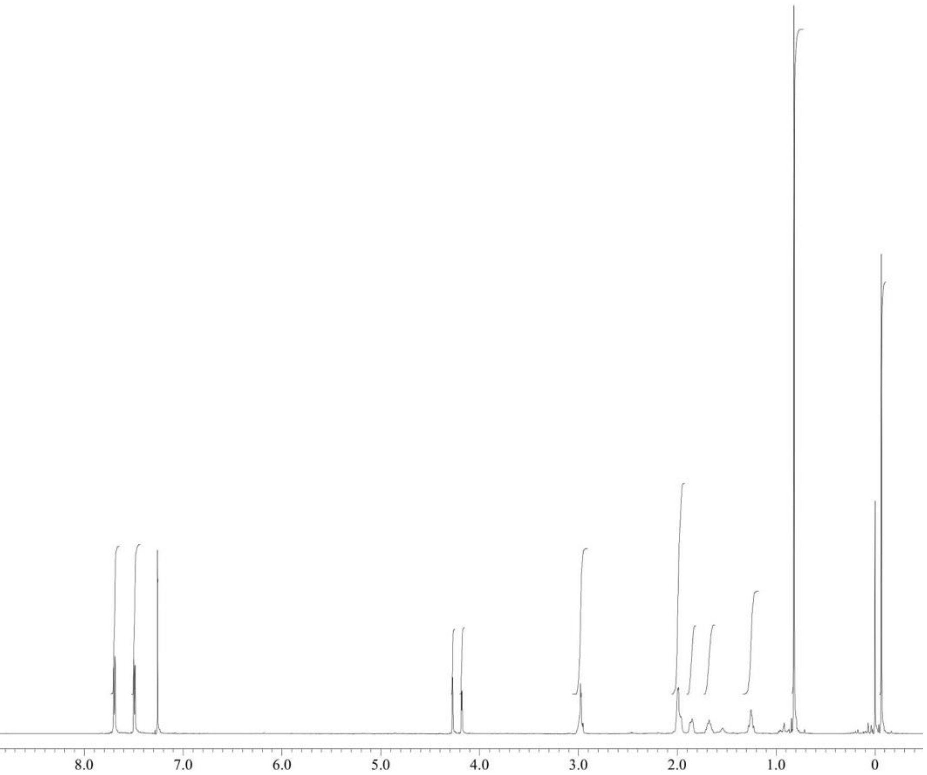

Supplementary Figure 18. ^{1}H NMR spectra of **3b** (major diastereomer).

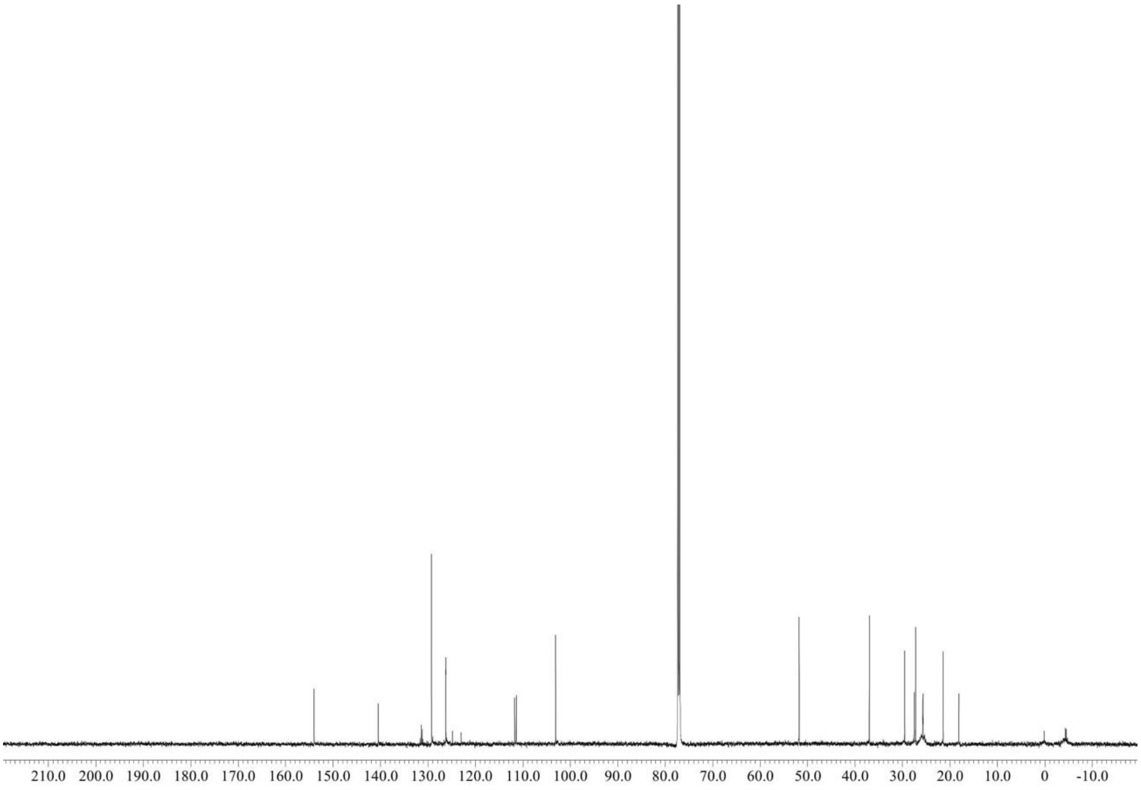

Supplementary Figure 19. ^{13}C NMR spectra of **3b** (major diastereomer).

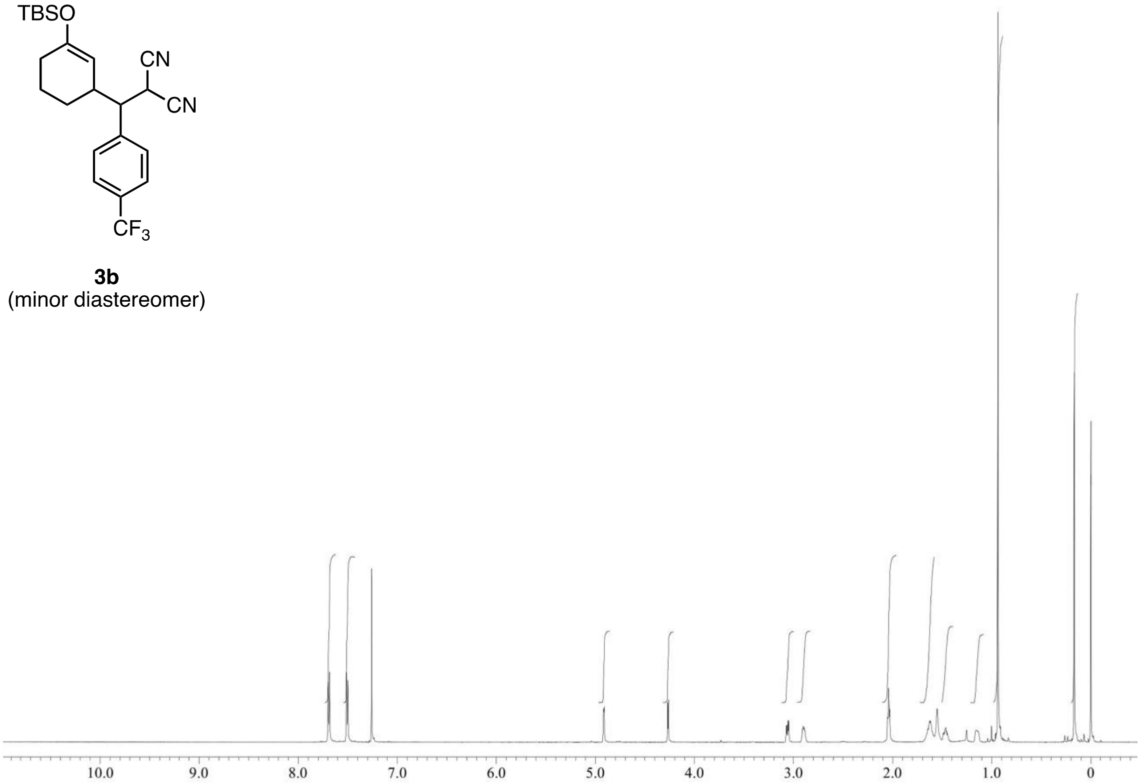

Supplementary Figure 20. ^{1}H NMR spectra of **3b** (minor diastereomer).

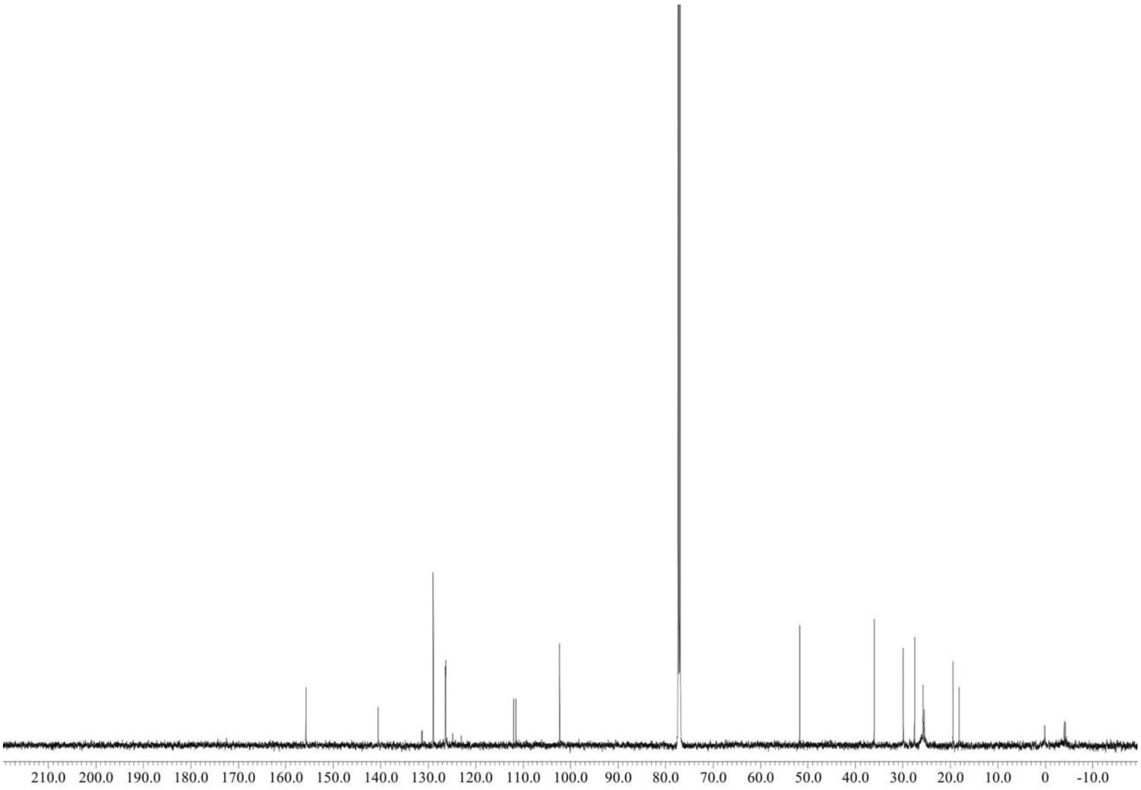

Supplementary Figure 21. ^{13}C NMR spectra of **3b** (minor diastereomer).

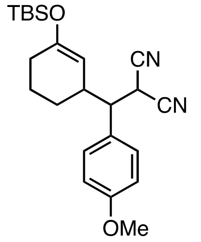

3c
(major diastereomer)

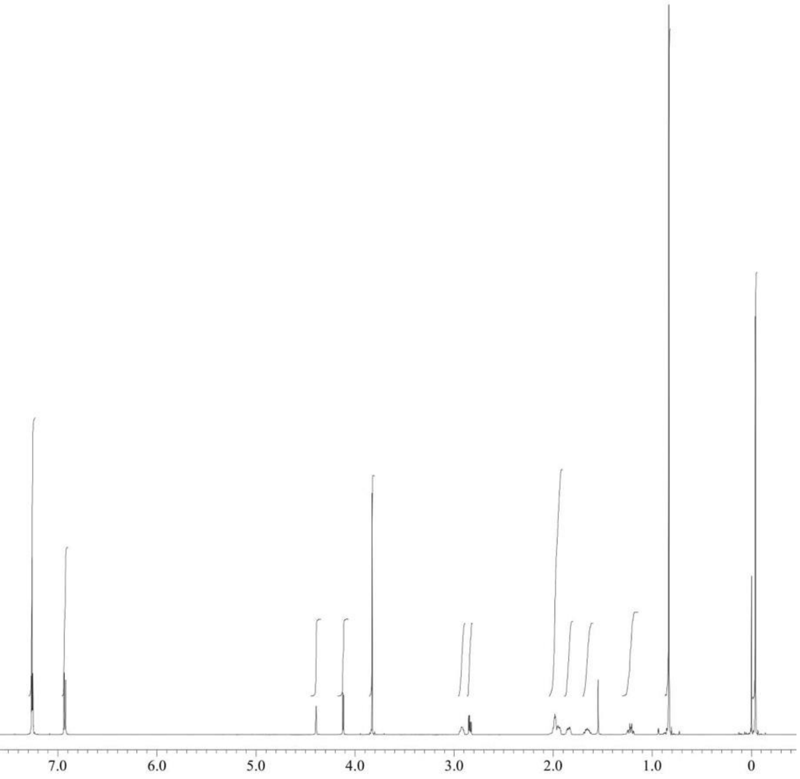

Supplementary Figure 22. ^{1}H NMR spectra of **3c** (major diastereomer).

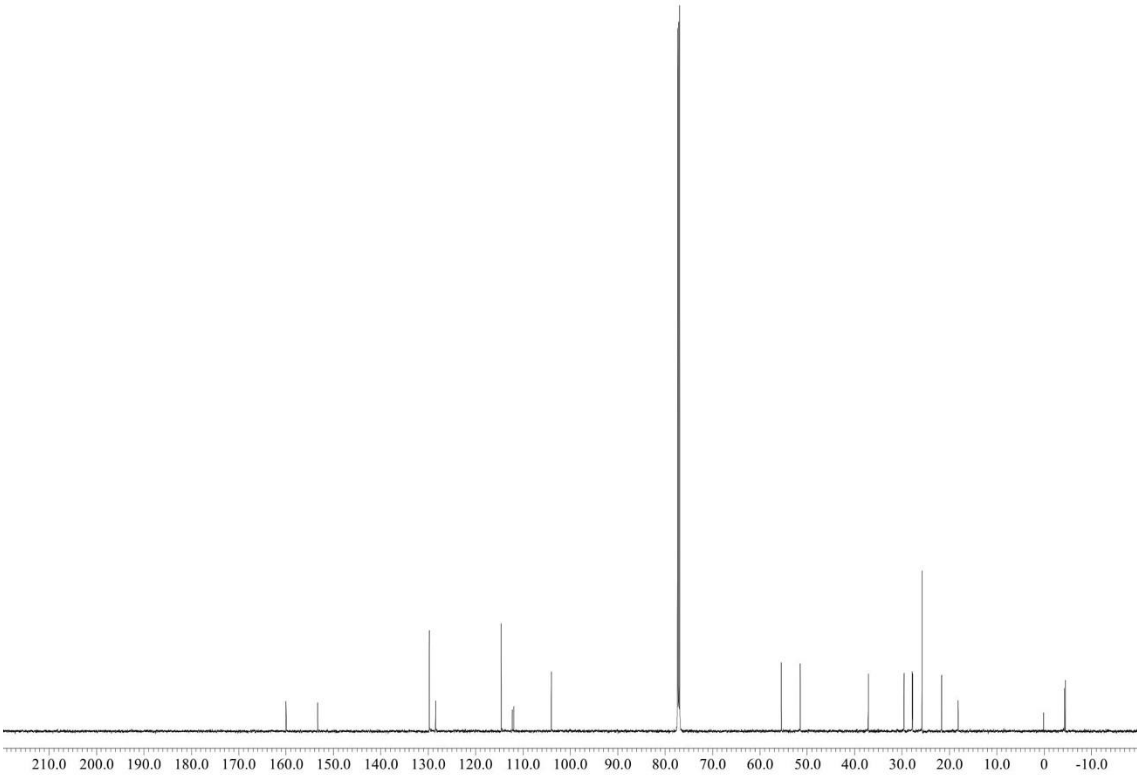

Supplementary Figure 23. ^{13}C NMR spectra of **3c** (major diastereomer).

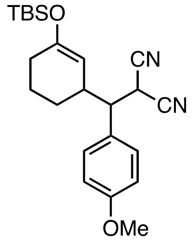

3c
(minor diastereomer)

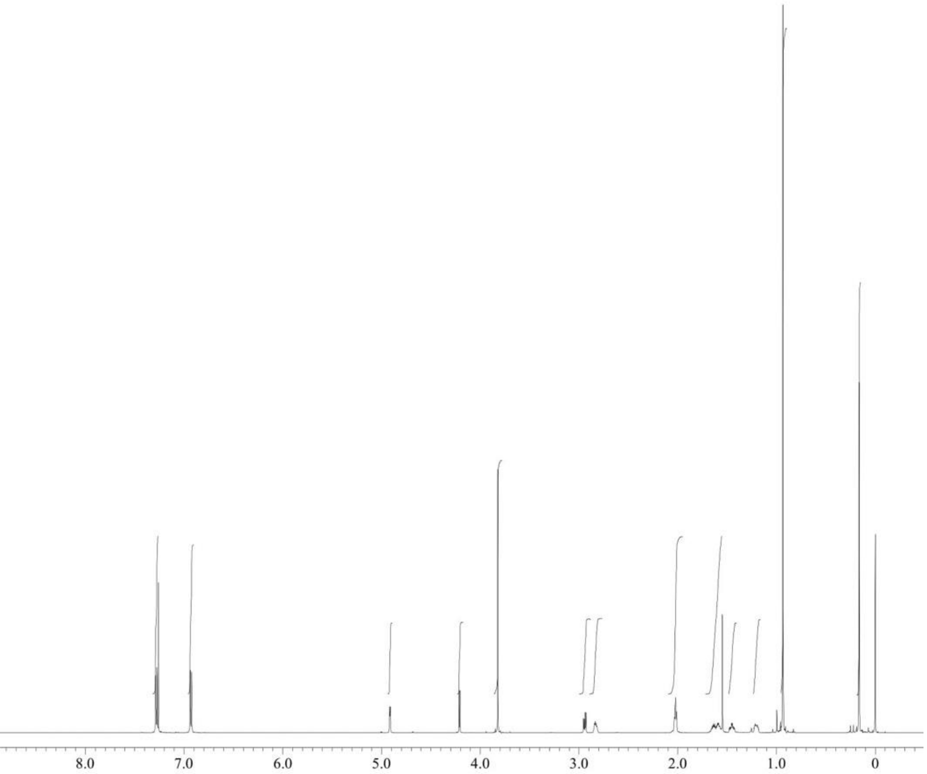

Supplementary Figure 24. ^{1}H NMR spectra of **3c** (minor diastereomer).

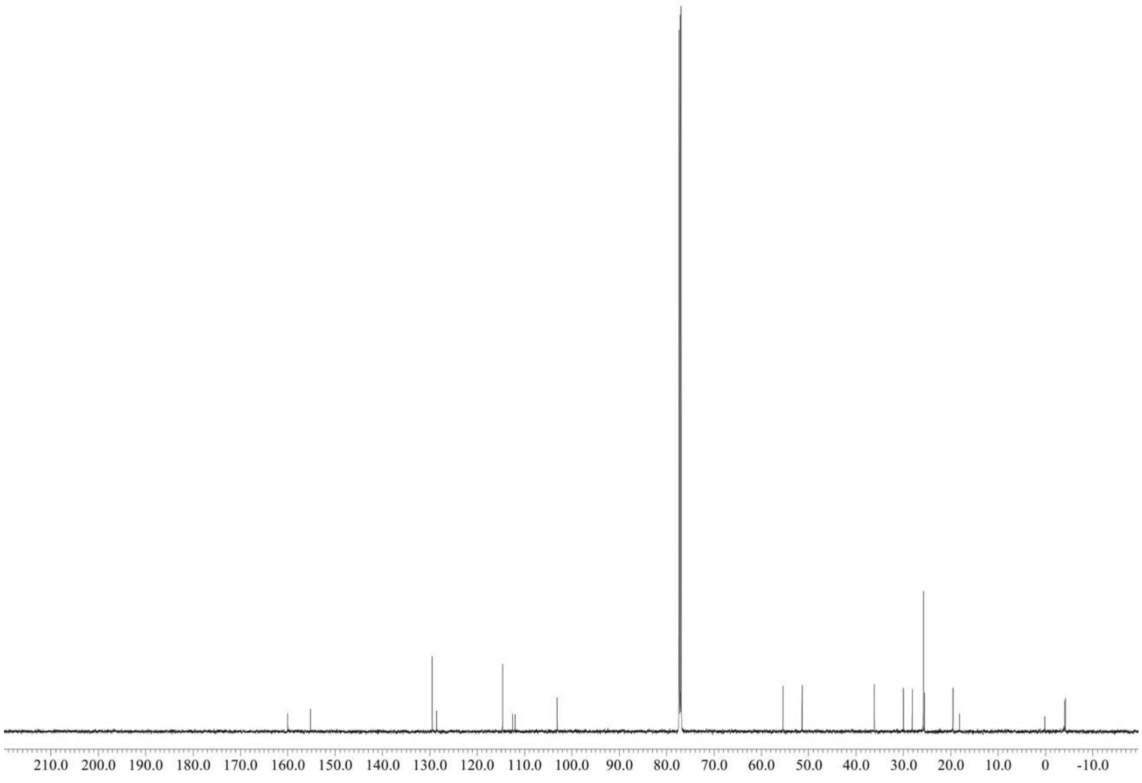

Supplementary Figure 25. ^{13}C NMR spectra of **3c** (minor diastereomer).

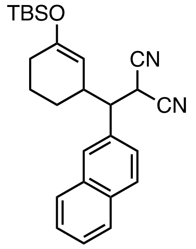

3d
(major diastereomer)

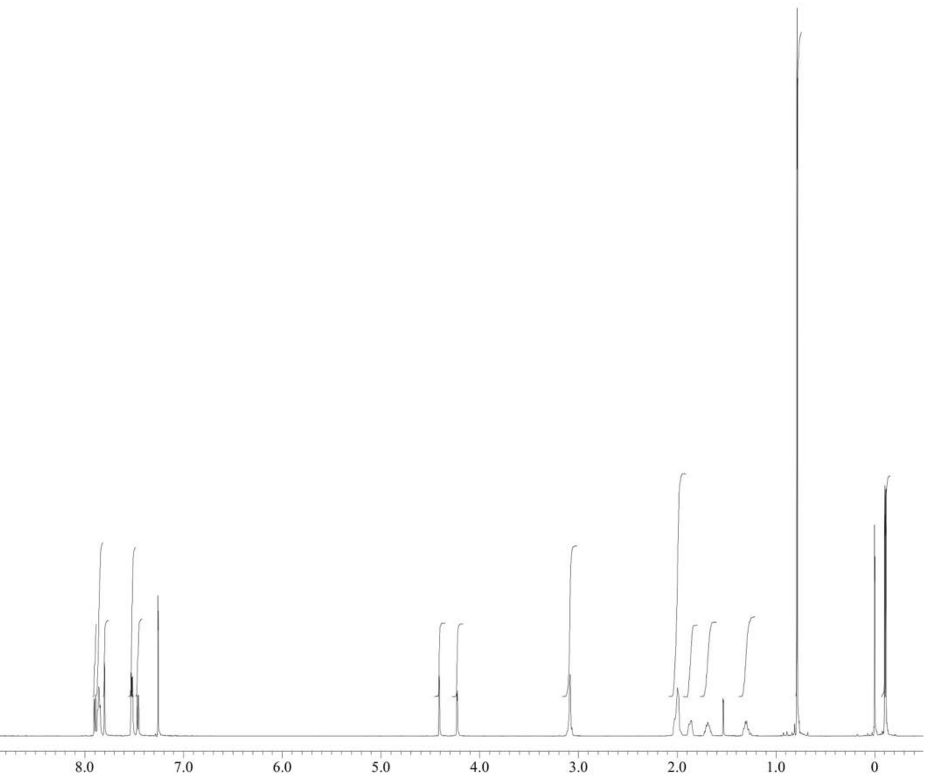

Supplementary Figure 26. ^{1}H NMR spectra of **3d** (major diastereomer).

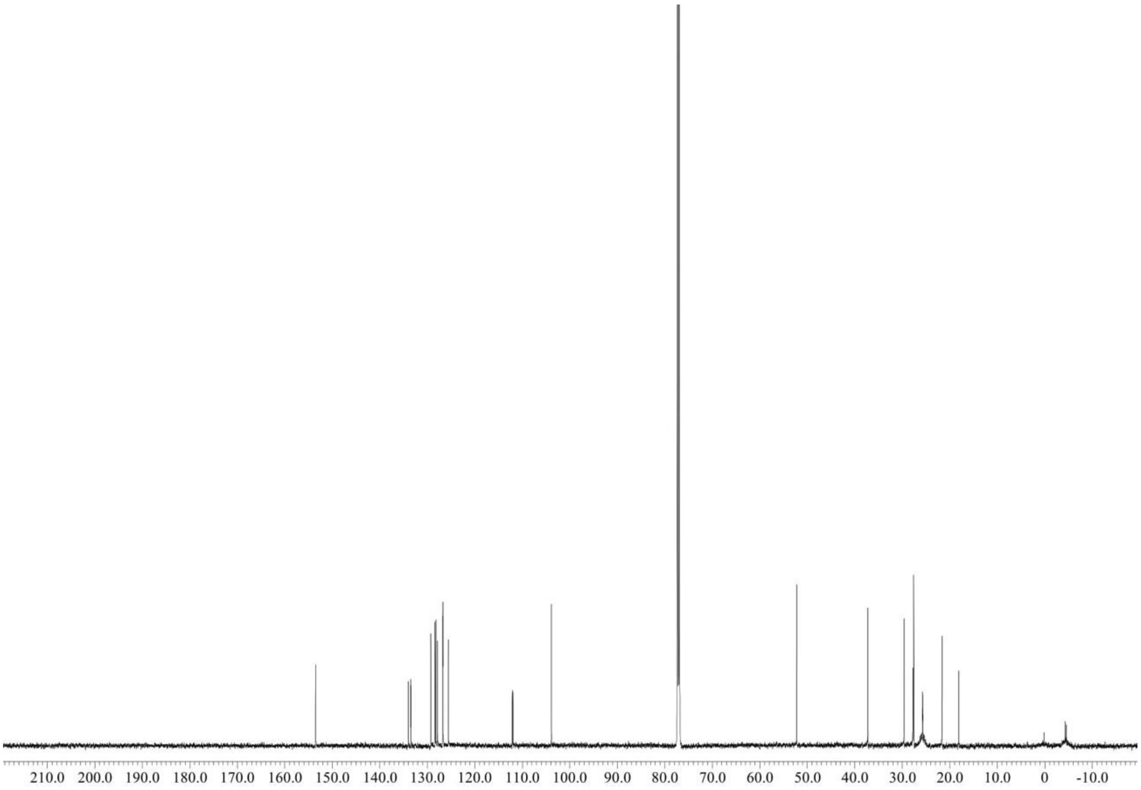

Supplementary Figure 27. ^{13}C NMR spectra of **3d** (major diastereomer).

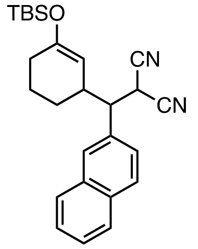

3d
(minor diastereomer)

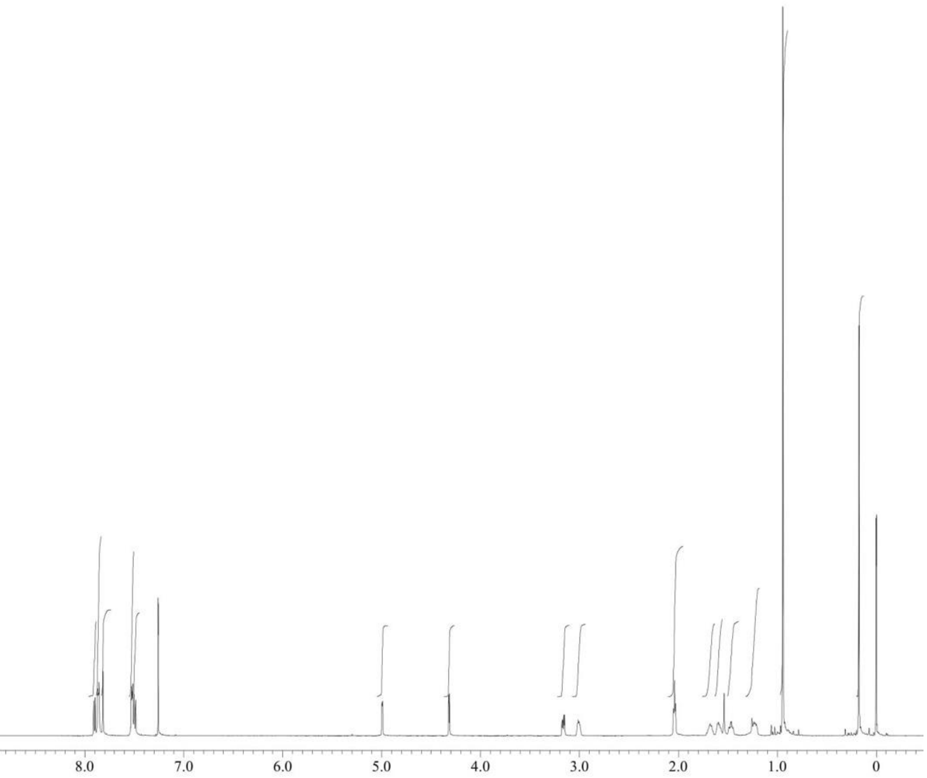

Supplementary Figure 28. ^{1}H NMR spectra of **3d** (minor diastereomer).

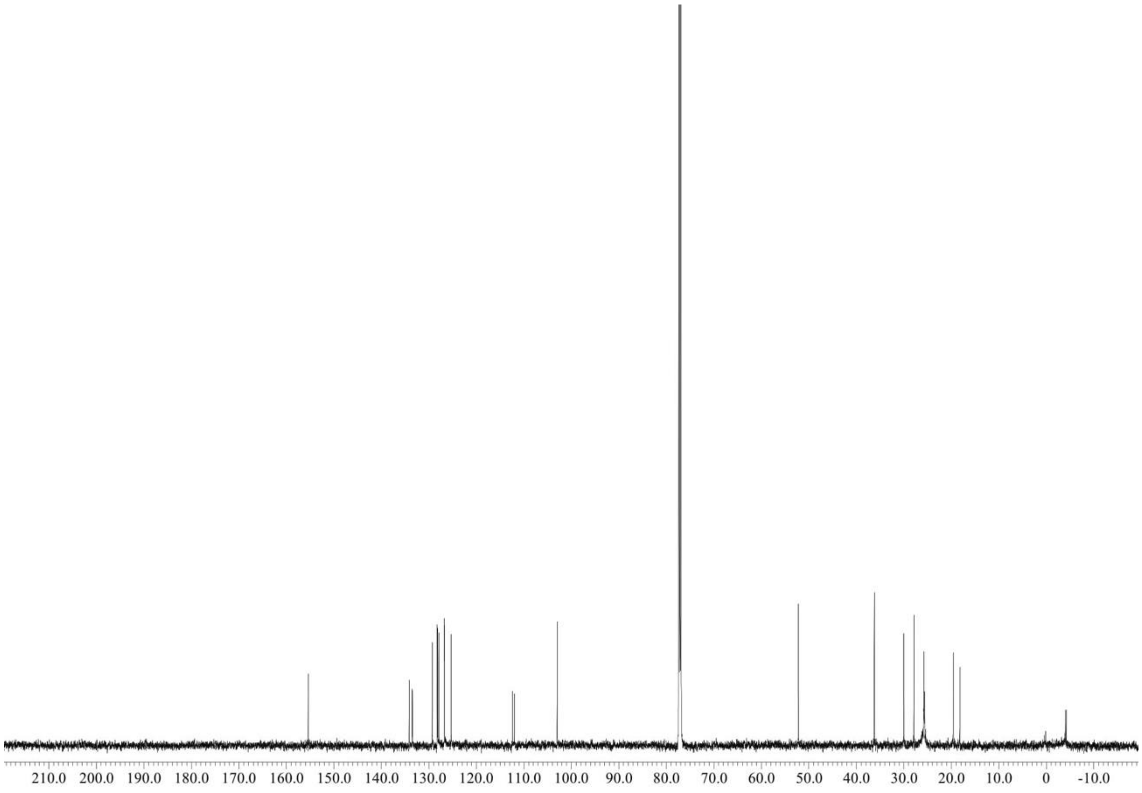

Supplementary Figure 29. ^{13}C NMR spectra of **3d** (minor diastereomer).

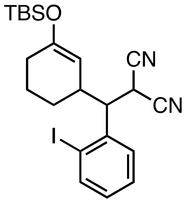

3e
(mixture of diastereomers)

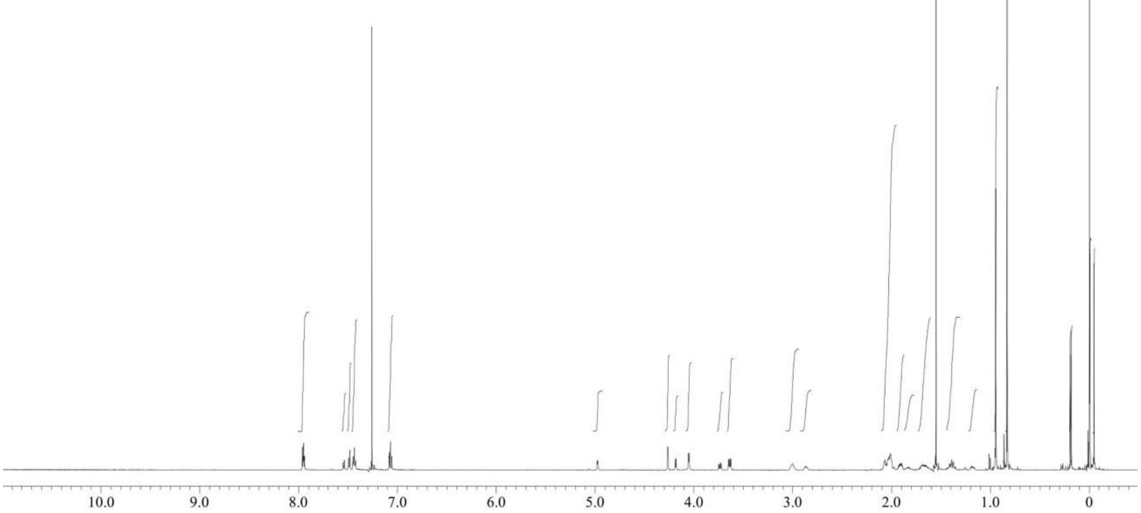

Supplementary Figure 30. ^{1}H NMR spectra of **3e** (mixture of diastereomers).

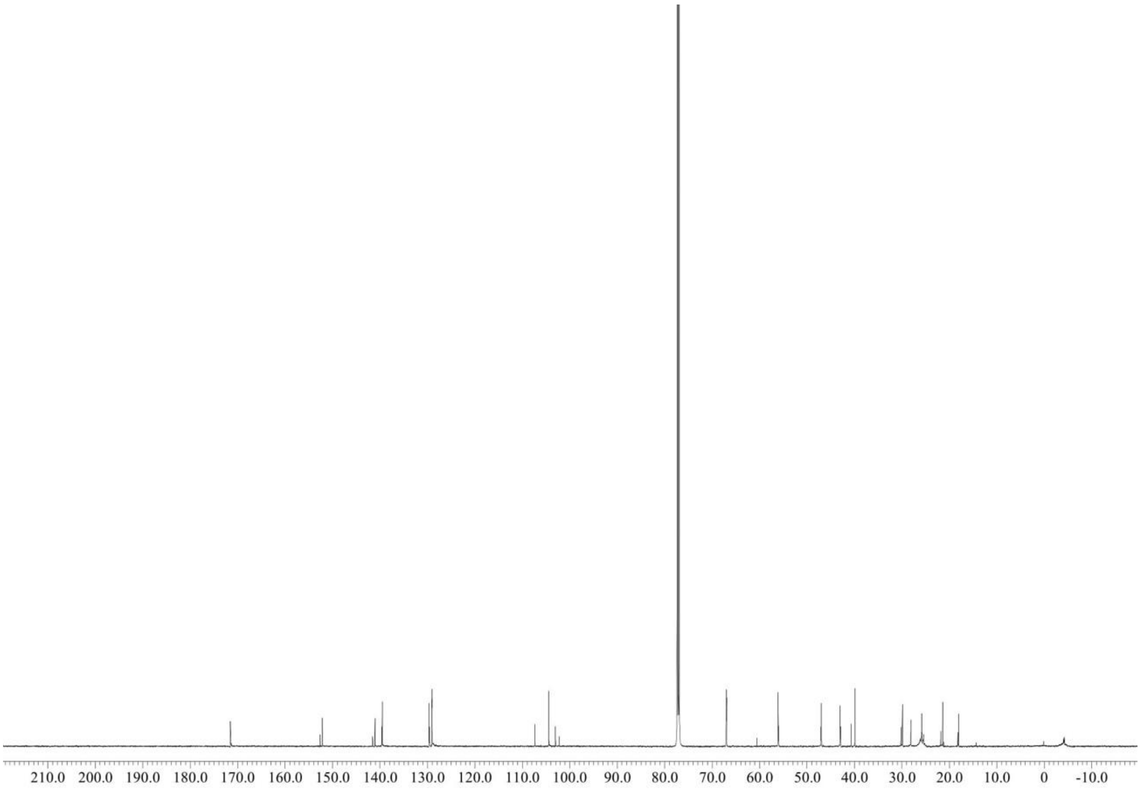

Supplementary Figure 31. ^{13}C NMR spectra of **3e** (mixture of diastereomers).

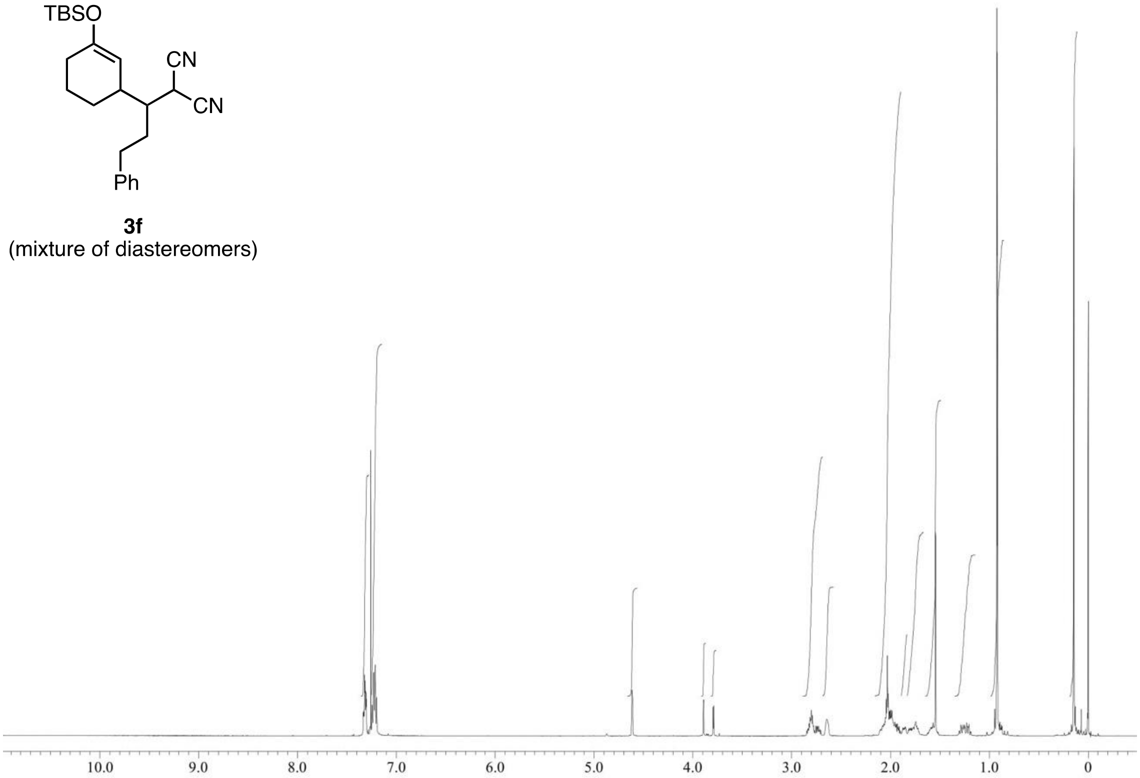

Supplementary Figure 32. ^{1}H NMR spectra of **3f** (mixture of diastereomers).

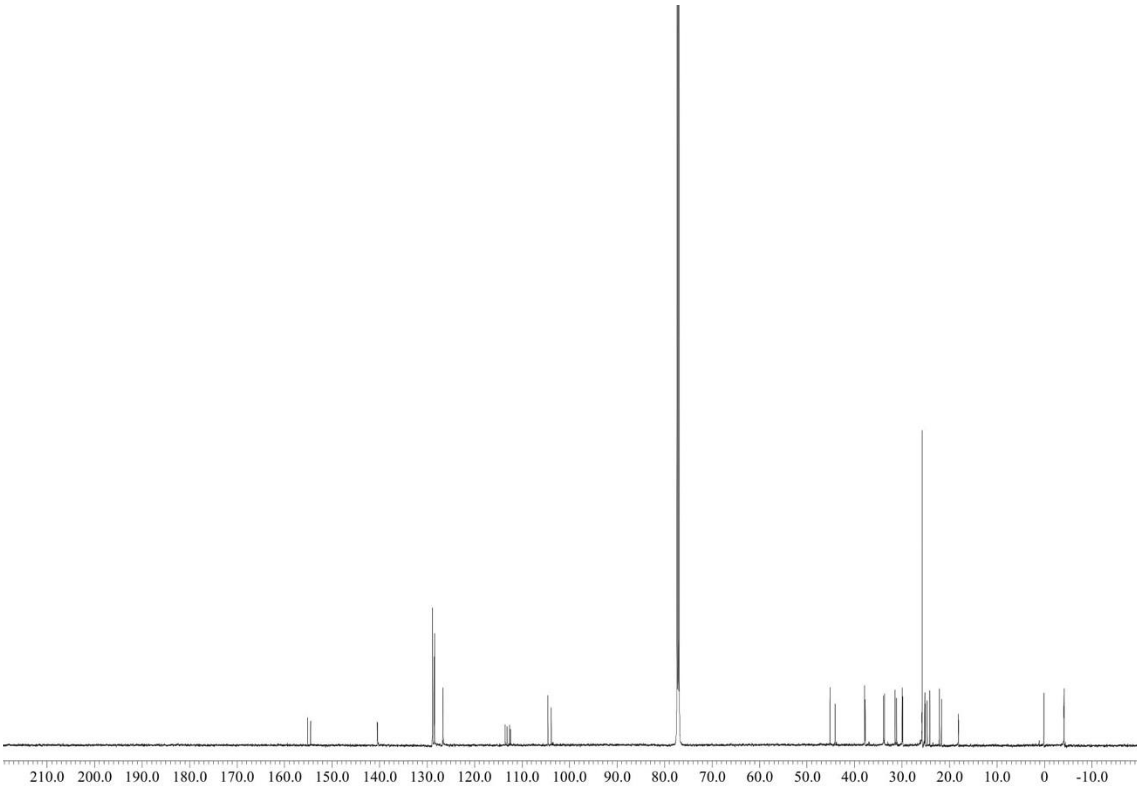

Supplementary Figure 33. ^{13}C NMR spectra of **3f** (mixture of diastereomers).

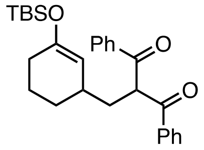

3g

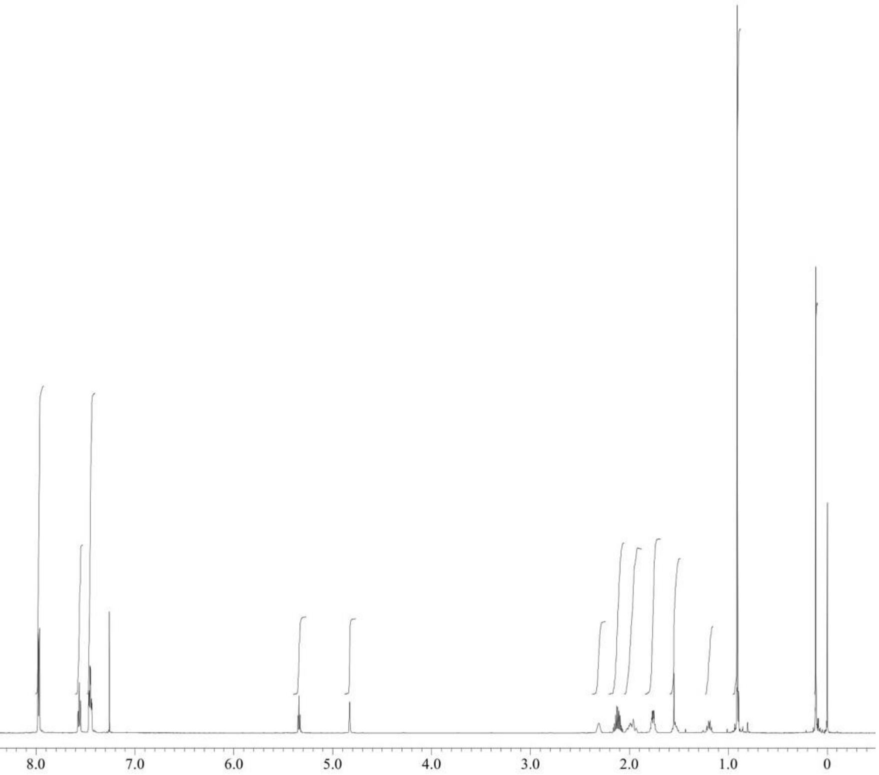

Supplementary Figure 34. ^{1}H NMR spectra of **3g**.

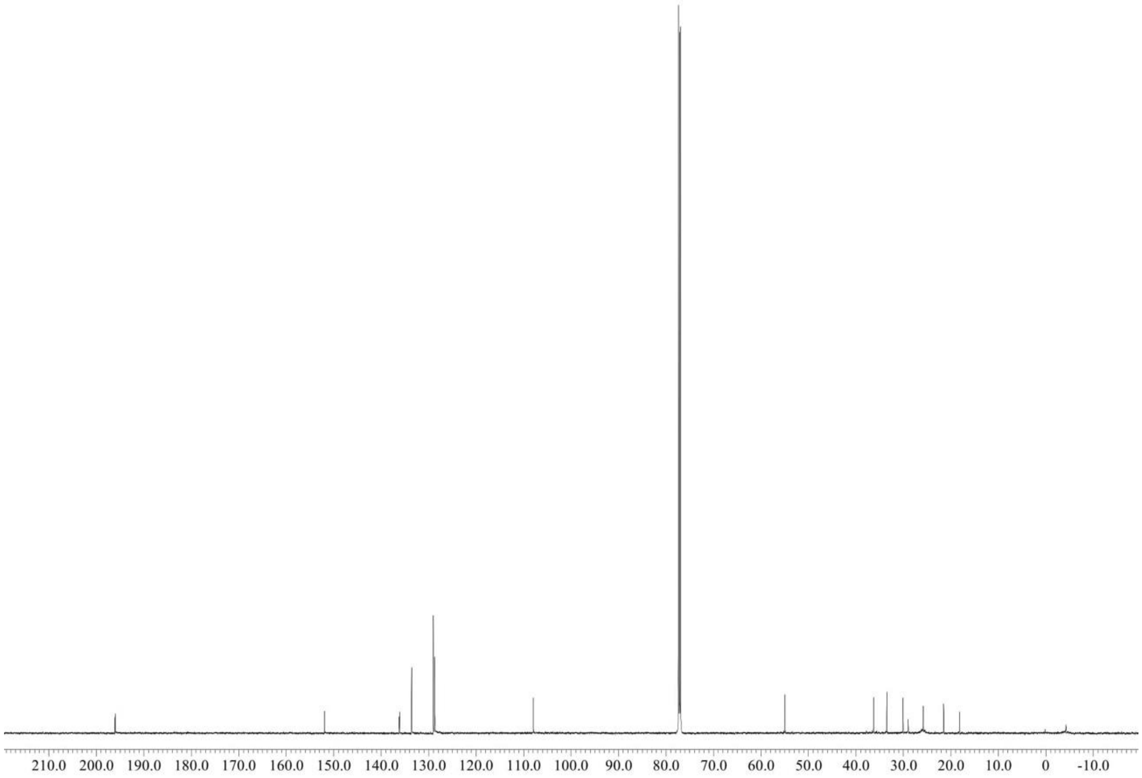

Supplementary Figure 35. ^{13}C NMR spectra of **3g**.

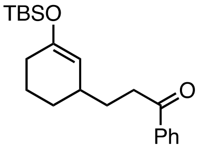

3h

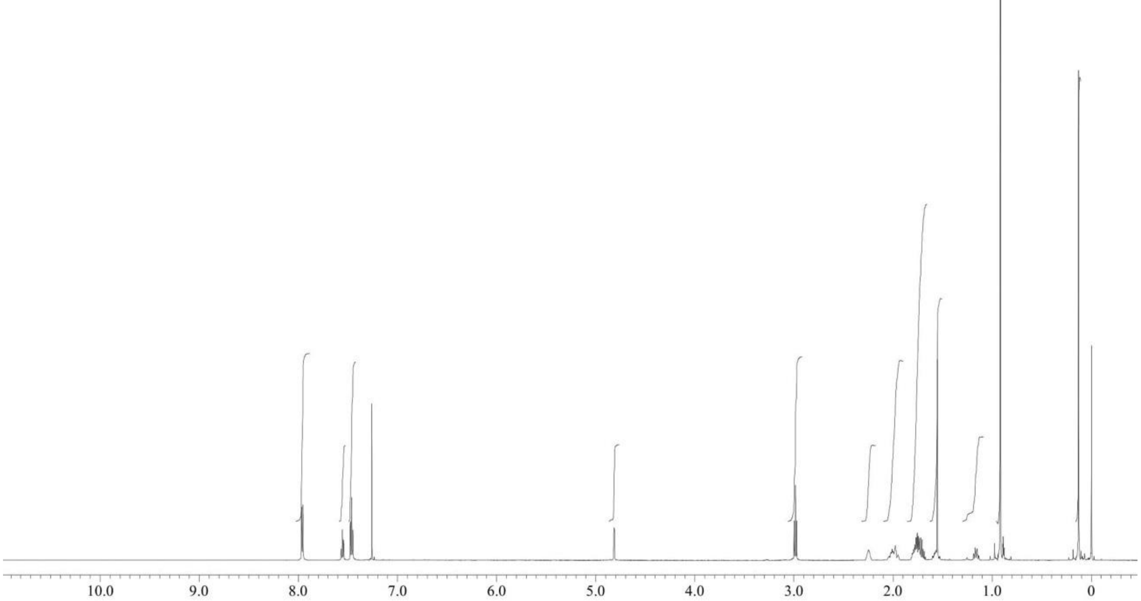

Supplementary Figure 36. ^{1}H NMR spectra of **3h**.

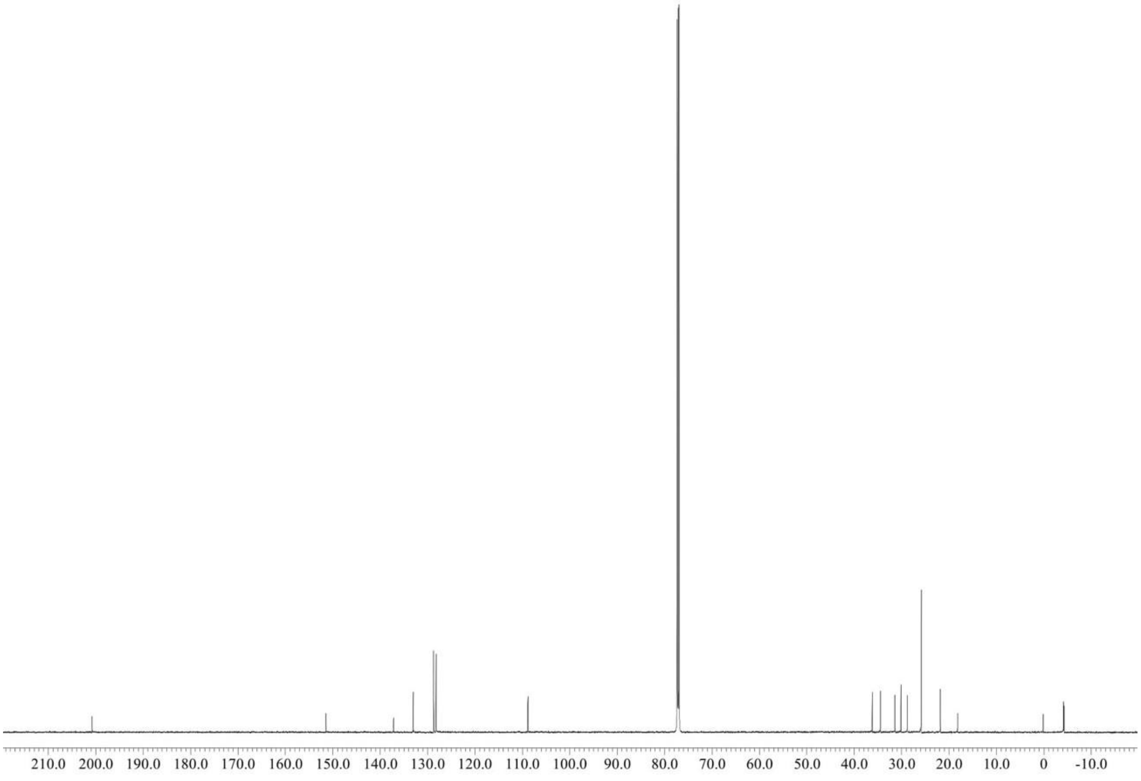

Supplementary Figure 37. ^{13}C NMR spectra of **3h**.

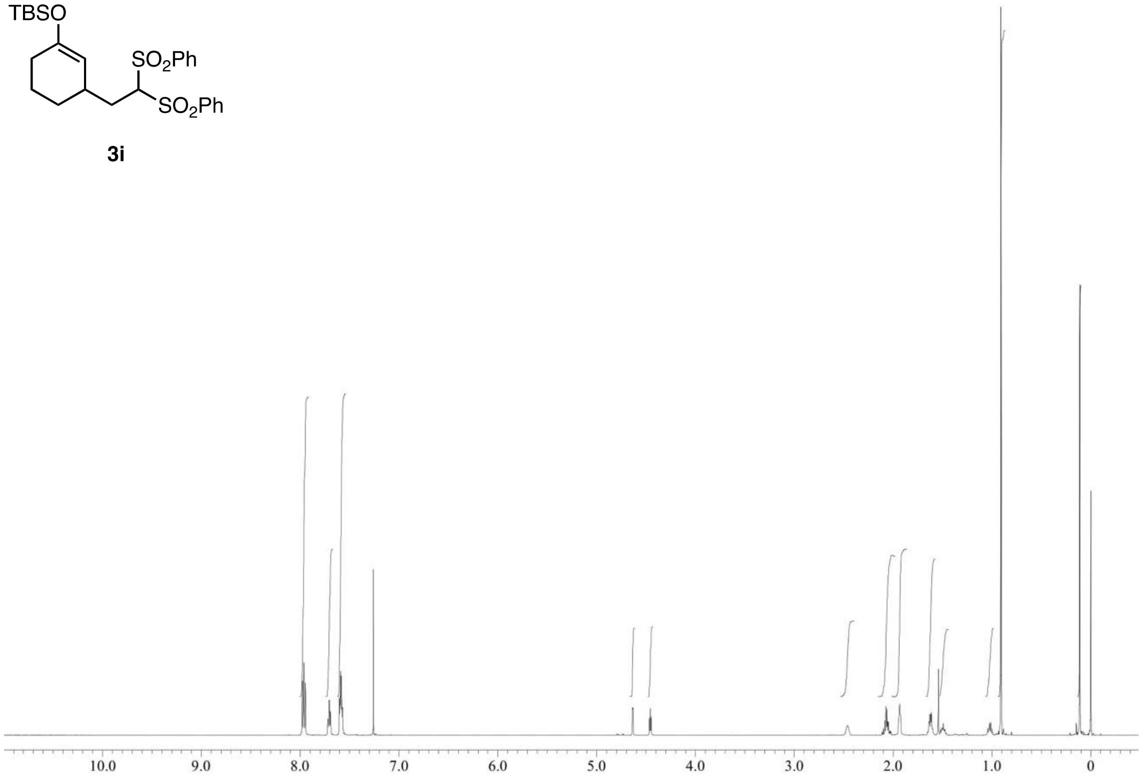

Supplementary Figure 38. ^{1}H NMR spectra of **3i**.

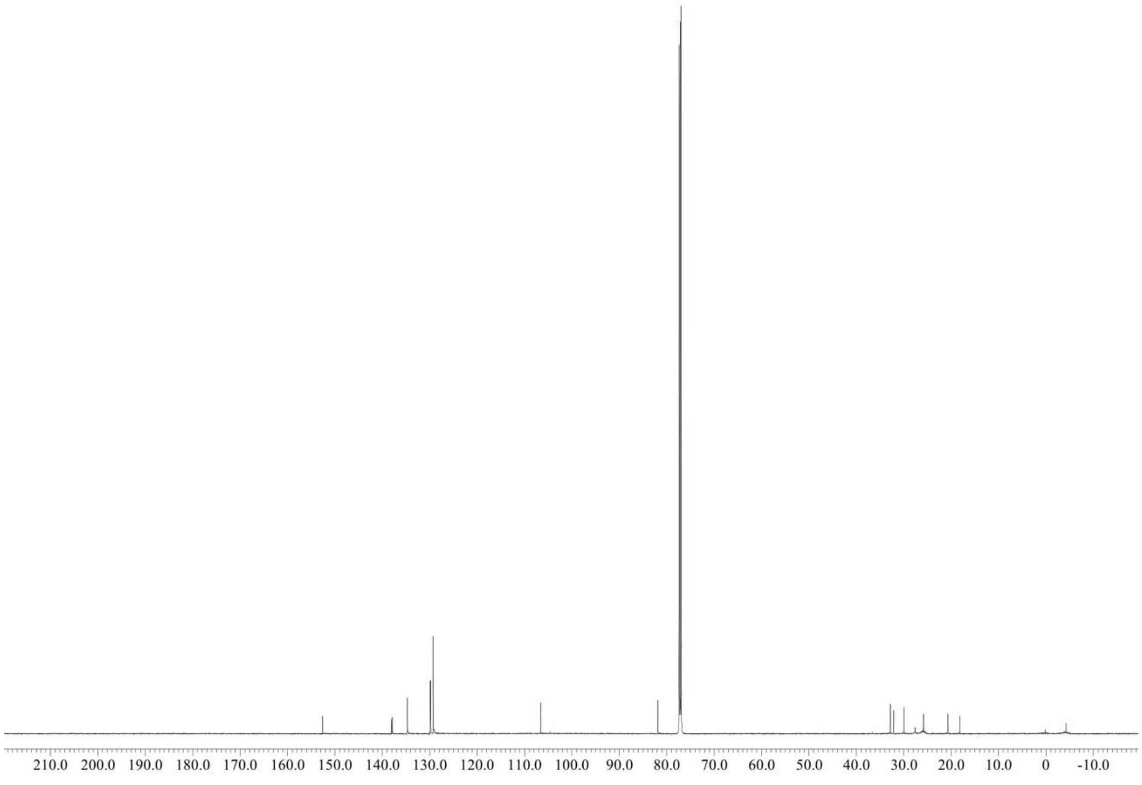

Supplementary Figure 39. ^{13}C NMR spectra of **3i**.

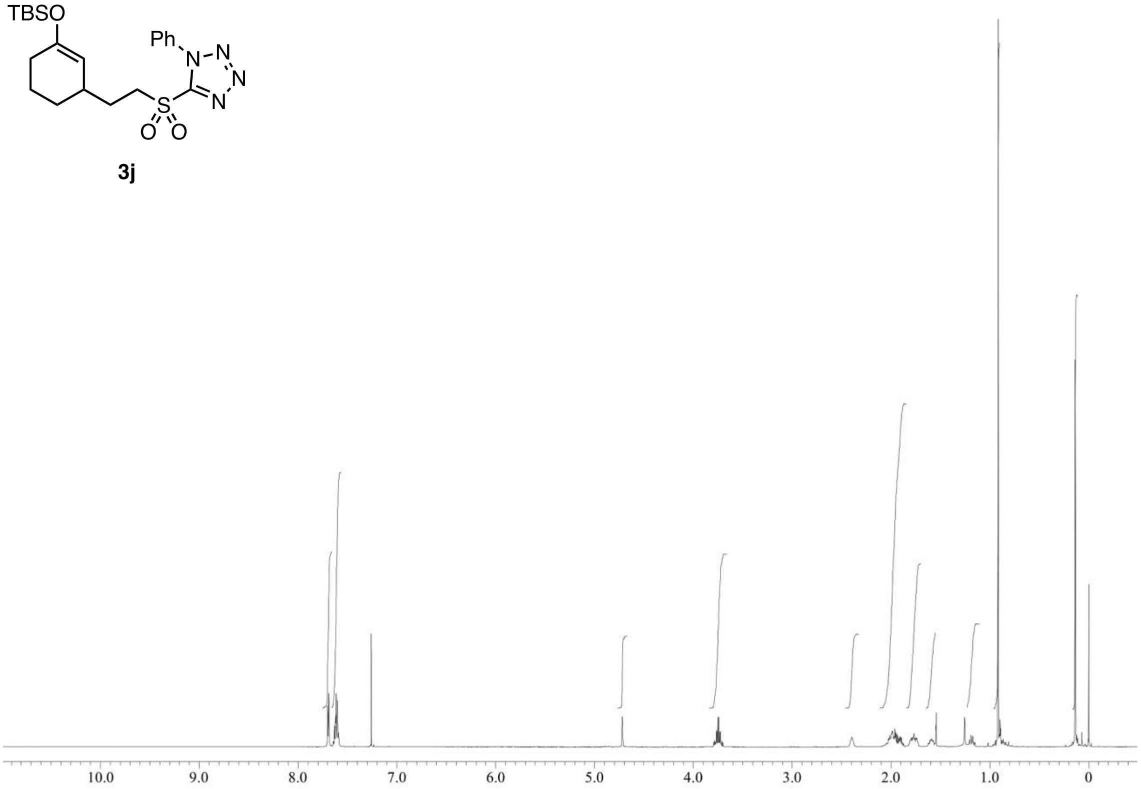

Supplementary Figure 40. ^{1}H NMR spectra of **3j**.

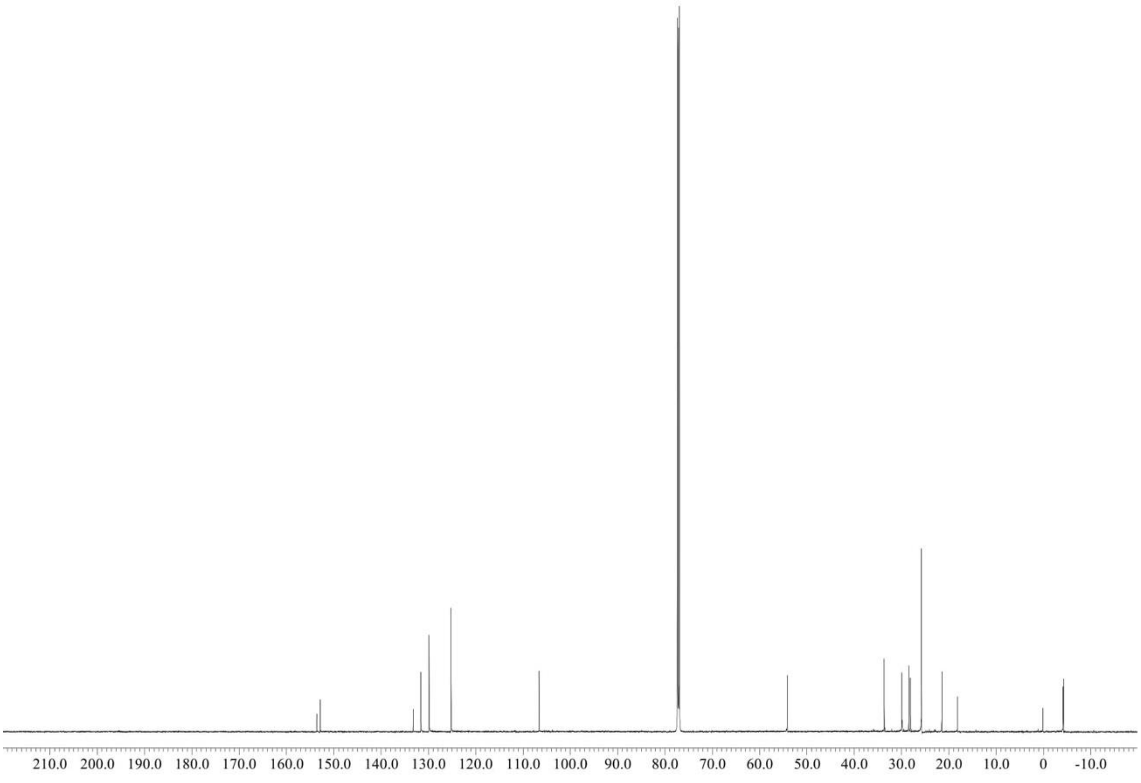

Supplementary Figure 41. ^{13}C NMR spectra of **3j**.

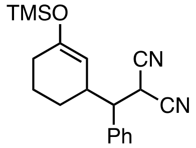

3k
(mixture of diastereomers)

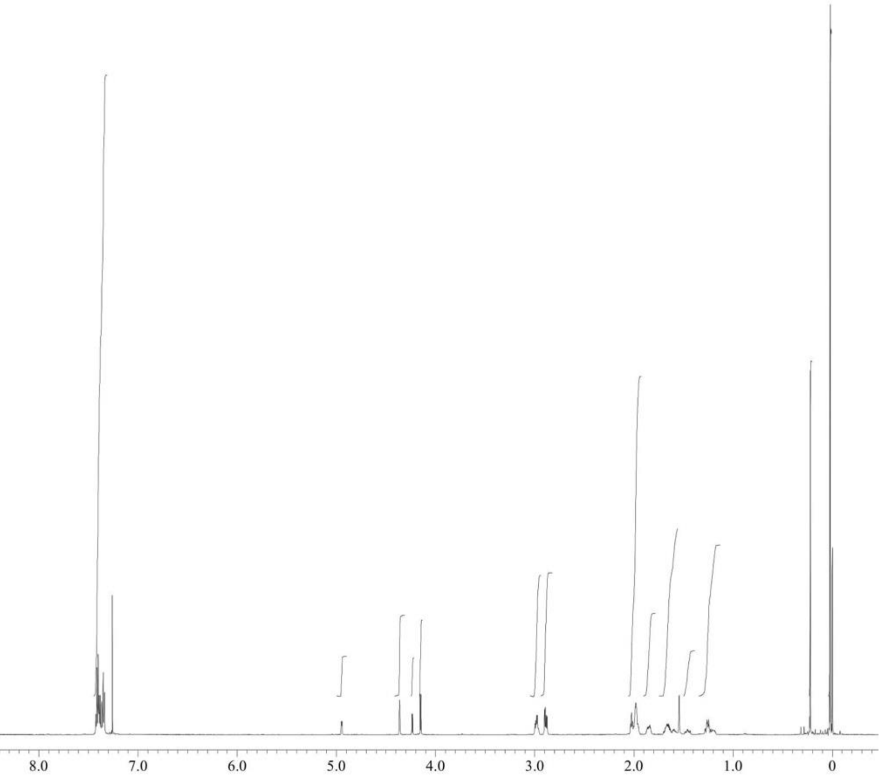

Supplementary Figure 42. ^{1}H NMR spectra of **3k** (mixture of diastereomers).

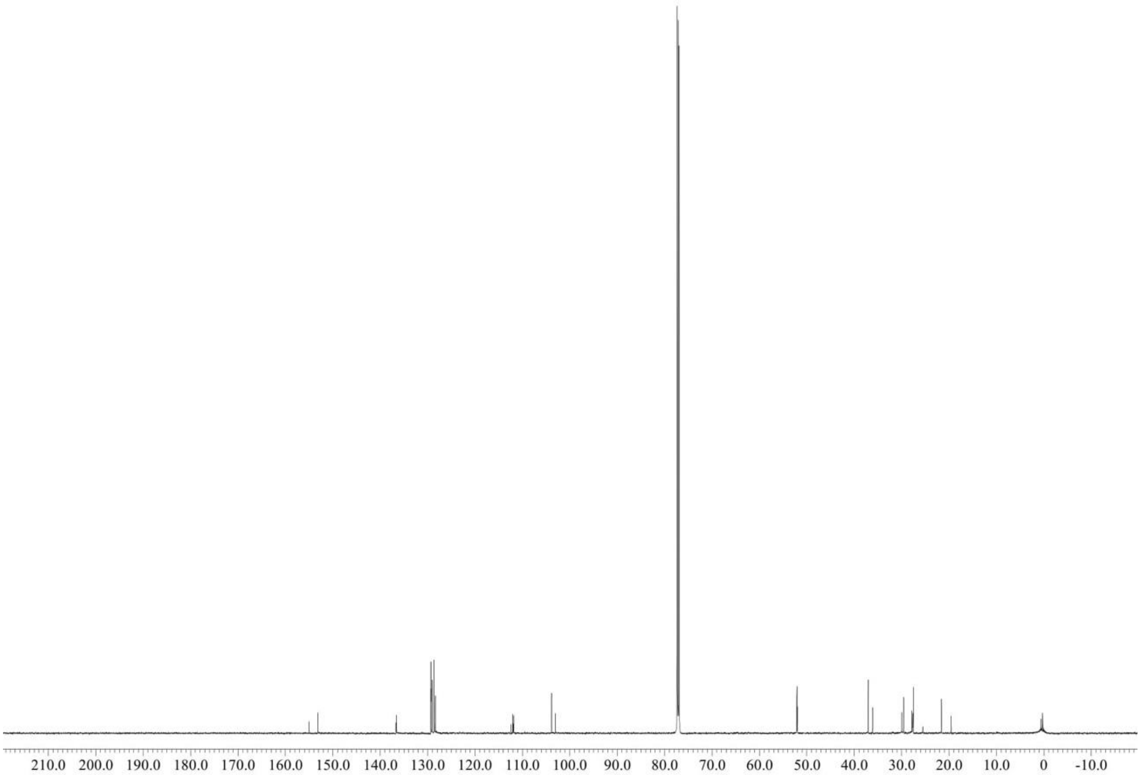

Supplementary Figure 43. ^{13}C NMR spectra of **3k** (mixture of diastereomers).

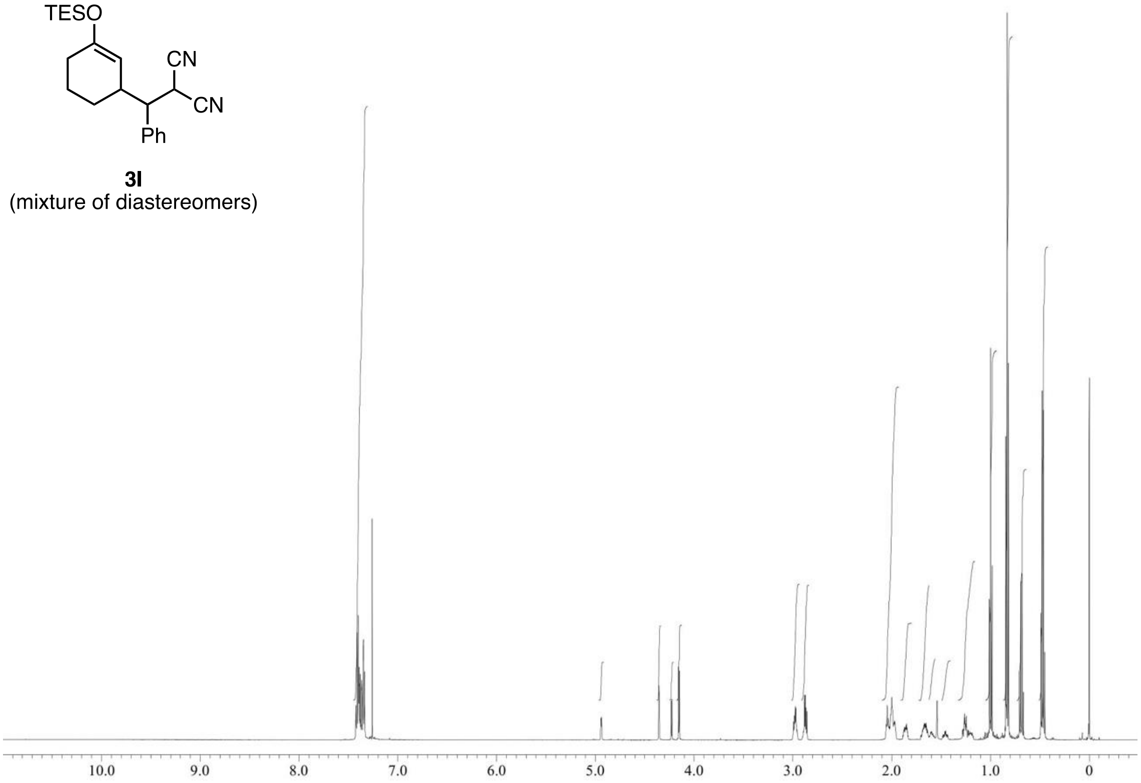

Supplementary Figure 44. ^{1}H NMR spectra of **3l** (mixture of diastereomers).

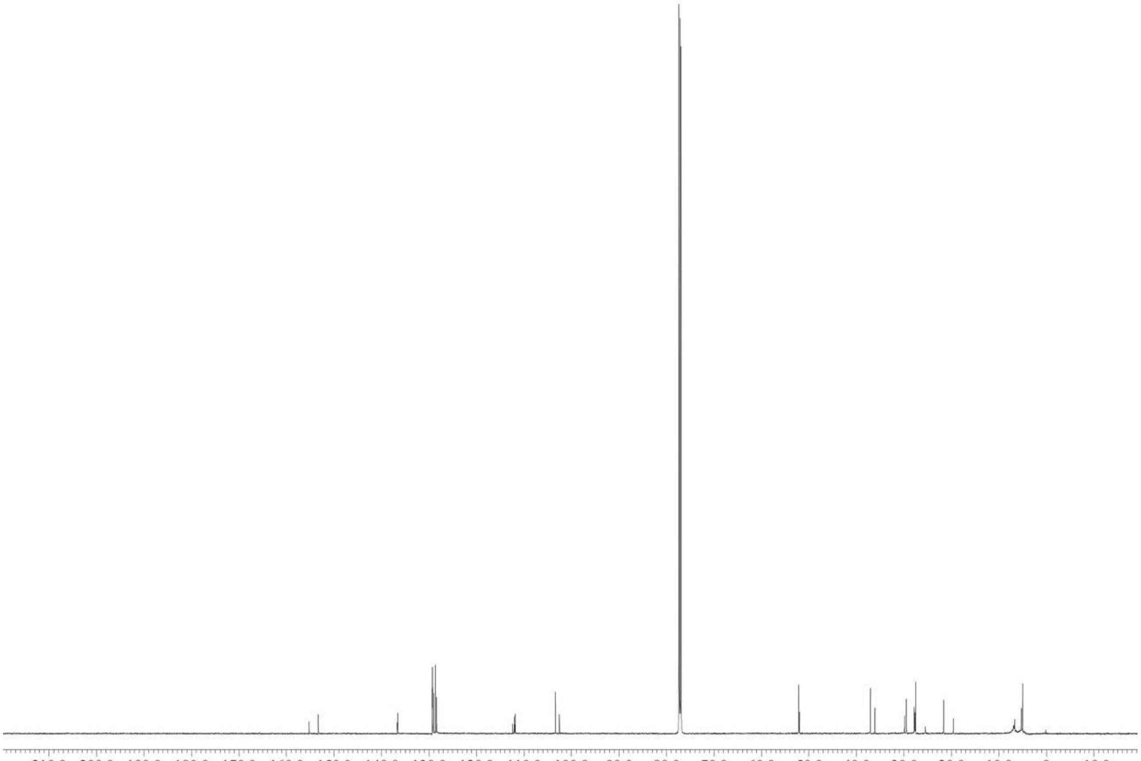

Supplementary Figure 45. ^{13}C NMR spectra of **3l** (mixture of diastereomers).

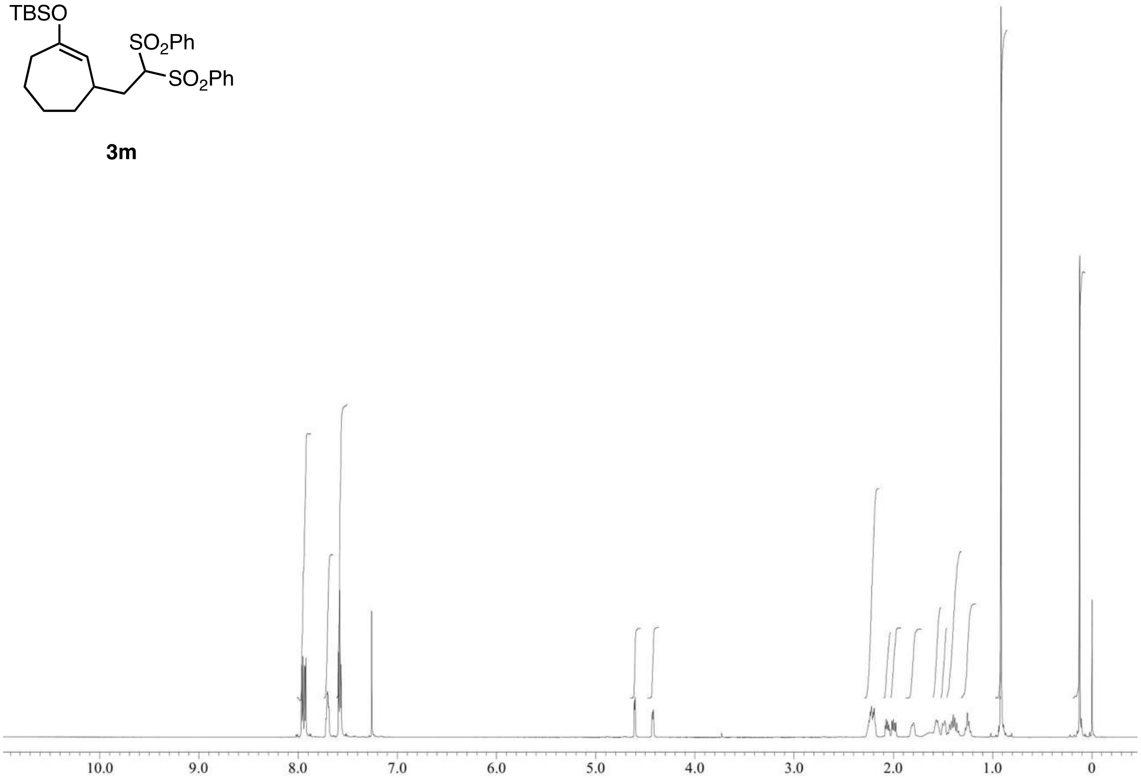

Supplementary Figure 46. ^{1}H NMR spectra of **3m**.

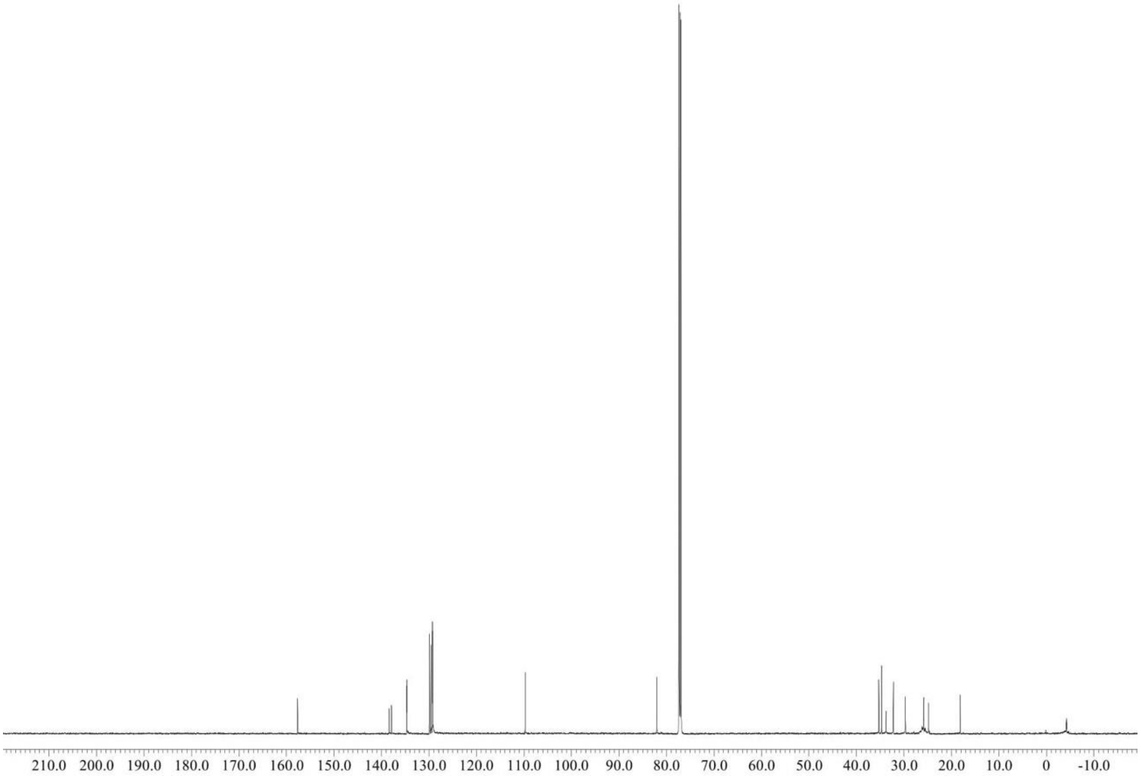

Supplementary Figure 47. ^{13}C NMR spectra of **3m**.

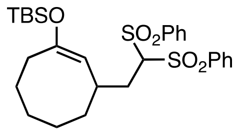

3n

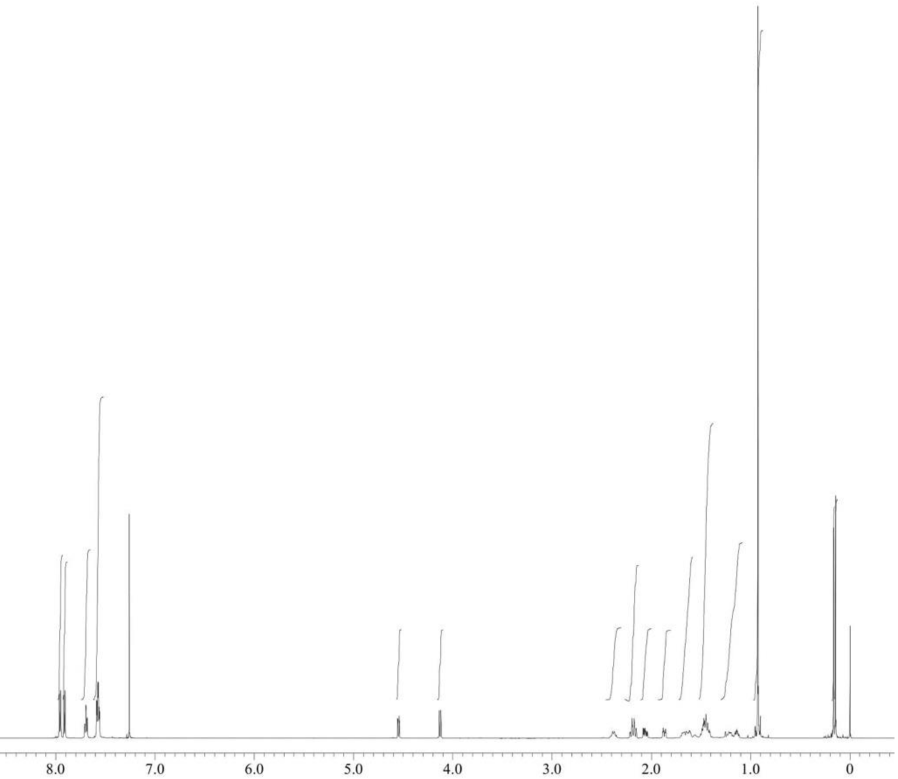

Supplementary Figure 48. ^{1}H NMR spectra of **3n**.

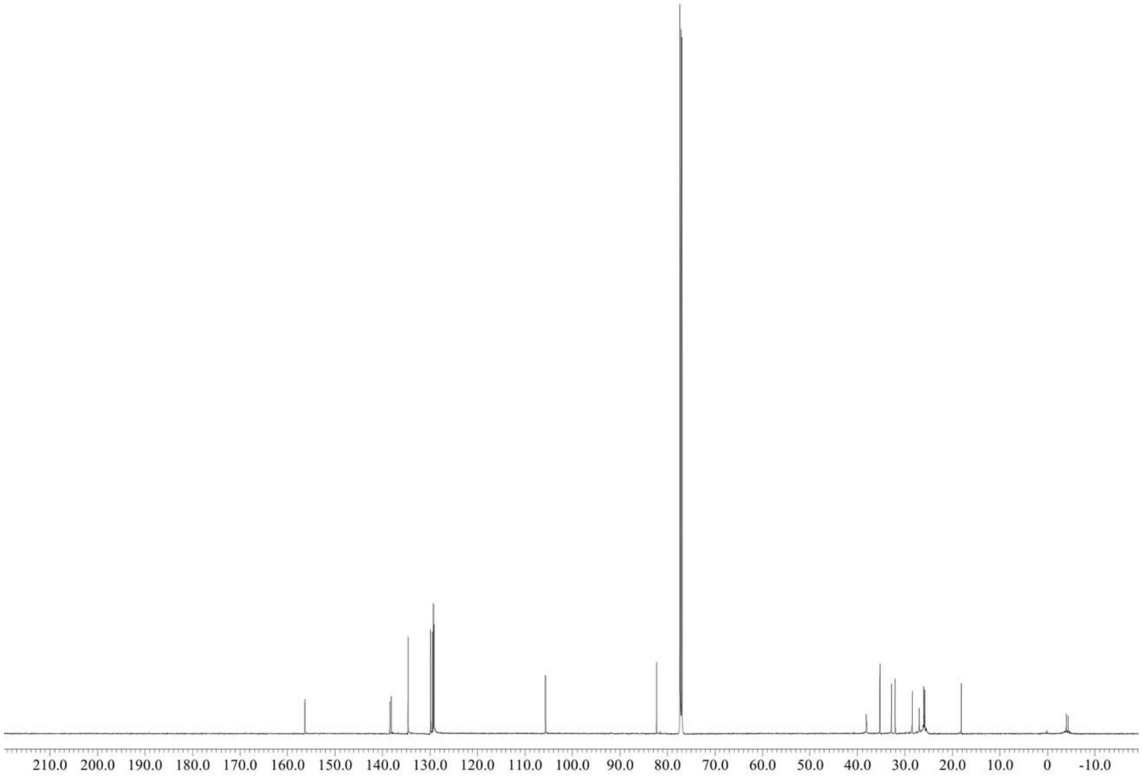

Supplementary Figure 49. ^{13}C NMR spectra of **3n**.

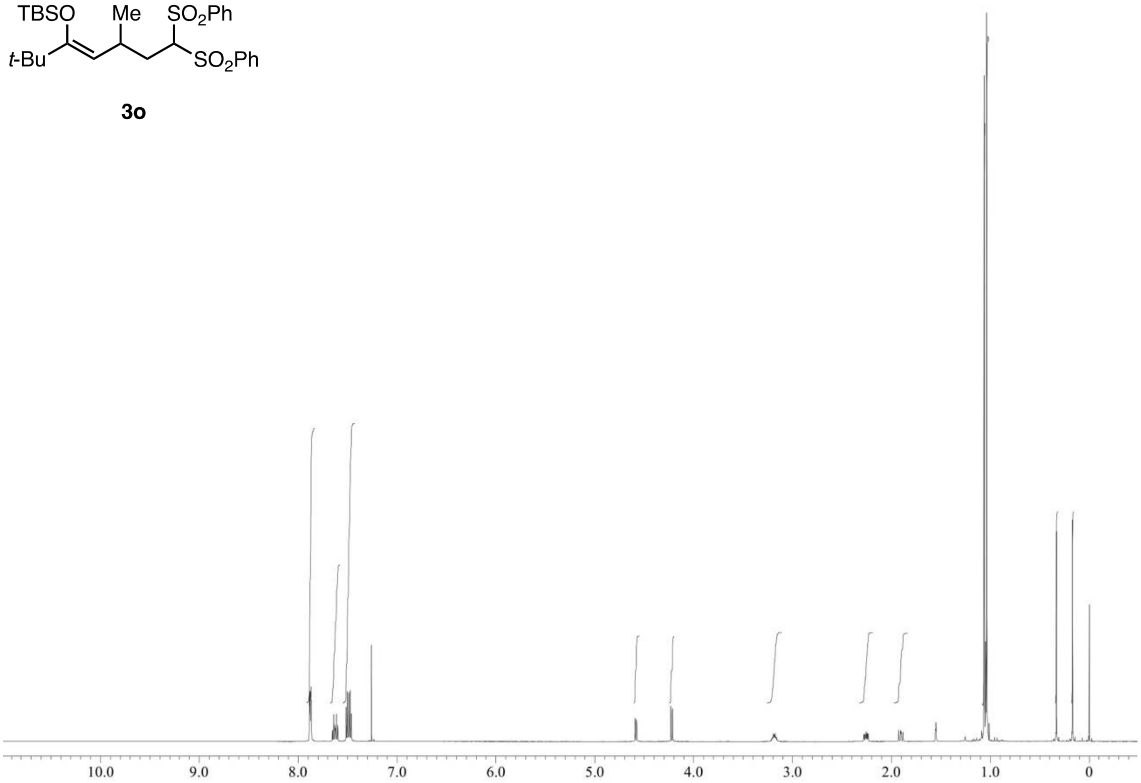

Supplementary Figure 50. ^{1}H NMR spectra of **3o**.

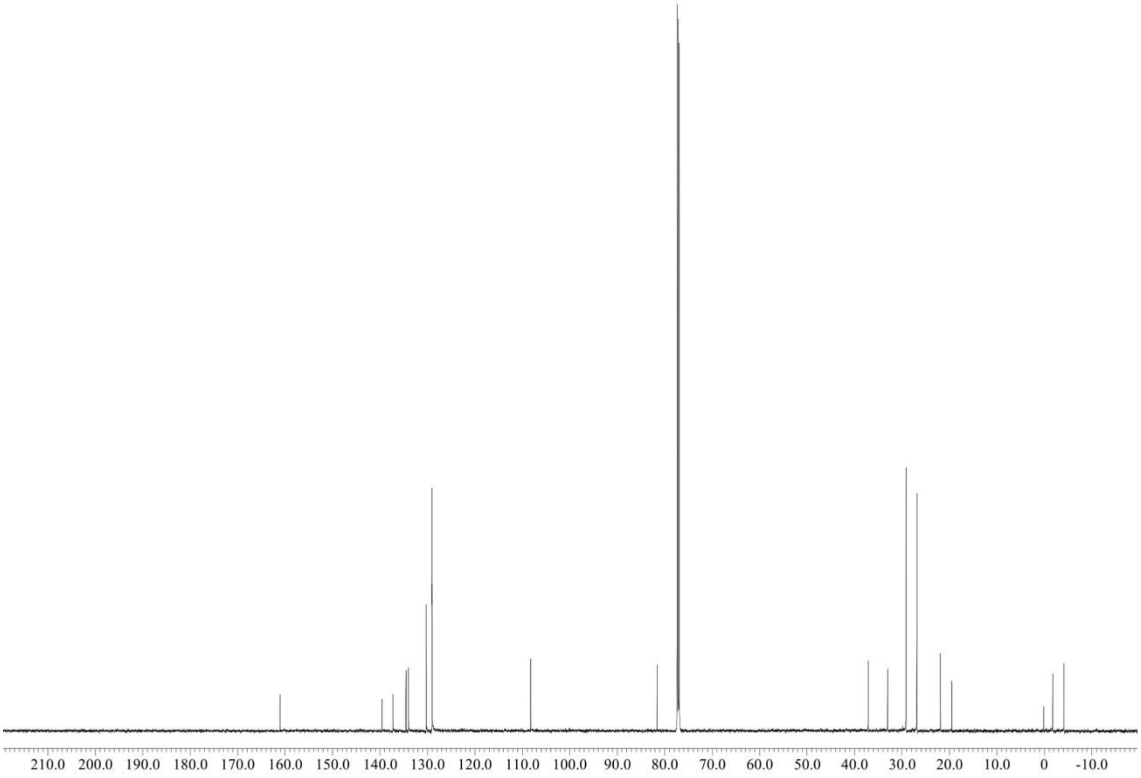

Supplementary Figure 51. ^{13}C NMR spectra of **3o**.

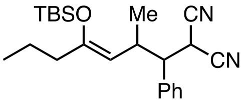

3p
(major isomer)

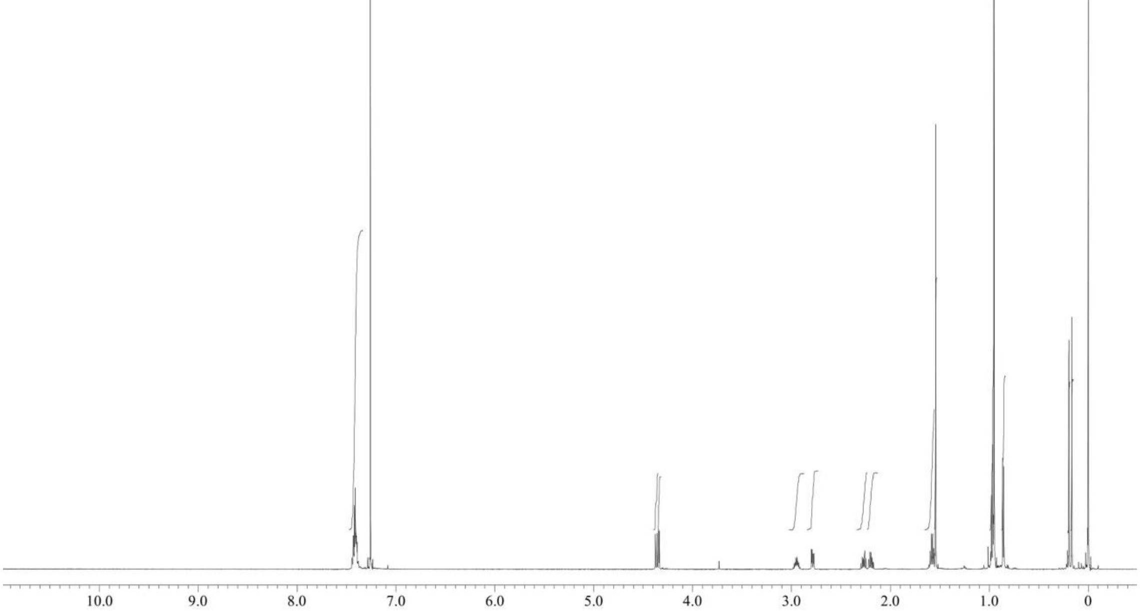

Supplementary Figure 52. ^{1}H NMR spectra of **3p** (major isomer).

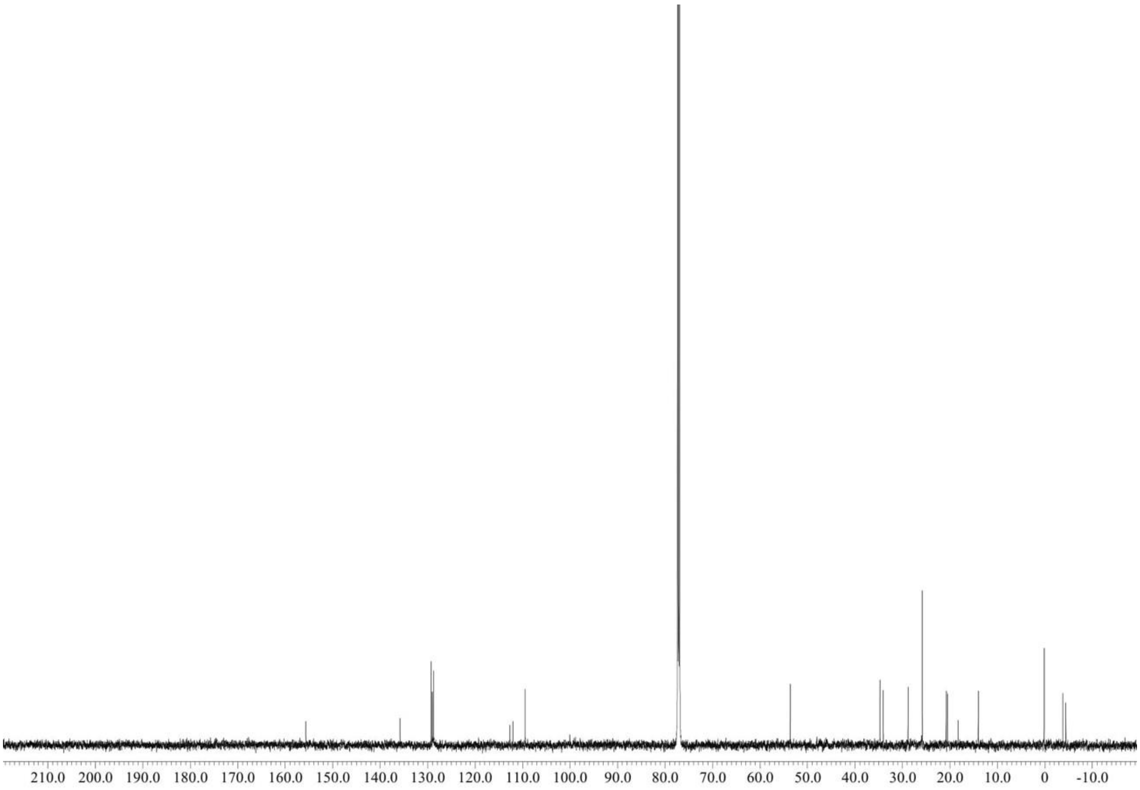

Supplementary Figure 53. ^{13}C NMR spectra of **3p** (major isomer).

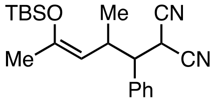

3q
(major diastereomer)

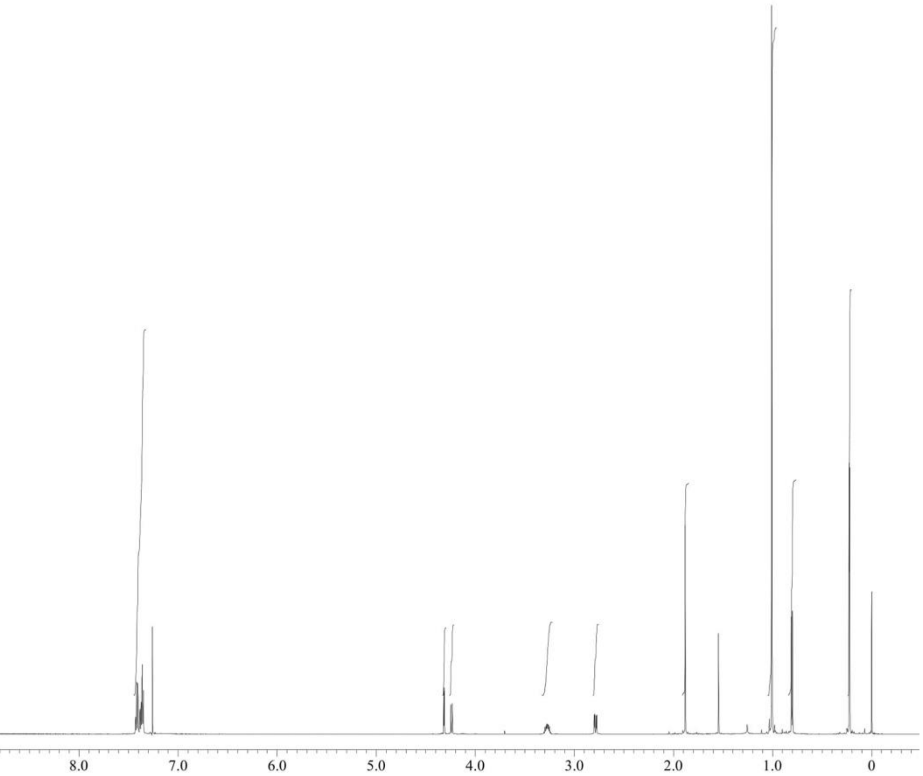

Supplementary Figure 54. ^{1}H NMR spectra of **3q** (major diastereomer).

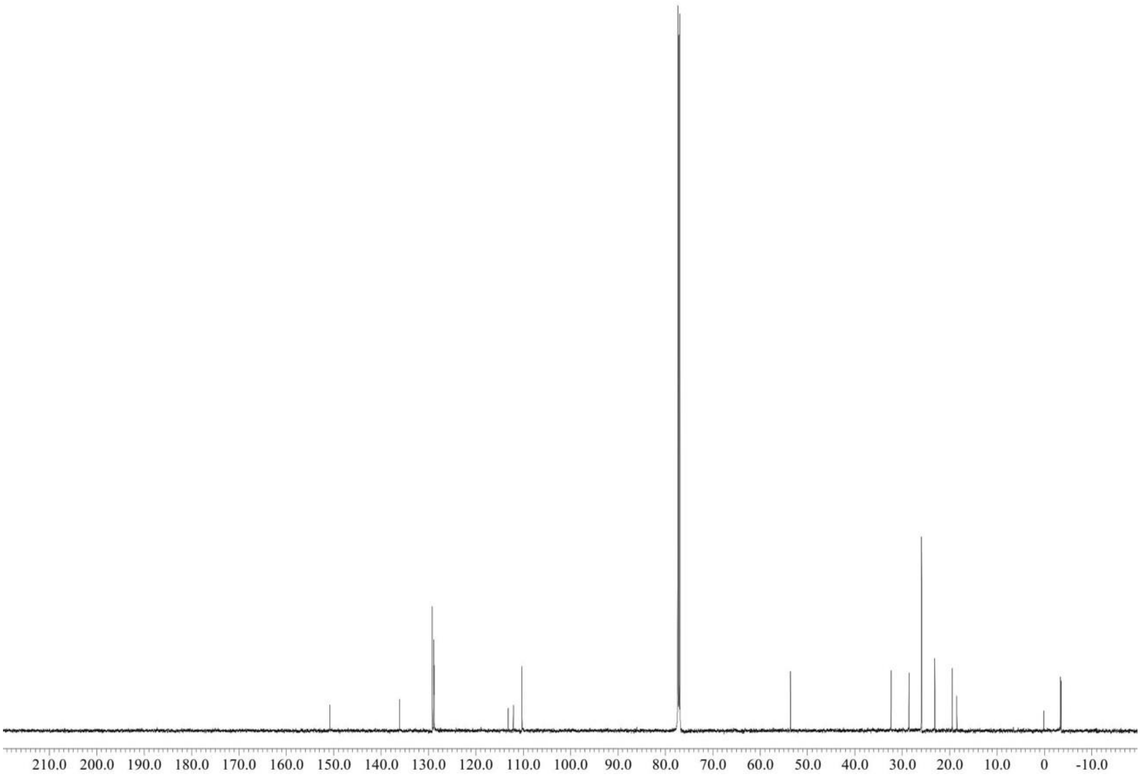

Supplementary Figure 55. ^{13}C NMR spectra of **3q** (major diastereomer).

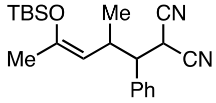

3q
(minor diastereomer)

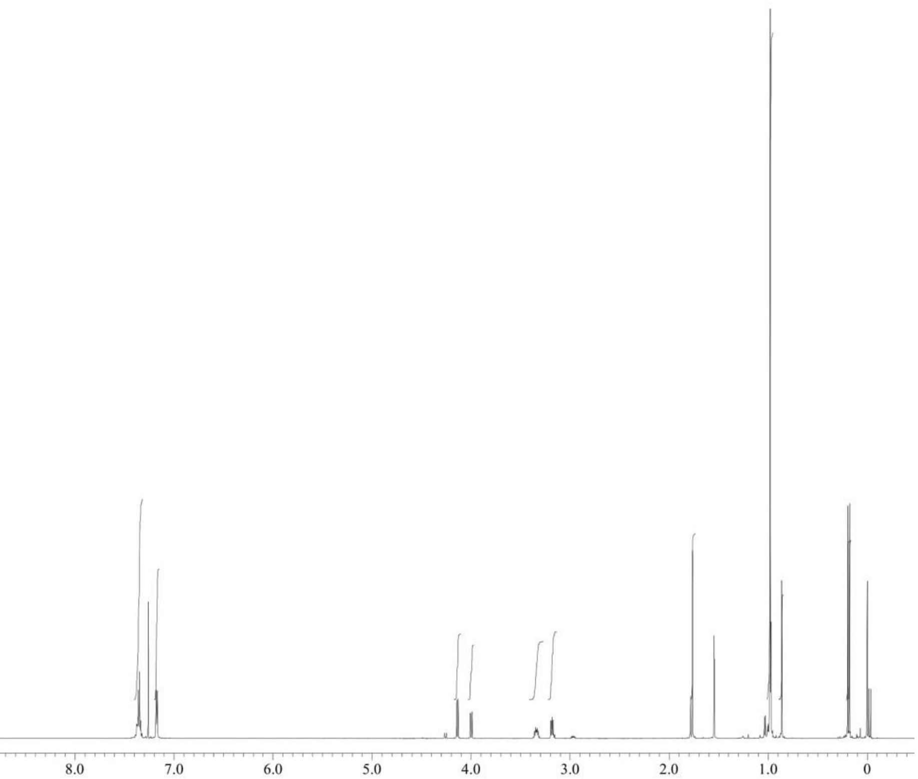

Supplementary Figure 56. ^{1}H NMR spectra of **3q** (minor diastereomer).

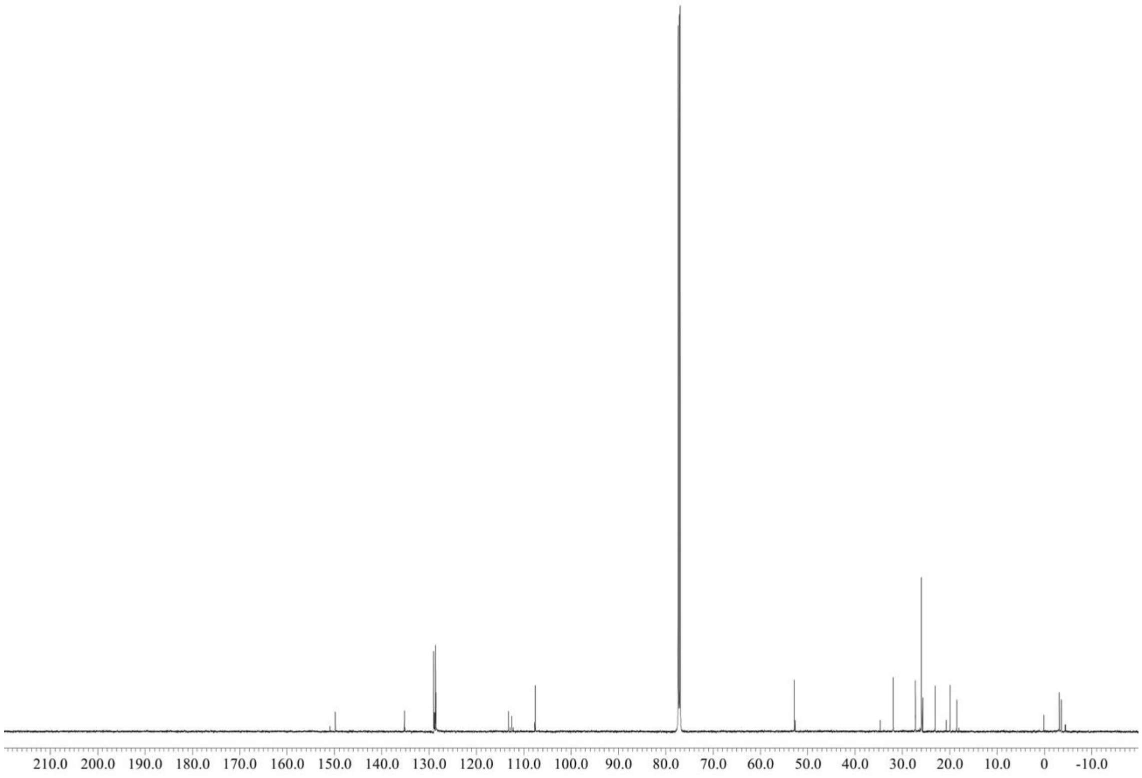

Supplementary Figure 57. ^{13}C NMR spectra of **3q** (minor diastereomer).

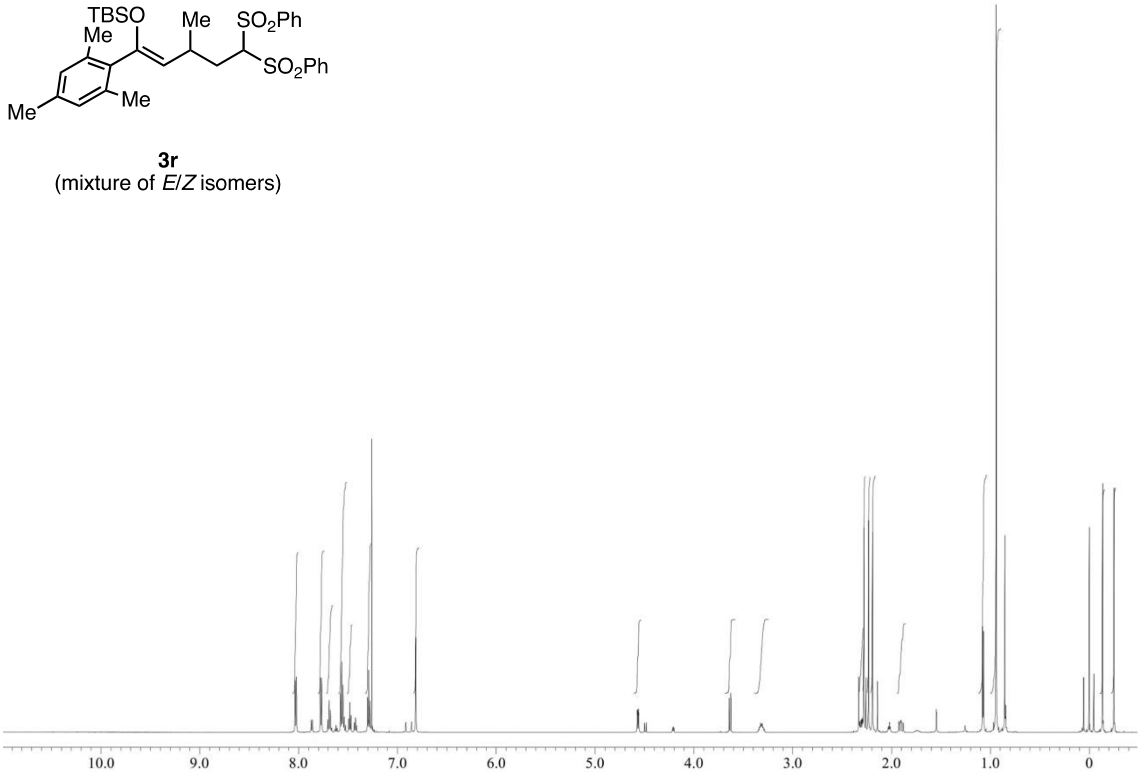

Supplementary Figure 58. ^{1}H NMR spectra of **3r** (mixture of *E*/*Z* isomers).

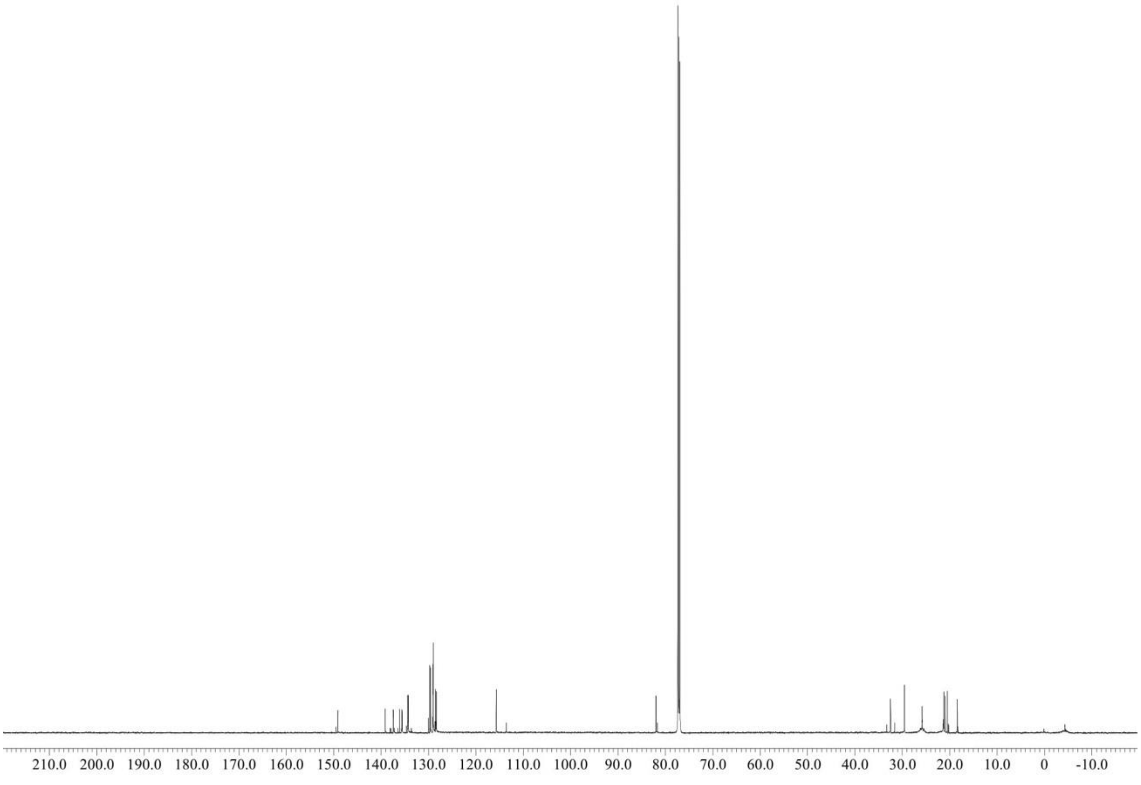

Supplementary Figure 59. ^{13}C NMR spectra of **3r** (mixture of *E*/*Z* isomers).

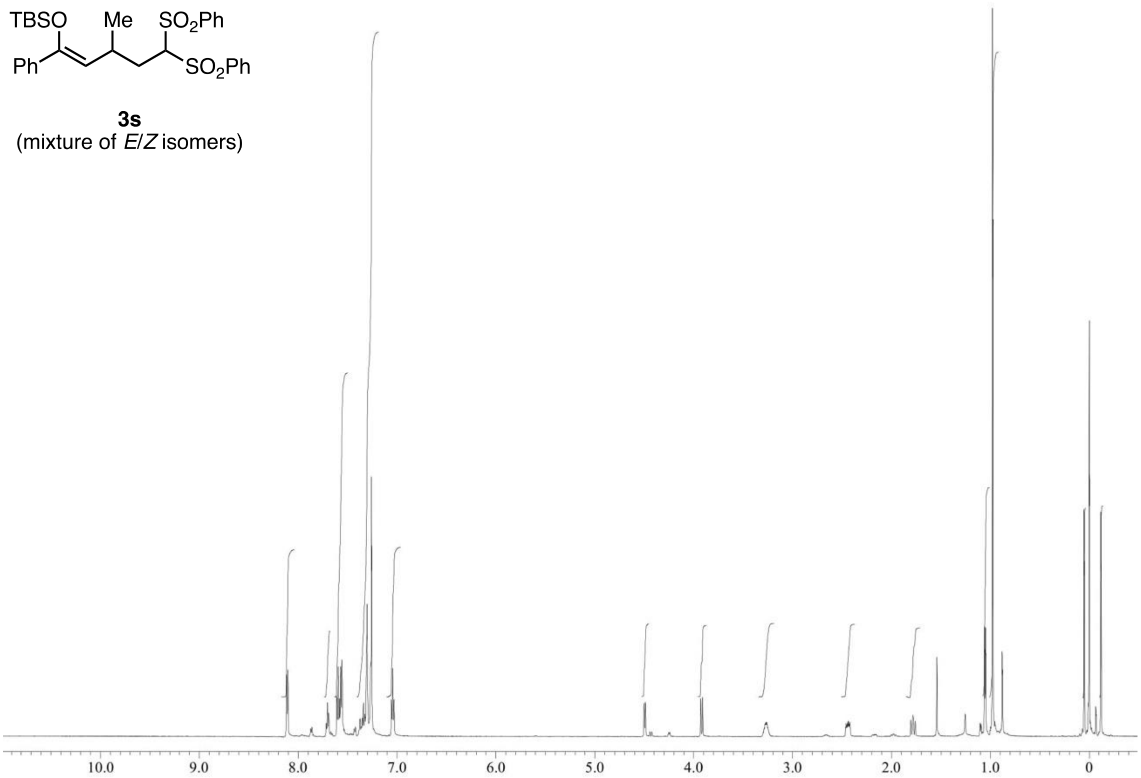

Supplementary Figure 60. ^{1}H NMR spectra of **3s** (mixture of *E*/*Z* isomers).

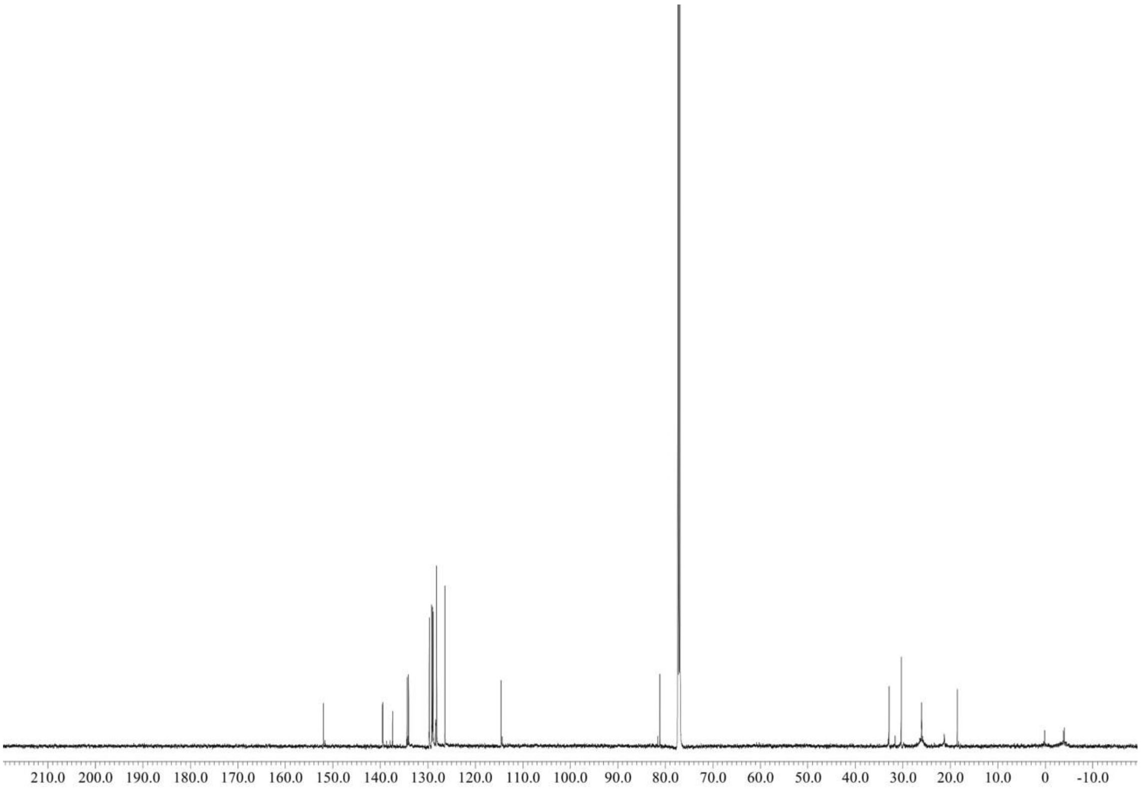

Supplementary Figure 61. ^{13}C NMR spectra of **3s** (mixture of *E*/*Z* isomers).

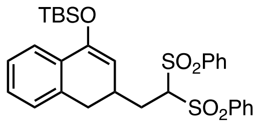

3t

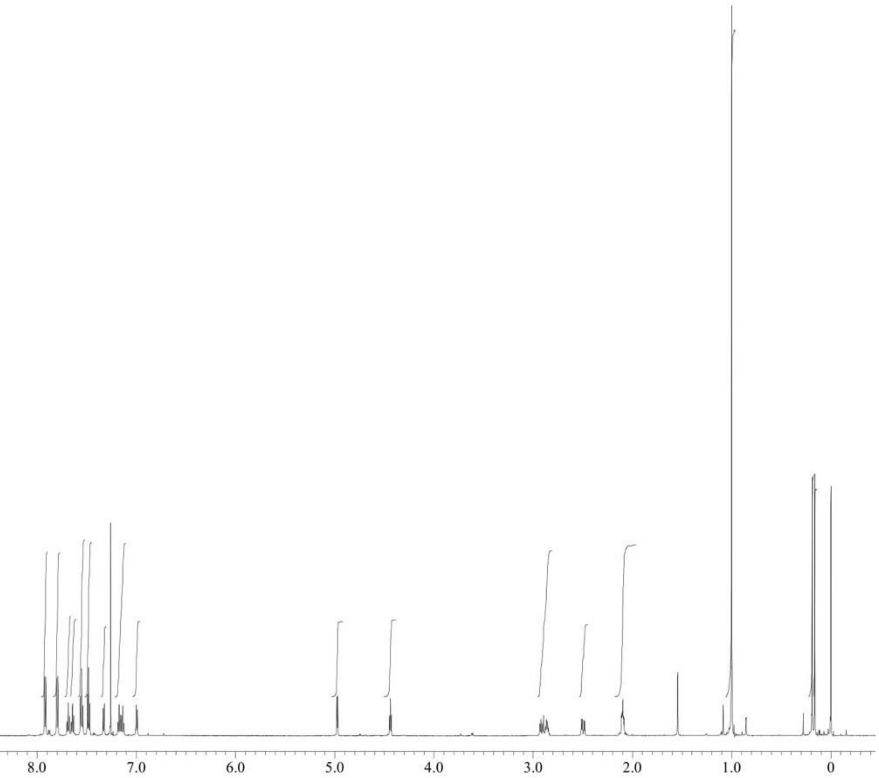

Supplementary Figure 62. ^{1}H NMR spectra of **3t**.

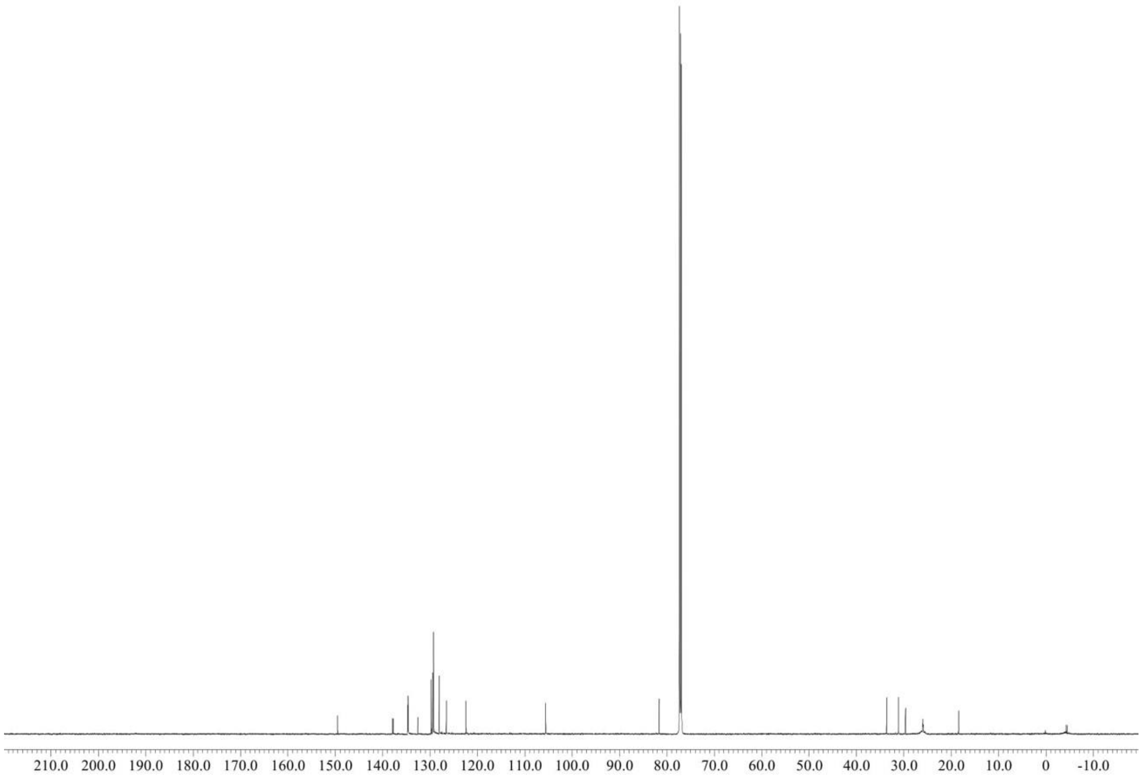

Supplementary Figure 63. ^{13}C NMR spectra of **3t**.

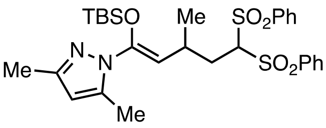

3u
(*Z* isomer)

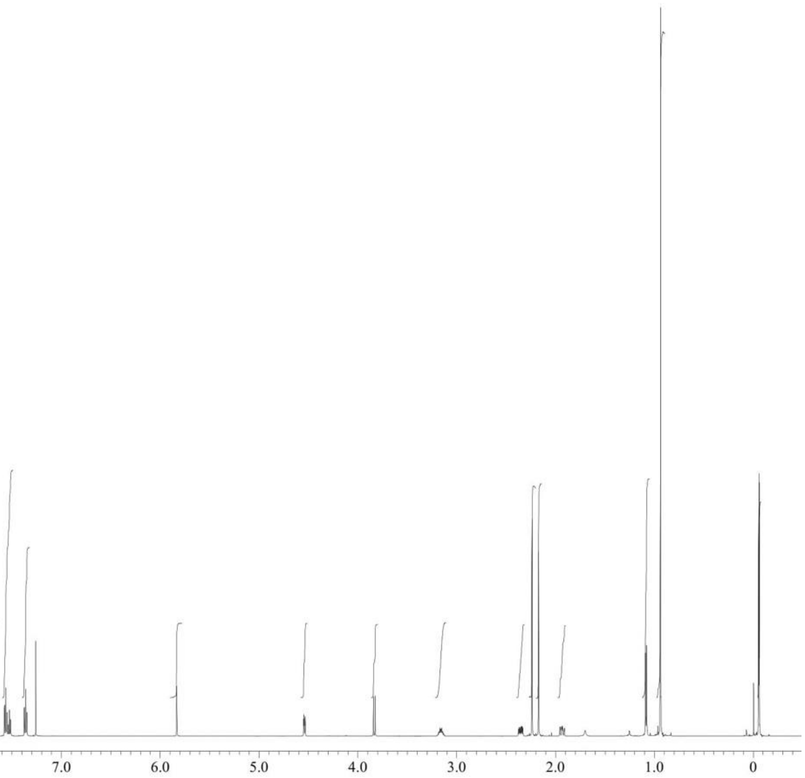

Supplementary Figure 64. ^{1}H NMR spectra of **3u** (*Z* isomer).

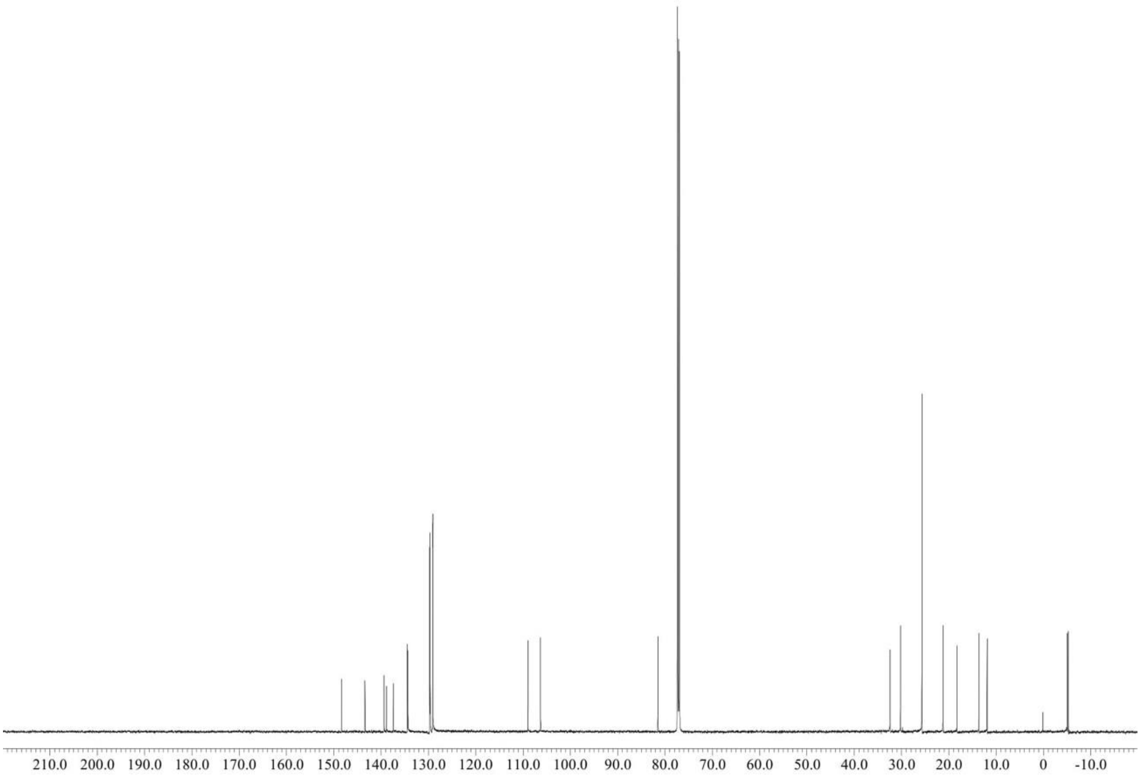

Supplementary Figure 65. ^{13}C NMR spectra of **3u** (*Z* isomer).

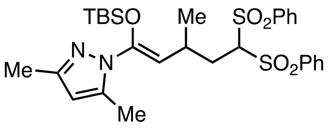

3u
(*E* isomer)

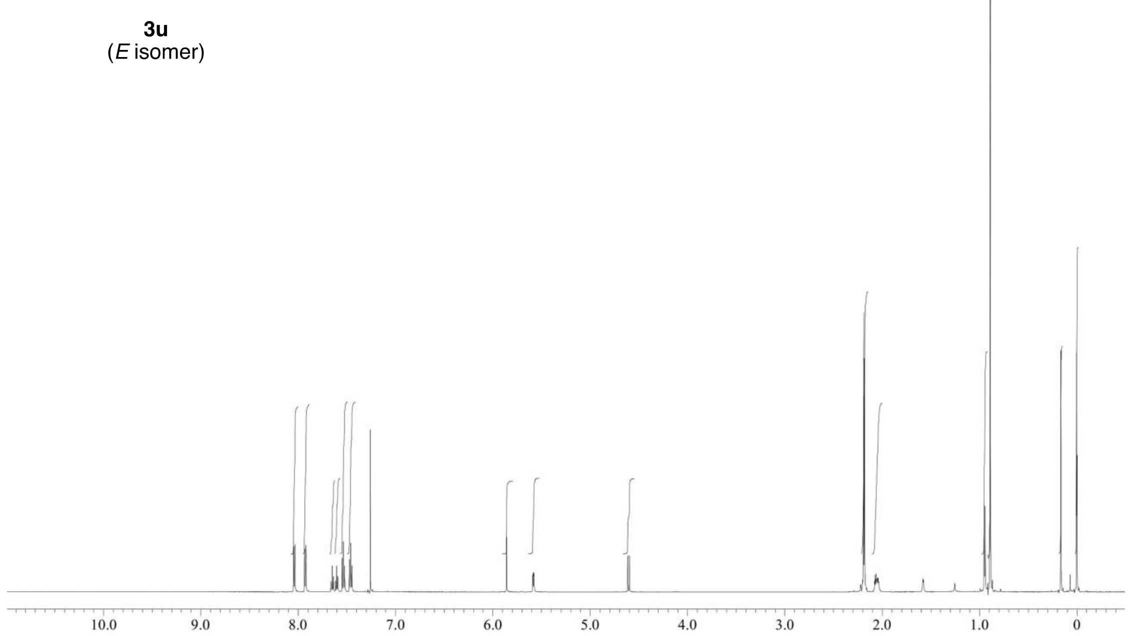

Supplementary Figure 66. ^{1}H NMR spectra of **3u** (*E* isomer).

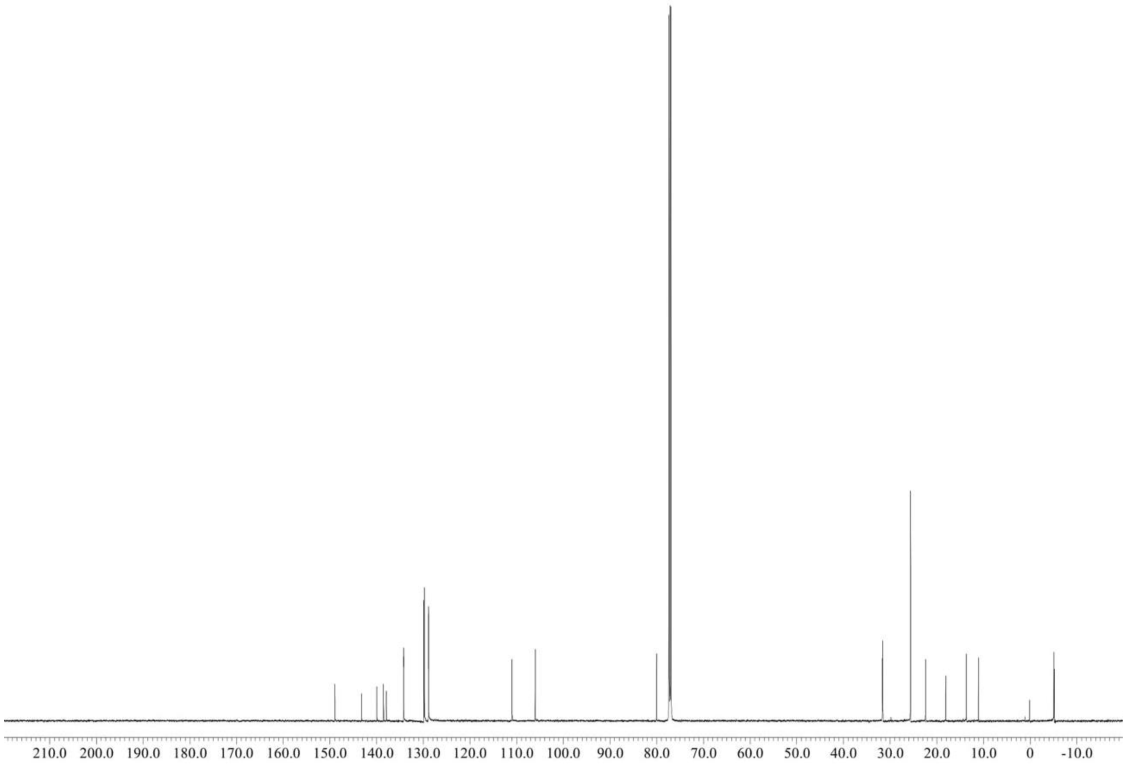

Supplementary Figure 67. ^{13}C NMR spectra of **3u** (*E* isomer).

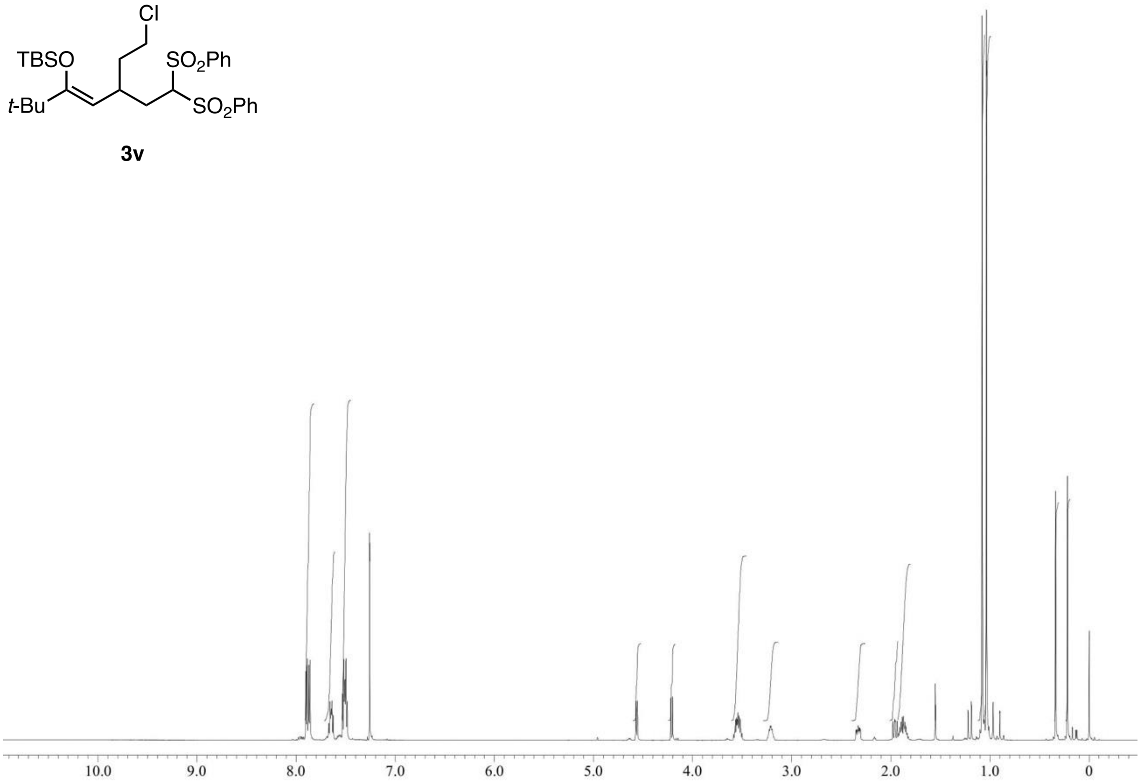

Supplementary Figure 68. ^{1}H NMR spectra of **3v**.

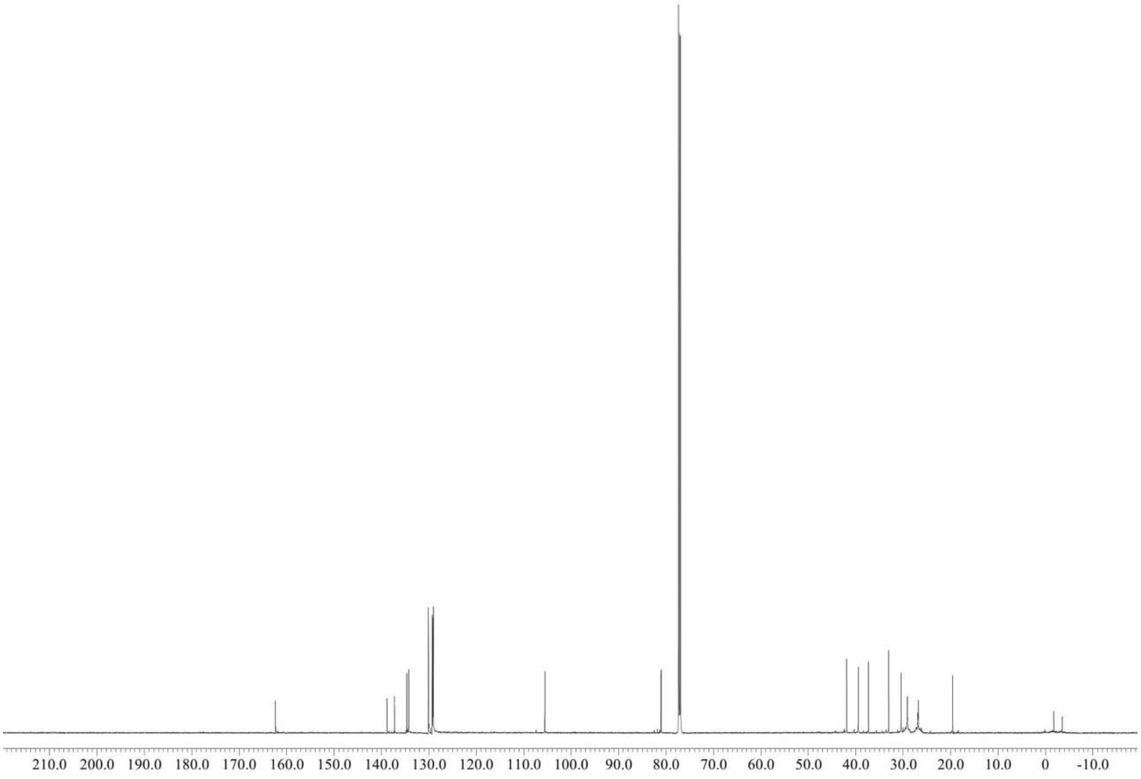

Supplementary Figure 69. ^{13}C NMR spectra of **3v**.

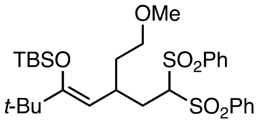

3w

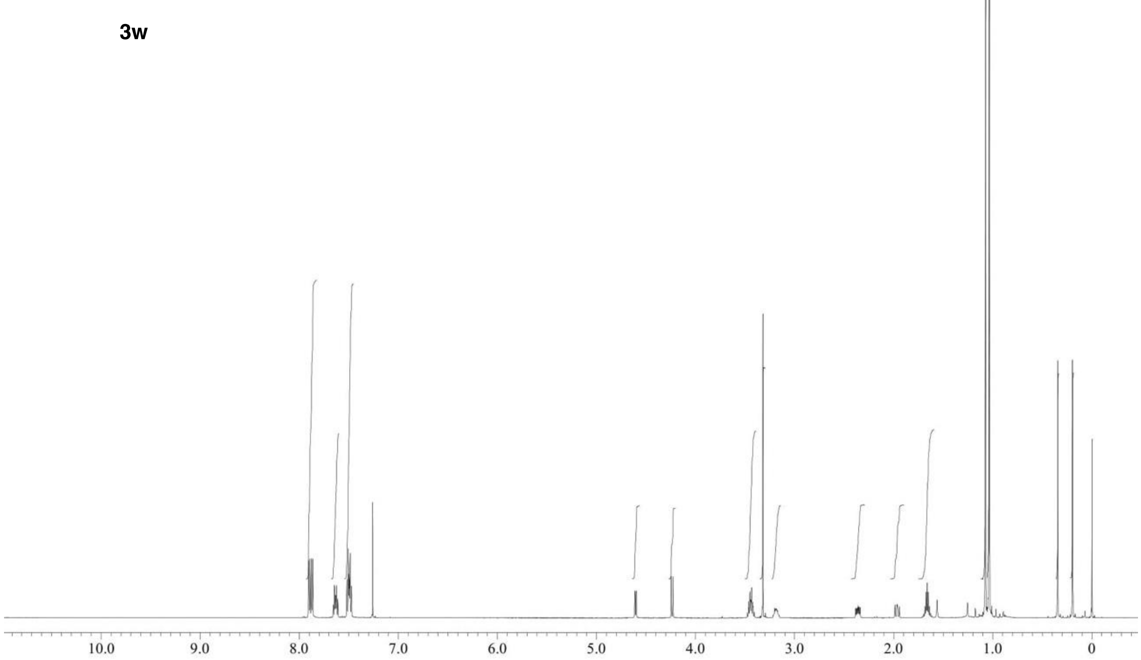

Supplementary Figure 70. ^{1}H NMR spectra of **3w**.

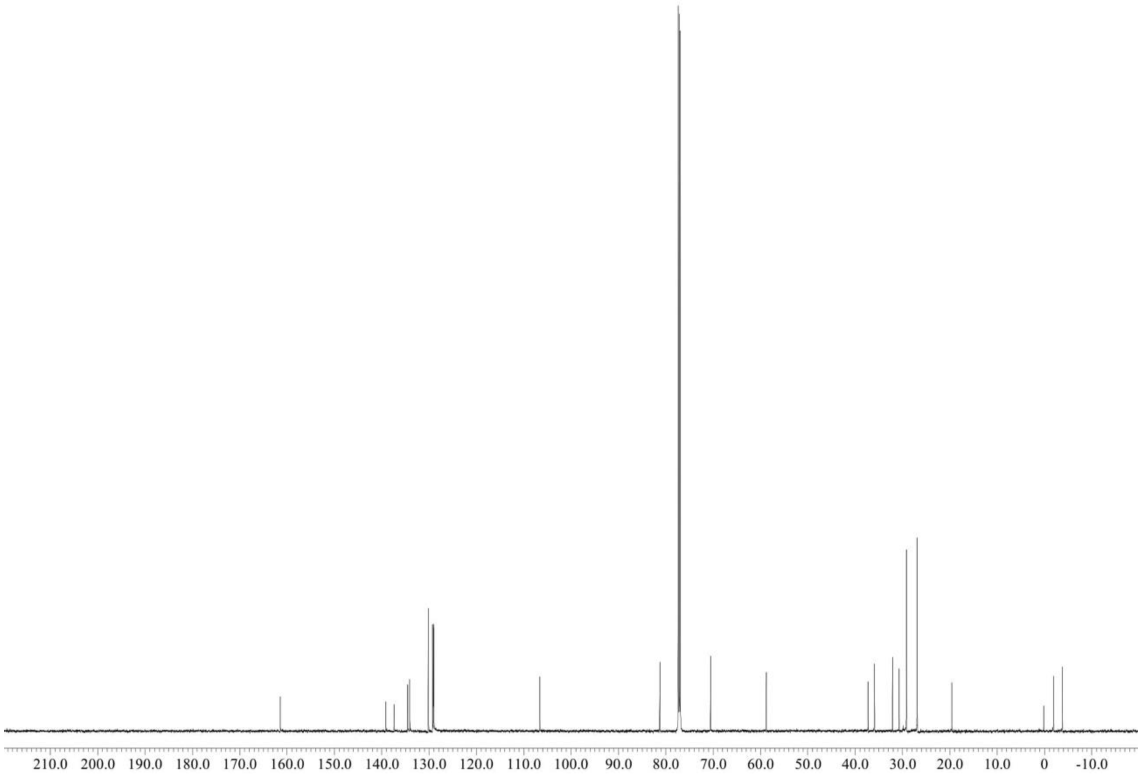

Supplementary Figure 71. ^{13}C NMR spectra of **3w**.

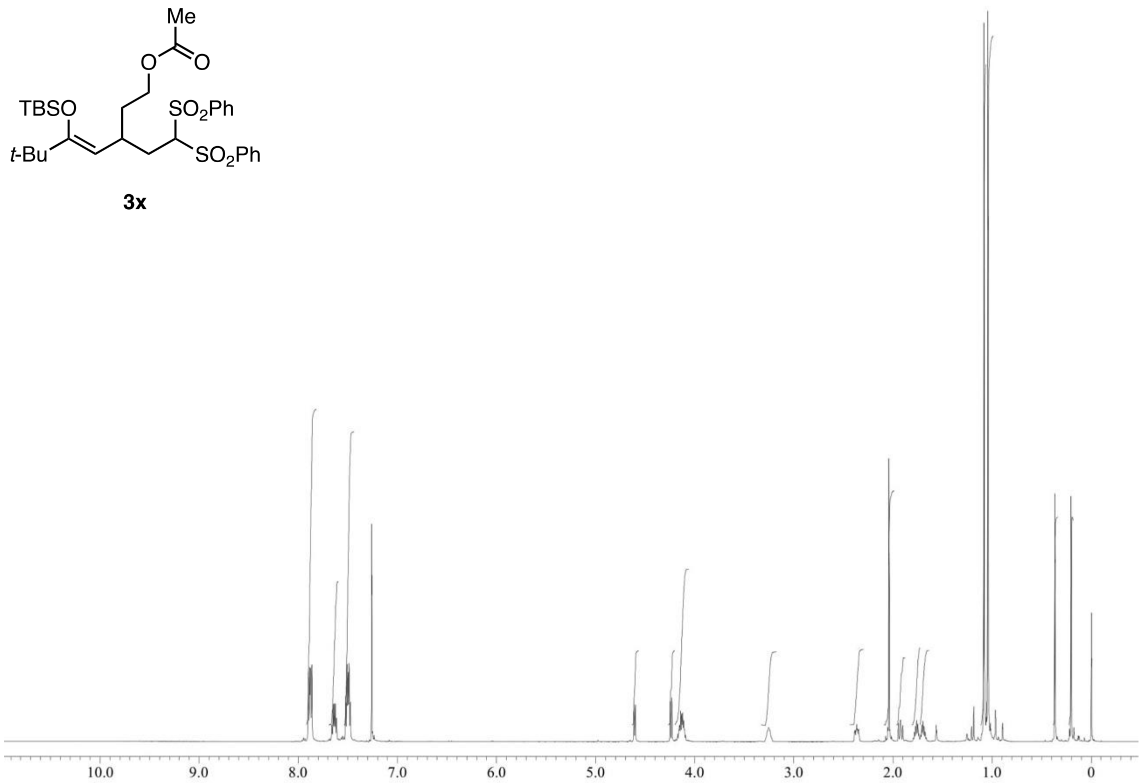

Supplementary Figure 72. ^{1}H NMR spectra of **3x**.

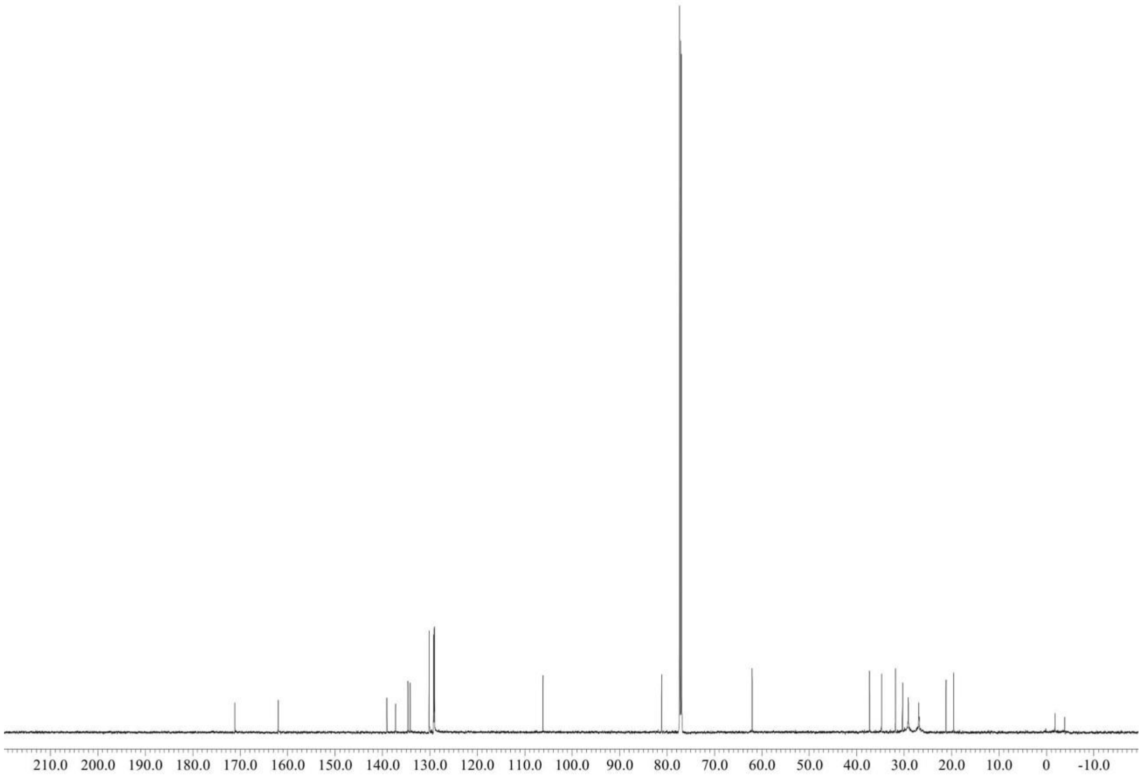

Supplementary Figure 73. ^{13}C NMR spectra of **3x**.

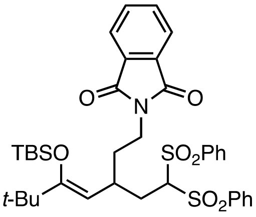

3y

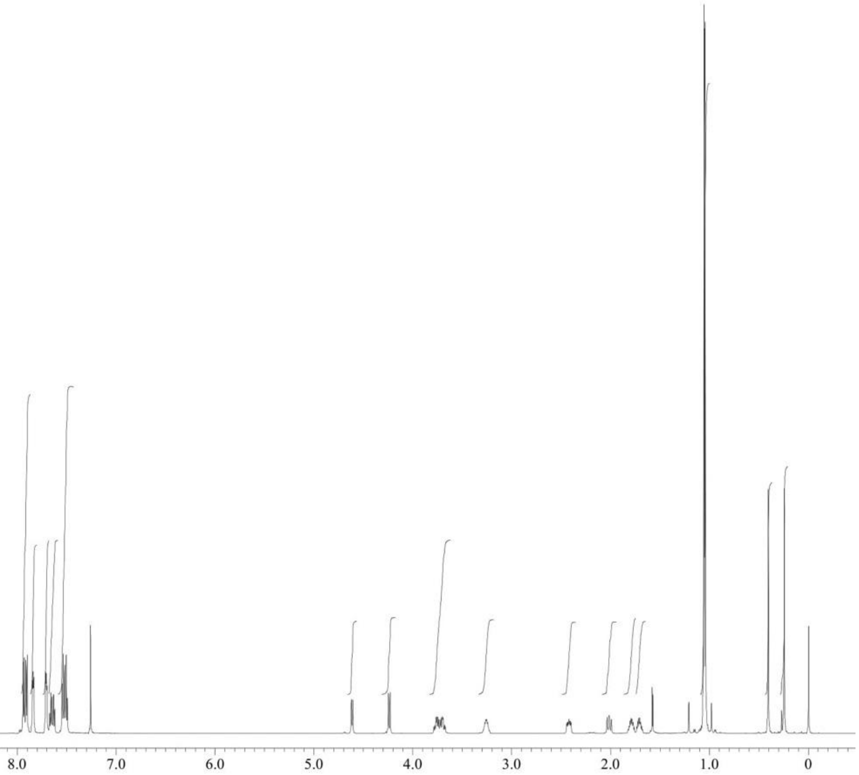

Supplementary Figure 74. ^{1}H NMR spectra of **3y**.

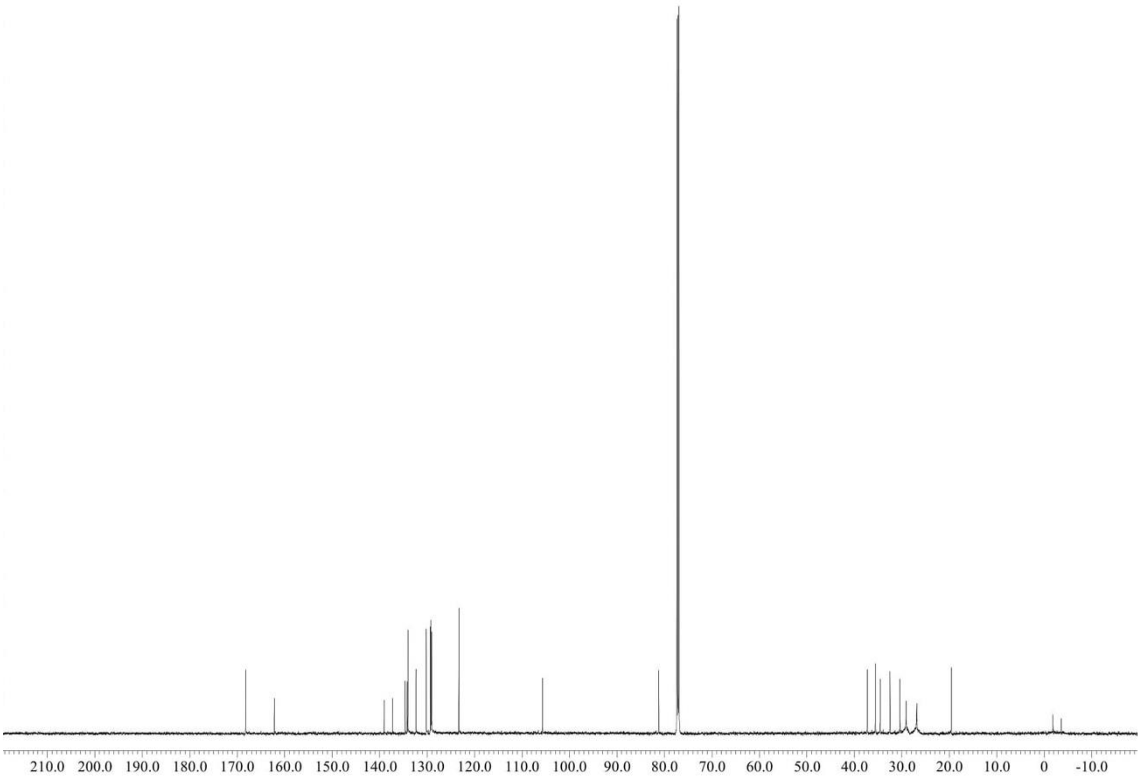

Supplementary Figure 75. ^{13}C NMR spectra of **3y**.

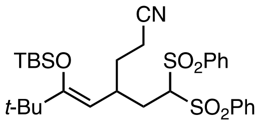

3z

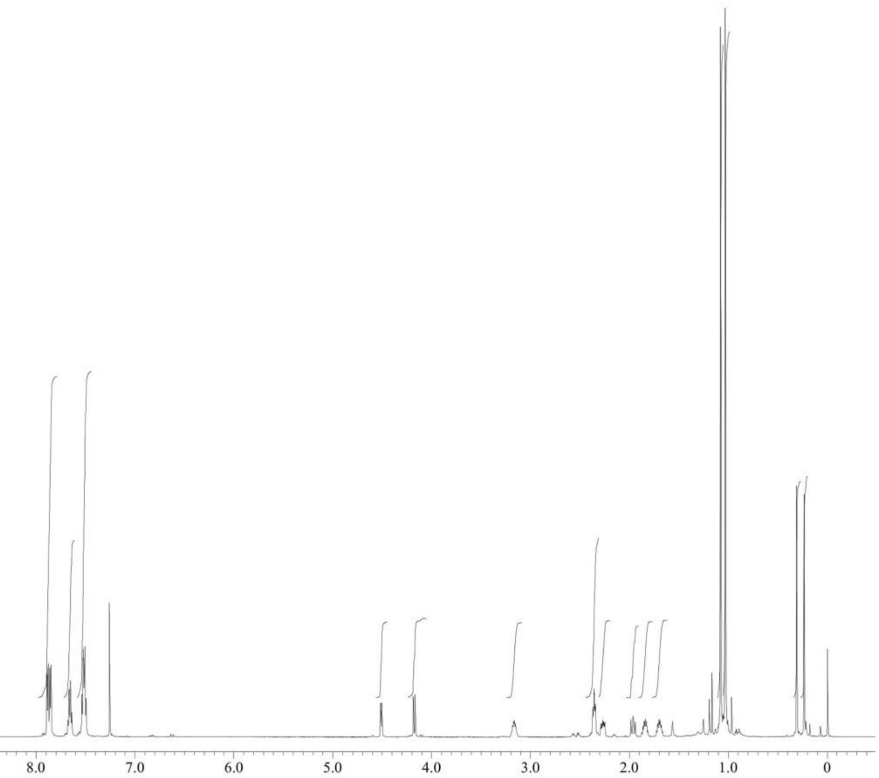

Supplementary Figure 76. ^{1}H NMR spectra of **3z**.

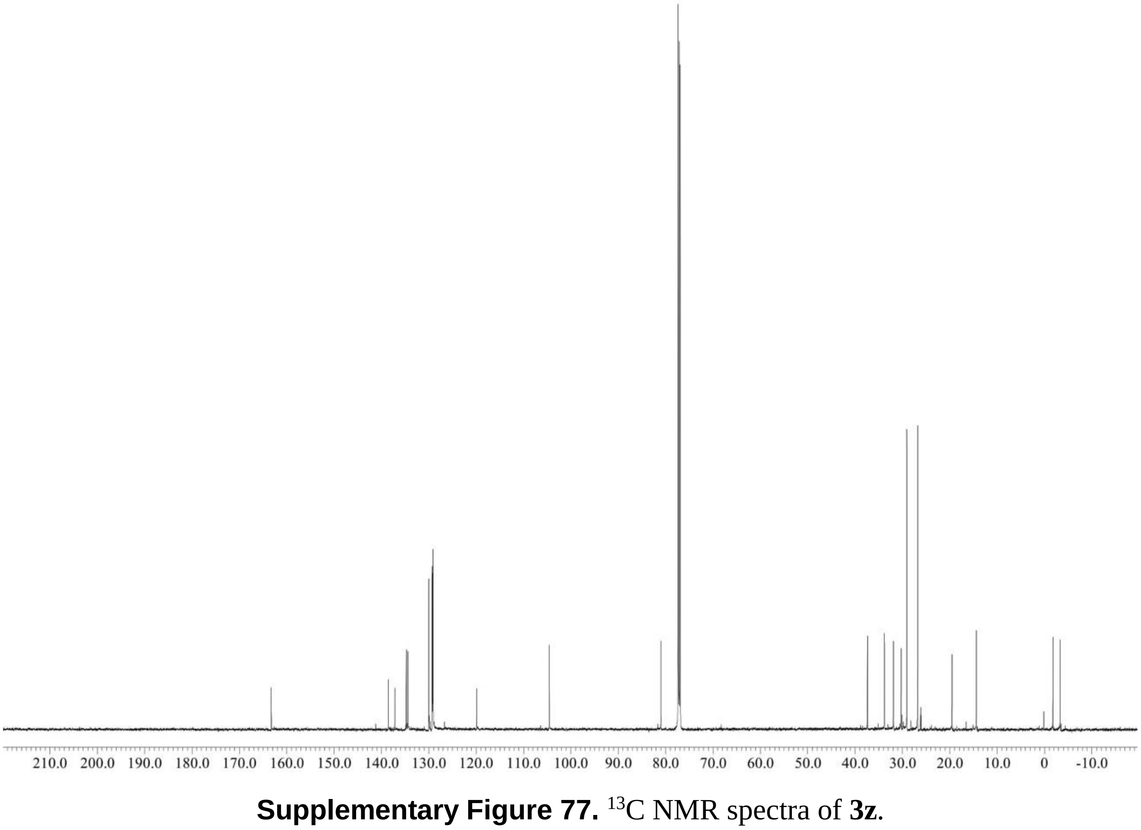

Supplementary Figure 77. ^{13}C NMR spectra of **3z**.

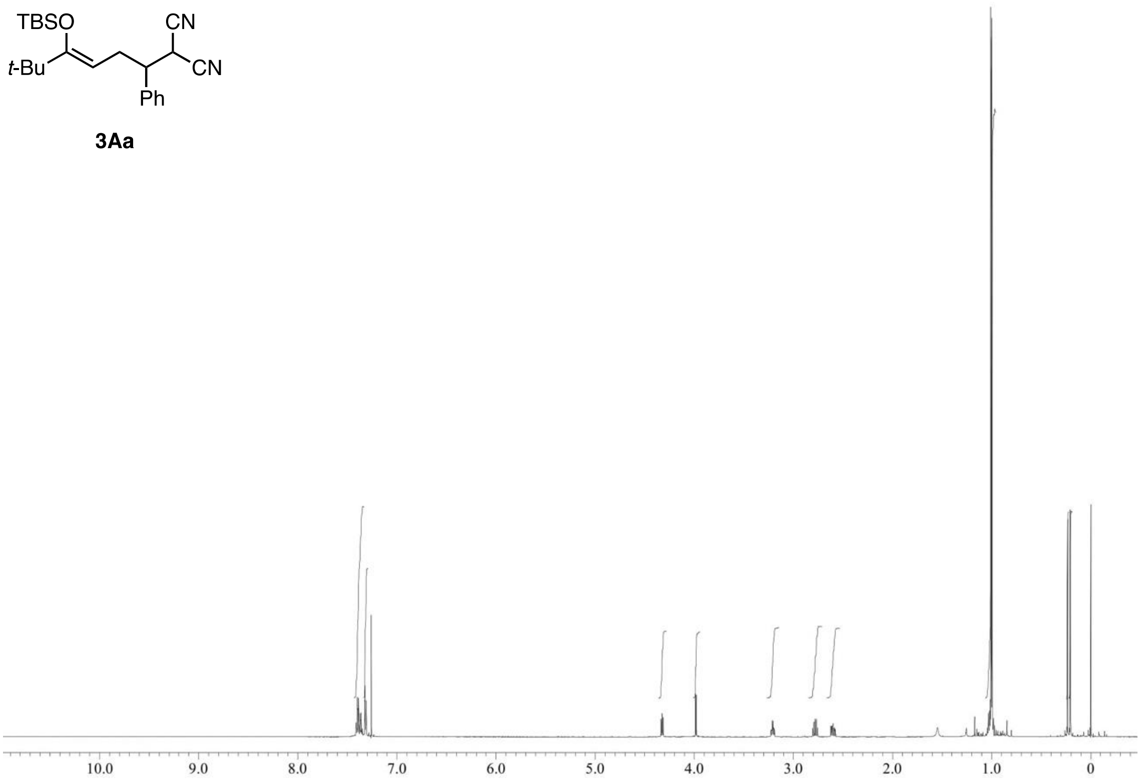

Supplementary Figure 78. ^{1}H NMR spectra of **3Aa**.

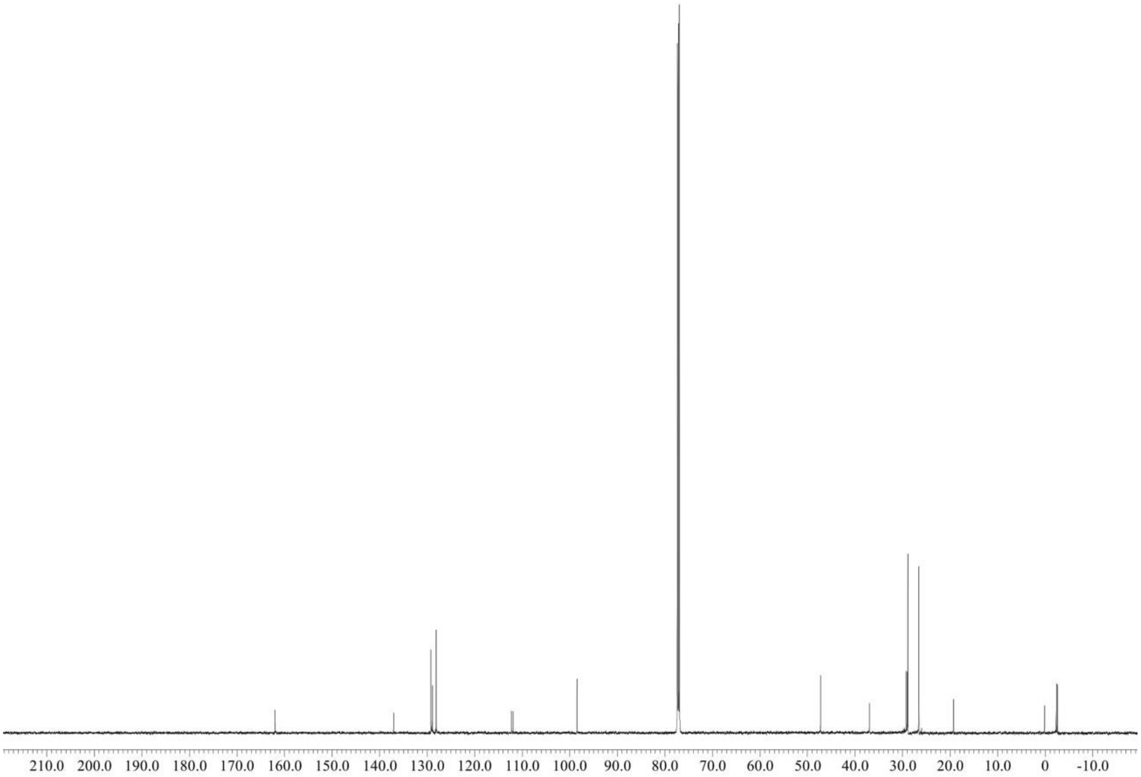

Supplementary Figure 79. ^{13}C NMR spectra of **3Aa**.

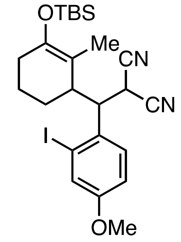

3Ab
(major diastereomer)

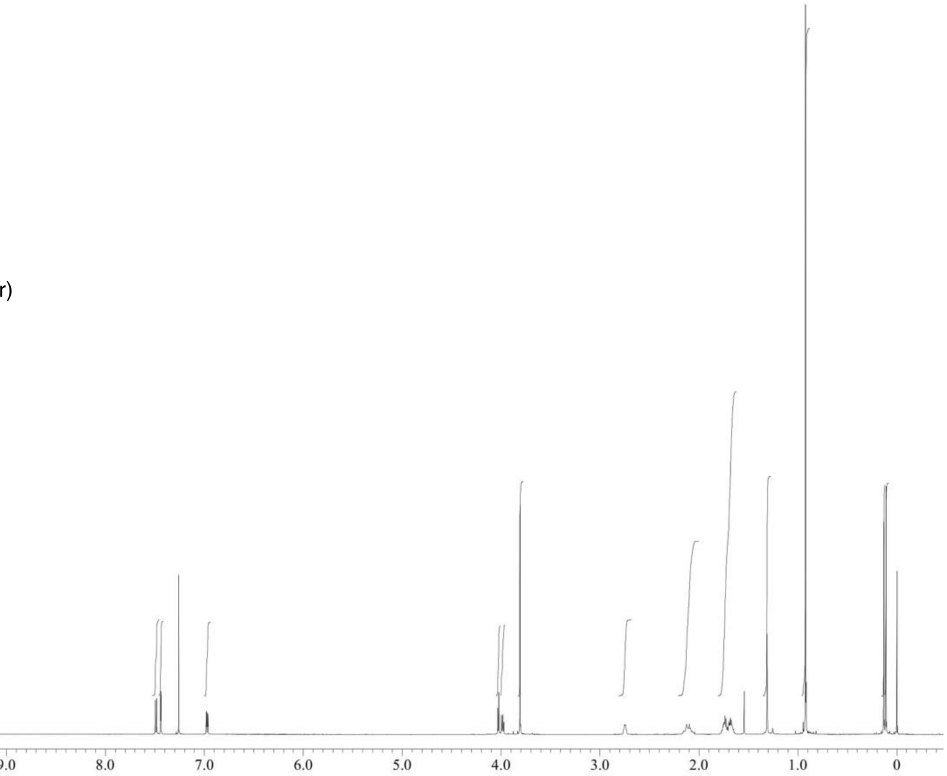

Supplementary Figure 80. ^{1}H NMR spectra of **3Ab** (major diastereomer).

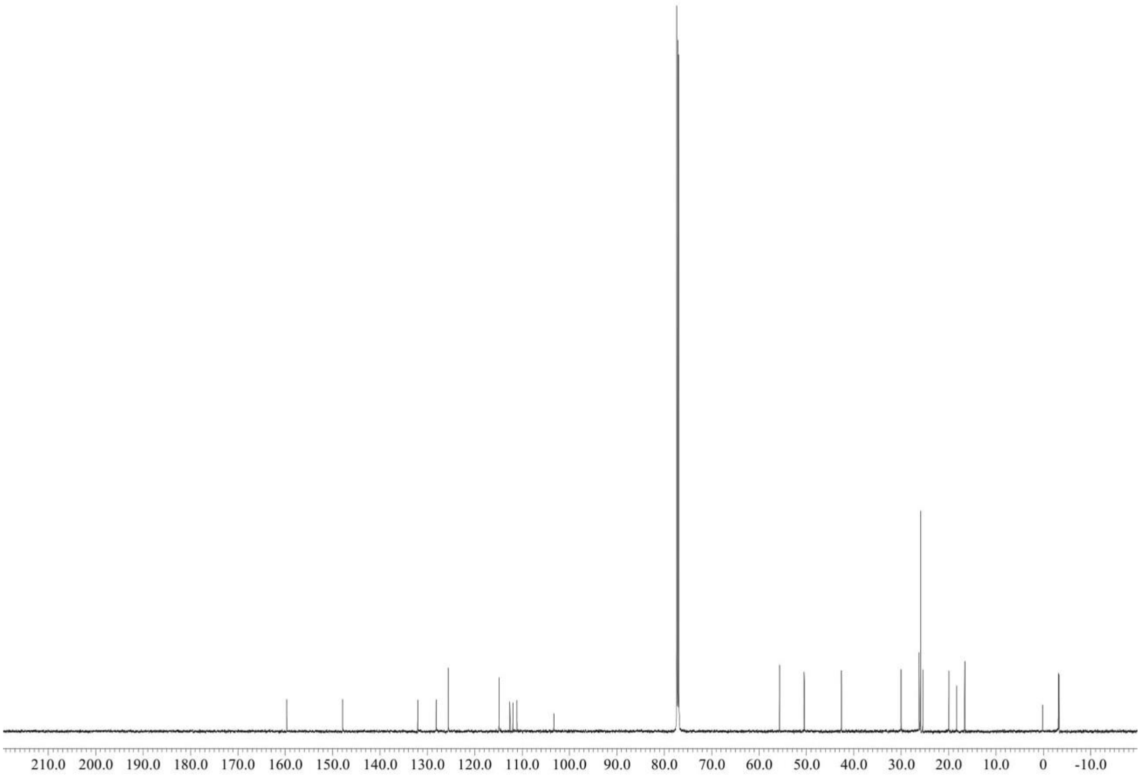

Supplementary Figure 81. ^{13}C NMR spectra of **3Ab** (major diastereomer).

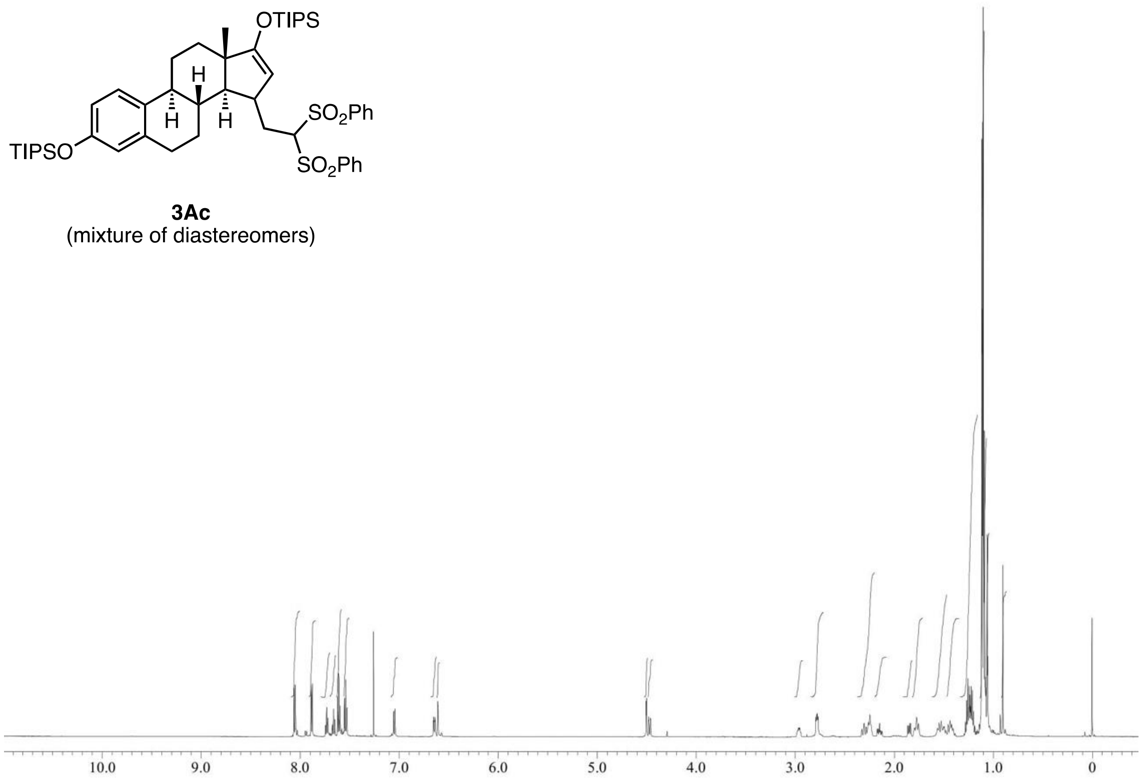

Supplementary Figure 82. ^{1}H NMR spectra of **3Ac** (mixture of diastereomers).

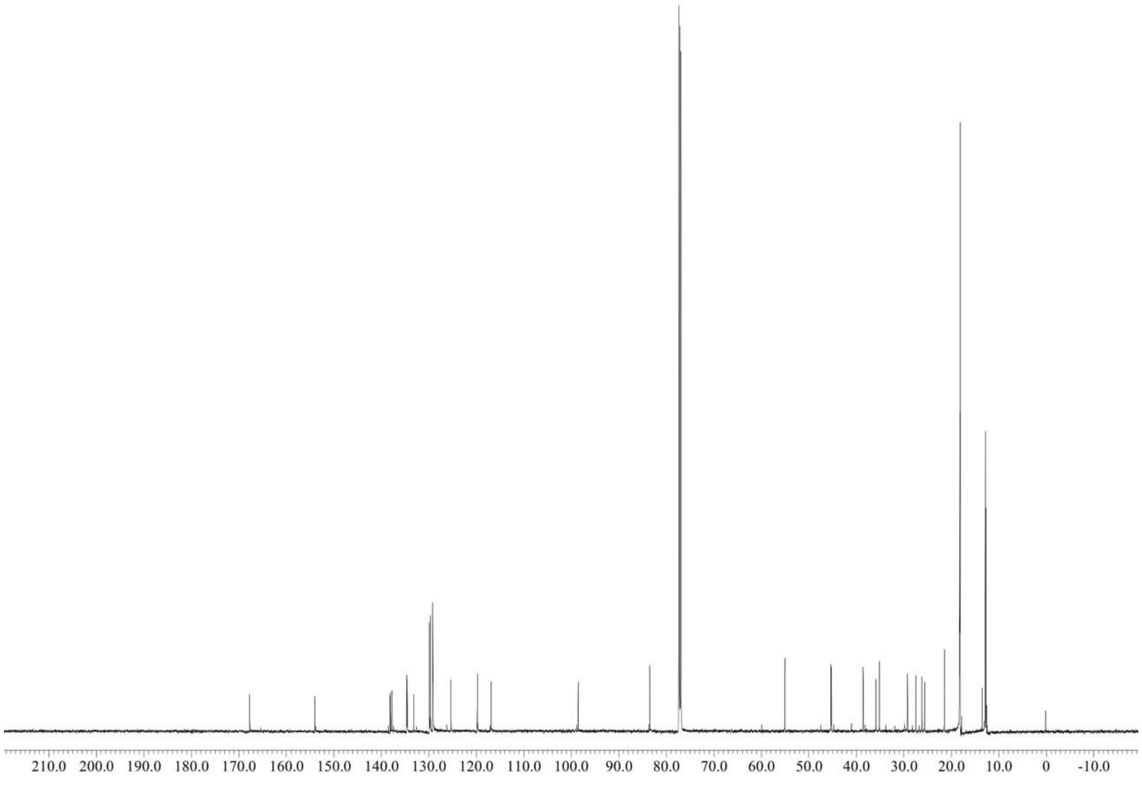

Supplementary Figure 83. ^{13}C NMR spectra of **3Ac** (mixture of diastereomers).

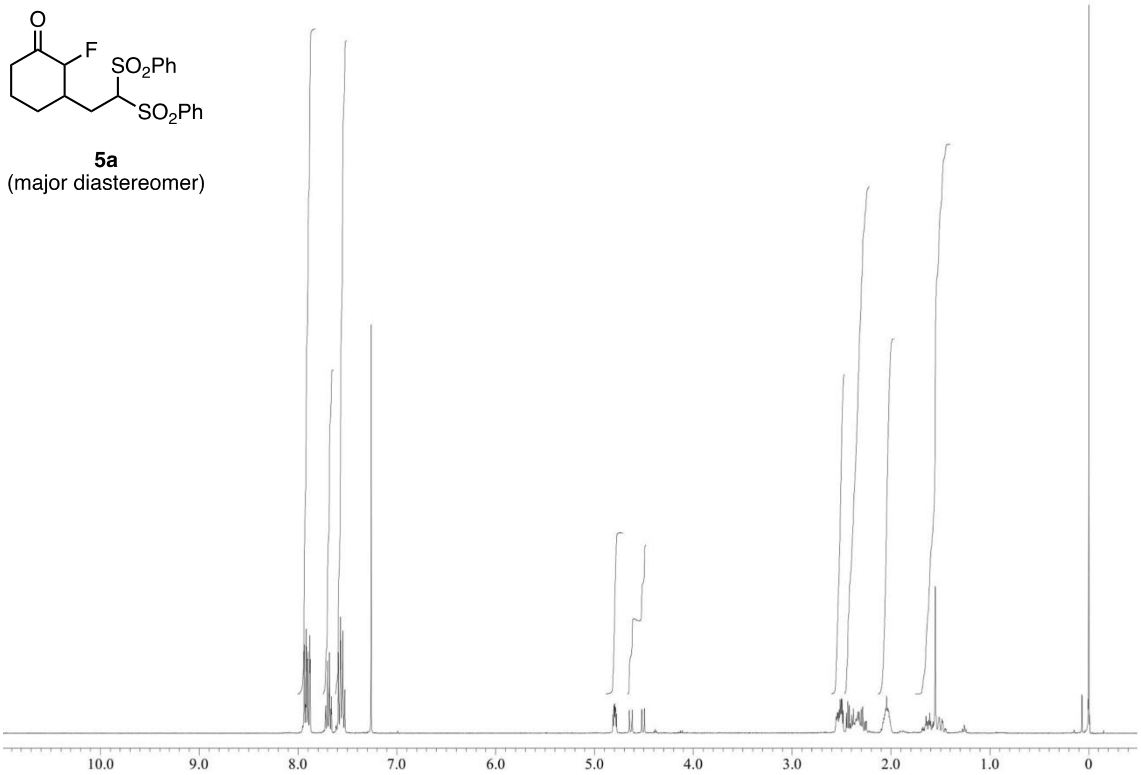

Supplementary Figure 84. ^{1}H NMR spectra of **5a** (major diastereomer).

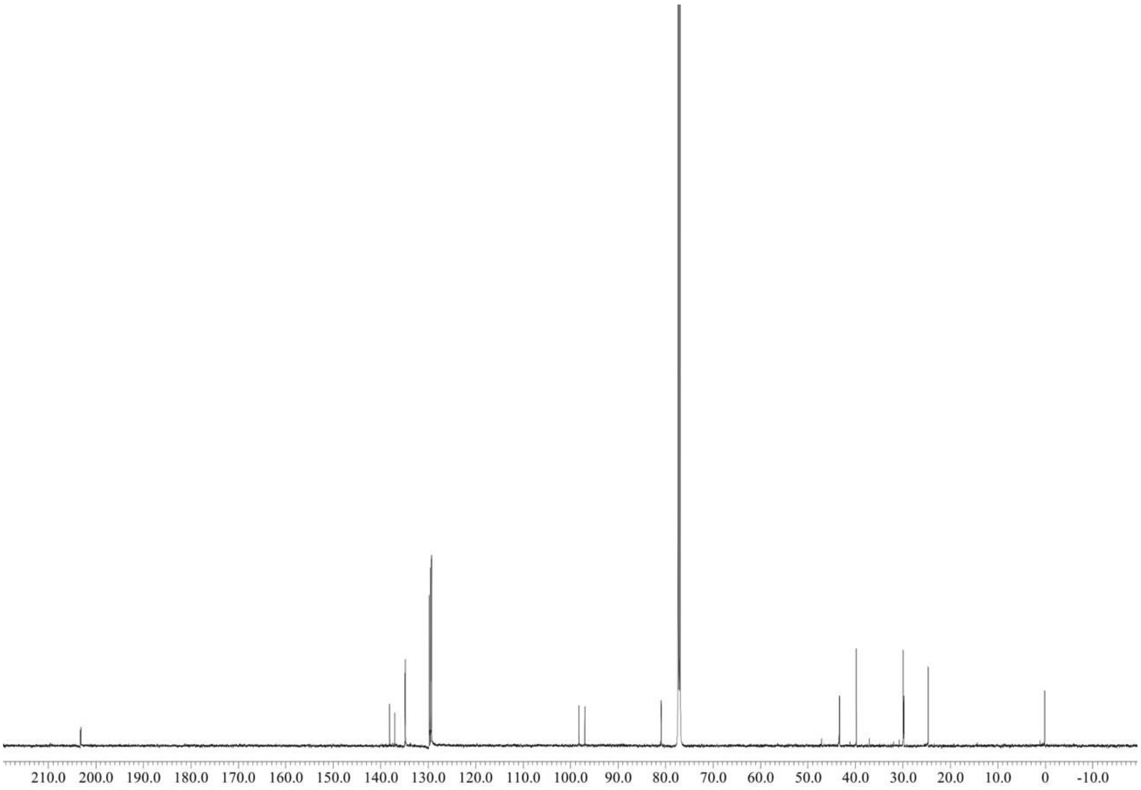

Supplementary Figure 85. ^{13}C NMR spectra of **5a** (major diastereomer).

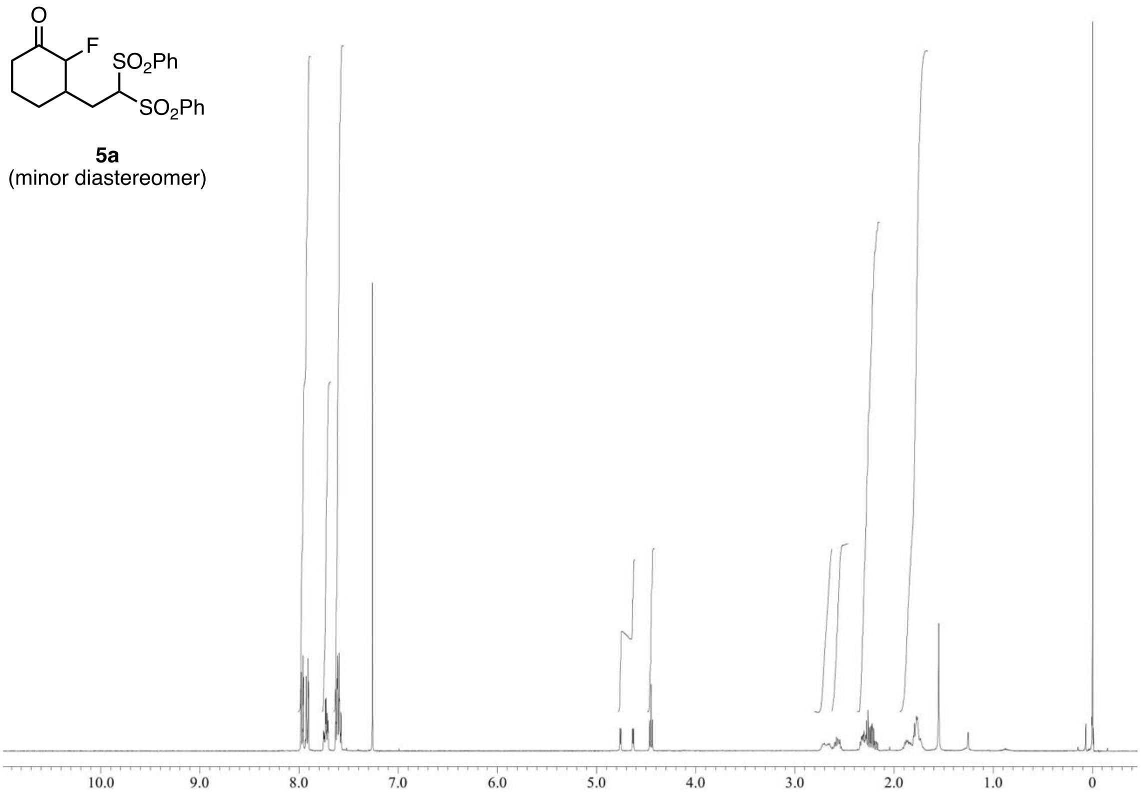

Supplementary Figure 86. ^{1}H NMR spectra of **5a** (minor diastereomer).

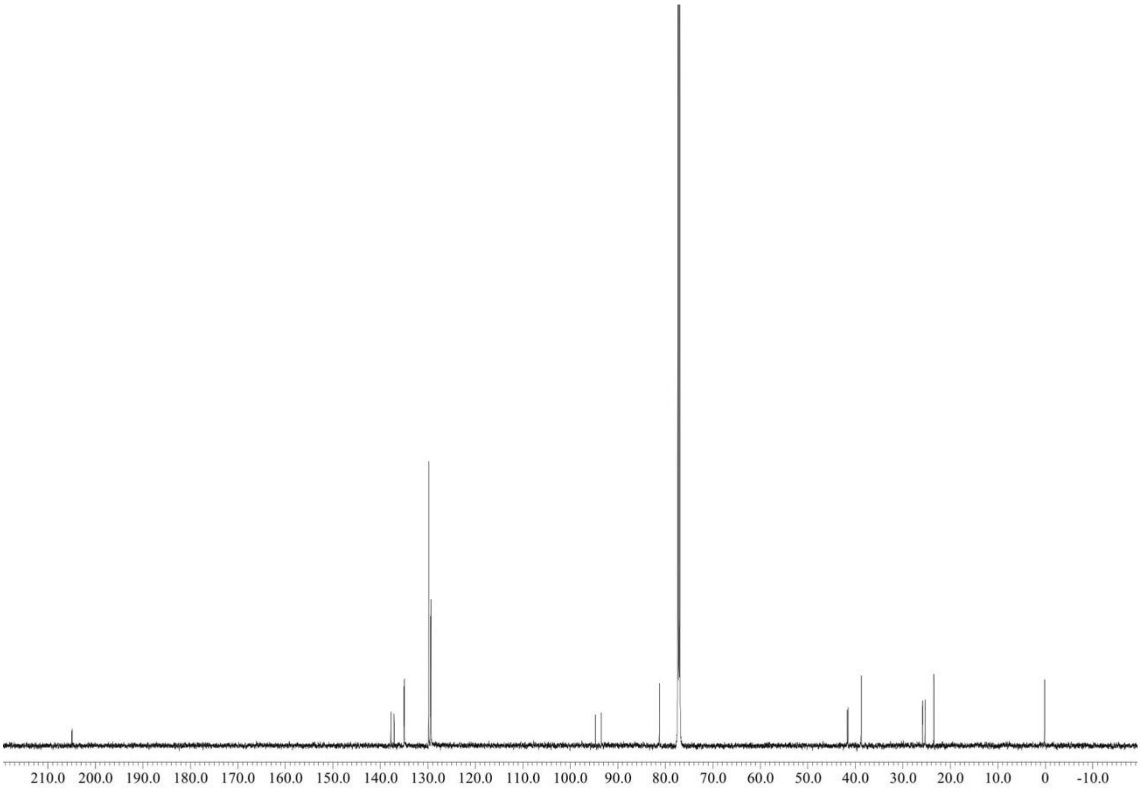

Supplementary Figure 87. ^{13}C NMR spectra of **5a** (minor diastereomer).

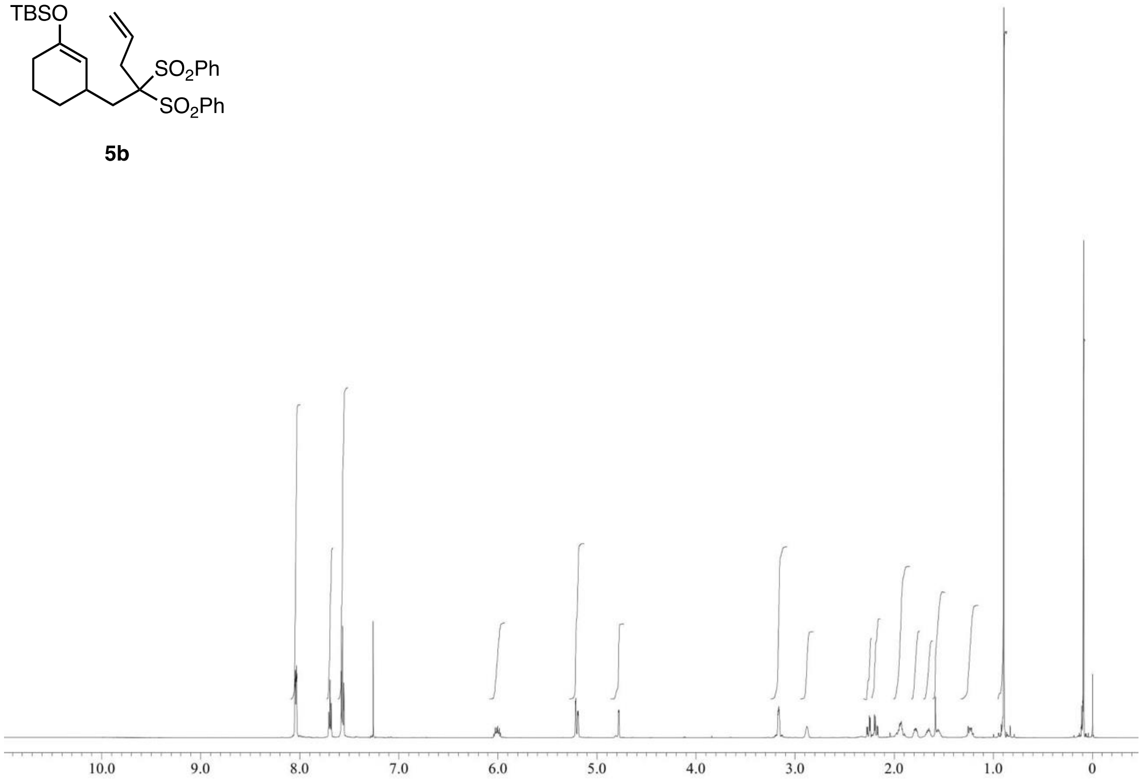

Supplementary Figure 88. ^{1}H NMR spectra of **5b**.

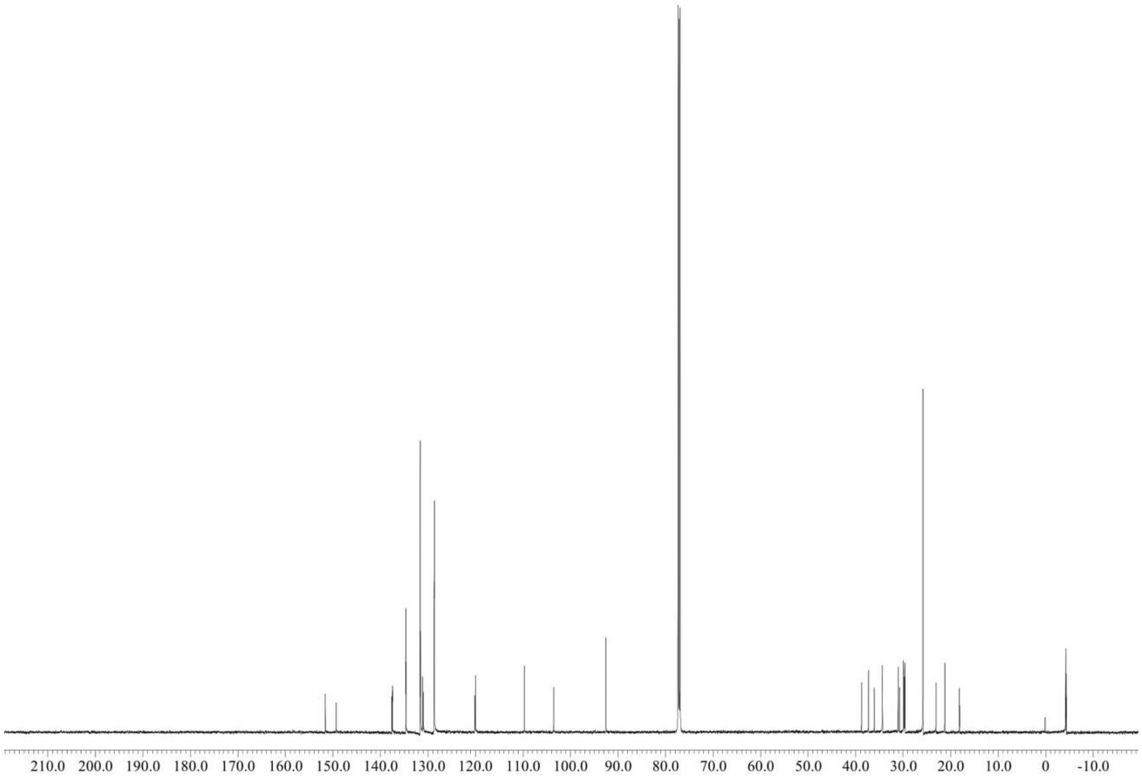

Supplementary Figure 89. ^{13}C NMR spectra of **5b**.

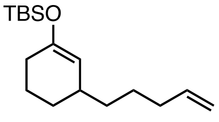

5c

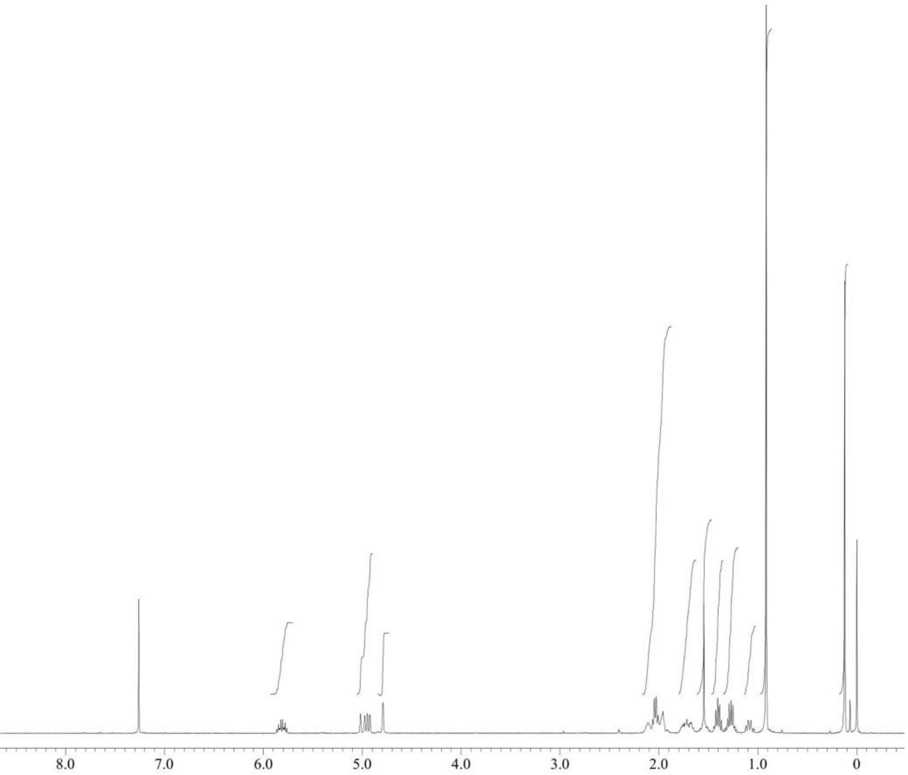

Supplementary Figure 90. ^{1}H NMR spectra of **5c**.

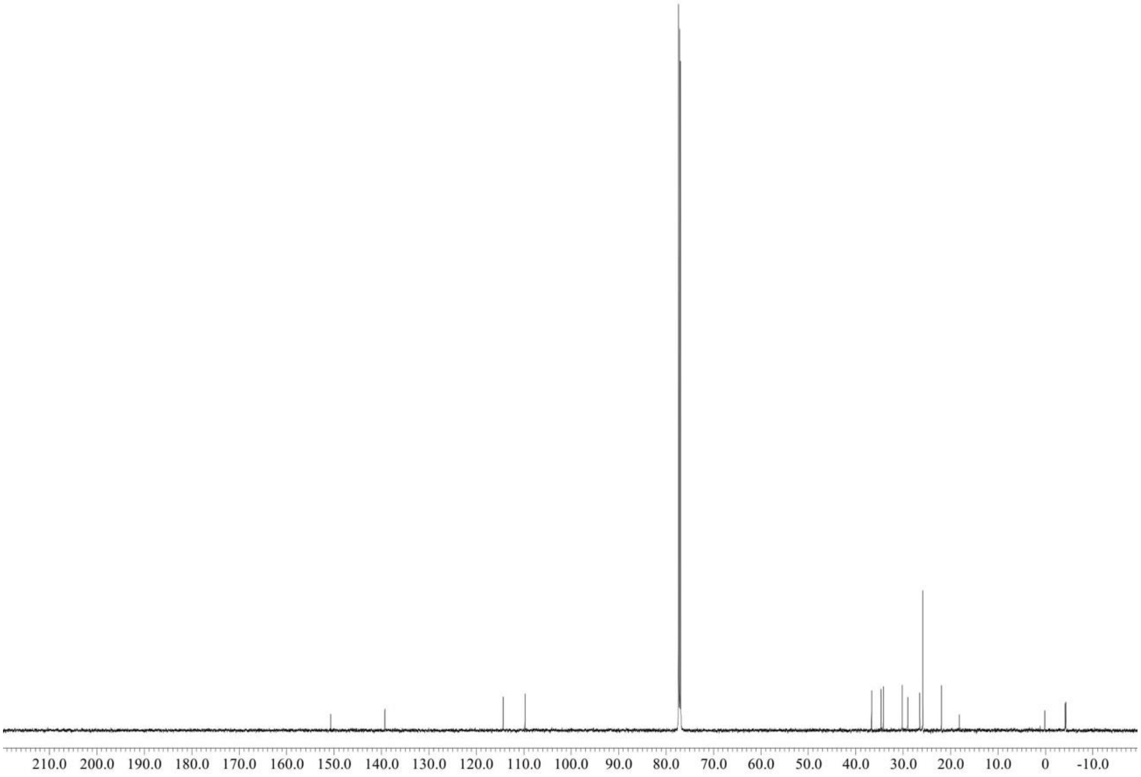

Supplementary Figure 91. ^{13}C NMR spectra of **5c**.

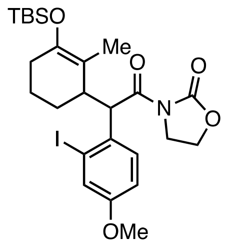

5d

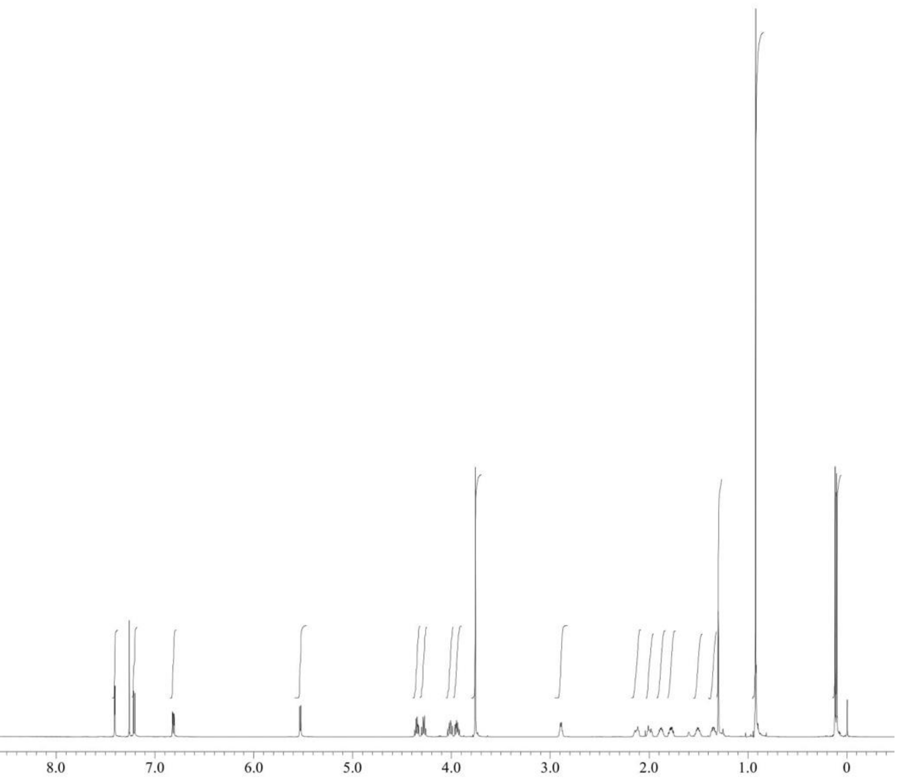

Supplementary Figure 92. ^{1}H NMR spectra of **5d**.

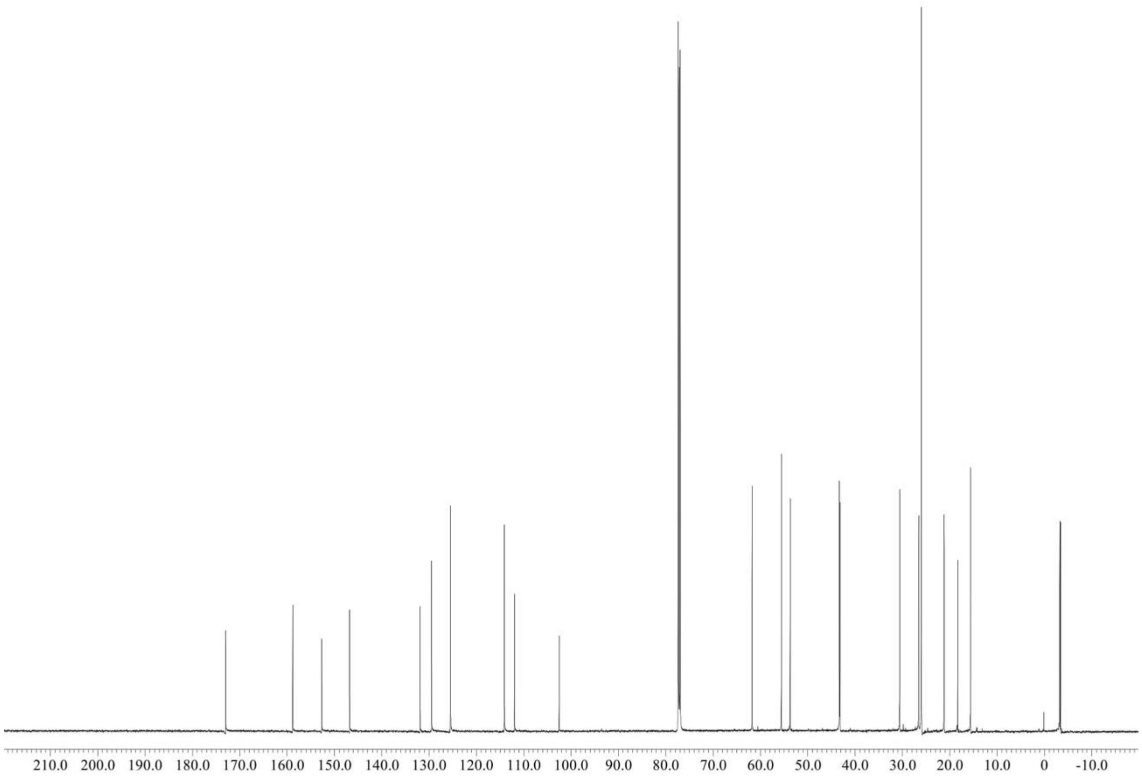

Supplementary Figure 93. ^{13}C NMR spectra of **5d**.

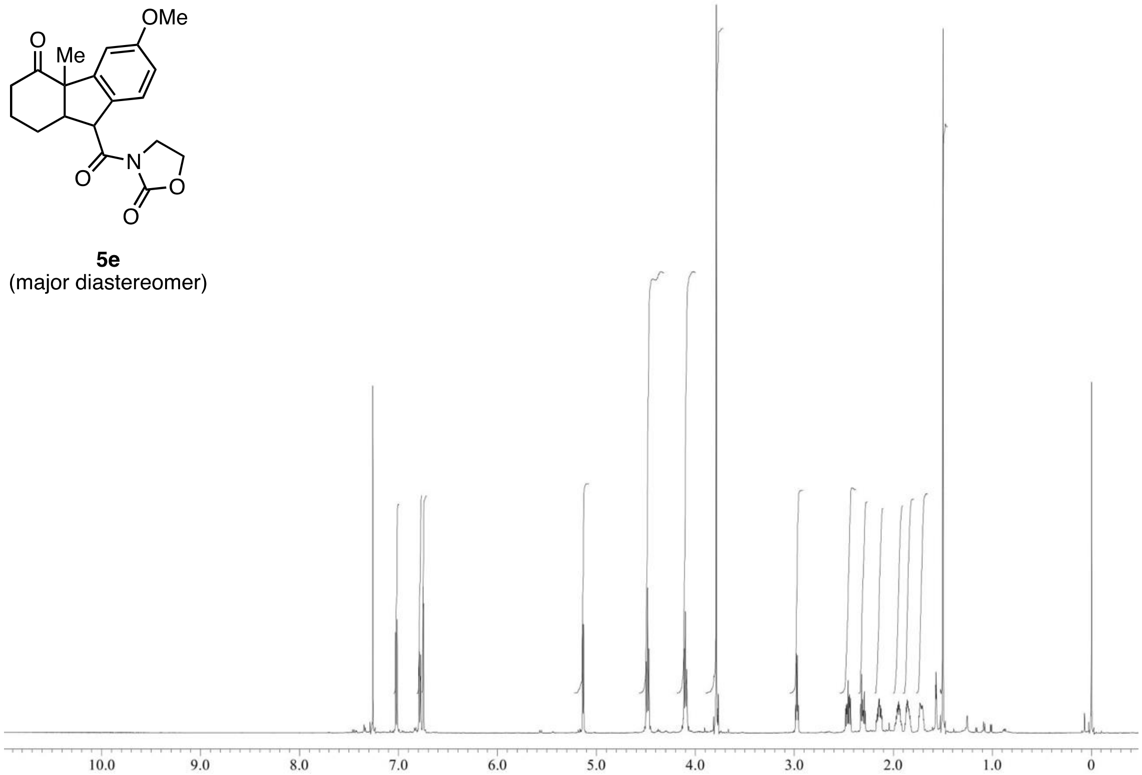

Supplementary Figure 94. ^{1}H NMR spectra of **5e** (major diastereomer).

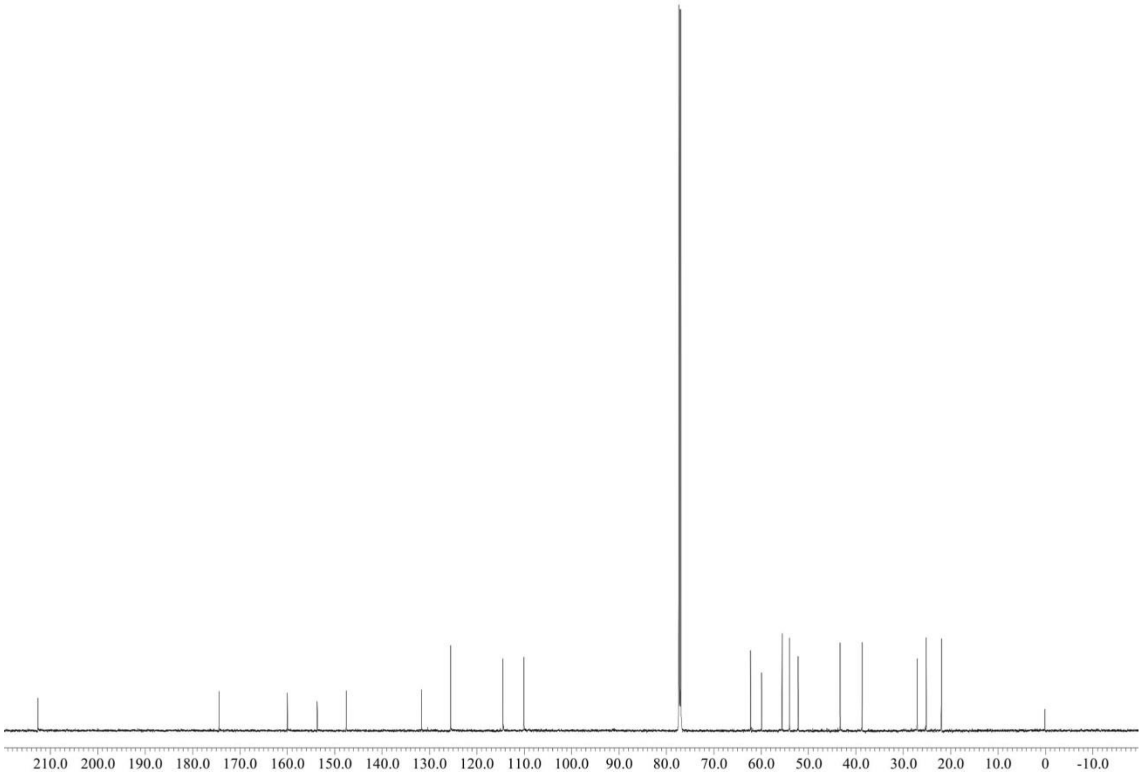

Supplementary Figure 95. ^{13}C NMR spectra of **5e** (major diastereomer).

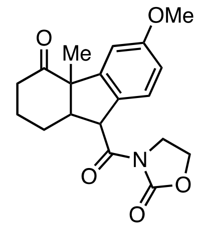

5e
(minor diastereomer)

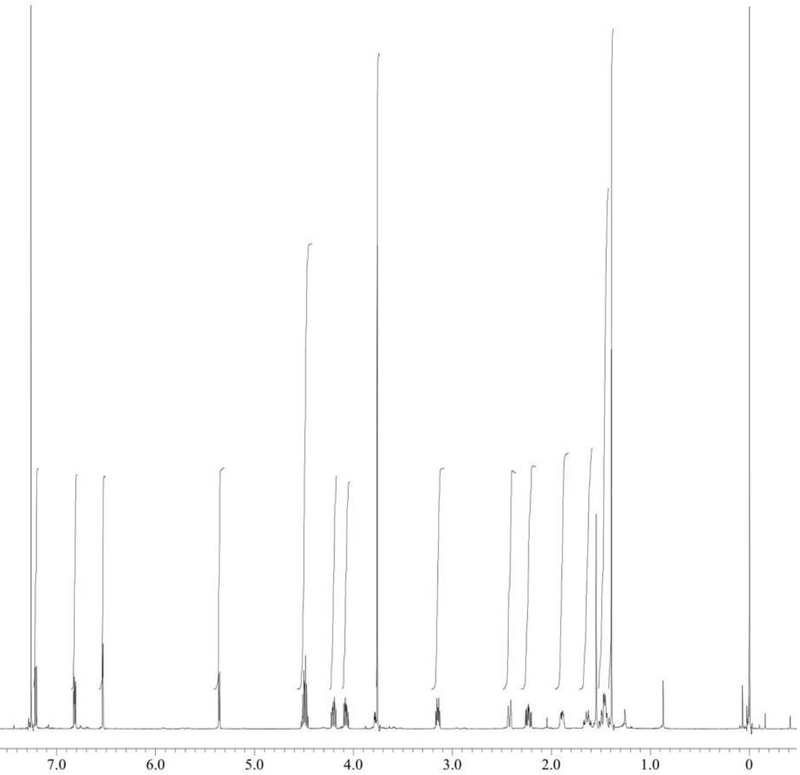

Supplementary Figure 96. ^{1}H NMR spectra of **5e** (minor diastereomer).

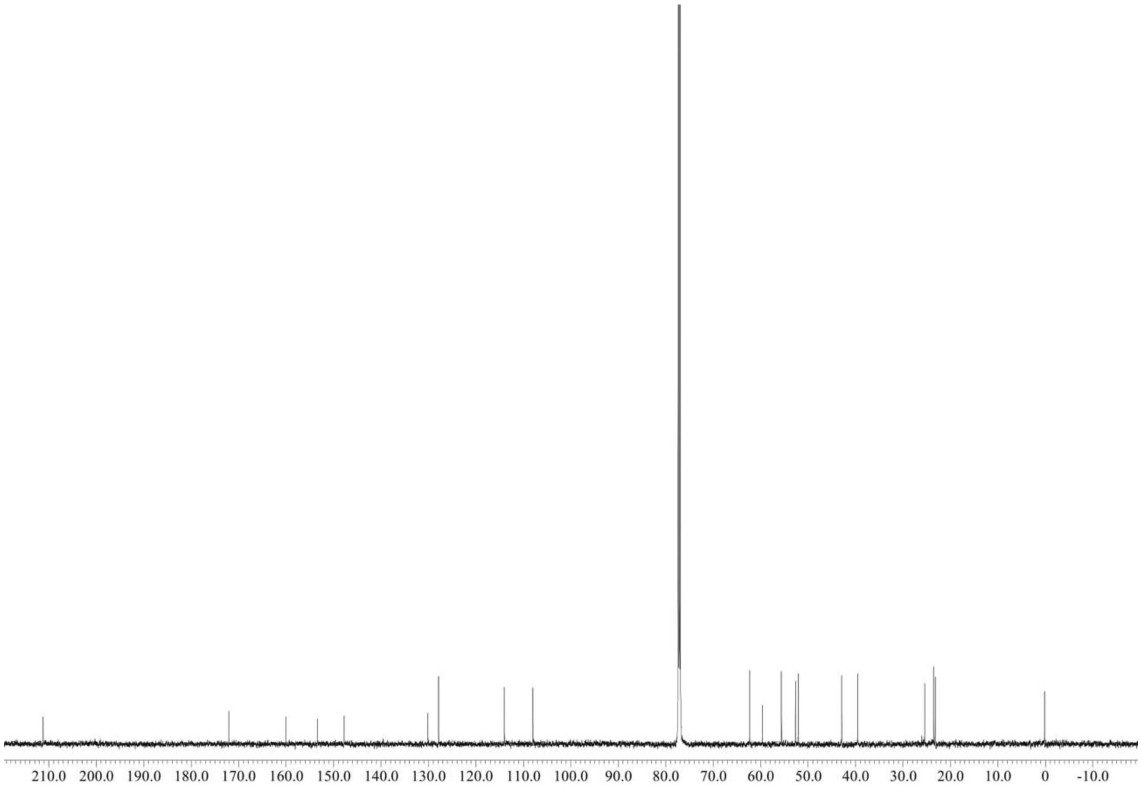

Supplementary Figure 97. ^{13}C NMR spectra of **5e** (minor diastereomer).

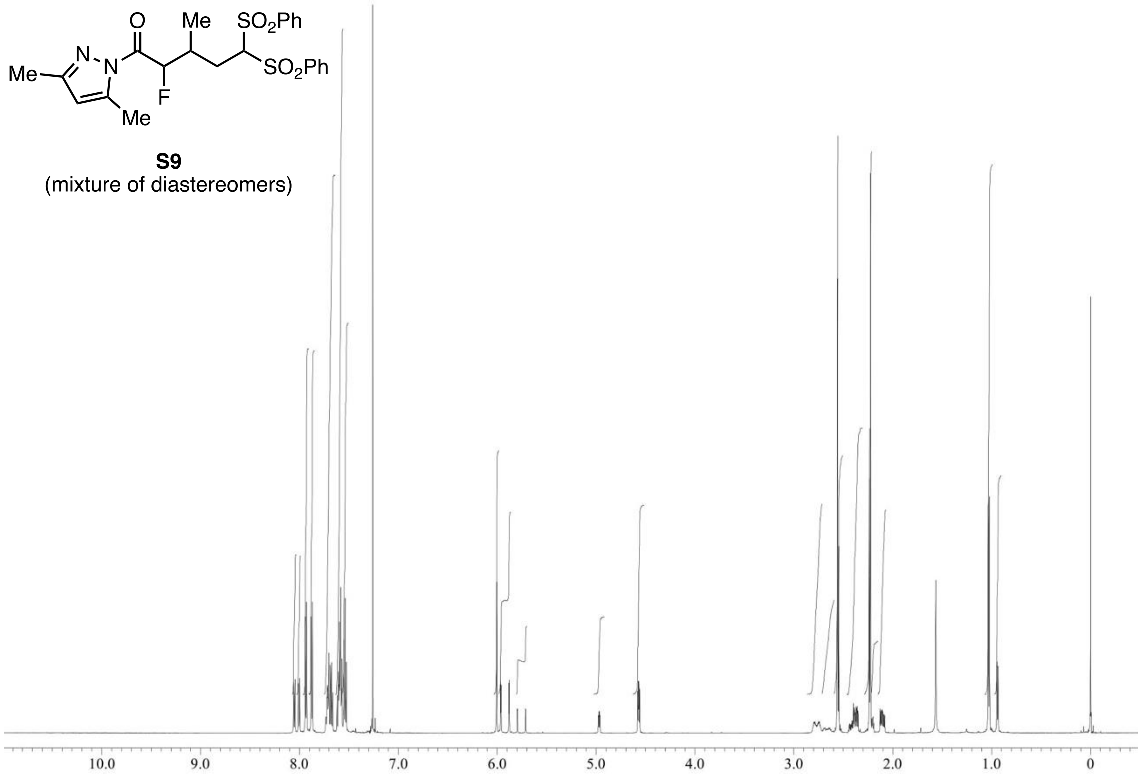

Supplementary Figure 98. ^{1}H NMR spectra of **S9** (mixture of diastereomers).

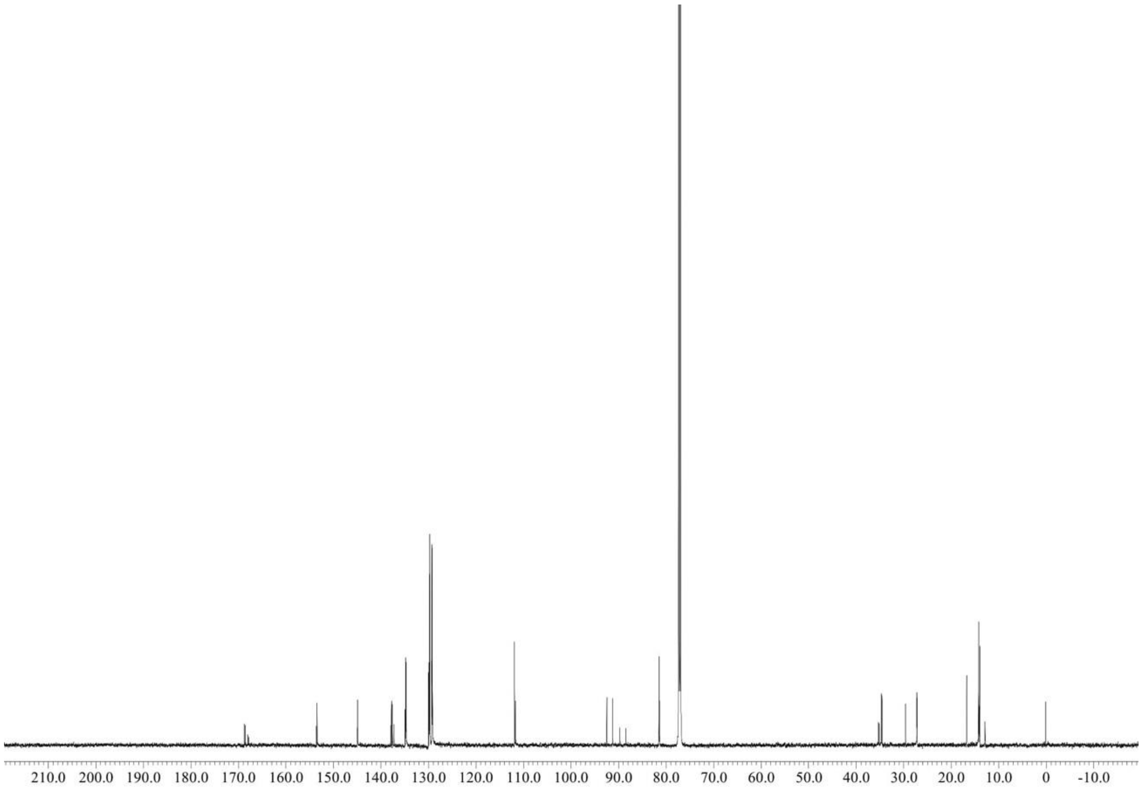

Supplementary Figure 99. ^{13}C NMR spectra of **S9** (mixture of diastereomers).

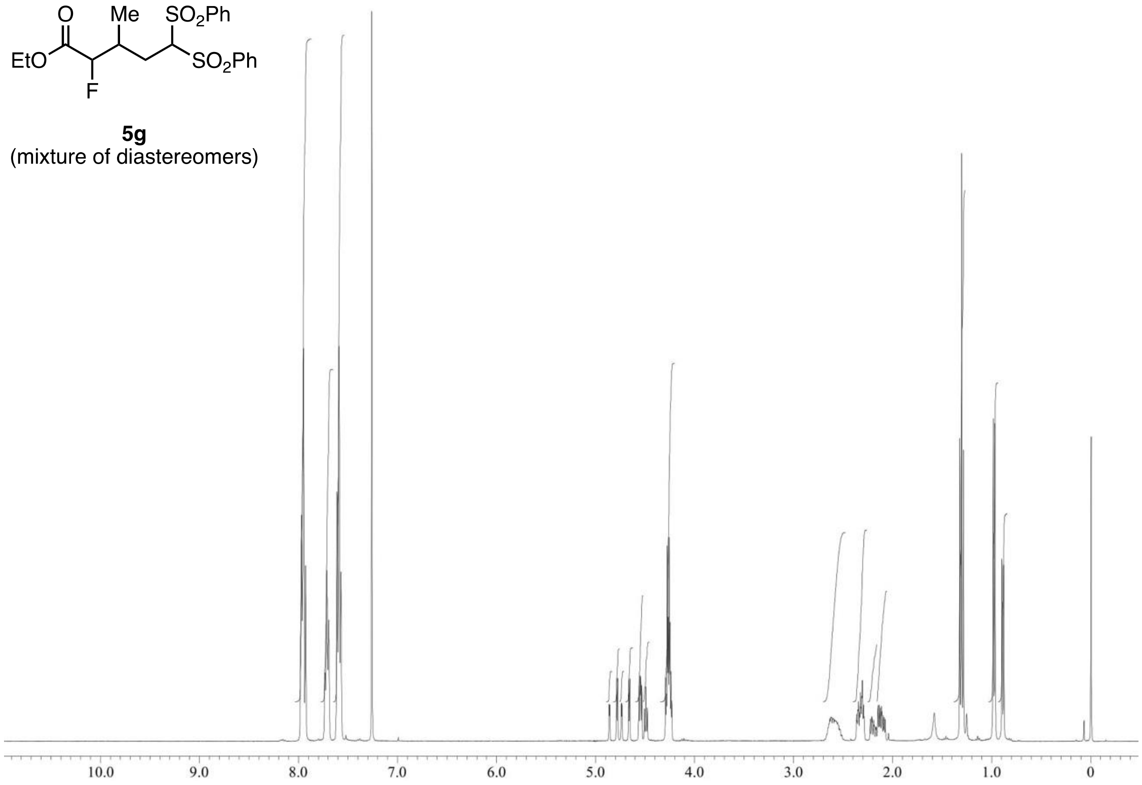

5g
(mixture of diastereomers)

Supplementary Figure 100. ^{1}H NMR spectra of **5g** (mixture of diastereomers).

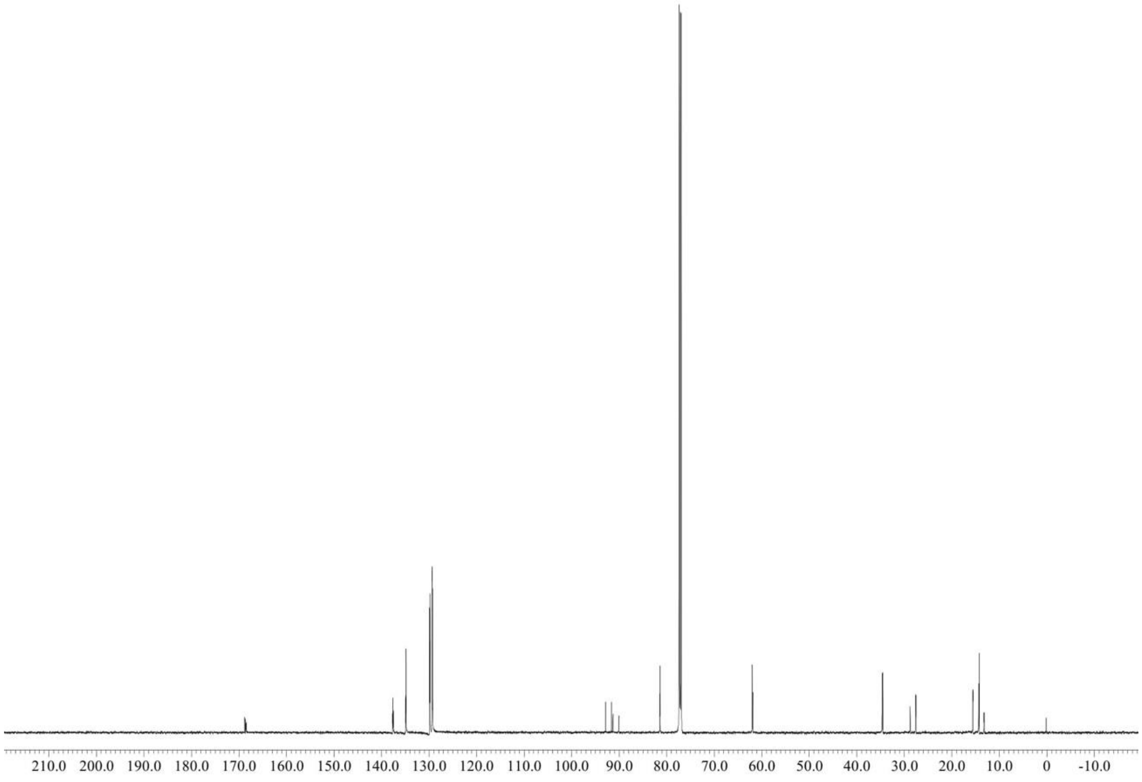

Supplementary Figure 101. ^{13}C NMR spectra of **5g** (mixture of diastereomers).

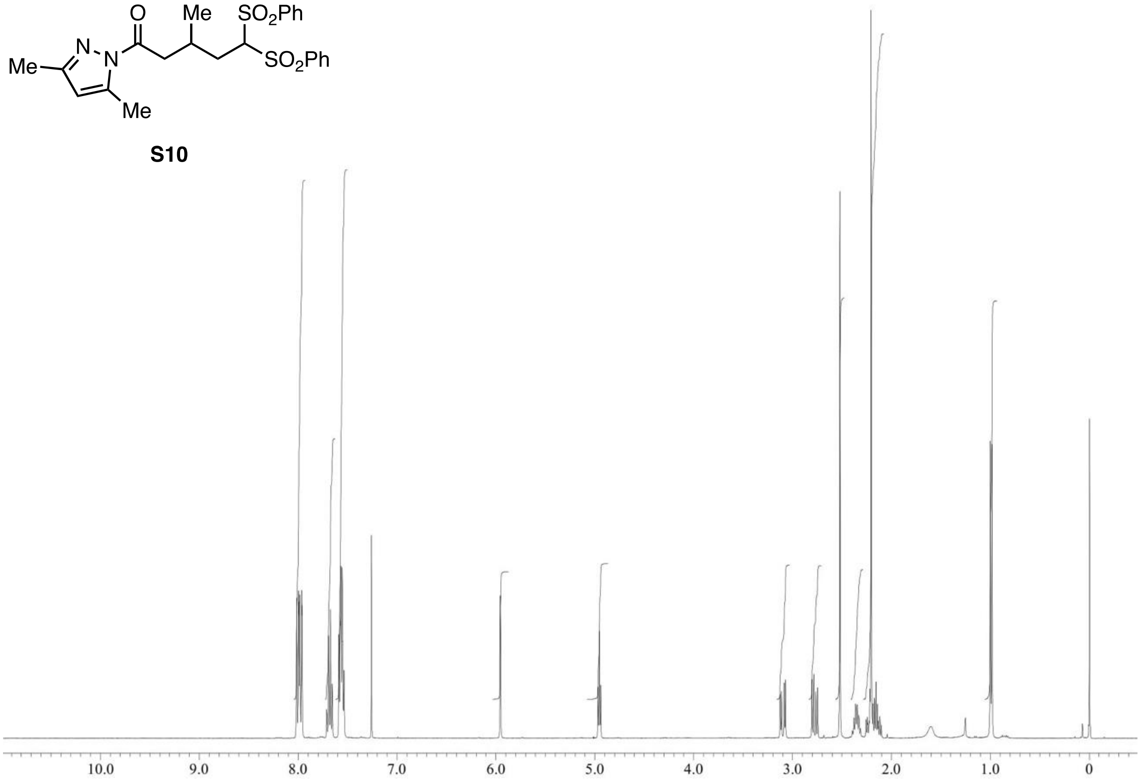

Supplementary Figure 102. ^{1}H NMR spectra of **S10**.

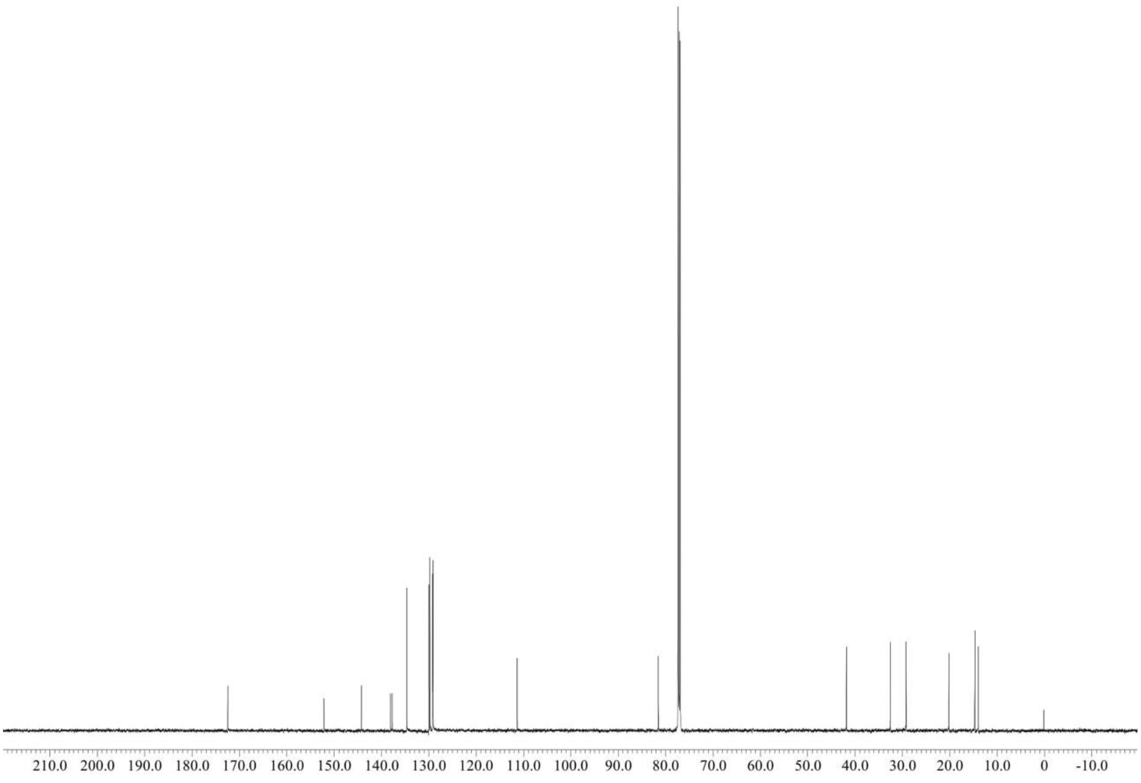

Supplementary Figure 103. ^{13}C NMR spectra of **S10**.

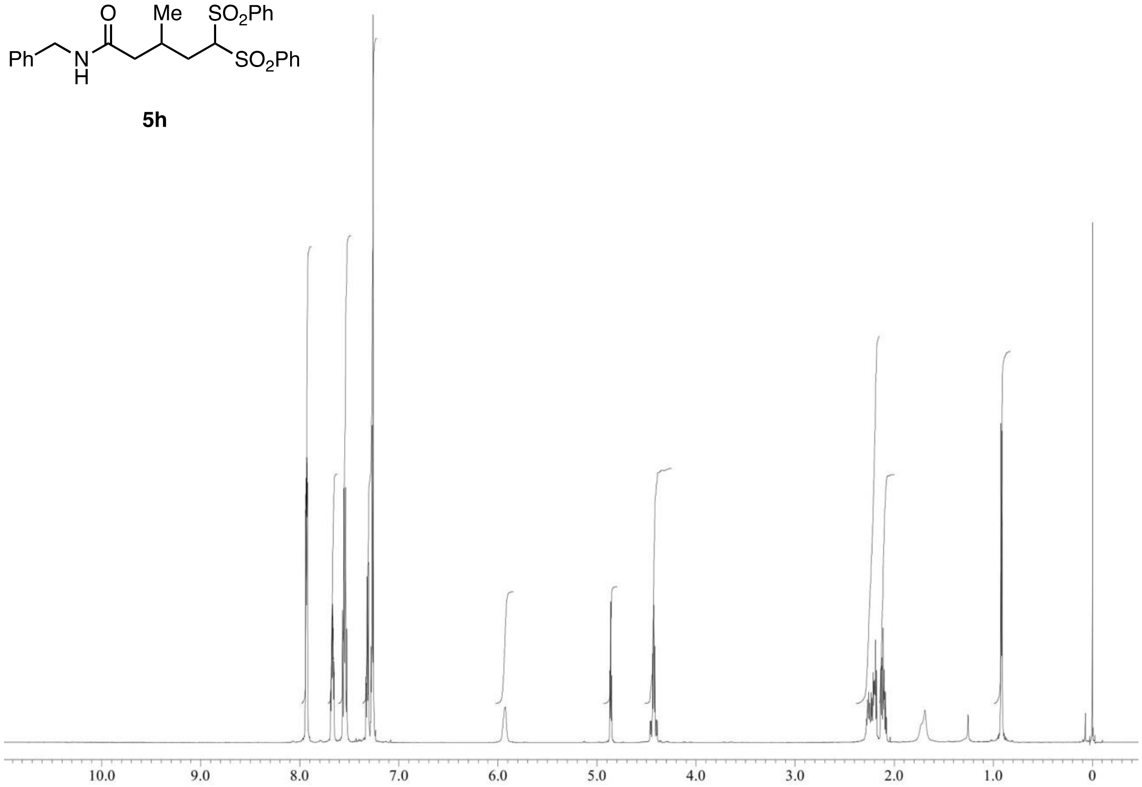

Supplementary Figure 104. ^{1}H NMR spectra of **5h**.

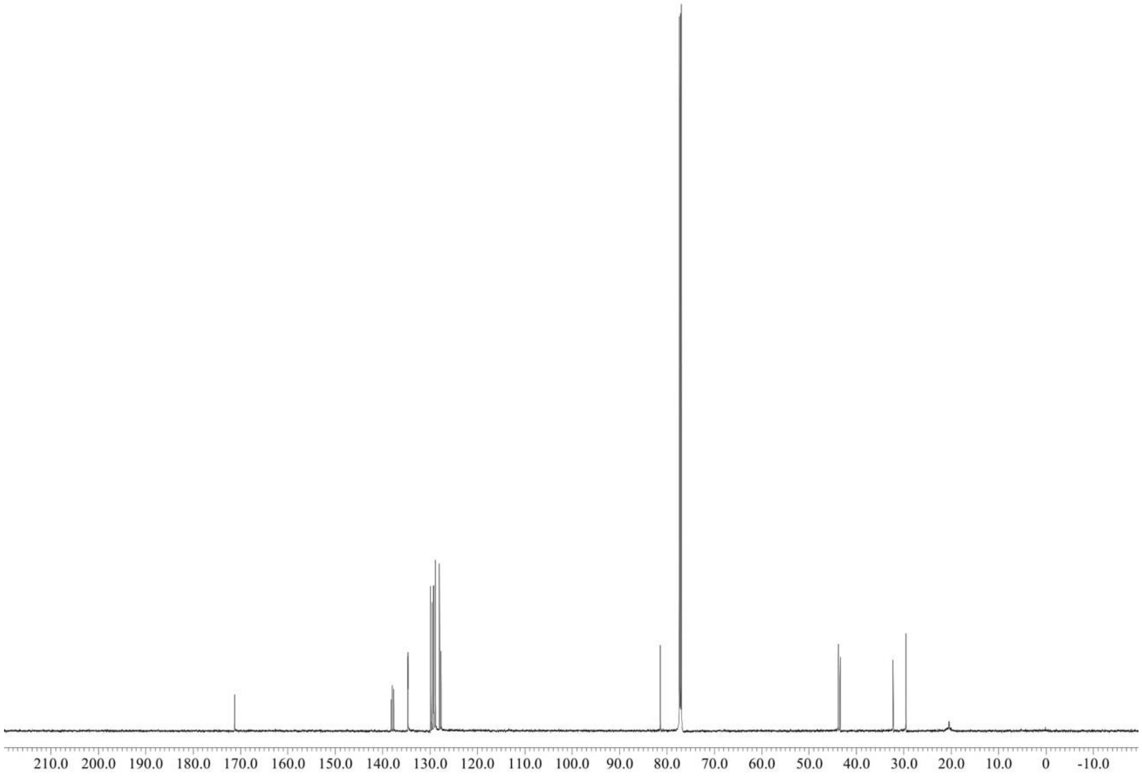

Supplementary Figure 105. ^{13}C NMR spectra of **5h**.

Supplementary References

1. Choi, G. J., Zhu, Q., Miller, D. C., Gu, C. J. & Knowles, R. R. Catalytic alkylation of remote C–H bonds enabled by proton-coupled electron transfer. *Nature* **539**, 268-271 (2016).
2. Lowry, M. S., Hudson, W. R., Pascal, R. A. Jr. & Bernhard, S. Accelerated luminophore discovery through combinatorial synthesis. *J. Am. Chem. Soc.* **126**, 14129-14135 (2004).
3. Sarabèr, F. C. E., Dratch, S., Bosselaar, G., Jansen, B. J. M. & de Groot, A. Domino Mukaiyama–Michael reactions in the synthesis of polycyclic systems. *Tetrahedron* **62**, 1717-1725 (2006).
4. Ito, H., Ohmiya, H. & Sawamura, M. Construction of methylenecycloheptane frameworks through 7-exo-dig cyclization of acetylenic silyl enol ethers catalyzed by triethynylphosphine−gold complex. *Org. Lett.* **12**, 4380-4383 (2010).
5. Sumida, Y., Yorimitsu, H. & Oshima, K. Palladium-catalyzed preparation of silyl enolates from α,β-unsaturated ketones or cyclopropyl ketones with hydrosilanes. *J. Org. Chem.* **74**, 7986-7989 (2009).
6. Gaussian 09, Revision D.01, Frisch, M. J.; Trucks, G. W.; Schlegel, H. B.; Scuseria, G. E.; Robb, M. A.; Cheeseman, J. R.; Scalmani, G.; Barone, V.; Mennucci, B.; Petersson, G. A.; Nakatsuji, H.; Caricato, M.; Li, X.; Hratchian, H. P.; Izmaylov, A. F.; Bloino, J.; Zheng, G.; Sonnenberg, J. L.; Hada, M.; Ehara, M.; Toyota, K.; Fukuda, R.; Hasegawa, J.; Ishida, M.; Nakajima, T.; Honda, Y.; Kitao, O.; Nakai, H.; Vreven, T.; Montgomery, Jr., J. A.; Peralta, J. E.; Ogliaro, F.; Bearpark, M.; Heyd, J. J.; Brothers, E.; Kudin, K. N.; Staroverov, V. N.; Keith, T.; Kobayashi, R.; Normand, J.; Raghavachari, K.; Rendell, A.; Burant, J. C.; Iyengar, S. S.; Tomasi, J.; Cossi, M.; Rega, N.; Millam, J. M.; Klene, M.; Knox, J. E.; Cross, J. B.; Bakken, V.; Adamo, C.; Jaramillo, J.; Gomperts, R.; Stratmann, R. E.; Yazyev, O.; Austin, A. J.; Cammi, R.; Pomelli, C.; Ochterski, J. W.; Martin, R. L.; Morokuma, K.; Zakrzewski, V. G.; Voth, G. A.; Salvador, P.; Dannenberg, J. J.; Dapprich, S.; Daniels, A. D.; Farkas, O.; Foresman, J. B.; Ortiz, J. V.; Cioslowski, J.; Fox, D. J. Gaussian, Inc., Wallingford CT, 2013.
7. Legault, C. Y. CYLview, 1.0b, Université de Sherbrooke, 2009.
8. Yanai, T., Tew, D. & Handy, N. A new hybrid exchange–correlation functional using the Coulomb-attenuating method (CAM-B3LYP). *Chem. Phys. Lett.* **393**, 51–57 (2004).
9. Marenich, A. V., Cramer, C. J. & Truhlar, D. G. Universal solvation model based on solute electron density and on a continuum model of the solvent defined by the bulk dielectric constant and atomic surface tensions. *J. Phys. Chem. B* **113**, 6378–6396 (2009).
10. Becke, A. D. Density-functional thermochemistry. III. The role of exact exchange. *J. Chem. Phys.* **98** 5648–5652 (1993).
11. Iikura, H., Tsuneda, T., Yanai, T. & Hirao, K. A long-range correction scheme for generalized-gradient-approximation exchange functionals. *J. Chem. Phys.* **115**, 3540–3544 (2001).
12. Riffet, V., Jacquemin, D., Cauët, E. & Frison, G. Benchmarking DFT and TD-DFT functionals for the ground and excited states of hydrogen-rich peptide radicals. *J. Chem. Theory Comput.* **10**, 3308−3318 (2014).
13. Kütt, A., Leito, I., Kaljurand, I., Sooväli, L., Vlasov, V. M., Yagupolskii, L. M. & Koppel, I. A. A comprehensive self-consistent spectrophotometric acidity scale of neutral Brønsted acids in acetonitrile. *J. Org. Chem.* **71**, 2829–2838 (2006).
14. Kütt, A., Rodima, T., Saame, J., Raamat, E., Mäemets, V., Kaljurand, I., Koppel, I. A., Garlyauskayte, R. Y., Yagupolskii, Y. L., Yagupolskii, L. M., Bernhardt, E., Willner, H. & Leito, I. Equilibrium acidities of superacids. *J. Org. Chem.* **76**, 391–395 (2011).
