# Supplementary material for: Direct allylic C–H alkylation of enol silyl ethers enabled by photoredox–Brønsted base hybrid catalysis
Source: Nat Commun. 2019 Jun 20;10:2706. doi: 10.1038/s41467-019-10641-y (PMC6586846; doi:10.1038/s41467-019-10641-y)
Supplement: Supplementary file 2 — Description of Additional Supplementary Files [file 41467_2019_10641_MOESM2_ESM.pdf]

Description of Additional Supplementary Files

File Name: Supplementary Data 1

Description: Cartesian Coordinates
