# Supplementary material for: Direct allylic C–H alkylation of enol silyl ethers enabled by photoredox–Brønsted base hybrid catalysis
Source: Nat Commun. 2019 Jun 20;10:2706. doi: 10.1038/s41467-019-10641-y (PMC6586846; doi:10.1038/s41467-019-10641-y)
Supplement: Supplementary file 3 — Supplementary Data 1 [file 41467_2019_10641_MOESM3_ESM.pdf]

Supplementary Data 1

Cartesian Coordinates

[**1a**]$^{\bullet+}$

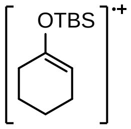

SMD(MeCN)-CAM-B3LYP/6-311+G(d,p)

E = -836.178439
Zero-point correction = 0.337261 (Hartree/Particle)
Thermal correction to Energy = 0.355667
Thermal correction to Enthalpy = 0.356611
Thermal correction to Gibbs Free Energy = 0.290805
Sum of electronic and zero-point Energies = -835.841178
Sum of electronic and thermal Energies = -835.822772
Sum of electronic and thermal Enthalpies = -835.821828
Sum of electronic and thermal Free Energies = -835.887634

O	0.182372	-0.371406	-0.005165
Si	-1.249740	0.680022	0.026894
C	-1.122744	1.739197	-1.492018
H	-2.048480	2.307168	-1.623153
H	-0.303931	2.458741	-1.416714
H	-0.971107	1.136455	-2.390333
C	-1.140657	1.623224	1.622092
H	-2.075046	2.165450	1.793926
H	-0.979026	0.957687	2.473228
H	-0.333149	2.359024	1.600849
C	-2.640369	-0.589100	-0.029847
C	2.096161	1.008622	0.043995
H	1.488100	1.901932	0.128788
C	1.458143	-0.252294	-0.008380
C	2.248114	-1.509410	-0.087994
C	3.701717	-1.330820	0.345576
C	3.559736	1.153694	0.031975
H	3.834542	1.485159	1.048360
C	4.319019	-0.117377	-0.338552
H	1.726997	-2.277455	0.487001
H	2.202885	-1.826157	-1.139196
H	4.257957	-2.237072	0.102395
H	3.746082	-1.205759	1.431768
H	5.367233	-0.006645	-0.057879
H	4.287576	-0.255584	-1.423548
H	3.823495	2.003933	-0.606228
C	-2.563284	-1.404468	-1.328336
H	-1.622733	-1.955618	-1.411595
H	-3.377371	-2.137242	-1.350243
H	-2.665393	-0.772745	-2.214580
C	-2.549315	-1.534808	1.176097
H	-3.366474	-2.263347	1.134880
H	-1.610348	-2.094459	1.188142

H	-2.635407	-0.997849	2.124544
C	-3.982868	0.160996	0.016056
H	-4.107340	0.836736	-0.834812
H	-4.805188	-0.561686	-0.017762
H	-4.098407	0.745539	0.932979

<u>SMD(MeCN)-B3LYP/6-311+G(d,p)</u>

E = -836.512677
Zero-point correction = 0.333587 (Hartree/Particle)
Thermal correction to Energy = 0.352370
Thermal correction to Enthalpy = 0.353314
Thermal correction to Gibbs Free Energy = 0.286217
Sum of electronic and zero-point Energies = -836.179090
Sum of electronic and thermal Energies = -836.160307
Sum of electronic and thermal Enthalpies = -836.159363
Sum of electronic and thermal Free Energies = -836.226460

O	0.187289	-0.372640	0.000730
Si	-1.257279	0.681349	0.039071
C	-1.125686	1.777295	-1.467101
H	-2.057184	2.338464	-1.595422
H	-0.313729	2.502755	-1.364286
H	-0.956089	1.195250	-2.377106
C	-1.150038	1.612337	1.653976
H	-2.070084	2.184938	1.810688
H	-1.025672	0.935913	2.503991
H	-0.316117	2.320155	1.650070
C	-2.659404	-0.595245	-0.041592
C	2.113456	1.010935	0.025082
H	1.506575	1.906548	0.098069
C	1.470815	-0.252455	-0.010581
C	2.262847	-1.515459	-0.081921
C	3.723292	-1.336755	0.353867
C	3.579929	1.156648	0.010163
H	3.855506	1.512456	1.020965

C	4.349435	-0.122945	-0.335314
H	1.742049	-2.282708	0.496803
H	2.220390	-1.841336	-1.132245
H	4.277792	-2.246284	0.113399
H	3.765901	-1.209655	1.441042
H	5.394231	-0.006818	-0.039719
H	4.334871	-0.271056	-1.420560
H	3.844625	1.998540	-0.642129
C	-2.579777	-1.398974	-1.356119
H	-1.643692	-1.959495	-1.438275
H	-3.402286	-2.123555	-1.394638
H	-2.667974	-0.755116	-2.236340
C	-2.580134	-1.560822	1.159114
H	-3.405236	-2.281560	1.106170
H	-1.645948	-2.130196	1.166927
H	-2.664781	-1.034617	2.114709
C	-4.004142	0.166482	0.007972
H	-4.120538	0.855761	-0.834244
H	-4.832074	-0.551002	-0.039745
H	-4.120503	0.738611	0.933767

SMD(MeCN)-LC-BLYP/6-311+G(d,p)

E = -834.627552
Zero-point correction = 0.341890 (Hartree/Particle)
Thermal correction to Energy = 0.359975
Thermal correction to Enthalpy = 0.360919
Thermal correction to Gibbs Free Energy = 0.295962
Sum of electronic and zero-point Energies = -834.285662
Sum of electronic and thermal Energies = -834.267577
Sum of electronic and thermal Enthalpies = -834.266633
Sum of electronic and thermal Free Energies = -834.331589

O	0.177573	-0.368950	-0.007457
Si	-1.242217	0.674503	0.027164
C	-1.117591	1.724317	-1.479384
H	-2.045270	2.285488	-1.615490
H	-0.304665	2.448886	-1.405525
H	-0.960319	1.119808	-2.374198
C	-1.136021	1.600511	1.614582
H	-2.076503	2.125454	1.798653
H	-0.959072	0.929565	2.457036
H	-0.341319	2.348305	1.596487
C	-2.617083	-0.583056	-0.031325
C	2.075954	1.004553	0.047708
H	1.466123	1.896946	0.135177
C	1.444645	-0.251695	-0.006445
C	2.231284	-1.499851	-0.082863
C	3.674657	-1.316435	0.344459
C	3.532586	1.149278	0.033811
H	3.808322	1.469515	1.050971
C	4.280438	-0.113442	-0.346439
H	1.710587	-2.264285	0.495399
H	2.182429	-1.817206	-1.132055
H	4.233367	-2.221815	0.108653

H	3.721622	-1.181567	1.428462
H	5.332286	-0.004239	-0.083324
H	4.231906	-0.253943	-1.429580
H	3.794775	2.001271	-0.599592
C	-2.529718	-1.393984	-1.318170
H	-1.585194	-1.937454	-1.395964
H	-3.337290	-2.131874	-1.342627
H	-2.632353	-0.764635	-2.204952
C	-2.523712	-1.517425	1.168583
H	-3.334271	-2.251338	1.127526
H	-1.581045	-2.069154	1.184531
H	-2.616220	-0.978445	2.114279
C	-3.951887	0.156462	0.007887
H	-4.075989	0.828271	-0.844999
H	-4.771070	-0.567836	-0.025597
H	-4.071698	0.743448	0.921661

[1a-H]$^{\bullet}$

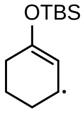

SMD(MeCN)-CAM-B3LYP/6-311+G(d,p)

E = -835.738437
Zero-point correction = 0.323989 (Hartree/Particle)
Thermal correction to Energy = 0.342274
Thermal correction to Enthalpy = 0.343218
Thermal correction to Gibbs Free Energy = 0.276740
Sum of electronic and zero-point Energies = -835.414448
Sum of electronic and thermal Energies = -835.396163
Sum of electronic and thermal Enthalpies = -835.395219
Sum of electronic and thermal Free Energies = -835.461696

O	0.186288	-0.408740	-0.008075
Si	-1.159846	0.621028	0.029024
C	-1.121306	1.744846	-1.463929
H	-2.033156	2.347345	-1.513126
H	-0.275908	2.436247	-1.424663
H	-1.047389	1.175532	-2.394202
C	-1.134751	1.612118	1.613858
H	-2.083968	2.138052	1.752822
H	-0.976847	0.971242	2.485045
H	-0.342800	2.365230	1.602166
C	-2.625006	-0.580895	-0.026626
C	2.136764	1.025024	0.047650
H	1.523428	1.912282	0.164047
C	1.525258	-0.215088	-0.029983
C	2.328505	-1.470410	-0.166860
C	3.754587	-1.285742	0.355923
C	3.514213	1.170482	-0.036754
H	3.952094	2.161285	-0.001556
C	4.403539	-0.022955	-0.215516
H	1.821058	-2.274826	0.372902
H	2.350297	-1.773474	-1.222814
H	4.356228	-2.165886	0.117494
H	3.722943	-1.204200	1.447306
H	5.374420	0.150393	0.257224
H	4.613348	-0.176769	-1.284322
C	-2.546576	-1.452043	-1.287915
H	-1.628235	-2.043962	-1.315950
H	-3.392338	-2.149436	-1.314654
H	-2.587876	-0.853079	-2.202257
C	-3.940142	0.213710	-0.050289
H	-4.791713	-0.476305	-0.070220
H	-4.055346	0.847655	0.833550
H	-4.018221	0.852481	-0.934627
C	-2.609696	-1.486784	1.212937
H	-2.707116	-0.913346	2.139128
H	-3.448668	-2.191700	1.173370
H	-1.689040	-2.072815	1.276631

SMD(MeCN)-B3LYP/6-311+G(d,p)

E = -836.069976
Zero-point correction = 0.320483 (Hartree/Particle)
Thermal correction to Energy = 0.339130
Thermal correction to Enthalpy = 0.340074
Thermal correction to Gibbs Free Energy = 0.272430
Sum of electronic and zero-point Energies = -835.749493
Sum of electronic and thermal Energies = -835.730847
Sum of electronic and thermal Enthalpies = -835.729902
Sum of electronic and thermal Free Energies = -835.797546

O	-0.190502	-0.402794	-0.062986
Si	1.167983	0.628320	-0.029823
C	1.084241	1.716681	1.500540
H	2.018712	2.274130	1.624428
H	0.274057	2.447927	1.425006
H	0.922579	1.126990	2.407896
C	1.193522	1.679931	-1.587647
H	2.108990	2.279497	-1.630261
H	1.151988	1.065698	-2.492226
H	0.346677	2.372494	-1.612210
C	2.638421	-0.590218	0.033172
C	-2.156803	1.029040	-0.064498
H	-1.548484	1.919692	-0.187691
C	-1.534571	-0.213409	-0.008801
C	-2.333282	-1.477077	0.134244
C	-3.780198	-1.294196	-0.352146
C	-3.537289	1.171099	0.048377
H	-3.977946	2.162064	0.028313
C	-4.423699	-0.028029	0.236728
H	-1.838514	-2.276935	-0.426759
H	-2.327238	-1.794016	1.188186
H	-4.372800	-2.177603	-0.097838
H	-3.777278	-1.212943	-1.445170
H	-5.403977	0.144793	-0.220485
H	-4.619254	-0.187287	1.309482
C	2.573667	-1.436231	1.321659
H	1.652602	-2.024165	1.376913
H	3.416995	-2.138448	1.352456
H	2.631733	-0.816464	2.222063
C	3.962301	0.204920	0.020293
H	4.813359	-0.486783	0.064142
H	4.074878	0.802428	-0.889962
H	4.047173	0.879760	0.878198
C	2.606103	-1.529862	-1.190065
H	2.680378	-0.978493	-2.132825
H	3.453797	-2.226420	-1.150621
H	1.689361	-2.125938	-1.220762

SMD(MeCN)-LC-BLYP/6-311+G(d,p)
E = -834.192332
Zero-point correction = 0.328356 (Hartree/Particle)
Thermal correction to Energy = 0.346367
Thermal correction to Enthalpy = 0.347311
Thermal correction to Gibbs Free Energy = 0.281633

Sum of electronic and zero-point Energies = -833.863976
Sum of electronic and thermal Energies = -833.845965
Sum of electronic and thermal Enthalpies = -833.845021
Sum of electronic and thermal Free Energies = -833.910699

O	0.182920	-0.413888	-0.008004
Si	-1.152244	0.611164	0.029711
C	-1.111568	1.724485	-1.451551
H	-2.020899	2.328357	-1.499534
H	-0.265093	2.412579	-1.415400
H	-1.041831	1.152958	-2.379267
C	-1.127758	1.588666	1.605089
H	-2.083056	2.098135	1.753202
H	-0.955146	0.944713	2.469555
H	-0.348045	2.352339	1.592806
C	-2.606478	-0.572918	-0.027933
C	2.113766	1.018717	0.050105
H	1.496011	1.902443	0.167475
C	1.514297	-0.217689	-0.027937
C	2.318702	-1.461785	-0.164853
C	3.731772	-1.269300	0.357606
C	3.483317	1.168728	-0.037330
H	3.918471	2.160327	-0.004202
C	4.369513	-0.016864	-0.220061
H	1.812116	-2.266542	0.372405
H	2.342698	-1.760651	-1.220230
H	4.338275	-2.147411	0.129776
H	3.696395	-1.177755	1.446950
H	5.343234	0.160576	0.241645
H	4.566583	-0.171836	-1.289211
C	-2.514139	-1.450472	-1.269558
H	-1.596077	-2.041588	-1.278747
H	-3.359083	-2.146466	-1.299792
H	-2.542946	-0.861050	-2.189442
C	-3.908345	0.219856	-0.074031
H	-4.761439	-0.466117	-0.091696
H	-4.028307	0.866234	0.799003
H	-3.975266	0.845732	-0.967255
C	-2.602718	-1.457141	1.212916
H	-2.712893	-0.873313	2.129911
H	-3.437099	-2.165209	1.171902
H	-1.680706	-2.037353	1.293454

[**1o**]$^{\bullet+}$

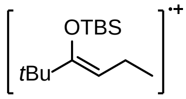

SMD(MeCN)-CAM-B3LYP/6-311+G(d,p)

E = -915.967760
Zero-point correction = 0.412084 (Hartree/Particle)
Thermal correction to Energy = 0.435736
Thermal correction to Enthalpy = 0.436680
Thermal correction to Gibbs Free Energy = 0.358766

Sum of electronic and zero-point Energies = -915.555676
Sum of electronic and thermal Energies = -915.532023
Sum of electronic and thermal Enthalpies = -915.531079
Sum of electronic and thermal Free Energies = -915.608993

C	1.230196	2.525194	0.222698
C	2.024894	1.298513	0.062941
H	3.101168	1.395446	-0.000306
C	1.495917	-0.015239	0.010715
O	0.242223	-0.204632	0.156175
C	1.928272	3.773130	-0.309365
H	2.091103	3.701726	-1.386799
H	1.317258	4.656280	-0.117463
H	2.896809	3.918829	0.173643
Si	-1.396273	-0.192185	0.773077
C	-1.563103	1.245843	1.934669
H	-2.504777	1.141736	2.482737
H	-0.757056	1.251434	2.672833
H	-1.576147	2.208508	1.422165
C	-1.545080	-1.808129	1.673037
H	-2.565105	-1.922069	2.053013
H	-1.327958	-2.663357	1.030607
H	-0.871084	-1.838401	2.532939
C	-2.467178	-0.063219	-0.775882
H	0.239037	2.403564	-0.217562
H	1.054187	2.638353	1.305436
C	-3.925059	0.081581	-0.302563
H	-4.587500	0.132661	-1.173384
H	-4.250122	-0.768472	0.304411
H	-4.078404	0.993827	0.280497
C	-2.087560	1.165516	-1.613064
H	-2.173666	2.095056	-1.044148
H	-1.068086	1.097346	-2.001471
H	-2.761453	1.244183	-2.473257
C	-2.339528	-1.326273	-1.638276
H	-1.319446	-1.474148	-2.001388
H	-2.638933	-2.226057	-1.094852
H	-2.991451	-1.238487	-2.514382
C	2.356905	-1.243128	-0.198398
C	2.473431	-1.931528	1.178211
C	1.661135	-2.184861	-1.191397
C	3.756177	-0.900500	-0.715776
H	2.941238	-1.273510	1.914091
H	1.497360	-2.241883	1.554325
H	3.098240	-2.820508	1.069653
H	1.544810	-1.710558	-2.168735
H	2.276661	-3.077253	-1.317889
H	0.679659	-2.496320	-0.835453
H	4.293470	-1.831700	-0.904210
H	3.716006	-0.340575	-1.653036
H	4.337700	-0.329393	0.010210

<u>SMD(MeCN)-B3LYP/6-311+G(d,p)</u>
E = -916.358961

Zero-point correction = 0.408614 (Hartree/Particle)
Thermal correction to Energy = 0.432298
Thermal correction to Enthalpy = 0.433242
Thermal correction to Gibbs Free Energy = 0.356073
Sum of electronic and zero-point Energies = -915.950348
Sum of electronic and thermal Energies = -915.926663
Sum of electronic and thermal Enthalpies = -915.925719
Sum of electronic and thermal Free Energies = -916.002889

C	1.216498	2.516509	0.262976
C	1.996511	1.273151	0.117812
H	3.075058	1.365075	0.053300
C	1.476279	-0.049368	0.066076
O	0.223292	-0.276128	0.214515
C	1.915792	3.746226	-0.328570
H	2.046627	3.640884	-1.408656
H	1.317157	4.641035	-0.144971
H	2.899922	3.896477	0.122892
Si	-1.442653	-0.195877	0.787136
C	-1.615270	1.264040	1.937421
H	-2.568079	1.168997	2.470163
H	-0.821540	1.266432	2.690372
H	-1.612358	2.225318	1.420688
C	-1.647008	-1.802061	1.715246
H	-2.665578	-1.868133	2.112691
H	-1.473623	-2.673469	1.080409
H	-0.958695	-1.847930	2.564752
C	-2.480280	-0.074090	-0.805399
H	0.211053	2.397817	-0.145952
H	1.074187	2.672507	1.347157
C	-3.952226	0.113224	-0.366427
H	-4.592578	0.163780	-1.255296
H	-4.312226	-0.718775	0.247278
H	-4.099299	1.040095	0.196440
C	-2.052757	1.133064	-1.663470
H	-2.130453	2.077226	-1.116432
H	-1.026522	1.034684	-2.029060
H	-2.706745	1.207716	-2.540904
C	-2.359601	-1.364907	-1.640043
H	-1.332498	-1.547251	-1.969534
H	-2.698136	-2.245720	-1.086971
H	-2.982876	-1.280359	-2.538559
C	2.399351	-1.232865	-0.188254
C	3.419345	-1.307675	0.979079
C	1.608859	-2.546245	-0.257706
C	3.149311	-1.012395	-1.525926
H	4.050818	-0.418777	1.036750
H	2.911687	-1.437795	1.938353
H	4.067971	-2.172133	0.813822
H	0.869984	-2.530664	-1.061973
H	2.304615	-3.365679	-0.452697
H	1.093082	-2.757591	0.680969
H	3.803418	-1.870216	-1.703150
H	2.447648	-0.937800	-2.360731

H	3.769973	-0.114097	-1.514060

SMD(MeCN)-LC-BLYP/6-311+G(d,p)

E = -914.207235
Zero-point correction = 0.417865 (Hartree/Particle)
Thermal correction to Energy = 0.440876
Thermal correction to Enthalpy = 0.441820
Thermal correction to Gibbs Free Energy = 0.366400
Sum of electronic and zero-point Energies = -913.789370
Sum of electronic and thermal Energies = -913.766359
Sum of electronic and thermal Enthalpies = -913.765415
Sum of electronic and thermal Free Energies = -913.840835

C	1.255368	2.497504	0.187077
C	2.039298	1.278346	-0.014779
H	3.108878	1.363002	-0.160533
C	1.487429	-0.018218	0.006471
O	0.246771	-0.158681	0.216615
C	1.965577	3.754525	-0.267567
H	2.161154	3.727947	-1.340518
H	1.356023	4.633387	-0.058404
H	2.919321	3.874721	0.248527
Si	-1.394531	-0.166535	0.779701
C	-1.586936	1.282199	1.898461
H	-2.544967	1.204473	2.418805
H	-0.803932	1.296354	2.659204
H	-1.569659	2.231205	1.362259
C	-1.561706	-1.765351	1.674051
H	-2.605070	-1.921833	1.960300
H	-1.252955	-2.610805	1.058033
H	-0.967006	-1.767219	2.589657
C	-2.410295	-0.056514	-0.784477
H	0.276584	2.396165	-0.286924
H	1.041250	2.553922	1.264811
C	-3.852116	0.223916	-0.365425
H	-4.491130	0.254097	-1.252939
H	-4.250805	-0.551044	0.294767
H	-3.951826	1.185169	0.143776
C	-1.919016	1.076318	-1.676257
H	-1.932785	2.040520	-1.162403
H	-0.904042	0.899086	-2.039019
H	-2.569655	1.162515	-2.551856
C	-2.359171	-1.369801	-1.554651
H	-1.344125	-1.623095	-1.868163
H	-2.748283	-2.203110	-0.965550
H	-2.971690	-1.289873	-2.457892
C	2.297298	-1.269835	-0.180888
C	2.391468	-1.928567	1.196132
C	1.561319	-2.192812	-1.143714
C	3.692086	-0.985486	-0.709670
H	2.889562	-1.277098	1.916672
H	1.405138	-2.189112	1.581861
H	2.975601	-2.845580	1.106236
H	1.442227	-1.728576	-2.124691

H	2.144090	-3.106043	-1.268705
H	0.577540	-2.467289	-0.765771
H	4.202432	-1.935501	-0.870893
H	3.662981	-0.455043	-1.663204
H	4.290805	-0.407741	-0.004761

[**1o-H**]$^{\bullet}$

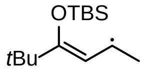

<u>CAM-B3LYP/6-311+G(d,p)</u>

E = -915.521204
Zero-point correction = 0.400996 (Hartree/Particle)
Thermal correction to Energy = 0.424006
Thermal correction to Enthalpy = 0.424950
Thermal correction to Gibbs Free Energy = 0.349546
Sum of electronic and zero-point Energies = -915.120208
Sum of electronic and thermal Energies = -915.097198
Sum of electronic and thermal Enthalpies = -915.096254
Sum of electronic and thermal Free Energies = -915.171658

C	1.079287	2.516280	-0.109012
C	1.895723	1.412034	-0.001628
H	2.953488	1.598451	0.141882
C	1.517770	0.077390	-0.078441
O	0.205847	-0.255581	-0.240811
C	2.477689	-1.093165	-0.200556
C	1.570183	3.921340	0.009533
H	1.351117	4.502172	-0.893181
H	1.088172	4.448978	0.840779
H	2.648909	3.956755	0.176183
Si	-1.210594	-0.304955	0.660964
C	-1.226826	1.015513	1.988969
H	-2.022670	0.807658	2.709478
H	-0.280068	1.020161	2.534719
H	-1.381815	2.020352	1.595256
C	-1.368723	-1.972588	1.503507
H	-2.352101	-2.064893	1.972707
H	-1.254219	-2.803270	0.804455
H	-0.621438	-2.090231	2.291122
C	-2.594714	-0.079032	-0.619103
H	0.025228	2.377770	-0.313310
C	2.185507	-2.139202	0.887109
H	2.293998	-1.709495	1.886467
H	1.177557	-2.539397	0.791216
H	2.885467	-2.974482	0.801884
C	2.278069	-1.743069	-1.582659
H	2.518813	-1.038457	-2.382142
H	2.929229	-2.615047	-1.688180
H	1.245198	-2.066321	-1.714656
C	3.939777	-0.655697	-0.070385
H	4.588118	-1.529663	-0.165329
H	4.223562	0.052389	-0.851302
H	4.141712	-0.198084	0.900922
C	-3.957847	-0.072880	0.090675

H	-4.761687	0.057042	-0.642451
H	-4.151923	-1.009644	0.619795
H	-4.041409	0.744861	0.812011
C	-2.419297	1.245319	-1.375085
H	-2.468656	2.109528	-0.706623
H	-1.466290	1.280842	-1.906883
H	-3.218923	1.363299	-2.115190
C	-2.556909	-1.233641	-1.631707
H	-2.727703	-2.202297	-1.154880
H	-3.340453	-1.098828	-2.386026
H	-1.597806	-1.277745	-2.153023

<u>SMD(MeCN)-CAM-B3LYP/6-311+G(d,p)</u>

E = -915.531699
Zero-point correction = 0.399375 (Hartree/Particle)
Thermal correction to Energy = 0.422253
Thermal correction to Enthalpy = 0.423197
Thermal correction to Gibbs Free Energy = 0.348702
Sum of electronic and zero-point Energies = -915.132324
Sum of electronic and thermal Energies = -915.109446
Sum of electronic and thermal Enthalpies = -915.108502
Sum of electronic and thermal Free Energies = -915.182996

C	1.036628	2.527247	-0.077975
C	1.876181	1.434104	-0.010264
H	2.932986	1.637979	0.119047
C	1.520767	0.093029	-0.107038
O	0.216362	-0.258594	-0.279605
C	2.506521	-1.059018	-0.210665
C	1.510121	3.937662	0.049969
H	1.262100	4.528759	-0.839555
H	1.034360	4.447970	0.896186
H	2.591860	3.987580	0.195136
Si	-1.199098	-0.366768	0.632527
C	-1.183358	0.859755	2.045186
H	-1.972675	0.601872	2.758127
H	-0.232700	0.816042	2.584757
H	-1.342568	1.890496	1.724081
C	-1.355649	-2.087277	1.353055
H	-2.361519	-2.222120	1.763303
H	-1.193649	-2.870529	0.609136
H	-0.648200	-2.243310	2.171198
C	-2.611123	-0.049337	-0.598753
H	-0.023372	2.377081	-0.242134
C	2.243549	-2.084417	0.904099
H	2.326660	-1.624614	1.892812
H	1.253008	-2.528374	0.812856
H	2.978059	-2.892537	0.845826
C	2.318014	-1.748688	-1.573771
H	2.530808	-1.059101	-2.395456
H	2.998681	-2.600216	-1.661407
H	1.297061	-2.114572	-1.691867
C	3.959160	-0.586926	-0.098939
H	4.624106	-1.449760	-0.185169

H	4.222822	0.116009	-0.892215
H	4.159615	-0.109948	0.863624
C	-3.938972	0.012354	0.173648
H	-4.769389	0.171256	-0.524301
H	-4.144876	-0.913388	0.718499
H	-3.955853	0.835963	0.893272
C	-2.411986	1.276518	-1.344078
H	-2.373745	2.130245	-0.661669
H	-1.492856	1.276306	-1.934529
H	-3.248683	1.447333	-2.032194
C	-2.681443	-1.190324	-1.624459
H	-2.874315	-2.156452	-1.150483
H	-3.495879	-1.006430	-2.335336
H	-1.755823	-1.278119	-2.200163

SMD(DCE)-CAM-B3LYP/6-311+G(d,p)

E = -915.532276
Zero-point correction = 0.399633 (Hartree/Particle)
Thermal correction to Energy = 0.422513
Thermal correction to Enthalpy = 0.423457
Thermal correction to Gibbs Free Energy = 0.348965
Sum of electronic and zero-point Energies = -915.132642
Sum of electronic and thermal Energies = -915.109763
Sum of electronic and thermal Enthalpies = -915.108818
Sum of electronic and thermal Free Energies = -915.183310

C	1.037128	2.526129	-0.078261
C	1.876704	1.433668	-0.008467
H	2.933465	1.637589	0.120499
C	1.521542	0.092684	-0.103422
O	0.216771	-0.259784	-0.273613
C	2.506537	-1.059629	-0.210238
C	1.508714	3.937190	0.047075
H	1.260787	4.525935	-0.843982
H	1.032459	4.448751	0.892232
H	2.590276	3.989084	0.192589
Si	-1.199908	-0.362902	0.634835
C	-1.189247	0.868045	2.043981
H	-1.975299	0.608205	2.759798
H	-0.237336	0.831673	2.581730
H	-1.353630	1.897803	1.722577
C	-1.357551	-2.080530	1.362937
H	-2.362743	-2.215094	1.774868
H	-1.196094	-2.867452	0.622809
H	-0.650150	-2.234098	2.181583
C	-2.609484	-0.052152	-0.601373
H	-0.022067	2.374308	-0.245364
C	2.243174	-2.087749	0.901909
H	2.327879	-1.630966	1.891863
H	1.251941	-2.530028	0.810284
H	2.975931	-2.897280	0.841556
C	2.316827	-1.745134	-1.575316
H	2.529362	-1.053250	-2.394950
H	2.996284	-2.597294	-1.666505

H	1.295445	-2.109335	-1.694200
C	3.959474	-0.588629	-0.097640
H	4.624603	-1.451161	-0.185405
H	4.223749	0.115244	-0.889787
H	4.160508	-0.113667	0.865778
C	-3.939820	0.012168	0.166381
H	-4.768496	0.168031	-0.534223
H	-4.147974	-0.911259	0.714301
H	-3.959929	0.838532	0.882785
C	-2.408666	1.270094	-1.352705
H	-2.378593	2.128230	-0.675381
H	-1.485448	1.269293	-1.936556
H	-3.240697	1.435304	-2.047734
C	-2.674877	-1.197880	-1.622175
H	-2.867702	-2.162381	-1.144892
H	-3.487276	-1.019087	-2.336583
H	-1.747433	-1.286375	-2.194602

SMD(MeCN)-B3LYP/6-311+G(d,p)

E = -915.918922
Zero-point correction = 0.395352 (Hartree/Particle)
Thermal correction to Energy = 0.418564
Thermal correction to Enthalpy = 0.419508
Thermal correction to Gibbs Free Energy = 0.344021
Sum of electronic and zero-point Energies = -915.523571
Sum of electronic and thermal Energies = -915.500359
Sum of electronic and thermal Enthalpies = -915.499414
Sum of electronic and thermal Free Energies = -915.574901

C	1.083848	2.543370	-0.071103
C	1.901564	1.427129	-0.012559
H	2.963926	1.612777	0.104023
C	1.527730	0.084117	-0.103522
O	0.213668	-0.256475	-0.266895
C	2.509043	-1.081073	-0.212935
C	1.591922	3.946681	0.045647
H	1.340065	4.543949	-0.841129
H	1.142127	4.471847	0.899558
H	2.678119	3.975520	0.171340
Si	-1.216887	-0.357065	0.641299
C	-1.204520	0.874859	2.062981
H	-2.004425	0.624575	2.768645
H	-0.258839	0.820741	2.612669
H	-1.349778	1.908608	1.741156
C	-1.380226	-2.085090	1.369118
H	-2.385631	-2.215860	1.785001
H	-1.224032	-2.872004	0.626188
H	-0.668863	-2.241540	2.185467
C	-2.632329	-0.034928	-0.608468
H	0.017462	2.419838	-0.220192
C	2.265647	-2.096413	0.927466
H	2.374887	-1.623614	1.908591
H	1.270113	-2.536602	0.867119
H	2.994767	-2.910526	0.862849

C	2.290323	-1.793746	-1.569695
H	2.485163	-1.114986	-2.406137
H	2.970712	-2.646605	-1.660379
H	1.267127	-2.163217	-1.662395
C	3.974996	-0.616722	-0.137725
H	4.631986	-1.486512	-0.228874
H	4.227966	0.075792	-0.945076
H	4.201842	-0.130708	0.815606
C	-3.969654	0.030786	0.162598
H	-4.798043	0.191778	-0.539407
H	-4.181407	-0.894767	0.707492
H	-3.988275	0.855438	0.882456
C	-2.427072	1.296528	-1.357454
H	-2.384389	2.151243	-0.675035
H	-1.508223	1.292927	-1.950051
H	-3.264306	1.471679	-2.046008
C	-2.703048	-1.181184	-1.639582
H	-2.897040	-2.148835	-1.166946
H	-3.518115	-0.996574	-2.351653
H	-1.777155	-1.269523	-2.216502

<u>SMD(MeCN)-LC-BLYP/6-311+G(d,p)</u>

E = -913.775900
Zero-point correction = 0.404746 (Hartree/Particle)
Thermal correction to Energy = 0.427182
Thermal correction to Enthalpy = 0.428126
Thermal correction to Gibbs Free Energy = 0.354771
Sum of electronic and zero-point Energies = -913.371154
Sum of electronic and thermal Energies = -913.348718
Sum of electronic and thermal Enthalpies = -913.347774
Sum of electronic and thermal Free Energies = -913.421129

C	0.995686	2.506673	-0.084244
C	1.852618	1.436659	-0.009765
H	2.904566	1.656180	0.130666
C	1.512875	0.101258	-0.113009
O	0.218957	-0.258068	-0.289401
C	2.497153	-1.038326	-0.207512
C	1.435320	3.919059	0.054409
H	1.188039	4.506431	-0.835374
H	0.937854	4.413589	0.894977
H	2.512430	3.989744	0.214942
Si	-1.182528	-0.375311	0.622195
C	-1.162900	0.843666	2.021191
H	-1.945034	0.583065	2.738802
H	-0.209094	0.805729	2.552990
H	-1.329391	1.871558	1.698498
C	-1.332912	-2.084201	1.332413
H	-2.338791	-2.222476	1.737607
H	-1.165990	-2.862446	0.586130
H	-0.628476	-2.240729	2.151192
C	-2.585785	-0.060724	-0.588941
H	-0.058097	2.331687	-0.261701
C	2.210281	-2.066531	0.881148

H	2.269040	-1.617806	1.875640
H	1.222935	-2.508422	0.760174
H	2.945414	-2.873039	0.832156
C	2.336944	-1.705734	-1.571109
H	2.567623	-1.007728	-2.379252
H	3.016142	-2.557326	-1.654952
H	1.317898	-2.066333	-1.712565
C	3.933597	-0.565063	-0.059878
H	4.603718	-1.423223	-0.138071
H	4.211240	0.145325	-0.840150
H	4.108556	-0.095489	0.910078
C	-3.901199	-0.004233	0.181494
H	-4.732927	0.156145	-0.512342
H	-4.103615	-0.931703	0.722743
H	-3.916290	0.815769	0.903819
C	-2.388764	1.258261	-1.323543
H	-2.353398	2.108289	-0.637709
H	-1.468498	1.261201	-1.910680
H	-3.223286	1.430023	-2.011777
C	-2.652121	-1.191725	-1.608490
H	-2.845661	-2.157120	-1.135446
H	-3.463213	-1.007891	-2.321019
H	-1.724730	-1.278341	-2.179885

[**1r**]$^{\bullet+}$

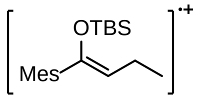

SMD(MeCN)-CAM-B3LYP/6-311+G(d,p)

E = -1107.653979
Zero-point correction = 0.463905 (Hartree/Particle)
Thermal correction to Energy = 0.491634
Thermal correction to Enthalpy = 0.492578
Thermal correction to Gibbs Free Energy = 0.405728
Sum of electronic and zero-point Energies = -1107.190074
Sum of electronic and thermal Energies = -1107.162345
Sum of electronic and thermal Enthalpies = -1107.161400
Sum of electronic and thermal Free Energies = -1107.248251

C	-1.072680	3.297834	-0.160037
C	0.177762	2.526554	-0.152671
H	1.128643	3.047892	-0.190116
C	0.235037	1.117733	-0.065840
O	-0.881501	0.486774	0.000521
C	-0.939555	4.687323	-0.770935
H	-0.679228	4.626151	-1.829649
H	-1.883591	5.227537	-0.686045
H	-0.167976	5.269554	-0.262631
Si	-1.555770	-1.033194	0.627202
C	-1.633397	-0.698561	2.451447
H	-2.217106	-1.479438	2.947998
H	-0.631218	-0.706860	2.887311
H	-2.099299	0.263874	2.672249

C	-0.457689	-2.473005	0.226068
H	-0.879122	-3.358650	0.713282
H	-0.396894	-2.680743	-0.843130
H	0.551848	-2.337708	0.618244
C	-3.226064	-1.080045	-0.244257
H	-1.870525	2.716433	-0.629287
H	-1.375143	3.385474	0.897994
C	-3.922715	-2.399189	0.131223
H	-4.906338	-2.442666	-0.348753
H	-3.356948	-3.272946	-0.203123
H	-4.082258	-2.491072	1.209573
C	-4.101041	0.098739	0.204815
H	-4.301510	0.072767	1.279180
H	-3.642500	1.062795	-0.030689
H	-5.066354	0.057275	-0.311688
C	-3.036917	-1.025780	-1.767139
H	-2.562956	-0.094382	-2.087567
H	-2.432743	-1.858687	-2.136268
H	-4.013256	-1.085823	-2.260514
C	1.531525	0.404474	-0.097720
C	1.890126	-0.309909	-1.253220
C	2.389348	0.481925	1.008997
C	3.114477	-0.963766	-1.264481
C	1.009728	-0.347847	-2.474554
C	3.594377	-0.210402	0.955643
C	2.034187	1.280146	2.235125
C	3.971983	-0.940073	-0.166731
H	3.409818	-1.502451	-2.159074
H	0.210652	-1.085467	-2.372785
H	0.543580	0.619602	-2.673475
H	1.596737	-0.622741	-3.351141
H	4.256176	-0.172941	1.814192
H	2.061975	2.354172	2.028298
H	1.033603	1.043743	2.604438
H	2.744371	1.080809	3.037828
C	5.277449	-1.682671	-0.205507
H	5.895961	-1.444111	0.660557
H	5.108076	-2.763072	-0.212349
H	5.840683	-1.438682	-1.109226

SMD(MeCN)-B3LYP/6-311+G(d,p)

E = -1108.155915
Zero-point correction = 0.459821 (Hartree/Particle)
Thermal correction to Energy = 0.487442
Thermal correction to Enthalpy = 0.488386
Thermal correction to Gibbs Free Energy = 0.401626
Sum of electronic and zero-point Energies = -1107.696094
Sum of electronic and thermal Energies = -1107.668473
Sum of electronic and thermal Enthalpies = -1107.667529
Sum of electronic and thermal Free Energies = -1107.754289

C	-0.929019	3.296486	-0.280212
C	0.285167	2.471179	-0.131607
H	1.240854	2.979059	-0.066873

C	0.306097	1.063145	-0.089355
O	-0.837576	0.433627	-0.152759
C	-0.727976	4.516246	-1.192058
H	-0.505445	4.205917	-2.216095
H	-1.637582	5.120545	-1.209824
H	0.093086	5.145143	-0.838089
Si	-1.689507	-0.834032	0.733674
C	-1.917675	-0.084342	2.432544
H	-2.541720	-0.738817	3.050199
H	-0.953875	0.027324	2.939086
H	-2.397289	0.896614	2.386258
C	-0.655800	-2.387246	0.817226
H	-1.174525	-3.112522	1.454310
H	-0.500225	-2.853488	-0.157935
H	0.319331	-2.195813	1.273156
C	-3.308560	-1.035408	-0.242949
H	-1.777694	2.688385	-0.597817
H	-1.174405	3.655826	0.735572
C	-4.130400	-2.152961	0.441327
H	-5.086826	-2.276055	-0.081036
H	-3.615635	-3.118251	0.414344
H	-4.357875	-1.919648	1.486524
C	-4.123810	0.273940	-0.221059
H	-4.382313	0.580257	0.796938
H	-3.586750	1.099768	-0.696748
H	-5.063372	0.132169	-0.769378
C	-3.030311	-1.438579	-1.705394
H	-2.475213	-0.665569	-2.244300
H	-2.466742	-2.373733	-1.775655
H	-3.980519	-1.588294	-2.233054
C	1.579206	0.331414	-0.102149
C	1.834629	-0.603419	-1.148119
C	2.561373	0.581759	0.896290
C	3.059816	-1.258212	-1.163066
C	0.870993	-0.844284	-2.285631
C	3.753316	-0.135723	0.847723
C	2.345828	1.548020	2.036542
C	4.027988	-1.054119	-0.170904
H	3.276257	-1.940028	-1.979397
H	0.125843	-1.600792	-2.028571
H	0.336452	0.061731	-2.576509
H	1.416110	-1.209038	-3.158006
H	4.486909	0.019643	1.631737
H	2.554911	2.577887	1.727779
H	1.325842	1.519487	2.423927
H	3.026596	1.315147	2.857409
C	5.334257	-1.795829	-0.218082
H	5.959818	-1.561367	0.644745
H	5.167937	-2.877473	-0.242866
H	5.891061	-1.538988	-1.125599

<u>SMD(MeCN)-LC-BLYP/6-311+G(d,p)</u>

E = -1105.410599

Zero-point correction = 0.471175 (Hartree/Particle)
Thermal correction to Energy = 0.497955
Thermal correction to Enthalpy = 0.498899
Thermal correction to Gibbs Free Energy = 0.414533
Sum of electronic and zero-point Energies = -1104.939425
Sum of electronic and thermal Energies = -1104.912644
Sum of electronic and thermal Enthalpies = -1104.911700
Sum of electronic and thermal Free Energies = -1104.996066

C	-1.201606	3.282870	0.076045
C	0.061965	2.558887	-0.056528
H	0.984189	3.100098	-0.244470
C	0.166968	1.163764	0.078869
O	-0.904258	0.518581	0.310231
C	-1.148490	4.701596	-0.446317
H	-0.932253	4.716976	-1.515456
H	-2.103880	5.202068	-0.289895
H	-0.376841	5.279387	0.064574
Si	-1.475447	-1.128145	0.604761
C	-1.771745	-1.132633	2.419865
H	-2.387122	-1.994363	2.690605
H	-0.835329	-1.210965	2.974492
H	-2.294299	-0.231946	2.744957
C	-0.207295	-2.346716	0.067761
H	-0.594255	-3.346951	0.282310
H	0.000816	-2.294891	-1.001064
H	0.728864	-2.233041	0.615187
C	-3.033721	-1.157199	-0.422013
H	-2.003889	2.703406	-0.389461
H	-1.448665	3.277635	1.148583
C	-3.616131	-2.566589	-0.351318
H	-4.548871	-2.606386	-0.921832
H	-2.939399	-3.310917	-0.777372
H	-3.848205	-2.867057	0.673683
C	-4.046394	-0.163355	0.133331
H	-4.332237	-0.403588	1.159751
H	-3.664565	0.860234	0.119724
H	-4.955993	-0.184142	-0.474688
C	-2.725305	-0.814792	-1.874398
H	-2.326254	0.196452	-1.982041
H	-2.008672	-1.510636	-2.316540
H	-3.643747	-0.869528	-2.466716
C	1.471314	0.477785	-0.033834
C	1.896953	0.021333	-1.275609
C	2.246195	0.319600	1.108016
C	3.115622	-0.622897	-1.349072
C	1.055770	0.195075	-2.500631
C	3.456732	-0.335463	0.988002
C	1.793245	0.845008	2.434429
C	3.903362	-0.817400	-0.228599
H	3.461726	-0.980820	-2.313204
H	0.214586	-0.501788	-2.501221
H	0.644726	1.203937	-2.574423
H	1.642732	0.005239	-3.398062

H	4.071700	-0.464815	1.872188
H	1.595232	1.918442	2.391375
H	0.875826	0.357149	2.770210
H	2.555228	0.676288	3.193715
C	5.205095	-1.544385	-0.333772
H	5.856408	-1.315915	0.509547
H	5.040083	-2.624270	-0.342720
H	5.727491	-1.286842	-1.255599

[**1r-H**]•

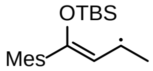

CAM-B3LYP/6-311+G(d,p)

E = -1107.205038
Zero-point correction = 0.452097 (Hartree/Particle)
Thermal correction to Energy = 0.479686
Thermal correction to Enthalpy = 0.480631
Thermal correction to Gibbs Free Energy = 0.392433
Sum of electronic and zero-point Energies = -1106.752941
Sum of electronic and thermal Energies = -1106.725351
Sum of electronic and thermal Enthalpies = -1106.724407
Sum of electronic and thermal Free Energies = -1106.812605

C	-1.065271	3.172856	-0.589462
C	0.113093	2.486516	-0.444439
H	1.041220	3.050625	-0.434743
C	0.246219	1.103571	-0.331648
O	-0.874021	0.331521	-0.426267
C	1.549920	0.412108	-0.226596
C	2.431753	0.679787	0.832959
C	1.916397	-0.529253	-1.210294
C	3.641370	-0.009293	0.902080
C	3.133146	-1.186094	-1.103674
C	4.009697	-0.948518	-0.048706
H	4.311119	0.195226	1.731518
H	3.412993	-1.897230	-1.875076
C	-1.153401	4.655659	-0.736201
H	-1.617002	4.937131	-1.688166
H	-1.770866	5.101195	0.051497
H	-0.167502	5.123265	-0.693936
Si	-1.568472	-0.741579	0.674801
C	-1.810501	0.145000	2.306538
H	-2.369271	-0.475958	3.011660
H	-0.847185	0.372992	2.769065
H	-2.350896	1.085270	2.181387
C	-0.471883	-2.235752	0.936827
H	-0.921920	-2.916431	1.664730
H	-0.306409	-2.794504	0.013728
H	0.506155	-1.938062	1.320590
C	-3.215097	-1.211336	-0.140796
H	-1.990915	2.608591	-0.615820
C	2.119679	1.687503	1.910923
H	2.419712	2.695757	1.612038

H	1.054805	1.730603	2.137493
H	2.661403	1.445592	2.826594
C	1.033352	-0.806575	-2.398802
H	0.213539	-1.480835	-2.144210
H	0.579179	0.110057	-2.778564
H	1.610536	-1.266265	-3.202144
C	5.309915	-1.699241	0.054498
H	5.831234	-1.721416	-0.905010
H	5.974510	-1.243640	0.789757
H	5.140659	-2.736524	0.356860
C	-2.955995	-1.834989	-1.520263
H	-3.906562	-2.093339	-2.000753
H	-2.426428	-1.143074	-2.178725
H	-2.367871	-2.754074	-1.450126
C	-3.958962	-2.227554	0.739855
H	-4.186843	-1.825210	1.730901
H	-4.912483	-2.499997	0.273983
H	-3.388603	-3.150571	0.874229
C	-4.090778	0.037615	-0.317738
H	-4.327448	0.511670	0.638564
H	-3.602989	0.780141	-0.952792
H	-5.041002	-0.233113	-0.791961

<u>SMD(MeCN)-CAM-B3LYP/6-311+G(d,p)</u>

E = -1107.220682
Zero-point correction = 0.450833 (Hartree/Particle)
Thermal correction to Energy = 0.478125
Thermal correction to Enthalpy = 0.479069
Thermal correction to Gibbs Free Energy = 0.393195
Sum of electronic and zero-point Energies = -1106.769849
Sum of electronic and thermal Energies = -1106.742557
Sum of electronic and thermal Enthalpies = -1106.741612
Sum of electronic and thermal Free Energies = -1106.827487

C	-1.050123	3.146675	-0.595928
C	0.126168	2.458007	-0.432256
H	1.051748	3.025899	-0.398485
C	0.259047	1.072098	-0.324150
O	-0.856395	0.300845	-0.436830
C	1.569463	0.389789	-0.216896
C	2.441849	0.660513	0.854787
C	1.954359	-0.539293	-1.203876
C	3.662125	-0.007892	0.924185
C	3.184868	-1.179278	-1.096156
C	4.054622	-0.930770	-0.038771
H	4.320543	0.194847	1.763372
H	3.476010	-1.885780	-1.867362
C	-1.130539	4.630802	-0.733488
H	-1.572029	4.921334	-1.693948
H	-1.769376	5.070891	0.040879
H	-0.144665	5.096075	-0.662108
Si	-1.583791	-0.780396	0.645909
C	-1.722010	0.052486	2.313797

H	-2.352133	-0.537127	2.986489
H	-0.740235	0.146635	2.786151
H	-2.155470	1.052608	2.237433
C	-0.569412	-2.341968	0.808725
H	-1.042190	-3.020449	1.525360
H	-0.469123	-2.875384	-0.139427
H	0.433943	-2.122997	1.182042
C	-3.277246	-1.131524	-0.132581
H	-1.979724	2.588272	-0.639953
C	2.097572	1.642823	1.944942
H	2.287700	2.672649	1.630380
H	1.046262	1.584114	2.229952
H	2.706634	1.454122	2.830487
C	1.081363	-0.834773	-2.395111
H	0.256975	-1.502079	-2.135751
H	0.636961	0.076572	-2.800953
H	1.664881	-1.312831	-3.183537
C	5.392111	-1.614669	0.044408
H	6.179586	-0.983367	-0.378928
H	5.666489	-1.825328	1.080110
H	5.392473	-2.554893	-0.509854
C	-3.108906	-1.601540	-1.584475
H	-4.090394	-1.813952	-2.025183
H	-2.625254	-0.841485	-2.202693
H	-2.516262	-2.518385	-1.652522
C	-3.983259	-2.235608	0.669989
H	-4.125923	-1.958368	1.718836
H	-4.975921	-2.428193	0.246389
H	-3.428529	-3.177573	0.644545
C	-4.144755	0.134687	-0.111615
H	-4.325661	0.489175	0.906976
H	-3.685477	0.951652	-0.674319
H	-5.121404	-0.071258	-0.565643

<u>SMD(DCE)-CAM-B3LYP/6-311+G(d,p)</u>

E = -1107.221441
Zero-point correction = 0.451166 (Hartree/Particle)
Thermal correction to Energy = 0.478452
Thermal correction to Enthalpy = 0.479396
Thermal correction to Gibbs Free Energy = 0.393619
Sum of electronic and zero-point Energies = -1106.770275
Sum of electronic and thermal Energies = -1106.742989
Sum of electronic and thermal Enthalpies = -1106.742045
Sum of electronic and thermal Free Energies = -1106.827822

C	-1.053227	3.142312	-0.592109
C	0.123423	2.455418	-0.425744
H	1.047933	3.024660	-0.387411
C	0.259477	1.069910	-0.321271
O	-0.854046	0.295393	-0.437606
C	1.571268	0.390053	-0.214789
C	2.439702	0.654887	0.860951
C	1.961395	-0.529950	-1.208169
C	3.660736	-0.012225	0.929635

C	3.192442	-1.168035	-1.101273
C	4.057864	-0.926697	-0.038768
H	4.316358	0.185514	1.772197
H	3.487958	-1.867597	-1.877095
C	-1.136329	4.626486	-0.725729
H	-1.573828	4.918842	-1.687416
H	-1.779097	5.063358	0.047194
H	-0.151941	5.094001	-0.648977
Si	-1.583728	-0.785357	0.642451
C	-1.716589	0.042276	2.313917
H	-2.352250	-0.543021	2.985127
H	-0.734884	0.125429	2.788387
H	-2.139536	1.047135	2.241874
C	-0.575929	-2.351685	0.802829
H	-1.043178	-3.024124	1.528760
H	-0.486523	-2.892734	-0.142002
H	0.432621	-2.135845	1.163605
C	-3.280068	-1.128580	-0.134446
H	-1.981103	2.581949	-0.642921
C	2.091811	1.632181	1.954462
H	2.302609	2.661737	1.652005
H	1.035332	1.588277	2.221672
H	2.683259	1.427924	2.848553
C	1.092305	-0.816050	-2.404432
H	0.266867	-1.485075	-2.153259
H	0.648944	0.098474	-2.804078
H	1.677824	-1.287606	-3.195285
C	5.396170	-1.609206	0.042739
H	6.181389	-0.980744	-0.388871
H	5.675675	-1.813619	1.078270
H	5.394718	-2.553333	-0.504907
C	-3.115081	-1.590578	-1.589329
H	-4.097544	-1.795875	-2.031133
H	-2.628299	-0.829128	-2.203179
H	-2.527276	-2.510008	-1.663896
C	-3.987898	-2.235097	0.663083
H	-4.129068	-1.963729	1.713677
H	-4.981727	-2.423322	0.240404
H	-3.436259	-3.178680	0.631718
C	-4.144119	0.139822	-0.105846
H	-4.321284	0.491541	0.914347
H	-3.684423	0.957056	-0.667539
H	-5.122826	-0.061052	-0.557576

SMD(MeCN)-B3LYP/6-311+G(d,p)

E = -1107.718536

Zero-point correction = 0.446561 (Hartree/Particle)

Thermal correction to Energy = 0.473966

Thermal correction to Enthalpy = 0.474910

Thermal correction to Gibbs Free Energy = 0.389210

Sum of electronic and zero-point Energies = -1107.271974

Sum of electronic and thermal Energies = -1107.244570

Sum of electronic and thermal Enthalpies = -1107.243626

Sum of electronic and thermal Free Energies = -1107.329326

C	-1.025025	3.149465	-0.605718
C	0.146118	2.445254	-0.428809
H	1.076231	3.007080	-0.394829
C	0.275697	1.053084	-0.315388
O	-0.846123	0.281039	-0.436035
C	1.590047	0.372367	-0.210682
C	2.474464	0.653503	0.860357
C	1.980661	-0.564124	-1.200926
C	3.703649	-0.010676	0.925112
C	3.221086	-1.198032	-1.094505
C	4.099602	-0.940313	-0.038811
H	4.364800	0.199471	1.761172
H	3.513365	-1.904422	-1.866322
C	-1.089319	4.636541	-0.756859
H	-1.520093	4.925056	-1.725082
H	-1.733788	5.091126	0.007161
H	-0.099673	5.095529	-0.679639
Si	-1.621279	-0.775443	0.655159
C	-1.766282	0.082177	2.321566
H	-2.398466	-0.501667	2.999208
H	-0.784749	0.181355	2.796159
H	-2.200119	1.082370	2.235014
C	-0.635135	-2.362830	0.855990
H	-1.123875	-3.016478	1.586633
H	-0.541404	-2.919926	-0.080381
H	0.372304	-2.154734	1.228180
C	-3.326025	-1.105637	-0.146086
H	-1.962420	2.603311	-0.654424
C	2.133823	1.638382	1.955985
H	2.308397	2.671687	1.638504
H	1.086431	1.569996	2.257501
H	2.757650	1.458437	2.834546
C	1.111485	-0.867284	-2.399031
H	0.282550	-1.532480	-2.143373
H	0.671033	0.042623	-2.815855
H	1.699631	-1.352019	-3.181554
C	5.445543	-1.618066	0.040547
H	6.230708	-0.982762	-0.385750
H	5.726182	-1.826911	1.076395
H	5.450903	-2.559764	-0.513617
C	-3.155797	-1.588688	-1.601298
H	-4.138742	-1.796715	-2.044240
H	-2.665605	-0.835867	-2.224778
H	-2.569676	-2.511224	-1.663338
C	-4.057395	-2.200515	0.661630
H	-4.203798	-1.916040	1.709034
H	-5.051320	-2.380530	0.232035
H	-3.516995	-3.152030	0.645009
C	-4.181789	0.177737	-0.138473
H	-4.368576	0.539781	0.877378
H	-3.708417	0.987797	-0.701304
H	-5.158032	-0.018243	-0.600997

SMD(MeCN)-LC-BLYP/6-311+G(d,p)

E = -1104.982150
Zero-point correction = 0.456969 (Hartree/Particle)
Thermal correction to Energy = 0.483809
Thermal correction to Enthalpy = 0.484753
Thermal correction to Gibbs Free Energy = 0.399648
Sum of electronic and zero-point Energies = -1104.525180
Sum of electronic and thermal Energies = -1104.498340
Sum of electronic and thermal Enthalpies = -1104.497396
Sum of electronic and thermal Free Energies = -1104.582502

C	-1.089035	3.149863	-0.584102
C	0.093548	2.483357	-0.440620
H	1.012674	3.061175	-0.410244
C	0.234054	1.105971	-0.342792
O	-0.871520	0.333210	-0.441330
C	1.535836	0.423009	-0.233480
C	2.398805	0.686904	0.832241
C	1.904053	-0.511926	-1.204997
C	3.600453	0.004519	0.912301
C	3.115548	-1.166628	-1.090499
C	3.978204	-0.924796	-0.037212
H	4.258996	0.201795	1.752438
H	3.397930	-1.885166	-1.853532
C	-1.194392	4.626576	-0.704131
H	-1.655796	4.918771	-1.652369
H	-1.824343	5.047157	0.085493
H	-0.215198	5.104647	-0.644859
Si	-1.525078	-0.782535	0.635916
C	-1.660841	0.028638	2.297702
H	-2.281973	-0.570184	2.968429
H	-0.678981	0.130077	2.765497
H	-2.104803	1.023320	2.226764
C	-0.457948	-2.294041	0.757736
H	-0.884511	-2.991684	1.482844
H	-0.368208	-2.816610	-0.196108
H	0.546597	-2.040785	1.101295
C	-3.199325	-1.182100	-0.109290
H	-2.007855	2.573333	-0.620235
C	2.059937	1.675384	1.906419
H	2.267885	2.698296	1.585847
H	1.005616	1.633578	2.178639
H	2.655894	1.484367	2.798811
C	1.024395	-0.806040	-2.380768
H	0.208881	-1.478354	-2.110813
H	0.568628	0.102668	-2.776155
H	1.600058	-1.278119	-3.176929
C	5.296556	-1.625625	0.056567
H	6.092570	-1.010423	-0.370087
H	5.563771	-1.829905	1.094014
H	5.283602	-2.569746	-0.488385
C	-3.028849	-1.633962	-1.554558
H	-4.005116	-1.871286	-1.990526

H	-2.569515	-0.856354	-2.168038
H	-2.410722	-2.532073	-1.631236
C	-3.855497	-2.302525	0.689737
H	-3.992133	-2.034680	1.740825
H	-4.845849	-2.523841	0.278566
H	-3.272557	-3.225863	0.652230
C	-4.092902	0.051139	-0.070350
H	-4.274010	0.390594	0.952378
H	-3.657708	0.883814	-0.627653
H	-5.066167	-0.175025	-0.518508

[**1s**]$^{\bullet +}$

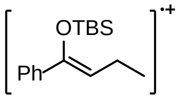

SMD(MeCN)-CAM-B3LYP/6-311+G(d,p)

E = -989.751559
Zero-point correction = 0.381320 (Hartree/Particle)
Thermal correction to Energy = 0.403829
Thermal correction to Enthalpy = 0.404773
Thermal correction to Gibbs Free Energy = 0.328706
Sum of electronic and zero-point Energies = -989.370238
Sum of electronic and thermal Energies = -989.347729
Sum of electronic and thermal Enthalpies = -989.346785
Sum of electronic and thermal Free Energies = -989.422853

C	0.222431	3.248239	-0.105760
C	1.265742	2.213497	-0.062076
H	2.303276	2.522391	-0.020395
C	0.989873	0.828849	-0.067152
O	-0.250241	0.462250	-0.128013
C	0.715152	4.595816	-0.619743
H	1.055923	4.519093	-1.654434
H	-0.089827	5.331318	-0.583573
H	1.543942	4.969673	-0.014727
Si	-1.502794	-0.382109	0.755576
C	-2.088630	0.863920	2.003065
H	-2.917062	0.448974	2.584963
H	-1.287293	1.114951	2.703558
H	-2.435946	1.784907	1.531233
C	-0.754825	-1.867985	1.581285
H	-1.488194	-2.285577	2.278400
H	-0.477867	-2.654351	0.877582
H	0.128468	-1.598203	2.166024
C	-2.765211	-0.797306	-0.581824
H	-0.641700	2.886694	-0.669116
H	-0.142363	3.356337	0.929970
C	-3.864791	-1.661442	0.059217
H	-4.625632	-1.900844	-0.691636
H	-3.472552	-2.607624	0.442332
H	-4.369834	-1.146469	0.881709
C	-3.395043	0.483679	-1.146311
H	-3.922789	1.052644	-0.376565

H	-2.649153	1.141023	-1.601503
H	-4.123593	0.224691	-1.922543
C	-2.108156	-1.586275	-1.722715
H	-1.343755	-0.998521	-2.237481
H	-1.646235	-2.512580	-1.370469
H	-2.867584	-1.860088	-2.463491
C	2.050456	-0.164353	-0.101829
C	1.795311	-1.426738	-0.660785
C	3.331326	0.119994	0.401655
C	2.796623	-2.376156	-0.717391
C	4.319991	-0.844663	0.361553
C	4.057176	-2.089922	-0.200713
H	2.600034	-3.341202	-1.167799
H	5.299570	-0.627446	0.768795
H	0.822998	-1.643104	-1.081637
H	3.544667	1.079166	0.854537
H	4.839000	-2.839266	-0.240537

SMD(MeCN)-B3LYP/6-311+G(d,p)

E = -990.180251
Zero-point correction = 0.377297 (Hartree/Particle)
Thermal correction to Energy = 0.400139
Thermal correction to Enthalpy = 0.401083
Thermal correction to Gibbs Free Energy = 0.323090
Sum of electronic and zero-point Energies = -989.802953
Sum of electronic and thermal Energies = -989.780111
Sum of electronic and thermal Enthalpies = -989.779167
Sum of electronic and thermal Free Energies = -989.857161

C	0.245983	3.262705	-0.036849
C	1.273166	2.206672	0.007627
H	2.309476	2.513815	0.085528
C	1.003071	0.820026	-0.044697
O	-0.241228	0.435250	-0.150249
C	0.713059	4.541572	-0.744959
H	0.925333	4.347828	-1.799401
H	-0.066966	5.304064	-0.689884
H	1.617532	4.943750	-0.281028
Si	-1.523112	-0.355606	0.752086
C	-2.118805	0.939736	1.961307
H	-2.955656	0.544254	2.546881
H	-1.322512	1.206335	2.663583
H	-2.457119	1.850005	1.460980
C	-0.795158	-1.823593	1.649837
H	-1.553786	-2.234847	2.325166
H	-0.475426	-2.623269	0.978175
H	0.058926	-1.527044	2.266421
C	-2.792573	-0.813546	-0.586677
H	-0.685557	2.881218	-0.459495
H	0.017208	3.505768	1.017070
C	-3.913507	-1.636897	0.088811
H	-4.676309	-1.895136	-0.655716
H	-3.538211	-2.573681	0.512304
H	-4.414351	-1.080423	0.887691

C	-3.406275	0.455482	-1.213124
H	-3.926547	1.068101	-0.470945
H	-2.651145	1.080418	-1.699582
H	-4.139757	0.170812	-1.977497
C	-2.141117	-1.665276	-1.695245
H	-1.366793	-1.111751	-2.234264
H	-1.692823	-2.582990	-1.302559
H	-2.903742	-1.960662	-2.426477
C	2.073981	-0.165071	-0.091365
C	1.826867	-1.435277	-0.658608
C	3.365190	0.123220	0.410898
C	2.837656	-2.379522	-0.725996
C	4.362782	-0.837308	0.359390
C	4.105647	-2.086727	-0.212266
H	2.646216	-3.343551	-1.182220
H	5.343029	-0.616329	0.764554
H	0.853657	-1.656193	-1.075478
H	3.575836	1.079127	0.872352
H	4.893064	-2.830320	-0.261882

<u>SMD(MeCN)-LC-BLYP/6-311+G(d,p)</u>

E = -987.806410
Zero-point correction = 0.386247 (Hartree/Particle)
Thermal correction to Energy = 0.408449
Thermal correction to Enthalpy = 0.409394
Thermal correction to Gibbs Free Energy = 0.333502
Sum of electronic and zero-point Energies = -987.420163
Sum of electronic and thermal Energies = -987.397961
Sum of electronic and thermal Enthalpies = -987.397016
Sum of electronic and thermal Free Energies = -987.472908

C	0.155081	3.211712	-0.188915
C	1.231428	2.221272	-0.170068
H	2.263780	2.547840	-0.217731
C	0.972884	0.840931	-0.101682
O	-0.254754	0.476648	-0.087479
C	0.627274	4.625984	-0.437841
H	1.121592	4.709222	-1.406865
H	-0.214725	5.317849	-0.430278
H	1.332261	4.945942	0.330904
Si	-1.481045	-0.422283	0.759523
C	-2.076799	0.766597	2.033315
H	-2.896630	0.323498	2.604215
H	-1.278604	1.009567	2.738246
H	-2.438309	1.694116	1.588077
C	-0.719936	-1.916132	1.521436
H	-1.436397	-2.346911	2.225961
H	-0.465713	-2.684806	0.791870
H	0.178361	-1.664196	2.088738
C	-2.718038	-0.796454	-0.585772
H	-0.594879	2.898714	-0.923803
H	-0.370684	3.136400	0.773506
C	-3.810191	-1.677519	0.015011
H	-4.562604	-1.900131	-0.747467

H	-3.415573	-2.631195	0.374225
H	-4.323933	-1.188816	0.846930
C	-3.338604	0.492998	-1.108704
H	-3.878443	1.031532	-0.326875
H	-2.587752	1.168234	-1.526523
H	-4.053546	0.262416	-1.904423
C	-2.049370	-1.540191	-1.735089
H	-1.281135	-0.933870	-2.220430
H	-1.588516	-2.475087	-1.407251
H	-2.796921	-1.792008	-2.493420
C	2.034933	-0.145937	-0.120528
C	1.779738	-1.416570	-0.626662
C	3.306702	0.166137	0.352530
C	2.780740	-2.356953	-0.659722
C	4.296861	-0.787115	0.336773
C	4.036253	-2.045668	-0.171497
H	2.584816	-3.339771	-1.070217
H	5.280333	-0.548102	0.721853
H	0.803223	-1.655236	-1.026541
H	3.516798	1.146768	0.760012
H	4.822133	-2.791732	-0.191720

[**1s-H**]•

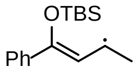

CAM-B3LYP/6-311+G(d,p)

E = -989.307159
Zero-point correction = 0.369576 (Hartree/Particle)
Thermal correction to Energy = 0.391750
Thermal correction to Enthalpy = 0.392694
Thermal correction to Gibbs Free Energy = 0.316792
Sum of electronic and zero-point Energies = -988.937583
Sum of electronic and thermal Energies = -988.915409
Sum of electronic and thermal Enthalpies = -988.914464
Sum of electronic and thermal Free Energies = -988.990367

C	0.006414	3.100948	-0.360052
C	1.045926	2.239513	-0.190457
H	2.037820	2.660297	-0.067809
C	0.958767	0.833305	-0.212413
O	-0.257214	0.262833	-0.432510
C	2.107603	-0.051706	-0.163565
C	3.357825	0.368764	0.326813
C	2.000644	-1.374733	-0.624495
C	4.440081	-0.491542	0.349109
H	3.477923	1.371492	0.715772
C	3.089397	-2.229271	-0.602988
H	1.054284	-1.714165	-1.022174
C	4.316802	-1.797096	-0.115835
H	5.389143	-0.143861	0.740209
H	2.981091	-3.241464	-0.975252
H	5.167077	-2.467744	-0.096929
C	0.143416	4.587238	-0.362977

H	-0.187933	5.014878	-1.315151
H	-0.475854	5.046067	0.415254
H	1.176925	4.897266	-0.196686
Si	-1.394505	-0.347228	0.657769
C	-1.938995	1.007389	1.829777
H	-2.603478	0.603146	2.598224
H	-1.075474	1.443496	2.337989
H	-2.465493	1.816619	1.321511
C	-0.645157	-1.737075	1.662499
H	-1.358579	-2.092563	2.411017
H	-0.349008	-2.587997	1.047430
H	0.244822	-1.392561	2.194752
C	-2.798739	-0.941273	-0.466888
H	-0.984453	2.693484	-0.526240
C	-3.926978	-1.538911	0.387918
H	-4.744502	-1.882031	-0.255738
H	-3.589026	-2.400761	0.969944
H	-4.347418	-0.806960	1.083233
C	-3.350482	0.237583	-1.282263
H	-3.777032	1.014148	-0.641722
H	-2.574241	0.695174	-1.899618
H	-4.148294	-0.107465	-1.949603
C	-2.275454	-2.012240	-1.435472
H	-1.895946	-2.891694	-0.908295
H	-3.083522	-2.351287	-2.093465
H	-1.474475	-1.622357	-2.067609

SMD(MeCN)-CAM-B3LYP/6-311+G(d,p)

E = -989.323061
Zero-point correction = 0.368144 (Hartree/Particle)
Thermal correction to Energy = 0.390288
Thermal correction to Enthalpy = 0.391232
Thermal correction to Gibbs Free Energy = 0.316498
Sum of electronic and zero-point Energies = -988.954917
Sum of electronic and thermal Energies = -988.932773
Sum of electronic and thermal Enthalpies = -988.931829
Sum of electronic and thermal Free Energies = -989.006563

C	-0.016982	3.073056	-0.346000
C	1.035722	2.227010	-0.176434
H	2.018503	2.665614	-0.041974
C	0.969157	0.816161	-0.202693
O	-0.234569	0.226372	-0.426942
C	2.134026	-0.048200	-0.158435
C	3.398213	0.412532	0.263108
C	2.031259	-1.395312	-0.552835
C	4.494291	-0.431933	0.281462
H	3.522921	1.434858	0.595648
C	3.134901	-2.233017	-0.535808
H	1.075869	-1.774125	-0.888800
C	4.374849	-1.761109	-0.118448
H	5.452526	-0.052011	0.617528
H	3.026678	-3.264089	-0.853496
H	5.236271	-2.418203	-0.102641

C	0.097979	4.560481	-0.334818
H	-0.244728	4.991134	-1.282224
H	-0.532491	4.999619	0.446501
H	1.126333	4.886538	-0.165083
Si	-1.406712	-0.361428	0.651445
C	-1.937431	0.999691	1.817163
H	-2.602169	0.591215	2.584454
H	-1.074393	1.430209	2.332991
H	-2.467286	1.809400	1.311547
C	-0.693752	-1.763027	1.662060
H	-1.444332	-2.131855	2.368164
H	-0.367415	-2.605394	1.049300
H	0.162680	-1.421479	2.250481
C	-2.813226	-0.928516	-0.483723
H	-1.005386	2.656890	-0.508780
C	-3.989905	-1.408877	0.380148
H	-4.809729	-1.750074	-0.262591
H	-3.712681	-2.247159	1.026164
H	-4.384103	-0.610852	1.015918
C	-3.283973	0.233816	-1.369004
H	-3.646817	1.079543	-0.778204
H	-2.484361	0.595902	-2.019871
H	-4.110506	-0.095189	-2.010137
C	-2.345003	-2.082415	-1.381200
H	-2.024939	-2.951360	-0.799712
H	-3.166342	-2.407900	-2.030668
H	-1.514336	-1.784257	-2.026469

SMD(DCE)-CAM-B3LYP/6-311+G(d,p)

E = -989.323605
Zero-point correction = 0.368431 (Hartree/Particle)
Thermal correction to Energy = 0.390546
Thermal correction to Enthalpy = 0.391490
Thermal correction to Gibbs Free Energy = 0.316962
Sum of electronic and zero-point Energies = -988.955174
Sum of electronic and thermal Energies = -988.933059
Sum of electronic and thermal Enthalpies = -988.932114
Sum of electronic and thermal Free Energies = -989.006642

C	-0.013616	3.076156	-0.346179
C	1.037214	2.227725	-0.177401
H	2.021688	2.663866	-0.047757
C	0.968633	0.817495	-0.201188
O	-0.236542	0.228828	-0.422376
C	2.132628	-0.048470	-0.158405
C	3.394455	0.406947	0.274407
C	2.030468	-1.391626	-0.564690
C	4.489369	-0.438791	0.291929
H	3.517646	1.426111	0.617007
C	3.132733	-2.230763	-0.548589
H	1.076483	-1.764880	-0.910614
C	4.370501	-1.764063	-0.119826
H	5.446054	-0.063140	0.636900
H	3.025396	-3.258753	-0.876210

H	5.230943	-2.422356	-0.104635
C	0.105025	4.563278	-0.340346
H	-0.236749	4.991530	-1.289150
H	-0.522908	5.007515	0.440102
H	1.134316	4.887589	-0.172981
Si	-1.406864	-0.362254	0.654113
C	-1.939503	0.994997	1.824014
H	-2.599245	0.584012	2.594241
H	-1.076247	1.428651	2.336729
H	-2.474307	1.804395	1.323186
C	-0.694028	-1.765361	1.663128
H	-1.443102	-2.136111	2.369824
H	-0.366264	-2.607421	1.050816
H	0.162709	-1.424913	2.251582
C	-2.812332	-0.927248	-0.484005
H	-1.002836	2.661489	-0.507024
C	-3.987768	-1.418069	0.375409
H	-4.807287	-1.755327	-0.269693
H	-3.709163	-2.261839	1.013596
H	-4.384299	-0.627212	1.018684
C	-3.286311	0.239380	-1.361964
H	-3.657853	1.077860	-0.766244
H	-2.486012	0.611660	-2.006070
H	-4.107865	-0.088916	-2.009727
C	-2.338952	-2.072509	-1.389816
H	-2.014230	-2.944263	-0.815155
H	-3.158704	-2.398125	-2.041111
H	-1.510815	-1.764954	-2.033742

SMD(MeCN)-B3LYP/6-311+G(d,p)

E = -989.747649
Zero-point correction = 0.364581 (Hartree/Particle)
Thermal correction to Energy = 0.386917
Thermal correction to Enthalpy = 0.387861
Thermal correction to Gibbs Free Energy = 0.312954
Sum of electronic and zero-point Energies = -989.383067
Sum of electronic and thermal Energies = -989.360732
Sum of electronic and thermal Enthalpies = -989.359787
Sum of electronic and thermal Free Energies = -989.434695

C	0.028022	3.097563	-0.345651
C	1.066451	2.224246	-0.176664
H	2.058642	2.645631	-0.050563
C	0.981929	0.809688	-0.196391
O	-0.233430	0.229370	-0.414894
C	2.142257	-0.064461	-0.154561
C	3.418134	0.388363	0.266989
C	2.033646	-1.418398	-0.552766
C	4.513324	-0.465219	0.280960
H	3.550378	1.409099	0.603335
C	3.137091	-2.264222	-0.540374
H	1.076515	-1.793316	-0.889236
C	4.386386	-1.798695	-0.123372
H	5.474568	-0.090671	0.616917

H	3.022404	-3.294469	-0.861080
H	5.244851	-2.460756	-0.111102
C	0.176379	4.585405	-0.341920
H	-0.163853	5.021517	-1.289997
H	-0.442093	5.043735	0.440740
H	1.212843	4.892455	-0.178909
Si	-1.426743	-0.354176	0.660002
C	-1.954732	1.015777	1.833375
H	-2.635517	0.612731	2.591152
H	-1.090226	1.429779	2.362569
H	-2.466711	1.837854	1.327090
C	-0.731530	-1.768906	1.684021
H	-1.491709	-2.127369	2.387013
H	-0.409988	-2.618249	1.076435
H	0.125241	-1.434485	2.278166
C	-2.840719	-0.912420	-0.495696
H	-0.971804	2.705684	-0.502532
C	-4.025717	-1.401025	0.365931
H	-4.847116	-1.732305	-0.282216
H	-3.752444	-2.248787	1.002745
H	-4.419268	-0.609201	1.011425
C	-3.311669	0.262739	-1.376904
H	-3.674858	1.105630	-0.780562
H	-2.511314	0.629603	-2.025815
H	-4.138839	-0.060803	-2.022269
C	-2.372236	-2.065426	-1.406966
H	-2.048045	-2.939131	-0.833136
H	-3.197050	-2.388426	-2.055547
H	-1.544845	-1.762301	-2.055564

SMD(MeCN)-LC-BLYP/6-311+G(d,p)

E = -987.384363
Zero-point correction = 0.372957 (Hartree/Particle)
Thermal correction to Energy = 0.394782
Thermal correction to Enthalpy = 0.395727
Thermal correction to Gibbs Free Energy = 0.321089
Sum of electronic and zero-point Energies = -987.011405
Sum of electronic and thermal Energies = -986.989580
Sum of electronic and thermal Enthalpies = -986.988636
Sum of electronic and thermal Free Energies = -987.063273

C	-0.083440	3.024449	-0.337330
C	0.989129	2.221111	-0.162536
H	1.954206	2.689339	-0.005775
C	0.958472	0.814900	-0.204350
O	-0.226269	0.205236	-0.430232
C	2.131733	-0.024235	-0.159558
C	3.390034	0.465521	0.213083
C	2.037827	-1.375281	-0.505760
C	4.490282	-0.359024	0.236227
H	3.509609	1.501543	0.501927
C	3.145614	-2.193598	-0.484282
H	1.079433	-1.775364	-0.807071
C	4.379966	-1.694843	-0.112584

H	5.451097	0.044166	0.535115
H	3.044570	-3.236059	-0.764219
H	5.250188	-2.339917	-0.093456
C	-0.024077	4.507550	-0.304489
H	-0.368318	4.936805	-1.249720
H	-0.679173	4.910461	0.473454
H	0.988719	4.866430	-0.116165
Si	-1.388975	-0.379200	0.640877
C	-1.917812	0.970057	1.796630
H	-2.573715	0.557818	2.567472
H	-1.056175	1.408356	2.305313
H	-2.456469	1.772518	1.291393
C	-0.675991	-1.772158	1.635136
H	-1.420979	-2.142527	2.344141
H	-0.354508	-2.610890	1.016953
H	0.183967	-1.432370	2.217048
C	-2.780800	-0.939722	-0.482259
H	-1.051896	2.571959	-0.521491
C	-3.953469	-1.407692	0.372639
H	-4.770342	-1.751374	-0.270191
H	-3.680660	-2.240857	1.025628
H	-4.347526	-0.604319	1.000129
C	-3.235029	0.215644	-1.365402
H	-3.593623	1.064255	-0.777773
H	-2.428707	0.569922	-2.010737
H	-4.059565	-0.107481	-2.009647
C	-2.312552	-2.089324	-1.365436
H	-2.009316	-2.959098	-0.777999
H	-3.125051	-2.408039	-2.026863
H	-1.469293	-1.798503	-1.996247

4-methoxyaniline

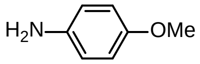

SMD(MeCN)-CAM-B3LYP/6-311+G(d,p)

E = -402.046061
Zero-point correction = 0.150114 (Hartree/Particle)
Thermal correction to Energy = 0.158503
Thermal correction to Enthalpy = 0.159447
Thermal correction to Gibbs Free Energy = 0.117168
Sum of electronic and zero-point Energies = -401.895947
Sum of electronic and thermal Energies = -401.887558
Sum of electronic and thermal Enthalpies = -401.886614
Sum of electronic and thermal Free Energies = -401.928893

C	0.069494	1.373008	-0.000467
C	0.942706	0.286240	0.000582
C	0.417800	-1.000568	-0.002941
C	-0.962506	-1.190908	-0.008798
C	-1.841681	-0.112299	-0.010705
C	-1.299850	1.177524	-0.007108
H	0.479903	2.376208	0.005421
H	1.064437	-1.867559	0.000297

H	-1.357373	-2.201440	-0.011520
H	-1.964554	2.035024	-0.008288
N	-3.229003	-0.306503	-0.075212
H	-3.541673	-1.190996	0.303724
H	-3.771757	0.458487	0.303878
O	2.276885	0.580450	0.007696
C	3.195857	-0.501244	0.005472
H	4.188597	-0.054944	0.013739
H	3.075143	-1.127631	0.894005
H	3.084305	-1.115743	-0.892557

SMD(MeCN)-B3LYP/6-311+G(d,p)

E = -402.256493
Zero-point correction = 0.148377 (Hartree/Particle)
Thermal correction to Energy = 0.156913
Thermal correction to Enthalpy = 0.157857
Thermal correction to Gibbs Free Energy = 0.115208
Sum of electronic and zero-point Energies = -402.108117
Sum of electronic and thermal Energies = -402.099581
Sum of electronic and thermal Enthalpies = -402.098636
Sum of electronic and thermal Free Energies = -402.141285

C	0.069701	1.377589	-0.000015
C	0.946878	0.285830	0.000462
C	0.416749	-1.006457	-0.001878
C	-0.968503	-1.196787	-0.006367
C	-1.852172	-0.112423	-0.008436
C	-1.305367	1.182434	-0.005299
H	0.479453	2.381829	0.005847
H	1.062579	-1.874831	0.001837
H	-1.362938	-2.208194	-0.007805
H	-1.968268	2.042083	-0.005615
N	-3.242326	-0.305965	-0.078495
H	-3.557838	-1.190531	0.300774
H	-3.786829	0.459716	0.299518
O	2.286277	0.582167	0.006544
C	3.214636	-0.502050	0.003718
H	4.204879	-0.048132	0.011675
H	3.097585	-1.129323	0.893217
H	3.105911	-1.117016	-0.895438

SMD(MeCN)-LC-BLYP/6-311+G(d,p)

E = -401.134707
Zero-point correction = 0.152197 (Hartree/Particle)
Thermal correction to Energy = 0.160424
Thermal correction to Enthalpy = 0.161368
Thermal correction to Gibbs Free Energy = 0.119473
Sum of electronic and zero-point Energies = -400.982510
Sum of electronic and thermal Energies = -400.974283
Sum of electronic and thermal Enthalpies = -400.973339
Sum of electronic and thermal Free Energies = -401.015234

C	0.067541	1.365647	-0.001222
C	0.936453	0.286522	0.000261
C	0.417668	-0.991449	-0.004503

C	-0.954567	-1.182213	-0.011305
C	-1.828187	-0.112787	-0.013064
C	-1.291744	1.170016	-0.008854
H	0.479432	2.368091	0.004815
H	1.065826	-1.857283	-0.001878
H	-1.349419	-2.192546	-0.015150
H	-1.958961	2.025379	-0.010601
N	-3.208155	-0.306489	-0.066662
H	-3.525551	-1.196212	0.295028
H	-3.757466	0.460667	0.297633
O	2.263461	0.576178	0.008427
C	3.172485	-0.497878	0.006911
H	4.168746	-0.061119	0.016556
H	3.048748	-1.125376	0.893589
H	3.060155	-1.112756	-0.890121

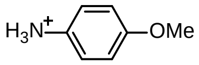

SMD(MeCN)-CAM-B3LYP/6-311+G(d,p)

E = -402.496278
Zero-point correction = 0.165212 (Hartree/Particle)
Thermal correction to Energy = 0.173838
Thermal correction to Enthalpy = 0.174782
Thermal correction to Gibbs Free Energy = 0.131600
Sum of electronic and zero-point Energies = -402.331066
Sum of electronic and thermal Energies = -402.322440
Sum of electronic and thermal Enthalpies = -402.321496
Sum of electronic and thermal Free Energies = -402.364678

C	-0.130837	1.385611	-0.003398
C	-0.991735	0.284972	-0.001602
C	-0.468296	-1.006912	-0.007834
C	0.909228	-1.190906	-0.011261
C	1.742244	-0.091698	-0.009606
C	1.237029	1.198854	-0.006658
H	-0.551662	2.383208	-0.003853
H	-1.114215	-1.873157	-0.012002
H	1.320882	-2.193580	-0.017432
H	1.906948	2.051063	-0.009149
N	3.196940	-0.289290	0.015535
H	3.475113	-1.120632	-0.515550
H	3.689678	0.510590	-0.394426
H	3.550934	-0.405329	0.971394
O	-2.312064	0.569361	0.003170
C	-3.243235	-0.509698	0.009284
H	-4.229262	-0.050779	0.022161
H	-3.138286	-1.122819	-0.888966
H	-3.118586	-1.129754	0.900177

SMD(MeCN)-B3LYP/6-311+G(d,p)

E = -402.708387
Zero-point correction = 0.163494 (Hartree/Particle)
Thermal correction to Energy = 0.172242

Thermal correction to Enthalpy = 0.173187
Thermal correction to Gibbs Free Energy = 0.129762
Sum of electronic and zero-point Energies = -402.544893
Sum of electronic and thermal Energies = -402.536144
Sum of electronic and thermal Enthalpies = -402.535200
Sum of electronic and thermal Free Energies = -402.578624

C	-0.131668	1.389907	-0.003438
C	-0.996068	0.284299	-0.001388
C	-0.467659	-1.012939	-0.007546
C	0.915012	-1.196997	-0.011076
C	1.750232	-0.091752	-0.010112
C	1.242134	1.203281	-0.006814
H	-0.551158	2.388679	-0.004216
H	-1.112735	-1.880439	-0.011809
H	1.326939	-2.200041	-0.017046
H	1.910803	2.056997	-0.009388
N	3.211965	-0.288328	0.015691
H	3.491511	-1.119855	-0.515369
H	3.703805	0.512370	-0.394872
H	3.567639	-0.403851	0.971821
O	-2.321289	0.571325	0.003354
C	-3.262501	-0.510497	0.009032
H	-4.245571	-0.043104	0.023240
H	-3.161324	-1.122834	-0.891257
H	-3.140251	-1.132038	0.900275

SMD(MeCN)-LC-BLYP/6-311+G(d,p)
E = -401.581557
Zero-point correction = 0.167156 (Hartree/Particle)
Thermal correction to Energy = 0.175647
Thermal correction to Enthalpy = 0.176591
Thermal correction to Gibbs Free Energy = 0.133569
Sum of electronic and zero-point Energies = -401.414402
Sum of electronic and thermal Energies = -401.405910
Sum of electronic and thermal Enthalpies = -401.404966
Sum of electronic and thermal Free Energies = -401.447988

C	-0.128645	1.378823	-0.003670
C	-0.984715	0.285547	-0.001962
C	-0.467640	-0.997952	-0.008164
C	0.901568	-1.181531	-0.011390
C	1.731487	-0.091795	-0.009010
C	1.229366	1.191772	-0.006663
H	-0.551360	2.375631	-0.004047
H	-1.115086	-1.863179	-0.012242
H	1.312758	-2.184650	-0.017680
H	1.901071	2.042893	-0.009137
N	3.175256	-0.290327	0.015555
H	3.452851	-1.124242	-0.514627
H	3.670856	0.508964	-0.395913
H	3.528803	-0.405392	0.973089
O	-2.298374	0.565101	0.002897
C	-3.219057	-0.506876	0.009727
H	-4.209054	-0.057902	0.022494

H	-3.112434	-1.121385	-0.886863
H	-3.092394	-1.127184	0.899651

aniline

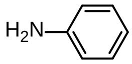

SMD(MeCN)-CAM-B3LYP/6-311+G(d,p)

E = -287.535878
Zero-point correction = 0.117603 (Hartree/Particle)
Thermal correction to Energy = 0.123343
Thermal correction to Enthalpy = 0.124287
Thermal correction to Gibbs Free Energy = 0.088496
Sum of electronic and zero-point Energies = -287.418275
Sum of electronic and thermal Energies = -287.412535
Sum of electronic and thermal Enthalpies = -287.411591
Sum of electronic and thermal Free Energies = -287.447382

C	-1.166701	-1.197590	0.003652
C	-1.874202	0.000000	0.008135
C	-1.166701	1.197590	0.003652
C	0.220485	1.203210	-0.006391
C	0.934756	0.000000	-0.010680
C	0.220485	-1.203210	-0.006391
H	-1.699645	-2.142008	0.009606
H	-2.957443	0.000000	0.016737
H	-1.699645	2.142008	0.009606
H	0.764064	2.142202	-0.009673
H	0.764064	-2.142202	-0.009673
N	2.324886	0.000000	-0.072997
H	2.772836	0.838479	0.271258
H	2.772836	-0.838479	0.271258

SMD(MeCN)-B3LYP/6-311+G(d,p)

E = -287.701806
Zero-point correction = 0.116366 (Hartree/Particle)
Thermal correction to Energy = 0.122167
Thermal correction to Enthalpy = 0.123111
Thermal correction to Gibbs Free Energy = 0.087219
Sum of electronic and zero-point Energies = -287.585441
Sum of electronic and thermal Energies = -287.579639
Sum of electronic and thermal Enthalpies = -287.578695
Sum of electronic and thermal Free Energies = -287.614588

C	-1.171336	-1.202605	0.003362
C	-1.881926	0.000000	0.006780
C	-1.171336	1.202605	0.003362
C	0.221269	1.208515	-0.004914
C	0.939996	0.000000	-0.008857
C	0.221269	-1.208515	-0.004914
H	-1.704428	-2.147743	0.009281
H	-2.965858	0.000000	0.014286
H	-1.704428	2.147743	0.009281
H	0.764105	2.148680	-0.006973
H	0.764105	-2.148680	-0.006973

N	2.333399	0.000000	-0.075571
H	2.782553	0.838379	0.270588
H	2.782553	-0.838379	0.270588

SMD(MeCN)-LC-BLYP/6-311+G(d,p)

E = -286.835044
Zero-point correction = 0.119040 (Hartree/Particle)
Thermal correction to Energy = 0.124730
Thermal correction to Enthalpy = 0.125675
Thermal correction to Gibbs Free Energy = 0.089976
Sum of electronic and zero-point Energies = -286.716004
Sum of electronic and thermal Energies = -286.710313
Sum of electronic and thermal Enthalpies = -286.709369
Sum of electronic and thermal Free Energies = -286.745068

C	-0.005745	-1.159444	-1.189659
C	-0.006370	-1.862807	0.000000
C	-0.005745	-1.159444	1.189659
C	-0.005745	0.218900	1.195282
C	-0.004968	0.927825	0.000000
C	-0.005745	0.218900	-1.195282
H	-0.004255	-1.692580	-2.133872
H	-0.005931	-2.945906	0.000000
H	-0.004255	-1.692580	2.133872
H	-0.005631	0.763386	2.133550
H	-0.005631	0.763386	-2.133550
N	-0.046354	2.310422	0.000000
H	0.278041	2.763878	0.843134
H	0.278041	2.763878	-0.843134

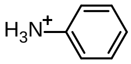

SMD(MeCN)-CAM-B3LYP/6-311+G(d,p)

E = -287.982217
Zero-point correction = 0.133014 (Hartree/Particle)
Thermal correction to Energy = 0.138869
Thermal correction to Enthalpy = 0.139813
Thermal correction to Gibbs Free Energy = 0.103526
Sum of electronic and zero-point Energies = -287.849203
Sum of electronic and thermal Energies = -287.843347
Sum of electronic and thermal Enthalpies = -287.842403
Sum of electronic and thermal Free Energies = -287.878690

C	0.004545	-1.220289	-1.204521
C	0.004030	-1.912728	0.000000
C	0.004545	-1.220289	1.204521
C	0.004545	0.168140	1.211287
C	0.004864	0.834403	0.000000
C	0.004545	0.168140	-1.211287
H	0.007321	-1.758763	-2.144166
H	0.006169	-2.996105	0.000000
H	0.007321	-1.758763	2.144166
H	0.007099	0.723137	2.142273
H	0.007099	0.723137	-2.142273
N	-0.017668	2.302921	0.000000

H	0.451961	2.688083	0.826263
H	0.451961	2.688083	-0.826263
H	-0.977695	2.666484	0.000000

<u>SMD(MeCN)-B3LYP/6-311+G(d,p)</u>

E = -288.149723
Zero-point correction = 0.131754 (Hartree/Particle)
Thermal correction to Energy = 0.137672
Thermal correction to Enthalpy = 0.138617
Thermal correction to Gibbs Free Energy = 0.102227
Sum of electronic and zero-point Energies = -288.017968
Sum of electronic and thermal Energies = -288.012050
Sum of electronic and thermal Enthalpies = -288.011106
Sum of electronic and thermal Free Energies = -288.047495

C	0.004579	-1.225483	-1.209274
C	0.004049	-1.920888	0.000000
C	0.004579	-1.225483	1.209274
C	0.004579	0.168735	1.216385
C	0.005126	0.837446	0.000000
C	0.004579	0.168735	-1.216385
H	0.007563	-1.763790	-2.149702
H	0.006308	-3.004863	0.000000
H	0.007563	-1.763790	2.149702
H	0.007067	0.723311	2.148115
H	0.007067	0.723311	-2.148115
N	-0.017996	2.313180	0.000000
H	0.451874	2.698624	0.826548
H	0.451874	2.698624	-0.826548
H	-0.978285	2.677939	0.000000

<u>SMD(MeCN)-LC-BLYP/6-311+G(d,p)</u>

E = -287.277928
Zero-point correction = 0.134448 (Hartree/Particle)
Thermal correction to Energy = 0.140220
Thermal correction to Enthalpy = 0.141164
Thermal correction to Gibbs Free Energy = 0.105028
Sum of electronic and zero-point Energies = -287.143480
Sum of electronic and thermal Energies = -287.137708
Sum of electronic and thermal Enthalpies = -287.136763
Sum of electronic and thermal Free Energies = -287.172899

C	1.212607	-1.196961	0.001304
C	1.900588	0.000000	0.005292
C	1.212607	1.196961	0.001304
C	-0.166817	1.203179	-0.005415
C	-0.830023	0.000000	-0.008600
C	-0.166817	-1.203179	-0.005415
H	1.751598	-2.136358	0.001358
H	2.983984	0.000000	0.008677
H	1.751598	2.136358	0.001358
H	-0.722239	2.134171	-0.010792
H	-0.722239	-2.134171	-0.010792
N	-2.287975	0.000000	0.006260
H	-2.672124	0.827462	-0.465320

H	-2.672124	-0.827462	-0.465320
H	-2.655501	0.000000	0.966202

4-bromoaniline

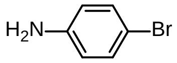

SMD(MeCN)-CAM-B3LYP/6-311+G(d,p)

E = -2861.182199
Zero-point correction = 0.107486 (Hartree/Particle)
Thermal correction to Energy = 0.114639
Thermal correction to Enthalpy = 0.115583
Thermal correction to Gibbs Free Energy = 0.075029
Sum of electronic and zero-point Energies = -2861.074712
Sum of electronic and thermal Energies = -2861.067560
Sum of electronic and thermal Enthalpies = -2861.066615
Sum of electronic and thermal Free Energies = -2861.107170

C	0.003243	0.366370	1.204072
C	0.004020	-0.320177	0.000000
C	0.003243	0.366370	-1.204072
C	0.003243	1.752546	-1.201606
C	0.002595	2.468320	0.000000
C	0.003243	1.752546	1.201606
H	0.001089	-0.167350	2.145896
H	0.001089	-0.167350	-2.145896
H	0.002554	2.290240	-2.143354
H	0.002554	2.290240	2.143354
N	0.053912	3.855346	0.000000
H	-0.286340	4.303801	-0.839794
H	-0.286340	4.303801	0.839794
Br	0.002014	-2.233047	0.000000

SMD(MeCN)-B3LYP/6-311+G(d,p)

E = -2861.246733
Zero-point correction = 0.106216 (Hartree/Particle)
Thermal correction to Energy = 0.113467
Thermal correction to Enthalpy = 0.114411
Thermal correction to Gibbs Free Energy = 0.073663
Sum of electronic and zero-point Energies = -2861.140517
Sum of electronic and thermal Energies = -2861.133267
Sum of electronic and thermal Enthalpies = -2861.132322
Sum of electronic and thermal Free Energies = -2861.173071

C	0.002907	0.371098	1.209818
C	0.003877	-0.317466	0.000000
C	0.002907	0.371098	-1.209818
C	0.002907	1.762849	-1.206609
C	0.002524	2.483659	0.000000
C	0.002907	1.762849	1.206609
H	0.000217	-0.162029	2.152544
H	0.000217	-0.162029	-2.152544
H	0.001990	2.298688	-2.150113
H	0.001990	2.298688	2.150113
N	0.060340	3.874000	0.000000

H	-0.281421	4.324057	-0.839651
H	-0.281421	4.324057	0.839651
Br	0.000797	-2.246970	0.000000

SMD(MeCN)-LC-BLYP/6-311+G(d,p)

E = -2859.979201
Zero-point correction = 0.108904 (Hartree/Particle)
Thermal correction to Energy = 0.115978
Thermal correction to Enthalpy = 0.116923
Thermal correction to Gibbs Free Energy = 0.076536
Sum of electronic and zero-point Energies = -2859.870296
Sum of electronic and thermal Energies = -2859.863222
Sum of electronic and thermal Enthalpies = -2859.862278
Sum of electronic and thermal Free Energies = -2859.902665

C	0.003368	0.360560	1.195008
C	0.003904	-0.325534	0.000000
C	0.003368	0.360560	-1.195008
C	0.003368	1.737284	-1.193968
C	0.002838	2.448296	0.000000
C	0.003368	1.737284	1.193968
H	0.001829	-0.174039	2.136809
H	0.001829	-0.174039	-2.136809
H	0.003350	2.276243	-2.134840
H	0.003350	2.276243	2.134840
N	0.044095	3.826756	0.000000
H	-0.266672	4.285784	-0.845100
H	-0.266672	4.285784	0.845100
Br	0.002658	-2.213542	0.000000

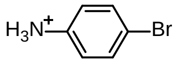

SMD(MeCN)-CAM-B3LYP/6-311+G(d,p)

E = -2861.625614
Zero-point correction = 0.122817 (Hartree/Particle)
Thermal correction to Energy = 0.130060
Thermal correction to Enthalpy = 0.131004
Thermal correction to Gibbs Free Energy = 0.090018
Sum of electronic and zero-point Energies = -2861.502797
Sum of electronic and thermal Energies = -2861.495554
Sum of electronic and thermal Enthalpies = -2861.494610
Sum of electronic and thermal Free Energies = -2861.535596

C	-0.001111	0.308709	1.210470
C	-0.000708	-0.366729	0.000000
C	-0.001111	0.308709	-1.210470
C	-0.001111	1.695909	-1.208830
C	-0.001967	2.364602	0.000000
C	-0.001111	1.695909	1.208830
H	-0.003633	-0.228052	2.149356
H	-0.003633	-0.228052	-2.149356
H	-0.003296	2.244735	-2.143034
H	-0.003296	2.244735	2.143034
N	0.019919	3.830990	0.000000
H	-0.449902	4.216251	-0.826412

H	-0.449902	4.216251	0.826412
H	0.980431	4.194279	0.000000
Br	-0.004671	-2.271992	0.000000

SMD(MeCN)-B3LYP/6-311+G(d,p)

E = -2861.691735
Zero-point correction = 0.121515 (Hartree/Particle)
Thermal correction to Energy = 0.128851
Thermal correction to Enthalpy = 0.129795
Thermal correction to Gibbs Free Energy = 0.088641
Sum of electronic and zero-point Energies = -2861.570221
Sum of electronic and thermal Energies = -2861.562884
Sum of electronic and thermal Enthalpies = -2861.561940
Sum of electronic and thermal Free Energies = -2861.603094

C	-0.001036	0.312676	1.216045
C	-0.000557	-0.365149	0.000000
C	-0.001036	0.312676	-1.216045
C	-0.001036	1.705570	-1.213652
C	-0.002152	2.377508	0.000000
C	-0.001036	1.705570	1.213652
H	-0.003755	-0.222927	2.156084
H	-0.003755	-0.222927	-2.156084
H	-0.003070	2.253065	-2.149092
H	-0.003070	2.253065	2.149092
N	0.020622	3.850735	0.000000
H	-0.449365	4.236486	-0.826656
H	-0.449365	4.236486	0.826656
H	0.981444	4.215210	0.000000
Br	-0.004923	-2.285620	0.000000

SMD(MeCN)-LC-BLYP/6-311+G(d,p)

E = -2860.419178
Zero-point correction = 0.124310 (Hartree/Particle)
Thermal correction to Energy = 0.131424
Thermal correction to Enthalpy = 0.132368
Thermal correction to Gibbs Free Energy = 0.091634
Sum of electronic and zero-point Energies = -2860.294868
Sum of electronic and thermal Energies = -2860.287754
Sum of electronic and thermal Enthalpies = -2860.286809
Sum of electronic and thermal Free Energies = -2860.327543

C	-0.001426	0.303175	1.202050
C	-0.001002	-0.370271	0.000000
C	-0.001426	0.303175	-1.202050
C	-0.001426	1.681096	-1.200984
C	-0.001897	2.346905	0.000000
C	-0.001426	1.681096	1.200984
H	-0.003831	-0.234894	2.140695
H	-0.003831	-0.234894	-2.140695
H	-0.003666	2.230803	-2.135037
H	-0.003666	2.230803	2.135037
N	0.019978	3.802765	0.000000
H	-0.449701	4.189744	-0.827618
H	-0.449701	4.189744	0.827618

H	0.982292	4.165280	0.000000
Br	-0.004461	-2.252200	0.000000

2,4-difluoroaniline

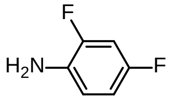

SMD(MeCN)-CAM-B3LYP/6-311+G(d,p)

E = -486.036059
Zero-point correction = 0.101108 (Hartree/Particle)
Thermal correction to Energy = 0.108476
Thermal correction to Enthalpy = 0.109420
Thermal correction to Gibbs Free Energy = 0.069452
Sum of electronic and zero-point Energies = -485.934951
Sum of electronic and thermal Energies = -485.927584
Sum of electronic and thermal Enthalpies = -485.926639
Sum of electronic and thermal Free Energies = -485.966607

C	1.176522	1.280330	-0.000671
C	1.582319	-0.036238	0.002036
C	0.687086	-1.085952	0.001597
C	-0.651514	-0.763707	-0.004763
C	-1.137757	0.540566	-0.008067
C	-0.183388	1.559536	-0.005271
H	1.909982	2.076624	0.002579
H	1.012675	-2.118065	0.006932
H	-0.520079	2.589741	-0.007008
N	-2.502272	0.792470	-0.072825
H	-3.094340	0.061903	0.299060
H	-2.776154	1.706793	0.261414
F	-1.564432	-1.771806	-0.003712
F	2.913788	-0.320583	0.007894

SMD(MeCN)-B3LYP/6-311+G(d,p)

E = -486.235061
Zero-point correction = 0.099890 (Hartree/Particle)
Thermal correction to Energy = 0.107350
Thermal correction to Enthalpy = 0.108295
Thermal correction to Gibbs Free Energy = 0.068156
Sum of electronic and zero-point Energies = -486.135171
Sum of electronic and thermal Energies = -486.127710
Sum of electronic and thermal Enthalpies = -486.126766
Sum of electronic and thermal Free Energies = -486.166904

C	-1.180634	-1.286620	-0.000552
C	-1.589254	0.035404	0.001667
C	-0.689514	1.089423	0.001632
C	0.655227	0.767896	-0.003874
C	1.144933	-0.542864	-0.006360
C	0.184756	-1.566156	-0.003859
H	-1.912718	-2.084917	0.002762
H	-1.015629	2.121860	0.006890
H	0.520774	-2.597169	-0.004615
N	2.511825	-0.798392	-0.075276

H	3.107376	-0.070109	0.298006
H	2.784558	-1.714195	0.258804
F	1.569561	1.783974	-0.003198
F	-2.927363	0.321668	0.006882

SMD(MeCN)-LC-BLYP/6-311+G(d,p)

E = -485.099421
Zero-point correction = 0.102527 (Hartree/Particle)
Thermal correction to Energy = 0.109796
Thermal correction to Enthalpy = 0.110740
Thermal correction to Gibbs Free Energy = 0.070962
Sum of electronic and zero-point Energies = -484.996895
Sum of electronic and thermal Energies = -484.989625
Sum of electronic and thermal Enthalpies = -484.988681
Sum of electronic and thermal Free Energies = -485.028459

C	1.170023	1.271065	-0.000902
C	1.573346	-0.037199	0.002406
C	0.682426	-1.079210	0.001581
C	-0.647098	-0.758407	-0.005310
C	-1.128183	0.538131	-0.009825
C	-0.181043	1.550046	-0.006706
H	1.905937	2.065357	0.002305
H	1.007508	-2.111818	0.006823
H	-0.518158	2.580111	-0.009534
N	-2.485902	0.784989	-0.065195
H	-3.084527	0.047717	0.281793
H	-2.770840	1.703118	0.248210
F	-1.557958	-1.756197	-0.004369
F	2.896244	-0.320019	0.008737

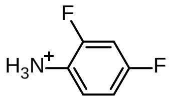

SMD(MeCN)-CAM-B3LYP/6-311+G(d,p)

E = -486.476773
Zero-point correction = 0.116352 (Hartree/Particle)
Thermal correction to Energy = 0.123760
Thermal correction to Enthalpy = 0.124704
Thermal correction to Gibbs Free Energy = 0.084486
Sum of electronic and zero-point Energies = -486.360421
Sum of electronic and thermal Energies = -486.353013
Sum of electronic and thermal Enthalpies = -486.352069
Sum of electronic and thermal Free Energies = -486.392287

C	-1.201303	-1.294640	-0.000028
C	-1.618316	0.020315	-0.000010
C	-0.750814	1.095341	0.000012
C	0.593472	0.802755	0.000008
C	1.056967	-0.500444	-0.000008
C	0.161270	-1.552213	-0.000023
H	-1.926348	-2.097448	-0.000048
H	-1.103308	2.118154	0.000033
H	0.527757	-2.571723	-0.000044

N	2.495971	-0.738572	0.000012
H	2.952736	-0.334836	0.827331
H	2.699867	-1.743858	-0.000220
H	2.952819	-0.334456	-0.827074
F	1.494315	1.800680	0.000022
F	-2.941312	0.277929	0.000003

<u>SMD(MeCN)-B3LYP/6-311+G(d,p)</u>

E = -486.677318
Zero-point correction = 0.115129 (Hartree/Particle)
Thermal correction to Energy = 0.122624
Thermal correction to Enthalpy = 0.123569
Thermal correction to Gibbs Free Energy = 0.083191
Sum of electronic and zero-point Energies = -486.562190
Sum of electronic and thermal Energies = -486.554694
Sum of electronic and thermal Enthalpies = -486.553750
Sum of electronic and thermal Free Energies = -486.594127

C	1.205052	-1.300991	0.000025
C	1.625712	0.019025	0.000043
C	0.754215	1.098385	0.000007
C	-0.596562	0.807270	0.000020
C	-1.062554	-0.501645	0.000009
C	-0.163214	-1.558038	-0.000027
H	1.927987	-2.106316	0.000027
H	1.107300	2.121424	-0.000021
H	-0.529796	-2.577918	-0.000033
N	-2.507588	-0.743885	-0.000004
H	-2.966327	-0.341471	-0.827557
H	-2.709264	-1.749949	0.000345
H	-2.966461	-0.340842	0.827172
F	-1.497770	1.813300	-0.000009
F	2.954857	0.277614	-0.000032

<u>SMD(MeCN)-LC-BLYP/6-311+G(d,p)</u>

E = -485.536671
Zero-point correction = 0.117719 (Hartree/Particle)
Thermal correction to Energy = 0.125023
Thermal correction to Enthalpy = 0.125968
Thermal correction to Gibbs Free Energy = 0.085938
Sum of electronic and zero-point Energies = -485.418952
Sum of electronic and thermal Energies = -485.411648
Sum of electronic and thermal Enthalpies = -485.410704
Sum of electronic and thermal Free Energies = -485.450734

C	-1.195478	-1.285223	-0.000037
C	-1.608202	0.021966	-0.000008
C	-0.745207	1.089674	0.000019
C	0.589368	0.796206	0.000016
C	1.049418	-0.499434	-0.000008
C	0.158161	-1.543237	-0.000033
H	-1.923982	-2.085283	-0.000063
H	-1.096963	2.113107	0.000043
H	0.523979	-2.563424	-0.000059
N	2.478734	-0.731717	0.000007

H	2.934990	-0.325348	0.828327
H	2.688085	-1.737657	-0.000166
H	2.935052	-0.325065	-0.828140
F	1.490011	1.783958	0.000042
F	-2.923418	0.278928	-0.000007

2-chloroaniline

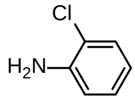

SMD(MeCN)-CAM-B3LYP/6-311+G(d,p)

E = -747.163010
Zero-point correction = 0.108388 (Hartree/Particle)
Thermal correction to Energy = 0.115200
Thermal correction to Enthalpy = 0.116144
Thermal correction to Gibbs Free Energy = 0.077195
Sum of electronic and zero-point Energies = -747.054622
Sum of electronic and thermal Energies = -747.047810
Sum of electronic and thermal Enthalpies = -747.046866
Sum of electronic and thermal Free Energies = -747.085815

C	2.347708	-0.005772	0.000211
C	1.825877	-1.295897	0.006356
C	0.450064	-1.466844	0.007217
C	-0.382754	-0.359758	-0.000338
C	0.114604	0.947361	-0.006055
C	1.508849	1.093883	-0.004699
H	3.420710	0.146339	0.001656
H	2.478883	-2.159454	0.011528
H	0.015933	-2.458806	0.013501
H	1.919745	2.097606	-0.007852
N	-0.711334	2.054422	-0.068633
H	-1.649403	1.943507	0.289701
H	-0.283877	2.924325	0.218104
Cl	-2.124043	-0.608960	-0.003668

SMD(MeCN)-B3LYP/6-311+G(d,p)

E = -747.325278
Zero-point correction = 0.107098 (Hartree/Particle)
Thermal correction to Energy = 0.114009
Thermal correction to Enthalpy = 0.114953
Thermal correction to Gibbs Free Energy = 0.075814
Sum of electronic and zero-point Energies = -747.218180
Sum of electronic and thermal Energies = -747.211269
Sum of electronic and thermal Enthalpies = -747.210325
Sum of electronic and thermal Free Energies = -747.249464

C	2.358817	-0.011309	0.001116
C	1.831430	-1.304929	0.004959
C	0.448753	-1.473693	0.005478
C	-0.383392	-0.359947	-0.000741
C	0.118054	0.953449	-0.005159
C	1.519512	1.095130	-0.002517
H	3.432840	0.138259	0.003801

H	2.481978	-2.171198	0.009091
H	0.013059	-2.465470	0.010917
H	1.933983	2.098079	-0.003551
N	-0.706206	2.065768	-0.072442
H	-1.645999	1.956150	0.284783
H	-0.278163	2.933908	0.222260
Cl	-2.138430	-0.608371	-0.002295

SMD(MeCN)-LC-BLYP/6-311+G(d,p)

E = -746.199571
Zero-point correction = 0.109863 (Hartree/Particle)
Thermal correction to Energy = 0.116589
Thermal correction to Enthalpy = 0.117533
Thermal correction to Gibbs Free Energy = 0.078759
Sum of electronic and zero-point Energies = -746.089707
Sum of electronic and thermal Energies = -746.082982
Sum of electronic and thermal Enthalpies = -746.082038
Sum of electronic and thermal Free Energies = -746.120811

C	2.331047	0.000972	-0.000458
C	1.817049	-1.283472	0.007277
C	0.451295	-1.455954	0.008329
C	-0.382201	-0.359432	0.000047
C	0.110023	0.939224	-0.007106
C	1.494384	1.090679	-0.006433
H	3.403558	0.156309	0.000038
H	2.473384	-2.144267	0.013092
H	0.019409	-2.449157	0.015083
H	1.901336	2.095804	-0.011367
N	-0.715097	2.037137	-0.058992
H	-1.663729	1.929196	0.270594
H	-0.292844	2.919311	0.195397
Cl	-2.103825	-0.609368	-0.004696

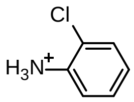

SMD(MeCN)-CAM-B3LYP/6-311+G(d,p)

E = -747.602424
Zero-point correction = 0.123187 (Hartree/Particle)
Thermal correction to Energy = 0.130257
Thermal correction to Enthalpy = 0.131201
Thermal correction to Gibbs Free Energy = 0.091367
Sum of electronic and zero-point Energies = -747.479237
Sum of electronic and thermal Energies = -747.472168
Sum of electronic and thermal Enthalpies = -747.471224
Sum of electronic and thermal Free Energies = -747.511057

C	-2.369210	0.040433	0.004090
C	-1.877832	-1.256979	-0.002064
C	-0.510008	-1.491791	-0.004443
C	0.365616	-0.419530	-0.001695
C	-0.128322	0.877598	-0.001703
C	-1.490402	1.112906	0.003417

H	-3.435977	0.223522	0.007801
H	-2.559824	-2.098101	-0.004903
H	-0.121242	-2.501581	-0.008609
H	-1.855059	2.133104	0.006723
N	0.786951	2.018233	-0.003808
H	1.468368	1.973602	-0.770674
H	0.266403	2.895310	-0.118500
H	1.314653	2.098736	0.874113
Cl	2.086763	-0.707532	0.003241

SMD(MeCN)-B3LYP/6-311+G(d,p)

E = -747.766337
Zero-point correction = 0.122144 (Hartree/Particle)
Thermal correction to Energy = 0.129178
Thermal correction to Enthalpy = 0.130123
Thermal correction to Gibbs Free Energy = 0.090528
Sum of electronic and zero-point Energies = -747.644193
Sum of electronic and thermal Energies = -747.637158
Sum of electronic and thermal Enthalpies = -747.636214
Sum of electronic and thermal Free Energies = -747.675809

C	-2.379762	0.032505	0.002531
C	-1.881515	-1.268225	-0.000419
C	-0.507310	-1.499300	-0.002207
C	0.368282	-0.419264	-0.001022
C	-0.132339	0.881927	-0.002245
C	-1.501372	1.112341	0.000764
H	-3.447648	0.212282	0.004515
H	-2.560295	-2.112714	-0.001620
H	-0.115766	-2.508448	-0.004484
H	-1.870233	2.131578	0.001378
N	0.779269	2.034032	-0.000986
H	1.474390	1.987629	-0.755766
H	0.253657	2.905924	-0.133021
H	1.291979	2.127835	0.885171
Cl	2.101361	-0.707189	0.001548

SMD(MeCN)-LC-BLYP/6-311+G(d,p)

E = -746.635447
Zero-point correction = 0.124324 (Hartree/Particle)
Thermal correction to Energy = 0.131415
Thermal correction to Enthalpy = 0.132359
Thermal correction to Gibbs Free Energy = 0.092203
Sum of electronic and zero-point Energies = -746.511124
Sum of electronic and thermal Energies = -746.504032
Sum of electronic and thermal Enthalpies = -746.503088
Sum of electronic and thermal Free Energie = -746.543245

C	-2.353238	0.057337	-0.000140
C	-1.875566	-1.235664	-0.000058
C	-0.518125	-1.479326	0.000204
C	0.359085	-0.420755	0.000128
C	-0.121097	0.872130	0.000062
C	-1.471902	1.117107	0.000094
H	-3.418832	0.247892	-0.000610

H	-2.564759	-2.071003	-0.000223
H	-0.135827	-2.491915	0.000513
H	-1.830765	2.139269	0.000403
N	0.806393	1.989429	0.000004
H	1.422728	1.984839	-0.823770
H	0.301512	2.885297	-0.009728
H	1.410078	1.995763	0.833198
Cl	2.062128	-0.710654	-0.000091

2,5-dichloroaniline

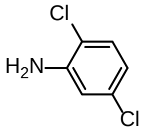

SMD(MeCN)-CAM-B3LYP/6-311+G(d,p)

E = -1206.789791
Zero-point correction = 0.098586 (Hartree/Particle)
Thermal correction to Energy = 0.106656
Thermal correction to Enthalpy = 0.107600
Thermal correction to Gibbs Free Energy = 0.065176
Sum of electronic and zero-point Energies = -1206.691205
Sum of electronic and thermal Energies = -1206.683135
Sum of electronic and thermal Enthalpies = -1206.682191
Sum of electronic and thermal Free Energies = -1206.724615

C	1.522504	-0.120205	-0.000867
C	0.967681	-1.390561	0.003191
C	-0.414900	-1.492718	0.004854
C	-1.202048	-0.355396	-0.000263
C	-0.646905	0.930428	-0.005755
C	0.751494	1.023548	-0.003712
H	1.588561	-2.275714	0.005852
H	-0.884039	-2.468248	0.009733
H	1.212354	2.003372	-0.004890
N	-1.418631	2.068470	-0.066572
H	-2.370167	2.003230	0.265237
H	-0.958222	2.927026	0.201505
Cl	-2.948114	-0.529097	-0.001393
Cl	3.270171	0.044419	0.001620

SMD(MeCN)-B3LYP/6-311+G(d,p)

E = -1206.948613
Zero-point correction = 0.097331 (Hartree/Particle)
Thermal correction to Energy = 0.105512
Thermal correction to Enthalpy = 0.106456
Thermal correction to Gibbs Free Energy = 0.063818
Sum of electronic and zero-point Energies = -1206.851282
Sum of electronic and thermal Energies = -1206.843101
Sum of electronic and thermal Enthalpies = -1206.842157
Sum of electronic and thermal Free Energies = -1206.884794

C	1.527887	-0.121403	-0.001245
C	0.970822	-1.396743	0.000813
C	-0.418295	-1.498173	0.002835

C	-1.207051	-0.355849	-0.000721
C	-0.649754	0.936998	-0.005278
C	0.755745	1.028218	-0.002177
H	1.589794	-2.283766	0.002048
H	-0.886563	-2.474630	0.007051
H	1.216904	2.008371	-0.001009
N	-1.421405	2.078829	-0.070322
H	-2.373661	2.014044	0.262669
H	-0.961028	2.937075	0.202341
Cl	-2.965791	-0.531526	0.000532
Cl	3.288630	0.042633	0.002632

SMD(MeCN)-LC-BLYP/6-311+G(d,p)

E = -1205.563480
Zero-point correction = 0.100111 (Hartree/Particle)
Thermal correction to Energy = 0.108056
Thermal correction to Enthalpy = 0.109000
Thermal correction to Gibbs Free Energy = 0.066831
Sum of electronic and zero-point Energies = -1205.463369
Sum of electronic and thermal Energies = -1205.455424
Sum of electronic and thermal Enthalpies = -1205.454480
Sum of electronic and thermal Free Energies = -1205.496649

C	1.514556	-0.118301	-0.000589
C	0.962743	-1.381363	0.004799
C	-0.409840	-1.483448	0.006163
C	-1.195159	-0.354677	0.000275
C	-0.643057	0.921917	-0.006388
C	0.745793	1.016105	-0.004802
H	1.585743	-2.265245	0.008271
H	-0.879801	-2.458926	0.011249
H	1.205986	1.996486	-0.007544
N	-1.411721	2.052338	-0.055551
H	-2.374828	1.993157	0.241559
H	-0.954220	2.923179	0.174714
Cl	-2.923003	-0.526123	-0.002920
Cl	3.243530	0.046334	0.000794

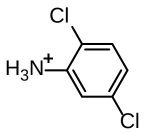

SMD(MeCN)-CAM-B3LYP/6-311+G(d,p)

E = -1207.225276
Zero-point correction = 0.113658 (Hartree/Particle)
Thermal correction to Energy = 0.121921
Thermal correction to Enthalpy = 0.122866
Thermal correction to Gibbs Free Energy = 0.079583
Sum of electronic and zero-point Energies = -1207.111617
Sum of electronic and thermal Energies = -1207.103354
Sum of electronic and thermal Enthalpies = -1207.102410
Sum of electronic and thermal Free Energies = -1207.145693

C	-1.556158	-0.121960	0.000000

C	-1.003175	-1.391305	0.002400
C	0.375365	-1.534315	0.002014
C	1.186811	-0.413027	0.000229
C	0.612642	0.850134	-0.001372
C	-0.759263	1.009952	-0.002093
H	-1.636720	-2.268366	0.004066
H	0.817444	-2.521666	0.003125
H	-1.188818	2.003870	-0.004317
N	1.455582	2.041798	0.002816
H	2.107693	2.063418	-0.790296
H	0.884299	2.892601	-0.059686
H	2.012236	2.122080	0.862842
Cl	2.919357	-0.591500	-0.001739
Cl	-3.291271	0.063182	-0.000762

SMD(MeCN)-B3LYP/6-311+G(d,p)

E = -1207.385693
Zero-point correction = 0.112491 (Hartree/Particle)
Thermal correction to Energy = 0.120799
Thermal correction to Enthalpy = 0.121743
Thermal correction to Gibbs Free Energy = 0.078508
Sum of electronic and zero-point Energies = -1207.273202
Sum of electronic and thermal Energies = -1207.264895
Sum of electronic and thermal Enthalpies = -1207.263950
Sum of electronic and thermal Free Energies = -1207.307185

C	-1.561883	-0.123471	0.000522
C	-1.005975	-1.397939	0.005596
C	0.378343	-1.539817	0.005237
C	1.192639	-0.413105	0.001356
C	0.614974	0.855194	-0.000782
C	-0.763182	1.014301	-0.002434
H	-1.637629	-2.276827	0.008712
H	0.820252	-2.527700	0.007703
H	-1.192940	2.008404	-0.006421
N	1.457681	2.055245	0.005401
H	2.121330	2.073326	-0.778727
H	0.883521	2.903150	-0.072294
H	2.002439	2.145201	0.872809
Cl	2.935765	-0.595331	-0.004679
Cl	-3.308132	0.061023	-0.002766

SMD(MeCN)-LC-BLYP/6-311+G(d,p)

E = -1205.995438
Zero-point correction = 0.115072 (Hartree/Particle)
Thermal correction to Energy = 0.123196
Thermal correction to Enthalpy = 0.124141
Thermal correction to Gibbs Free Energy = 0.081127
Sum of electronic and zero-point Energies = -1205.880366
Sum of electronic and thermal Energies = -1205.872242
Sum of electronic and thermal Enthalpies = -1205.871298
Sum of electronic and thermal Free Energies = -1205.914312

C	-1.548145	-0.119794	0.000039
C	-0.999051	-1.381178	0.000057

C	0.370507	-1.525629	0.000076
C	1.178259	-0.412976	0.000044
C	0.609135	0.842907	0.000069
C	-0.753289	1.002987	0.000055
H	-1.635193	-2.256834	0.000063
H	0.812453	-2.513423	0.000094
H	-1.182833	1.997467	0.000059
N	1.453009	2.021503	0.000020
H	2.059403	2.067107	-0.829909
H	0.886718	2.879987	0.001037
H	2.060983	2.066126	0.828834
Cl	2.894956	-0.586026	-0.000072
Cl	-3.266550	0.066682	-0.000066

2,6-dichloroaniline

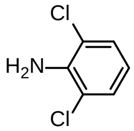

SMD(MeCN)-CAM-B3LYP/6-311+G(d,p)

E = -1206.787930
Zero-point correction = 0.099057 (Hartree/Particle)
Thermal correction to Energy = 0.107015
Thermal correction to Enthalpy = 0.107959
Thermal correction to Gibbs Free Energy = 0.065841
Sum of electronic and zero-point Energies = -1206.688873
Sum of electronic and thermal Energies = -1206.680915
Sum of electronic and thermal Enthalpies = -1206.679971
Sum of electronic and thermal Free Energies = -1206.722090

C	-1.197830	1.598030	0.004812
C	0.000002	2.297177	0.007189
C	1.197833	1.598026	0.004753
C	1.185906	0.216144	0.001502
C	-0.000002	-0.533717	0.001201
C	-1.185906	0.216147	0.001553
H	-2.144304	2.123188	0.005876
H	0.000005	3.379371	0.009918
H	2.144307	2.123184	0.005774
N	-0.000016	-1.906335	-0.054166
H	0.843495	-2.374134	0.244641
H	-0.843431	-2.374136	0.244909
Cl	2.715577	-0.643647	-0.007652
Cl	-2.715576	-0.643645	-0.007526

SMD(MeCN)-B3LYP/6-311+G(d,p)

E = -1206.946518
Zero-point correction = 0.097696 (Hartree/Particle)
Thermal correction to Energy = 0.105792
Thermal correction to Enthalpy = 0.106736
Thermal correction to Gibbs Free Energy = 0.064344
Sum of electronic and zero-point Energies = -1206.848821
Sum of electronic and thermal Energies = -1206.840726

Sum of electronic and thermal Enthalpies = -1206.839782
Sum of electronic and thermal Free Energies = -1206.882174

C	-1.203572	1.605180	0.004779
C	-0.000019	2.306250	0.007087
C	1.203560	1.605144	0.004760
C	1.190575	0.218016	0.001755
C	-0.000006	-0.538093	0.001718
C	-1.190519	0.218033	0.001702
H	-2.149891	2.131445	0.006084
H	0.000105	3.389126	0.009771
H	2.149836	2.131485	0.006069
N	-0.000012	-1.913933	-0.057275
H	0.843732	-2.380736	0.245908
H	-0.843341	-2.380789	0.246984
Cl	2.732388	-0.646468	-0.007191
Cl	-2.732416	-0.646484	-0.007204

<u>SMD(MeCN)-LC-BLYP/6-311+G(d,p)</u>
E = -1205.561920
Zero-point correction = 0.100561 (Hartree/Particle)
Thermal correction to Energy = 0.108417
Thermal correction to Enthalpy = 0.109361
Thermal correction to Gibbs Free Energy = 0.067453
Sum of electronic and zero-point Energies = -1205.461360
Sum of electronic and thermal Energies = -1205.453503
Sum of electronic and thermal Enthalpies = -1205.452559
Sum of electronic and thermal Free Energies = -1205.494467

C	-1.188988	1.586947	0.005069
C	0.000003	2.283254	0.007650
C	1.188991	1.586946	0.005069
C	1.179409	0.213618	0.001434
C	0.000000	-0.528671	0.000110
C	-1.179409	0.213621	0.001437
H	-2.136120	2.111563	0.006027
H	0.000002	3.365302	0.010623
H	2.136125	2.111559	0.006027
N	-0.000002	-1.893116	-0.043835
H	0.848776	-2.374696	0.214584
H	-0.848806	-2.374690	0.214508
Cl	2.692176	-0.638870	-0.007928
Cl	-2.692176	-0.638866	-0.007927

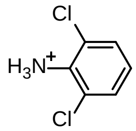

<u>SMD(MeCN)-CAM-B3LYP/6-311+G(d,p)</u>
E = -1207.218880
Zero-point correction = 0.114047 (Hartree/Particle)
Thermal correction to Energy = 0.122076
Thermal correction to Enthalpy = 0.123020
Thermal correction to Gibbs Free Energy = 0.080576

Sum of electronic and zero-point Energies = -1207.104833
Sum of electronic and thermal Energies = -1207.096804
Sum of electronic and thermal Enthalpies = -1207.095860
Sum of electronic and thermal Free Energies = -1207.138304

C	1.211292	1.620980	-0.000021
C	0.010514	2.310926	0.000009
C	-1.197952	1.632308	0.000030
C	-1.197630	0.249479	0.000014
C	-0.001994	-0.460453	0.000021
C	1.199688	0.237267	0.000002
H	2.154788	2.149873	-0.000060
H	0.016433	3.393433	0.000003
H	-2.136340	2.170291	0.000046
N	-0.022350	-1.917365	0.000024
H	-0.495612	-2.299278	-0.829480
H	0.925595	-2.313199	0.000004
H	-0.495557	-2.299279	0.829566
Cl	-2.713509	-0.609134	-0.000025
Cl	2.716076	-0.621650	-0.000010

SMD(MeCN)-B3LYP/6-311+G(d,p)

E = -1207.379288
Zero-point correction = 0.112672 (Hartree/Particle)
Thermal correction to Energy = 0.120835
Thermal correction to Enthalpy = 0.121779
Thermal correction to Gibbs Free Energy = 0.079036
Sum of electronic and zero-point Energies = -1207.266616
Sum of electronic and thermal Energies = -1207.258453
Sum of electronic and thermal Enthalpies = -1207.257508
Sum of electronic and thermal Free Energies = -1207.300252

C	1.216600	1.628502	-0.000031
C	0.010203	2.320185	0.000007
C	-1.203602	1.639500	0.000029
C	-1.202912	0.250776	0.000009
C	-0.002109	-0.462978	0.000005
C	1.204545	0.239122	-0.000032
H	2.159948	2.158389	-0.000068
H	0.016012	3.403309	0.000024
H	-2.141845	2.178451	0.000073
N	-0.021082	-1.926104	0.000007
H	-0.493993	-2.308957	-0.829728
H	0.928575	-2.319213	0.000130
H	-0.494203	-2.308960	0.829613
Cl	-2.729446	-0.612001	-0.000012
Cl	2.731607	-0.623936	0.000011

SMD(MeCN)-LC-BLYP/6-311+G(d,p)

E = -1205.989072
Zero-point correction = 0.115549 (Hartree/Particle)
Thermal correction to Energy = 0.123439
Thermal correction to Enthalpy = 0.124383
Thermal correction to Gibbs Free Energy = 0.082205
Sum of electronic and zero-point Energies = -1205.873523

Sum of electronic and thermal Energies = -1205.865633
Sum of electronic and thermal Enthalpies = -1205.864689
Sum of electronic and thermal Free Energies = -1205.906867

C	1.203341	1.609311	0.000070
C	0.011300	2.296338	-0.000098
C	-1.189161	1.621526	-0.000149
C	-1.189885	0.247668	0.000035
C	-0.001926	-0.457355	-0.000066
C	1.192638	0.234089	-0.000043
H	2.147516	2.137773	0.000143
H	0.017505	3.378961	-0.000184
H	-2.128086	2.159332	-0.000226
N	-0.025190	-1.904736	-0.000266
H	-0.499985	-2.286796	-0.831039
H	0.921826	-2.306683	-0.000361
H	-0.499911	-2.287108	0.830389
Cl	-2.690364	-0.604028	0.000158
Cl	2.693872	-0.617842	0.000116

2-Amino-1,1,3,3-tetracyano-1-propene

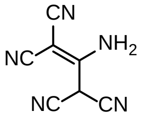

SMD(MeCN)-CAM-B3LYP/6-311+G(d,p)
E = -542.128103
Zero-point correction = 0.094384 (Hartree/Particle)
Thermal correction to Energy = 0.105806
Thermal correction to Enthalpy = 0.106750
Thermal correction to Gibbs Free Energy = 0.055586
Sum of electronic and zero-point Energies = -542.033719
Sum of electronic and thermal Energies = -542.022297
Sum of electronic and thermal Enthalpies = -542.021353
Sum of electronic and thermal Free Energies = -542.072517

C	-1.171081	0.340891	-0.115866
H	-0.911297	1.344459	-0.467064
C	0.103870	-0.491921	0.043205
C	1.335128	0.120515	-0.076315
C	1.481435	1.498512	-0.365939
N	1.615046	2.617529	-0.600301
C	2.517404	-0.640723	0.105229
N	3.455888	-1.289164	0.256984
C	-2.077032	-0.233549	-1.116561
N	-2.774658	-0.670046	-1.911524
C	-1.849301	0.500217	1.176186
N	-2.362819	0.640448	2.189094
N	-0.059745	-1.773961	0.311088
H	-0.978221	-2.188184	0.407976
H	0.730998	-2.393580	0.442060

SMD(MeCN)-B3LYP/6-311+G(d,p)
E = -542.391373
Zero-point correction = 0.092640 (Hartree/Particle)

Thermal correction to Energy = 0.104286
Thermal correction to Enthalpy = 0.105231
Thermal correction to Gibbs Free Energy = 0.053033
Sum of electronic and zero-point Energies = -542.298733
Sum of electronic and thermal Energies = -542.287086
Sum of electronic and thermal Enthalpies = -542.286142
Sum of electronic and thermal Free Energies = -542.338339

C	-1.178417	0.336353	-0.113828
H	-0.910877	1.340603	-0.461829
C	0.102665	-0.500492	0.048924
C	1.340947	0.117794	-0.073311
C	1.486475	1.496390	-0.361942
N	1.627959	2.621614	-0.596052
C	2.528671	-0.636913	0.104020
N	3.481159	-1.277977	0.251291
C	-2.083241	-0.231048	-1.124272
N	-2.782157	-0.660269	-1.930735
C	-1.865231	0.498881	1.176914
N	-2.386592	0.643926	2.192030
N	-0.059570	-1.786964	0.319992
H	-0.977829	-2.202495	0.415566
H	0.731910	-2.406204	0.451552

<u>SMD(MeCN)-LC-BLYP/6-311+G(d,p)</u>

E = -540.929643
Zero-point correction = 0.096502 (Hartree/Particle)
Thermal correction to Energy = 0.107685
Thermal correction to Enthalpy = 0.108629
Thermal correction to Gibbs Free Energy = 0.057972
Sum of electronic and zero-point Energies = -540.833141
Sum of electronic and thermal Energies = -540.821958
Sum of electronic and thermal Enthalpies = -540.821014
Sum of electronic and thermal Free Energies = -540.871671

C	-1.161448	0.345513	-0.119898
H	-0.908695	1.347126	-0.480727
C	0.102891	-0.483123	0.036667
C	1.325377	0.120566	-0.080758
C	1.474551	1.493988	-0.374994
N	1.603806	2.603411	-0.612461
C	2.499239	-0.645629	0.107700
N	3.423392	-1.297036	0.265643
C	-2.069709	-0.237850	-1.102680
N	-2.767514	-0.684678	-1.879368
C	-1.824130	0.509154	1.171550
N	-2.325008	0.649283	2.181230
N	-0.064833	-1.758274	0.304069
H	-0.985707	-2.170555	0.404281
H	0.724875	-2.381228	0.437128

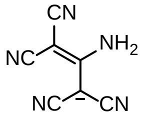

<u>SMD(MeCN)-CAM-B3LYP/6-311+G(d,p)</u>

E = -541.696925
Zero-point correction = 0.081151 (Hartree/Particle)
Thermal correction to Energy = 0.092525
Thermal correction to Enthalpy = 0.093469
Thermal correction to Gibbs Free Energy = 0.042744
Sum of electronic and zero-point Energies = -541.615775
Sum of electronic and thermal Energies = -541.604401
Sum of electronic and thermal Enthalpies = -541.603457
Sum of electronic and thermal Free Energies = -541.654181

C	1.248829	0.000349	0.039043
C	0.000000	0.676968	0.000000
C	-1.248829	0.000349	-0.039042
C	-1.385433	-1.337215	-0.450577
N	-1.536537	-2.423730	-0.813485
C	-2.439491	0.708874	0.207866
N	-3.410816	1.300463	0.413945
C	2.439491	0.708873	-0.207867
N	3.410816	1.300463	-0.413944
C	1.385433	-1.337215	0.450579
N	1.536537	-2.423730	0.813484
N	0.000000	2.016319	-0.000001
H	0.845560	2.542808	0.163323
H	-0.845560	2.542808	-0.163324

SMD(MeCN)-B3LYP/6-311+G(d,p)
E = -541.959797
Zero-point correction = 0.079759 (Hartree/Particle)
Thermal correction to Energy = 0.091271
Thermal correction to Enthalpy = 0.092215
Thermal correction to Gibbs Free Energy = 0.041437
Sum of electronic and zero-point Energies = -541.880038
Sum of electronic and thermal Energies = -541.868526
Sum of electronic and thermal Enthalpies = -541.867582
Sum of electronic and thermal Free Energies = -541.918360

C	-1.255279	0.002076	-0.043827
C	0.000001	0.681341	-0.000001
C	1.255279	0.002075	0.043827
C	1.395920	-1.334925	0.460102
N	1.559058	-2.425682	0.827253
C	2.448301	0.706138	-0.212492
N	3.429146	1.292811	-0.426499
C	-2.448300	0.706142	0.212487
N	-3.429143	1.292812	0.426509
C	-1.395921	-1.334926	-0.460095
N	-1.559062	-2.425678	-0.827258
N	0.000001	2.026551	-0.000004
H	-0.843570	2.553390	-0.174726
H	0.843571	2.553390	0.174717

SMD(MeCN)-LC-BLYP/6-311+G(d,p)
E = -540.501627
Zero-point correction = 0.083059 (Hartree/Particle)
Thermal correction to Energy = 0.094133

Thermal correction to Enthalpy = 0.095077
Thermal correction to Gibbs Free Energy = 0.045333
Sum of electronic and zero-point Energies = -540.418568
Sum of electronic and thermal Energies = -540.407495
Sum of electronic and thermal Enthalpies = -540.406551
Sum of electronic and thermal Free Energies = -540.456294

C	1.242323	-0.001123	0.025024
C	0.000000	0.667523	-0.000001
C	-1.242323	-0.001122	-0.025024
C	-1.386187	-1.351380	-0.374439
N	-1.540112	-2.445029	-0.680348
C	-2.425083	0.725718	0.177982
N	-3.382028	1.334924	0.344482
C	2.425083	0.725717	-0.177985
N	3.382028	1.334924	-0.344478
C	1.386187	-1.351379	0.374442
N	1.540111	-2.445030	0.680346
N	0.000000	2.000195	-0.000002
H	0.852146	2.528190	0.128578
H	-0.852145	2.528190	-0.128581

1,1,3,3-tetracyano-2-methyl-1-propene

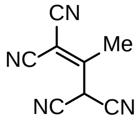

SMD(MeCN)-CAM-B3LYP/6-311+G(d,p)

E = -526.056754
Zero-point correction = 0.104630 (Hartree/Particle)
Thermal correction to Energy = 0.116430
Thermal correction to Enthalpy = 0.117374
Thermal correction to Gibbs Free Energy = 0.065874
Sum of electronic and zero-point Energies = -525.952124
Sum of electronic and thermal Energies = -525.940324
Sum of electronic and thermal Enthalpies = -525.939380
Sum of electronic and thermal Free Energies = -525.990880

C	-1.160448	0.347564	-0.182743
H	-0.909848	1.278924	-0.702082
C	0.095949	-0.492884	0.061066
C	1.302679	0.082043	-0.098757
C	1.478126	1.443244	-0.505485
N	1.628963	2.533979	-0.828238
C	2.514620	-0.641513	0.143349
N	3.497787	-1.202943	0.328698
C	-2.128901	-0.357957	-1.030953
N	-2.873338	-0.896925	-1.712840
C	-1.779022	0.726184	1.095154
N	-2.241161	1.036752	2.095201
C	-0.100150	-1.902545	0.480863
H	-0.559051	-2.466382	-0.336985
H	0.835125	-2.384876	0.755539
H	-0.789114	-1.948534	1.328823

SMD(MeCN)-B3LYP/6-311+G(d,p)

E = -526.322511
Zero-point correction = 0.102865 (Hartree/Particle)
Thermal correction to Energy = 0.114789
Thermal correction to Enthalpy = 0.115733
Thermal correction to Gibbs Free Energy = 0.063992
Sum of electronic and zero-point Energies = -526.219646
Sum of electronic and thermal Energies = -526.207722
Sum of electronic and thermal Enthalpies = -526.206778
Sum of electronic and thermal Free Energies = -526.258520

C	-1.168806	0.344787	-0.176741
H	-0.910320	1.281311	-0.686123
C	0.093809	-0.502023	0.064565
C	1.308198	0.080717	-0.096786
C	1.485180	1.442812	-0.498986
N	1.646336	2.539654	-0.820157
C	2.523497	-0.638575	0.140456
N	3.518649	-1.193866	0.321938
C	-2.137010	-0.347452	-1.041155
N	-2.883395	-0.875016	-1.739816
C	-1.794049	0.717234	1.103379
N	-2.262038	1.026709	2.108024
C	-0.097697	-1.916663	0.480585
H	-0.564549	-2.479899	-0.335452
H	0.840868	-2.400034	0.745536
H	-0.781602	-1.968757	1.334212

<u>SMD(MeCN)-LC-BLYP/6-311+G(d,p)</u>
E = -524.862116
Zero-point correction = 0.107001 (Hartree/Particle)
Thermal correction to Energy = 0.118618
Thermal correction to Enthalpy = 0.119562
Thermal correction to Gibbs Free Energy = 0.068244
Sum of electronic and zero-point Energies = -524.755115
Sum of electronic and thermal Energies = -524.743498
Sum of electronic and thermal Enthalpies = -524.742553
Sum of electronic and thermal Free Energies = -524.793872

C	-1.150545	0.351779	-0.190480
H	-0.907992	1.277441	-0.722682
C	0.096039	-0.481301	0.055326
C	1.294276	0.082041	-0.101490
C	1.471702	1.438540	-0.512067
N	1.616741	2.520161	-0.836180
C	2.497645	-0.647636	0.146459
N	3.464894	-1.216820	0.336418
C	-2.119108	-0.365946	-1.015843
N	-2.861911	-0.916915	-1.675387
C	-1.755484	0.737935	1.083193
N	-2.206910	1.049386	2.078164
C	-0.107810	-1.881244	0.478183
H	-0.555967	-2.446793	-0.342624
H	0.821174	-2.365397	0.768110
H	-0.807205	-1.920940	1.316416

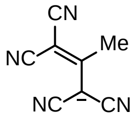

SMD(MeCN)-CAM-B3LYP/6-311+G(d,p)

E = -525.630755
Zero-point correction = 0.092176 (Hartree/Particle)
Thermal correction to Energy = 0.103651
Thermal correction to Enthalpy = 0.104595
Thermal correction to Gibbs Free Energy = 0.053470
Sum of electronic and zero-point Energies = -525.538579
Sum of electronic and thermal Energies = -525.527105
Sum of electronic and thermal Enthalpies = -525.526160
Sum of electronic and thermal Free Energies = -525.577285

C	-1.248822	-0.014268	0.000539
C	0.003942	-0.637867	-0.003624
C	1.244156	0.017728	-0.004074
C	1.445693	1.416013	-0.056522
N	1.675775	2.545507	-0.106274
C	2.439977	-0.737977	0.028853
N	3.422797	-1.342773	0.058736
C	-2.445166	-0.771877	-0.029102
N	-3.439795	-1.356666	-0.055565
C	-1.467452	1.383832	0.056314
N	-1.710941	2.509915	0.108588
C	0.049666	-2.140360	-0.000456
H	-0.939836	-2.587130	-0.042964
H	0.627239	-2.502022	-0.853401
H	0.545778	-2.494066	0.906402

SMD(MeCN)-B3LYP/6-311+G(d,p)

E = -525.897254
Zero-point correction = 0.090714 (Hartree/Particle)
Thermal correction to Energy = 0.102304
Thermal correction to Enthalpy = 0.103248
Thermal correction to Gibbs Free Energy = 0.051845
Sum of electronic and zero-point Energies = -525.806540
Sum of electronic and thermal Energies = -525.794950
Sum of electronic and thermal Enthalpies = -525.794006
Sum of electronic and thermal Free Energies = -525.845410

C	-1.254938	0.014575	-0.001197
C	0.003985	0.643993	0.003418
C	1.250682	-0.015679	0.004499
C	1.455247	-1.414643	0.055420
N	1.694443	-2.549284	0.104397
C	2.449716	0.736850	-0.027918
N	3.443345	1.337278	-0.057374
C	-2.454178	0.769343	0.028566
N	-3.458802	1.350842	0.055217
C	-1.476175	-1.384046	-0.055477
N	-1.728511	-2.515258	-0.106967
C	0.046874	2.151676	0.000044
H	-0.945424	2.594860	0.042697
H	0.623188	2.517748	0.853795

H	0.541639	2.509926	-0.907538

SMD(MeCN)-LC-BLYP/6-311+G(d,p)

E = -524.437937
Zero-point correction = 0.094168 (Hartree/Particle)
Thermal correction to Energy = 0.105500
Thermal correction to Enthalpy = 0.106444
Thermal correction to Gibbs Free Energy = 0.055557
Sum of electronic and zero-point Energies = -524.343770
Sum of electronic and thermal Energies = -524.332438
Sum of electronic and thermal Enthalpies = -524.331494
Sum of electronic and thermal Free Energies = -524.382381

C	-1.240244	-0.014554	-0.000135
C	0.003825	-0.631179	-0.004111
C	1.235142	0.019514	-0.003742
C	1.431942	1.414231	-0.062505
N	1.650456	2.536355	-0.118319
C	2.425243	-0.737135	0.032121
N	3.395640	-1.344392	0.064437
C	-2.431726	-0.772087	-0.032001
N	-3.414540	-1.358597	-0.060210
C	-1.454436	1.380160	0.062401
N	-1.686642	2.498857	0.121012
C	0.052809	-2.124374	-0.001405
H	-0.933094	-2.577070	-0.044318
H	0.633061	-2.482024	-0.852640
H	0.550305	-2.473917	0.904776

1,1,3,3-tetracyanopropene

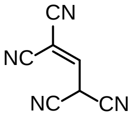

SMD(MeCN)-CAM-B3LYP/6-311+G(d,p)

E = -486.748416, υ_{min} = 8.96*i* cm^{-1} (first-order saddle point)
Zero-point correction = 0.076846 (Hartree/Particle)
Thermal correction to Energy = 0.086221
Thermal correction to Enthalpy = 0.087165
Thermal correction to Gibbs Free Energy = 0.041357
Sum of electronic and zero-point Energies = -486.671569
Sum of electronic and thermal Energies = -486.662195
Sum of electronic and thermal Enthalpies = -486.661251
Sum of electronic and thermal Free Energies = -486.707058

C	1.200812	0.198526	0.066337
H	0.984679	1.220178	0.396456
C	-0.071798	-0.581903	-0.180022
C	-1.297222	-0.076387	-0.021957
C	-1.550728	1.269788	0.400753
N	-1.757818	2.345580	0.739106
C	-2.451091	-0.888653	-0.277442
N	-3.377957	-1.532875	-0.478494
C	1.992042	-0.445071	1.122982
N	2.589751	-0.944086	1.961727
C	1.981110	0.295313	-1.174036

N	2.567671	0.382825	-2.152864
H	0.045035	-1.609967	-0.502470

SMD(MeCN)-B3LYP/6-311+G(d,p)

E = -486.989588
Zero-point correction = 0.075548 (Hartree/Particle)
Thermal correction to Energy = 0.085919
Thermal correction to Enthalpy = 0.086863
Thermal correction to Gibbs Free Energy = 0.037397
Sum of electronic and zero-point Energies = -486.914040
Sum of electronic and thermal Energies = -486.903669
Sum of electronic and thermal Enthalpies = -486.902725
Sum of electronic and thermal Free Energies = -486.952191

C	1.208134	0.205012	-0.008807
H	0.986316	1.278148	-0.057754
C	-0.069542	-0.616278	0.030273
C	-1.302737	-0.081870	0.005231
C	-1.556472	1.326988	-0.059417
N	-1.771922	2.458832	-0.109589
C	-2.458545	-0.929332	0.042995
N	-3.395618	-1.600644	0.073779
C	1.998735	-0.023733	1.211277
N	2.596583	-0.192083	2.179774
C	1.992793	-0.134008	-1.206777
N	2.584313	-0.389840	-2.159866
H	0.045986	-1.692677	0.080409

SMD(MeCN)-LC-BLYP/6-311+G(d,p)

E = -485.653596
Zero-point correction = 0.078852 (Hartree/Particle)
Thermal correction to Energy = 0.088985
Thermal correction to Enthalpy = 0.089929
Thermal correction to Gibbs Free Energy = 0.039923
Sum of electronic and zero-point Energies = -485.574744
Sum of electronic and thermal Energies = -485.564611
Sum of electronic and thermal Enthalpies = -485.563667
Sum of electronic and thermal Free Energies = -485.613673

C	1.189241	0.206265	-0.009801
H	0.976091	1.279270	-0.059917
C	-0.075603	-0.605966	0.025233
C	-1.288764	-0.078940	0.002338
C	-1.533445	1.329628	-0.060261
N	-1.728341	2.449435	-0.108337
C	-2.443120	-0.923987	0.039801
N	-3.364454	-1.590536	0.070414
C	1.970119	-0.027195	1.203021
N	2.559669	-0.201401	2.158272
C	1.973511	-0.136876	-1.194044
N	2.566348	-0.396551	-2.127612
H	0.039730	-1.683471	0.073036

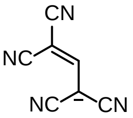

<u>SMD(MeCN)-CAM-B3LYP/6-311+G(d,p)</u>

E = -486.329242
Zero-point correction = 0.064721 (Hartree/Particle)
Thermal correction to Energy = 0.074531
Thermal correction to Enthalpy = 0.075476
Thermal correction to Gibbs Free Energy = 0.029092
Sum of electronic and zero-point Energies = -486.264521
Sum of electronic and thermal Energies = -486.254711
Sum of electronic and thermal Enthalpies = -486.253766
Sum of electronic and thermal Free Energies = -486.300151

C	0.000000	1.263396	0.217682
C	0.000000	0.000000	0.794228
C	0.000000	-1.263396	0.217682
C	-0.026933	-1.519150	-1.173982
N	-0.055274	-1.755755	-2.302084
C	0.016221	-2.402288	1.058666
N	0.031497	-3.333346	1.739748
C	-0.016221	2.402288	1.058666
N	-0.031497	3.333346	1.739748
C	0.026933	1.519150	-1.173982
N	0.055274	1.755755	-2.302084
H	0.000000	0.000000	1.878941

<u>SMD(MeCN)-B3LYP/6-311+G(d,p)</u>

E = -486.571664
Zero-point correction = 0.063560 (Hartree/Particle)
Thermal correction to Energy = 0.073463
Thermal correction to Enthalpy = 0.074408
Thermal correction to Gibbs Free Energy = 0.026826
Sum of electronic and zero-point Energies = -486.508105
Sum of electronic and thermal Energies = -486.498201
Sum of electronic and thermal Enthalpies = -486.497257
Sum of electronic and thermal Free Energies = -486.544838

C	-1.271110	0.215025	-0.000281
C	0.000000	0.792465	0.000000
C	1.271110	0.215026	0.000281
C	1.538843	-1.175275	0.016802
N	1.797829	-2.305729	0.034625
C	2.408343	1.060220	-0.009800
N	3.344284	1.746313	-0.019283
C	-2.408343	1.060219	0.009800
N	-3.344285	1.746312	0.019284
C	-1.538843	-1.175275	-0.016803
N	-1.797828	-2.305730	-0.034625
H	0.000000	1.877410	0.000000

<u>SMD(MeCN)-LC-BLYP/6-311+G(d,p)</u>

E = -485.235670
Zero-point correction = 0.066518 (Hartree/Particle)
Thermal correction to Energy = 0.076104

Thermal correction to Enthalpy = 0.077048
Thermal correction to Gibbs Free Energy = 0.031028
Sum of electronic and zero-point Energies = -485.169152
Sum of electronic and thermal Energies = -485.159566
Sum of electronic and thermal Enthalpies = -485.158622
Sum of electronic and thermal Free Energies = -485.204643

C	0.000000	1.254126	0.218613
C	0.000000	0.000000	0.791382
C	0.000000	-1.254126	0.218613
C	-0.017639	-1.502196	-1.171023
N	-0.035654	-1.725435	-2.292699
C	0.010184	-2.391093	1.055420
N	0.019755	-3.314796	1.731193
C	-0.010184	2.391093	1.055420
N	-0.019755	3.314796	1.731193
C	0.017639	1.502196	-1.171023
N	0.035654	1.725435	-2.292699
H	0.000000	0.000000	1.876673

1,1,3,3-tetracyano-2-trifuluoromethyl-1-propene

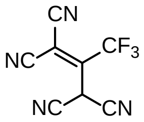

SMD(MeCN)-CAM-B3LYP/6-311+G(d,p)

E = -823.797564
Zero-point correction = 0.081153 (Hartree/Particle)
Thermal correction to Energy = 0.094981
Thermal correction to Enthalpy = 0.095925
Thermal correction to Gibbs Free Energy = 0.038942
Sum of electronic and zero-point Energies = -823.716411
Sum of electronic and thermal Energies = -823.702583
Sum of electronic and thermal Enthalpies = -823.701639
Sum of electronic and thermal Free Energies = -823.758622

C	-1.156943	0.944617	-0.058709
H	-0.888755	1.999508	-0.181286
C	0.115944	0.113304	-0.014872
C	1.329068	0.677573	-0.035018
C	1.514197	2.098188	-0.102208
N	1.674777	3.231343	-0.157718
C	2.540986	-0.090245	-0.002673
N	3.522486	-0.680517	0.017375
C	-2.006328	0.603831	-1.207496
N	-2.657533	0.366505	-2.117604
C	-1.908934	0.848590	1.199741
N	-2.479003	0.802320	2.190546
C	-0.024344	-1.403042	0.046143
F	0.682581	-1.920700	1.053552
F	0.402115	-1.972021	-1.086031
F	-1.302275	-1.751053	0.221773

SMD(MeCN)-B3LYP/6-311+G(d,p)

E = -824.111424
Zero-point correction = 0.079266 (Hartree/Particle)

Thermal correction to Energy = 0.093275
Thermal correction to Enthalpy = 0.094219
Thermal correction to Gibbs Free Energy = 0.036789
Sum of electronic and zero-point Energies = -824.032158
Sum of electronic and thermal Energies = -824.018150
Sum of electronic and thermal Enthalpies = -824.017205
Sum of electronic and thermal Free Energies = -824.074635

C	-1.162195	0.944755	-0.058310
H	-0.883557	1.998500	-0.180616
C	0.113313	0.104010	-0.013568
C	1.334998	0.675682	-0.034866
C	1.519102	2.095977	-0.100999
N	1.689413	3.234662	-0.155205
C	2.552665	-0.081631	-0.004430
N	3.550979	-0.656812	0.012998
C	-2.013955	0.614655	-1.212575
N	-2.669560	0.390776	-2.130561
C	-1.917052	0.859290	1.202934
N	-2.489751	0.824874	2.199750
C	-0.028538	-1.417697	0.047274
F	0.677447	-1.939188	1.064018
F	0.405102	-1.992591	-1.088934
F	-1.312998	-1.770804	0.218138

<u>SMD(MeCN)-LC-BLYP/6-311+G(d,p)</u>

E = -822.248825
Zero-point correction = 0.083629 (Hartree/Particle)
Thermal correction to Energy = 0.097210
Thermal correction to Enthalpy = 0.098155
Thermal correction to Gibbs Free Energy = 0.041658
Sum of electronic and zero-point Energies = -822.165196
Sum of electronic and thermal Energies = -822.151614
Sum of electronic and thermal Enthalpies = -822.150670
Sum of electronic and thermal Free Energies = -822.207167

C	-1.148028	0.945971	-0.056496
H	-0.888686	2.003481	-0.172257
C	0.117809	0.124114	-0.014726
C	1.321983	0.675498	-0.032498
C	1.512979	2.092783	-0.094543
N	1.669598	3.217530	-0.145687
C	2.522601	-0.105571	-0.002007
N	3.483122	-0.712633	0.016815
C	-1.987735	0.602911	-1.202697
N	-2.629023	0.357357	-2.107016
C	-1.899790	0.832490	1.192237
N	-2.469819	0.767369	2.172157
C	-0.024448	-1.383256	0.043707
F	0.669638	-1.898359	1.050699
F	0.408464	-1.946795	-1.078570
F	-1.298178	-1.723788	0.207928

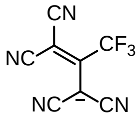

<u>SMD(MeCN)-CAM-B3LYP/6-311+G(d,p)</u>

E = -823.380575
Zero-point correction = 0.068780 (Hartree/Particle)
Thermal correction to Energy = 0.082027
Thermal correction to Enthalpy = 0.082972
Thermal correction to Gibbs Free Energy = 0.027535
Sum of electronic and zero-point Energies = -823.311795
Sum of electronic and thermal Energies = -823.298547
Sum of electronic and thermal Enthalpies = -823.297603
Sum of electronic and thermal Free Energies = -823.353040

C	1.251442	0.565974	-0.083902
C	-0.000056	-0.052499	-0.051526
C	-1.227525	0.610204	0.017202
C	-1.317832	1.978620	0.375084
N	-1.435267	3.075724	0.706793
C	-2.497112	0.003617	-0.163095
N	-3.563197	-0.413352	-0.292831
C	2.479005	-0.096614	0.172215
N	3.504750	-0.572761	0.392239
C	1.408977	1.938215	-0.398806
N	1.583700	3.037159	-0.697913
C	-0.026876	-1.578088	-0.016127
F	-0.091603	-2.019299	1.252751
F	-1.081845	-2.085107	-0.665107
F	1.056776	-2.129367	-0.572564

<u>SMD(MeCN)-B3LYP/6-311+G(d,p)</u>

E = -823.695873
Zero-point correction = 0.067168 (Hartree/Particle)
Thermal correction to Energy = 0.080582
Thermal correction to Enthalpy = 0.081526
Thermal correction to Gibbs Free Energy = 0.025757
Sum of electronic and zero-point Energies = -823.628705
Sum of electronic and thermal Energies = -823.615291
Sum of electronic and thermal Enthalpies = -823.614347
Sum of electronic and thermal Free Energies = -823.670116

C	-1.259259	-0.564520	-0.086357
C	0.000581	0.057721	-0.052397
C	1.232202	-0.615597	0.022019
C	1.317982	-1.984681	0.381364
N	1.439351	-3.087711	0.715868
C	2.506393	-0.020405	-0.163641
N	3.583571	0.386575	-0.296143
C	-2.487689	0.093220	0.177698
N	-3.522432	0.564146	0.403671
C	-1.420635	-1.935931	-0.405880
N	-1.606949	-3.038549	-0.711026
C	0.031566	1.587297	-0.017940
F	0.101767	2.037047	1.257673

F	1.091382	2.096615	-0.674085
F	-1.057775	2.147022	-0.574231

SMD(MeCN)-LC-BLYP/6-311+G(d,p)

E = -821.833074
Zero-point correction = 0.070871 (Hartree/Particle)
Thermal correction to Energy = 0.083956
Thermal correction to Enthalpy = 0.084900
Thermal correction to Gibbs Free Energy = 0.029112
Sum of electronic and zero-point Energies = -821.762203
Sum of electronic and thermal Energies = -821.749118
Sum of electronic and thermal Enthalpies = -821.748174
Sum of electronic and thermal Free Energies = -821.803961

C	1.241451	0.566821	-0.079286
C	0.000506	-0.046809	-0.051760
C	-1.221420	0.601865	0.009865
C	-1.319354	1.967837	0.361408
N	-1.438013	3.057294	0.685859
C	-2.483547	-0.016181	-0.159209
N	-3.536601	-0.443243	-0.280337
C	2.466018	-0.098081	0.167205
N	3.481736	-0.576774	0.379951
C	1.395144	1.937778	-0.385762
N	1.560520	3.030898	-0.675909
C	-0.020633	-1.564604	-0.016001
F	-0.077607	-1.996777	1.246348
F	-1.070620	-2.070969	-0.655658
F	1.056840	-2.106585	-0.573549

1,1,2,3,3-pentacyanopropene

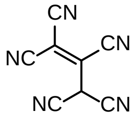

SMD(MeCN)-CAM-B3LYP/6-311+G(d,p)

E = -578.953136
Zero-point correction = 0.075409 (Hartree/Particle)
Thermal correction to Energy = 0.087425
Thermal correction to Enthalpy = 0.088369
Thermal correction to Gibbs Free Energy = 0.036068
Sum of electronic and zero-point Energies = -578.877727
Sum of electronic and thermal Energies = -578.865711
Sum of electronic and thermal Enthalpies = -578.864767
Sum of electronic and thermal Free Energies = -578.917068

C	1.170805	-0.563238	-0.146525
H	0.903489	-1.565891	-0.498333
C	-0.096232	0.280190	-0.023444
C	-1.328586	-0.253362	-0.093070
C	0.062471	1.682700	0.193262
N	0.215378	2.806250	0.364171
C	-1.551818	-1.651570	-0.301528
N	-1.746729	-2.768607	-0.467907
C	-2.501296	0.556478	0.046028
N	-3.445967	1.195687	0.156148

C	2.101650	-0.000642	-1.131082
N	2.815354	0.429867	-1.914566
C	1.820705	-0.723695	1.161171
N	2.309151	-0.862521	2.186364

SMD(MeCN)-B3LYP/6-311+G(d,p)

E = -579.235766
Zero-point correction = 0.073770 (Hartree/Particle)
Thermal correction to Energy = 0.085922
Thermal correction to Enthalpy = 0.086866
Thermal correction to Gibbs Free Energy = 0.034194
Sum of electronic and zero-point Energies = -579.161996
Sum of electronic and thermal Energies = -579.149845
Sum of electronic and thermal Enthalpies = -579.148900
Sum of electronic and thermal Free Energies = -579.201573

C	1.177074	-0.563108	-0.144740
H	0.900342	-1.565406	-0.494440
C	-0.093641	0.290734	-0.020577
C	-1.336921	-0.248772	-0.091932
C	0.068881	1.689390	0.194646
N	0.232082	2.818807	0.365717
C	-1.563760	-1.645050	-0.299634
N	-1.772564	-2.766792	-0.465653
C	-2.510540	0.558255	0.045173
N	-3.466485	1.193381	0.153280
C	2.110444	-0.009432	-1.136201
N	2.829298	0.411653	-1.928928
C	1.829887	-0.727480	1.164373
N	2.322113	-0.871594	2.193841

SMD(MeCN)-LC-BLYP/6-311+G(d,p)

E = -577.661023
Zero-point correction = 0.077663 (Hartree/Particle)
Thermal correction to Energy = 0.089468
Thermal correction to Enthalpy = 0.090412
Thermal correction to Gibbs Free Energy = 0.038606
Sum of electronic and zero-point Energies = -577.583360
Sum of electronic and thermal Energies = -577.571555
Sum of electronic and thermal Enthalpies = -577.570611
Sum of electronic and thermal Free Energies = -577.622417

C	1.161670	-0.565304	-0.147167
H	0.902737	-1.570193	-0.498207
C	-0.097691	0.267745	-0.025315
C	-1.318406	-0.255851	-0.092503
C	0.061524	1.670626	0.190566
N	0.209374	2.785557	0.359710
C	-1.542565	-1.652238	-0.299582
N	-1.728431	-2.761708	-0.463958
C	-2.485273	0.558879	0.046941
N	-3.415188	1.203219	0.157948
C	2.085599	0.001740	-1.125643
N	2.790821	0.439070	-1.900652
C	1.808595	-0.718184	1.155083

N 2.294359 -0.848177 2.173228

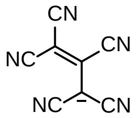

SMD(MeCN)-CAM-B3LYP/6-311+G(d,p)

E = -578.540545
Zero-point correction = 0.062887 (Hartree/Particle)
Thermal correction to Energy = 0.074529
Thermal correction to Enthalpy = 0.075473
Thermal correction to Gibbs Free Energy = 0.023932
Sum of electronic and zero-point Energies = -578.477658
Sum of electronic and thermal Energies = -578.466017
Sum of electronic and thermal Enthalpies = -578.465072
Sum of electronic and thermal Free Energies = -578.516613

C	-1.264595	-0.184686	-0.002396
C	0.000002	0.410764	0.000003
C	1.264597	-0.184690	0.002400
C	0.000004	1.851337	0.000005
N	0.000004	2.997972	0.000016
C	1.480616	-1.582310	0.043524
N	1.709424	-2.710496	0.082811
C	2.437915	0.607483	-0.022792
N	3.403624	1.235894	-0.045017
C	-2.437912	0.607489	0.022794
N	-3.403619	1.235906	0.044981
C	-1.480620	-1.582305	-0.043522
N	-1.709439	-2.710489	-0.082806

SMD(MeCN)-B3LYP/6-311+G(d,p)

E = -578.824215
Zero-point correction = 0.061579 (Hartree/Particle)
Thermal correction to Energy = 0.073322
Thermal correction to Enthalpy = 0.074267
Thermal correction to Gibbs Free Energy = 0.022546
Sum of electronic and zero-point Energies = -578.762636
Sum of electronic and thermal Energies = -578.750893
Sum of electronic and thermal Enthalpies = -578.749949
Sum of electronic and thermal Free Energies = -578.801669

C	1.272330	0.183651	-0.002489
C	0.000008	-0.419341	-0.000103
C	-1.272323	0.183684	0.002304
C	-0.000019	-1.857154	-0.000023
N	-0.000074	-3.010763	0.000002
C	-1.490227	1.581253	0.040688
N	-1.728655	2.714629	0.077667
C	-2.448464	-0.604677	-0.021302
N	-3.425767	-1.228087	-0.041954
C	2.448504	-0.604713	0.021269
N	3.425830	-1.228068	0.042179
C	1.490252	1.581236	-0.040723
N	1.728614	2.714627	-0.077569

SMD(MeCN)-LC-BLYP/6-311+G(d,p)

E = -577.250122
Zero-point correction = 0.064797 (Hartree/Particle)
Thermal correction to Energy = 0.076262
Thermal correction to Enthalpy = 0.077206
Thermal correction to Gibbs Free Energy = 0.026097
Sum of electronic and zero-point Energies = -577.185325
Sum of electronic and thermal Energies = -577.173860
Sum of electronic and thermal Enthalpies = -577.172915
Sum of electronic and thermal Free Energies = -577.224025

C	-1.254719	-0.185273	-0.002706
C	0.000000	0.400946	-0.000001
C	1.254719	-0.185273	0.002705
C	0.000000	1.840317	-0.000002
N	0.000000	2.977746	-0.000002
C	1.469340	-1.579927	0.047486
N	1.687797	-2.700624	0.089544
C	2.421927	0.609496	-0.024523
N	3.373958	1.241813	-0.048554
C	-2.421927	0.609496	0.024524
N	-3.373958	1.241813	0.048555
C	-1.469339	-1.579927	-0.047485
N	-1.687797	-2.700625	-0.089541

1,1-bis(phenylsulfonyl)ethylene

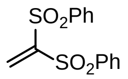

SMD(DCE)-CAM-B3LYP/6-311+G(d,p)

E = -1637.714023
Zero-point correction = 0.236542 (Hartree/Particle)
Thermal correction to Energy = 0.254205
Thermal correction to Enthalpy = 0.255149
Thermal correction to Gibbs Free Energy = 0.188855
Sum of electronic and zero-point Energies = -1637.477482
Sum of electronic and thermal Energies = -1637.459818
Sum of electronic and thermal Enthalpies = -1637.458874
Sum of electronic and thermal Free Energies = -1637.525168

C	0.000000	0.000000	2.636667
H	0.784401	-0.503230	3.190080
H	-0.784401	0.503230	3.190080
C	0.000000	0.000000	1.315296
S	-1.318242	0.823085	0.407441
O	-1.652342	-0.016576	-0.737774
O	-2.342107	1.138095	1.399453
S	1.318242	-0.823085	0.407441
O	1.652342	0.016576	-0.737774
O	2.342107	-1.138095	1.399453
C	-0.627012	2.353862	-0.181447
C	-0.243813	2.454652	-1.510471
C	-0.515589	3.419535	0.702998

C	0.262412	3.663848	-1.965108
H	-0.343152	1.606368	-2.173447
C	0.000000	4.618344	0.236111
H	-0.831831	3.319277	1.733635
C	0.386411	4.738789	-1.094284
H	0.561476	3.763363	-3.001187
H	0.093650	5.460542	0.910394
H	0.784954	5.679700	-1.454637
C	0.627012	-2.353862	-0.181447
C	0.243813	-2.454652	-1.510471
C	0.515589	-3.419535	0.702998
C	-0.262412	-3.663848	-1.965108
H	0.343152	-1.606368	-2.173447
C	0.000000	-4.618344	0.236111
H	0.831831	-3.319277	1.733635
C	-0.386411	-4.738789	-1.094284
H	-0.561476	-3.763363	-3.001187
H	-0.093650	-5.460542	0.910394
H	-0.784954	-5.679700	-1.454637

TS-AD_tBu_1a

SMD(DCE)-CAM-B3LYP/6-311+G(d,p)

E = -2553.236330
Zero-point correction = 0.638689 (Hartree/Particle)
Thermal correction to Energy = 0.679834
Thermal correction to Enthalpy = 0.680778
Thermal correction to Gibbs Free Energy = 0.562339
Sum of electronic and zero-point Energies = -2552.597754
Sum of electronic and thermal Energies = -2552.556609
Sum of electronic and thermal Enthalpies = -2552.555665
Sum of electronic and thermal Free Energies = -2552.674104

C	-0.524392	-0.793879	0.143550
H	0.123113	-0.532651	0.970763
H	-0.490405	-1.822921	-0.183953
C	-1.746307	-0.154567	0.052789
S	-3.039332	-0.866959	-0.923600
O	-3.769523	0.192822	-1.620108
O	-2.409595	-1.929629	-1.711592
S	-2.077236	1.314757	0.975675
O	-3.481184	1.280378	1.386657
O	-1.032151	1.405509	1.996815
C	-4.177267	-1.656707	0.205954
C	-5.401074	-1.059603	0.465068
C	-3.816780	-2.868448	0.782033
C	-6.287780	-1.696991	1.321785
H	-5.650797	-0.114201	0.003884
C	-4.708317	-3.491133	1.642215
H	-2.860900	-3.324492	0.556873
C	-5.940943	-2.906120	1.910836
H	-7.249386	-1.244298	1.530693
H	-4.442304	-4.436568	2.098901
H	-6.635350	-3.397524	2.582063

C	-1.869160	2.724112	-0.104386
C	-2.926804	3.133534	-0.906080
C	-0.653429	3.394389	-0.099722
C	-2.752025	4.237351	-1.727617
H	-3.863235	2.593873	-0.891409
C	-0.492498	4.497543	-0.926123
H	0.144538	3.066574	0.552934
C	-1.538283	4.915037	-1.740020
H	-3.567913	4.569842	-2.357781
H	0.448323	5.034430	-0.928405
H	-1.409426	5.777411	-2.383387
C	0.878839	-0.136479	-1.276769
C	1.641149	-1.326483	-1.462358
C	0.073085	0.373220	-2.432552
H	1.299068	0.623609	-0.628933
H	1.240632	-2.032818	-2.177809
C	2.756355	-1.707204	-0.778875
H	-0.444516	-0.437430	-2.948031
H	-0.660134	1.119915	-2.128178
H	0.746665	0.850272	-3.153963
O	3.341329	-0.896734	0.123121
Si	4.348545	0.470444	0.193102
C	3.999899	1.590039	-1.261466
C	6.142663	-0.050607	0.125138
C	3.954146	1.268663	1.866856
H	4.765801	2.370201	-1.308972
H	4.049799	1.033052	-2.201649
H	3.026992	2.081533	-1.211413
H	6.775591	0.813320	0.350036
H	6.382813	-0.832962	0.848554
H	6.424044	-0.404845	-0.868849
C	4.891304	2.468237	2.082729
H	4.649736	2.962350	3.030684
H	5.941222	2.168704	2.130267
H	4.791089	3.217924	1.291818
C	4.167146	0.253765	2.999123
H	3.498406	-0.605292	2.903581
H	5.194219	-0.120872	3.028057
H	3.963351	0.724548	3.967900
C	2.503706	1.763988	1.902466
H	2.281778	2.210552	2.878609
H	2.320870	2.532371	1.146596
H	1.786862	0.955247	1.747689
C	3.380827	-3.092513	-0.889442
C	4.827239	-2.980002	-1.393391
C	3.382987	-3.727337	0.512582
C	2.605777	-4.008609	-1.840267
H	4.873194	-2.471221	-2.360016
H	5.458437	-2.442500	-0.688527
H	5.248246	-3.980563	-1.519719
H	2.365522	-3.840217	0.896304
H	3.838211	-4.719762	0.464488
H	3.951071	-3.124260	1.221440

H	3.084158	-4.990457	-1.853605
H	1.570325	-4.148982	-1.522629
H	2.604360	-3.629824	-2.865014

TS-AD_tBu_1b

SMD(DCE)-CAM-B3LYP/6-311+G(d,p)

E = -2553.235106
Zero-point correction = 0.638702 (Hartree/Particle)
Thermal correction to Energy = 0.679994
Thermal correction to Enthalpy = 0.680938
Thermal correction to Gibbs Free Energy = 0.560535
Sum of electronic and zero-point Energies = -2552.596469
Sum of electronic and thermal Energies = -2552.555177
Sum of electronic and thermal Enthalpies = -2552.554233
Sum of electronic and thermal Free Energies = -2552.674636

C	-0.383747	-0.223752	0.742838
H	0.375491	0.539521	0.637411
H	-0.278915	-0.886182	1.590758
C	-1.668424	0.061249	0.329083
S	-3.048270	-0.764988	1.069117
O	-2.511346	-1.513591	2.208396
O	-4.087122	0.240128	1.301403
S	-1.951190	1.386617	-0.804470
O	-0.670279	1.609999	-1.479468
O	-3.136436	1.089611	-1.609475
C	-3.726443	-1.977880	-0.058385
C	-3.388591	-3.312851	0.118999
C	-4.615919	-1.575602	-1.046447
C	-3.946545	-4.264316	-0.723251
H	-2.711570	-3.601930	0.911655
C	-5.164172	-2.536760	-1.882700
H	-4.868119	-0.531072	-1.161171
C	-4.828628	-3.876379	-1.723747
H	-3.694615	-5.309756	-0.592705
H	-5.858767	-2.237221	-2.658063
H	-5.262572	-4.622190	-2.379315
C	-2.300305	2.856027	0.151711
C	-1.237278	3.626661	0.605050
C	-3.618592	3.206705	0.406134
C	-1.506854	4.776349	1.333007
H	-0.217795	3.343245	0.377491
C	-3.873948	4.361237	1.131978
H	-4.425869	2.585624	0.044250
C	-2.821897	5.141638	1.595861
H	-0.688484	5.390474	1.688649
H	-4.897896	4.650591	1.334805
H	-3.027884	6.042126	2.162444
C	0.710677	-1.407126	-0.647042
C	1.595775	-2.100447	0.220566
C	-0.307519	-2.187575	-1.414891
H	1.087431	-0.528353	-1.151590
H	1.233880	-3.036931	0.624419
C	2.817232	-1.672534	0.655831

H	-0.742589	-2.987628	-0.814326
H	-1.111003	-1.555110	-1.794342
H	0.177009	-2.651597	-2.282243
O	3.357473	-0.522144	0.221209
Si	4.106712	0.220283	-1.108880
C	3.229230	-0.216565	-2.699990
C	5.876699	-0.362202	-1.243007
C	4.022033	2.070447	-0.705456
H	3.865935	0.064288	-3.544732
H	3.055799	-1.293983	-2.772876
H	2.271047	0.291076	-2.825375
H	6.384875	0.212978	-2.023787
H	6.439246	-0.231427	-0.316725
H	5.930865	-1.414762	-1.532051
C	4.416229	2.869396	-1.958480
H	4.411054	3.942060	-1.733441
H	5.421031	2.617847	-2.311154
H	3.719502	2.706713	-2.785134
C	4.995246	2.412607	0.431772
H	4.771106	1.852390	1.343973
H	6.033665	2.207505	0.158084
H	4.925562	3.479084	0.676134
C	2.600815	2.465696	-0.283736
H	2.555773	3.545584	-0.098810
H	1.857241	2.233582	-1.050175
H	2.304171	1.959861	0.638353
C	3.633993	-2.392257	1.721415
C	4.952806	-2.893434	1.112206
C	3.944757	-1.394494	2.850247
C	2.888475	-3.588975	2.317986
H	4.769679	-3.575262	0.277499
H	5.570951	-2.070744	0.756962
H	5.523011	-3.434970	1.871388
H	3.026728	-1.027574	3.317126
H	4.541856	-1.887883	3.621160
H	4.505743	-0.536267	2.479851
H	3.508738	-4.041360	3.094978
H	1.942587	-3.294025	2.777548
H	2.684524	-4.359559	1.571207

TS-AD_tBu_1c

SMD(DCE)-CAM-B3LYP/6-311+G(d,p)

E = -2553.233965
Zero-point correction = 0.638493 (Hartree/Particle)
Thermal correction to Energy = 0.679850
Thermal correction to Enthalpy = 0.680794
Thermal correction to Gibbs Free Energy = 0.559989
Sum of electronic and zero-point Energies = -2552.595441
Sum of electronic and thermal Energies = -2552.554084
Sum of electronic and thermal Enthalpies = -2552.553140
Sum of electronic and thermal Free Energies = -2552.673945

C	-0.359568	-0.690999	0.132543
H	0.199550	0.188358	0.428485

H	-0.277859	-1.546025	0.788317
C	-1.555910	-0.527016	-0.534124
S	-2.681013	-1.896537	-0.646339
O	-2.977396	-2.224037	-2.038638
O	-2.097750	-2.942008	0.201021
S	-1.934820	1.006742	-1.321431
O	-0.711457	1.490900	-1.963968
O	-3.149739	0.832414	-2.115325
C	-4.201882	-1.368852	0.125108
C	-5.302463	-1.083031	-0.668464
C	-4.255987	-1.289617	1.511390
C	-6.488564	-0.705174	-0.053411
H	-5.225955	-1.155181	-1.744707
C	-5.445310	-0.907903	2.112690
H	-3.386596	-1.528560	2.111232
C	-6.558374	-0.616655	1.331161
H	-7.358378	-0.480873	-0.658768
H	-5.504846	-0.843307	3.192274
H	-7.486696	-0.321684	1.806149
C	-2.317103	2.154888	-0.003688
C	-1.299808	2.931881	0.533191
C	-3.626812	2.254366	0.447178
C	-1.603263	3.822876	1.552899
H	-0.292650	2.853913	0.146361
C	-3.917071	3.150504	1.465804
H	-4.407277	1.651397	0.001703
C	-2.907829	3.929427	2.019331
H	-0.820066	4.439352	1.977152
H	-4.935370	3.243258	1.823248
H	-3.140868	4.628691	2.813601
C	1.213977	-1.292603	-1.168201
C	2.006410	-2.083712	-0.291118
C	0.535315	-1.960001	-2.322471
H	1.529754	-0.273304	-1.345692
H	1.704043	-3.117044	-0.181066
C	3.051756	-1.661132	0.475798
H	0.099037	-2.919558	-2.038035
H	-0.241865	-1.333663	-2.759591
H	1.277734	-2.155156	-3.105264
O	3.524275	-0.403591	0.396589
Si	4.422330	0.615601	-0.623765
C	3.937344	0.377575	-2.412598
C	6.244773	0.236700	-0.463095
C	4.024933	2.353690	0.018258
H	4.693511	0.849721	-3.047545
H	3.912422	-0.683203	-2.677320
H	2.970705	0.815492	-2.667660
H	6.812553	0.976728	-1.036189
H	6.599129	0.272238	0.568728
H	6.486144	-0.746236	-0.875230
C	4.648840	3.390512	-0.929585
H	4.441527	4.402418	-0.562973
H	5.735602	3.286290	-0.997859

H	4.239771	3.320108	-1.941311
C	4.603813	2.548823	1.426799
H	4.196796	1.825247	2.138470
H	5.693087	2.456598	1.438448
H	4.356073	3.550450	1.797401
C	2.507129	2.569920	0.067640
H	2.287005	3.586404	0.414696
H	2.041889	2.452649	-0.914868
H	2.026150	1.872227	0.757605
C	3.712777	-2.514957	1.550026
C	5.206171	-2.691396	1.238617
C	3.560688	-1.784346	2.896119
C	3.073323	-3.900544	1.670321
H	5.354575	-3.167632	0.265789
H	5.733381	-1.739095	1.242090
H	5.663936	-3.329355	1.998837
H	2.506865	-1.660916	3.159921
H	4.041316	-2.366965	3.686075
H	4.025017	-0.798128	2.867252
H	3.571443	-4.452422	2.470647
H	2.011466	-3.841854	1.918686
H	3.181374	-4.481843	0.751797

TS-AD_tBu_1d

SMD(DCE)-CAM-B3LYP/6-311+G(d,p)

E = -2553.233375
Zero-point correction = 0.638778 (Hartree/Particle)
Thermal correction to Energy = 0.679986
Thermal correction to Enthalpy = 0.680930
Thermal correction to Gibbs Free Energy = 0.561552
Sum of electronic and zero-point Energies = -2552.594719
Sum of electronic and thermal Energies = -2552.553511
Sum of electronic and thermal Enthalpies = -2552.552567
Sum of electronic and thermal Free Energies = -2552.671946

C	0.250216	0.459558	-0.203877
H	-0.290632	-0.251347	0.405752
H	0.136591	1.500439	0.069307
C	1.456783	0.068933	-0.746614
S	2.623863	1.290516	-1.262666
O	3.714226	0.619344	-1.967511
O	1.893607	2.374007	-1.922216
S	1.796888	-1.657255	-0.964711
O	0.689700	-2.349603	-0.297573
O	2.046707	-1.959586	-2.373104
C	3.296469	1.973767	0.248764
C	4.422134	1.388121	0.812511
C	2.685297	3.080840	0.822876
C	4.939316	1.922967	1.983842
H	4.892732	0.536926	0.337685
C	3.210921	3.603742	1.995719
H	1.825229	3.537337	0.350543
C	4.333404	3.024697	2.575452
H	5.820831	1.480049	2.431108

H	2.747062	4.469928	2.451695
H	4.742540	3.439045	3.489267
C	3.287938	-2.018899	-0.053610
C	3.216104	-2.119686	1.330375
C	4.473909	-2.223050	-0.742892
C	4.367615	-2.428361	2.038110
H	2.275847	-1.967460	1.845441
C	5.618832	-2.534456	-0.022219
H	4.494772	-2.136652	-1.820446
C	5.565483	-2.634844	1.362457
H	4.328177	-2.513438	3.117194
H	6.552796	-2.699591	-0.545462
H	6.462358	-2.878651	1.919772
C	-1.327391	0.429760	-1.632223
C	-2.133257	1.512579	-1.184993
C	-0.664503	0.529232	-2.970421
H	-1.631057	-0.566775	-1.340786
H	-1.876109	2.489290	-1.573468
C	-3.143317	1.457555	-0.269339
H	-0.212814	1.510032	-3.123601
H	0.104592	-0.232288	-3.104943
H	-1.418546	0.375256	-3.751417
O	-3.542740	0.294803	0.274754
Si	-4.389082	-1.137868	-0.065643
C	-3.967470	-1.779743	-1.769520
C	-6.229119	-0.817077	-0.011570
C	-3.857479	-2.320079	1.316888
H	-4.706388	-2.535981	-2.052290
H	-4.021034	-0.982791	-2.516598
H	-2.980832	-2.241891	-1.834021
H	-6.758439	-1.770101	-0.112253
H	-6.554643	-0.353394	0.921372
H	-6.548039	-0.180769	-0.840948
C	-4.369244	-3.731198	0.984533
H	-4.098170	-4.423975	1.789457
H	-5.458042	-3.763544	0.881583
H	-3.932054	-4.118253	0.060085
C	-4.453231	-1.875524	2.660377
H	-4.133389	-0.866335	2.934972
H	-5.546328	-1.891051	2.649927
H	-4.123494	-2.552590	3.457126
C	-2.328690	-2.356044	1.434771
H	-2.030551	-3.079712	2.202818
H	-1.841508	-2.653863	0.503447
H	-1.926747	-1.382600	1.726105
C	-3.838247	2.688569	0.297767
C	-5.342562	2.627183	-0.006023
C	-3.630808	2.693647	1.822656
C	-3.278603	3.992218	-0.277274
H	-5.527645	2.574782	-1.082222
H	-5.814128	1.768646	0.468728
H	-5.828575	3.528581	0.375750
H	-2.569027	2.758435	2.075395

H	-4.136187	3.558877	2.259119
H	-4.037707	1.792133	2.281683
H	-3.799341	4.834956	0.182955
H	-2.212581	4.107553	-0.069667
H	-3.429397	4.060618	-1.357072

TS-AD_tBu_2a

SMD(DCE)-CAM-B3LYP/6-311+G(d,p)

E = -2553.233131
Zero-point correction = 0.638673 (Hartree/Particle)
Thermal correction to Energy = 0.679926
Thermal correction to Enthalpy = 0.680871
Thermal correction to Gibbs Free Energy = 0.561481
Sum of electronic and zero-point Energies = -2552.594458
Sum of electronic and thermal Energies = -2552.553204
Sum of electronic and thermal Enthalpies = -2552.552260
Sum of electronic and thermal Free Energies = -2552.671650

C	-0.281165	-0.053656	-0.904917
H	-0.138671	-0.535771	-1.863917
H	0.418699	0.724196	-0.635429
C	-1.573279	0.048403	-0.439198
S	-1.950127	1.201747	0.851153
O	-2.921894	0.613276	1.775863
O	-0.657673	1.641917	1.381206
S	-2.904796	-0.760921	-1.279190
O	-4.054475	0.144568	-1.232630
O	-2.383721	-1.184726	-2.580966
C	-2.721801	2.626206	0.093841
C	-4.046357	2.914269	0.380419
C	-1.959115	3.435905	-0.737574
C	-4.619398	4.048623	-0.178110
H	-4.616016	2.260134	1.026397
C	-2.545248	4.561382	-1.296058
H	-0.923647	3.196650	-0.945946
C	-3.872552	4.867122	-1.015492
H	-5.653204	4.288627	0.038701
H	-1.963820	5.202002	-1.947863
H	-4.326050	5.748687	-1.452972
C	-3.378793	-2.255709	-0.411696
C	-4.029569	-2.165002	0.812449
C	-3.141883	-3.479071	-1.022292
C	-4.435083	-3.332895	1.440920
H	-4.202925	-1.200624	1.269918
C	-3.559612	-4.639553	-0.385234
H	-2.645787	-3.519252	-1.982558
C	-4.200241	-4.566862	0.844824
H	-4.940211	-3.277235	2.397580
H	-3.384879	-5.600386	-0.854020
H	-4.523049	-5.475098	1.340061
C	0.994019	-1.526867	0.004491
C	1.388609	-0.964365	1.242887
C	0.150215	-2.762294	-0.006501
H	1.682996	-1.425280	-0.823758

H	0.740759	-1.148747	2.088912
C	2.468215	-0.155112	1.450397
H	-0.665781	-2.698230	0.715765
H	-0.264719	-2.963728	-0.994057
H	0.766293	-3.626187	0.271184
O	3.322711	0.139616	0.452545
Si	4.573766	-0.546083	-0.466516
C	4.333838	-2.394509	-0.607456
C	6.210629	-0.217807	0.373425
C	4.493392	0.343225	-2.140224
H	5.266568	-2.852218	-0.951101
H	4.099556	-2.831916	0.367449
H	3.545409	-2.682170	-1.305066
H	7.027783	-0.460788	-0.313051
H	6.326144	0.826356	0.671846
H	6.335969	-0.843065	1.260671
C	5.497619	-0.317187	-3.098996
H	5.482534	0.199130	-4.065662
H	6.523170	-0.269696	-2.721989
H	5.256382	-1.367396	-3.286500
C	4.863336	1.823856	-1.969254
H	4.181015	2.338441	-1.287091
H	5.879933	1.950420	-1.587630
H	4.810080	2.336708	-2.936712
C	3.088874	0.248974	-2.749482
H	3.073991	0.740163	-3.729443
H	2.773849	-0.787444	-2.901763
H	2.343486	0.742762	-2.122383
C	2.756353	0.560628	2.761441
C	4.125253	0.122345	3.303650
C	2.780124	2.074527	2.485128
C	1.691634	0.274832	3.823405
H	4.162613	-0.959292	3.460418
H	4.934001	0.402895	2.630714
H	4.308920	0.607990	4.265436
H	1.810804	2.416274	2.115134
H	3.001857	2.611810	3.410908
H	3.543691	2.331767	1.750056
H	1.935473	0.834463	4.729484
H	0.700350	0.587613	3.491053
H	1.654828	-0.784262	4.090122

TS-AD_tBu_2b

<u>SMD(DCE)-CAM-B3LYP/6-311+G(d,p)</u>

E = -2553.234706
Zero-point correction = 0.638815 (Hartree/Particle)
Thermal correction to Energy = 0.680168
Thermal correction to Enthalpy = 0.681112
Thermal correction to Gibbs Free Energy = 0.561151
Sum of electronic and zero-point Energies = -2552.595891
Sum of electronic and thermal Energies = -2552.554538
Sum of electronic and thermal Enthalpies = -2552.553594
Sum of electronic and thermal Free Energies = -2552.673555

C	-0.301024	-0.457803	-0.131401
H	0.032954	-1.399616	0.283038
H	0.230328	-0.096470	-0.999969
C	-1.646991	-0.172890	-0.039641
S	-2.377819	1.046869	-1.087163
O	-1.416904	1.302914	-2.162550
O	-3.728415	0.597175	-1.426544
S	-2.679477	-1.119518	1.040085
O	-1.767880	-1.868874	1.909630
O	-3.685486	-0.250991	1.652469
C	-2.533872	2.571607	-0.165216
C	-1.583089	3.565880	-0.352910
C	-3.611389	2.744524	0.694424
C	-1.713222	4.759007	0.344412
H	-0.766053	3.412465	-1.044708
C	-3.727466	3.940654	1.386571
H	-4.338182	1.954911	0.824072
C	-2.780066	4.943658	1.214563
H	-0.982159	5.545760	0.202652
H	-4.562364	4.089892	2.060418
H	-2.878378	5.876539	1.757108
C	-3.539875	-2.317569	0.030650
C	-2.860922	-3.458620	-0.377827
C	-4.866982	-2.097291	-0.304992
C	-3.532717	-4.397677	-1.145744
H	-1.828702	-3.617607	-0.092828
C	-5.530001	-3.048357	-1.067872
H	-5.367371	-1.197402	0.024285
C	-4.864218	-4.192318	-1.489480
H	-3.017164	-5.293306	-1.470327
H	-6.568275	-2.892347	-1.334330
H	-5.385865	-4.930318	-2.087424
C	0.922126	0.597072	1.249853
C	1.623189	1.530372	0.440320
C	0.001025	1.100586	2.317625
H	1.453895	-0.313515	1.493199
H	1.216453	2.530285	0.373556
C	2.738741	1.248659	-0.294347
H	-0.648671	1.898338	1.953902
H	-0.615027	0.301316	2.728470
H	0.597830	1.511192	3.140629
O	3.228371	-0.003608	-0.306692
Si	4.432120	-0.988702	0.372815
C	4.763483	-0.369995	2.102240
C	5.983498	-0.906301	-0.667475
C	3.742209	-2.760160	0.332631
H	5.474267	-1.026371	2.613287
H	5.199891	0.632468	2.080843
H	3.853712	-0.328074	2.705401
H	6.768443	-1.505904	-0.197095
H	5.822567	-1.303344	-1.673011
H	6.368429	0.110047	-0.764210
C	4.908139	-3.742937	0.540162

H	4.523434	-4.768584	0.571475
H	5.638964	-3.692499	-0.270175
H	5.436956	-3.567088	1.482263
C	3.091180	-3.043228	-1.028796
H	2.236059	-2.388845	-1.213488
H	3.795127	-2.912960	-1.855895
H	2.733745	-4.079037	-1.064320
C	2.711837	-2.998794	1.444735
H	2.363734	-4.037698	1.410170
H	3.133690	-2.829414	2.439052
H	1.830794	-2.362434	1.340194
C	3.458641	2.268712	-1.167607
C	4.737646	2.734824	-0.448932
C	3.820634	1.623709	-2.513856
C	2.590813	3.501534	-1.442428
H	4.495397	3.231929	0.493476
H	5.417115	1.910366	-0.234637
H	5.274015	3.447721	-1.080745
H	2.923717	1.284086	-3.038532
H	4.323287	2.359512	-3.146132
H	4.486223	0.770342	-2.392245
H	3.135062	4.176732	-2.106780
H	1.653310	3.232184	-1.934014
H	2.360396	4.056885	-0.531256

TS-AD_tBu_2c

SMD(DCE)-CAM-B3LYP/6-311+G(d,p)

E = -2553.233127
Zero-point correction = 0.638682 (Hartree/Particle)
Thermal correction to Energy = 0.679970
Thermal correction to Enthalpy = 0.680915
Thermal correction to Gibbs Free Energy = 0.561170
Sum of electronic and zero-point Energies = -2552.594445
Sum of electronic and thermal Energies = -2552.553157
Sum of electronic and thermal Enthalpies = -2552.552212
Sum of electronic and thermal Free Energies = -2552.671957

C	0.051153	0.287696	0.128668
H	-0.213456	1.333728	0.207302
H	-0.396331	-0.266304	-0.683889
C	1.318166	-0.075027	0.535854
S	1.873660	-1.729997	0.220969
O	1.922759	-2.492732	1.468496
O	1.016432	-2.232333	-0.857272
S	2.312200	1.051620	1.459213
O	1.410187	2.077059	1.990848
O	3.175246	0.313005	2.379464
C	3.537220	-1.623180	-0.413765
C	4.597724	-1.989075	0.401740
C	3.727074	-1.230690	-1.733491
C	5.882949	-1.960790	-0.122439
H	4.415546	-2.294186	1.423204
C	5.015796	-1.203083	-2.242711
H	2.882688	-0.961253	-2.355474

C	6.090021	-1.569096	-1.438639
H	6.721917	-2.247683	0.499752
H	5.180884	-0.902081	-3.269963
H	7.095259	-1.550206	-1.842947
C	3.376700	1.863349	0.270091
C	2.806436	2.598859	-0.762424
C	4.749823	1.786871	0.438939
C	3.638075	3.268283	-1.645343
H	1.730805	2.650209	-0.878712
C	5.572742	2.468565	-0.449413
H	5.165080	1.206741	1.252122
C	5.019330	3.203977	-1.487835
H	3.208097	3.844035	-2.455876
H	6.647900	2.420497	-0.326942
H	5.665071	3.731629	-2.179795
C	-1.475295	-0.261982	1.499960
C	-2.054575	-1.428562	0.927669
C	-0.781616	-0.377060	2.822658
H	-1.992471	0.672509	1.327155
H	-1.612226	-2.375149	1.207443
C	-3.067607	-1.460951	0.015795
H	-0.102221	-1.231617	2.847642
H	-0.222934	0.525511	3.067549
H	-1.532269	-0.528029	3.607411
O	-3.676408	-0.333109	-0.399543
Si	-4.777820	0.841144	0.140388
C	-4.683272	1.011864	1.999214
C	-6.512674	0.325550	-0.325276
C	-4.290626	2.428844	-0.776414
H	-5.571579	1.542858	2.354855
H	-4.679534	0.030079	2.481527
H	-3.806523	1.561579	2.345761
H	-7.193977	1.168857	-0.174571
H	-6.591994	0.016414	-1.369657
H	-6.868050	-0.494873	0.302669
C	-5.124427	3.593995	-0.218767
H	-4.878509	4.517404	-0.755556
H	-6.199098	3.427284	-0.335274
H	-4.924483	3.770182	0.841930
C	-4.575068	2.282151	-2.278387
H	-4.012356	1.455578	-2.721188
H	-5.636133	2.112979	-2.479779
H	-4.283550	3.198117	-2.805604
C	-2.802258	2.742928	-0.580904
H	-2.550322	3.684389	-1.083067
H	-2.539752	2.857542	0.474786
H	-2.169052	1.961617	-1.006331
C	-3.537185	-2.725836	-0.688422
C	-5.034954	-2.943316	-0.426911
C	-3.309392	-2.542505	-2.199396
C	-2.774375	-3.968745	-0.223585
H	-5.244309	-3.017786	0.643775
H	-5.637021	-2.137151	-0.842548

H	-5.355586	-3.875887	-0.897966
H	-2.247436	-2.411543	-2.422972
H	-3.661087	-3.428634	-2.733987
H	-3.851307	-1.675872	-2.579941
H	-3.141929	-4.837197	-0.774972
H	-1.702371	-3.884890	-0.411654
H	-2.924714	-4.164696	0.840722

TS-AD_tBu_2d

SMD(DCE)-CAM-B3LYP/6-311+G(d,p)

E = -2553.233387
Zero-point correction = 0.638784 (Hartree/Particle)
Thermal correction to Energy = 0.680046
Thermal correction to Enthalpy = 0.680990
Thermal correction to Gibbs Free Energy = 0.561578
Sum of electronic and zero-point Energies = -2552.594602
Sum of electronic and thermal Energies = -2552.553341
Sum of electronic and thermal Enthalpies = -2552.552397
Sum of electronic and thermal Free Energies = -2552.671809

C	0.418635	-0.369952	-0.149695
H	0.163493	-1.347138	0.237490
H	-0.033610	0.474569	0.353193
C	1.668272	-0.219348	-0.703756
S	2.306994	1.388689	-1.058433
O	3.729357	1.251506	-1.371521
O	1.433130	2.058370	-2.023776
S	2.616391	-1.650960	-1.145030
O	1.764132	-2.795533	-0.806638
O	3.119683	-1.536629	-2.511841
C	2.165536	2.281665	0.485554
C	3.011330	1.941556	1.534413
C	1.240251	3.307654	0.596454
C	2.919851	2.648573	2.722714
H	3.733533	1.141615	1.424286
C	1.160937	4.011354	1.792000
H	0.601793	3.555508	-0.241528
C	1.995684	3.680943	2.850634
H	3.573484	2.396993	3.548943
H	0.446380	4.819283	1.892285
H	1.930457	4.232267	3.781167
C	4.020070	-1.695984	-0.041343
C	3.832168	-2.157039	1.256134
C	5.265894	-1.301142	-0.504625
C	4.923673	-2.216581	2.109150
H	2.852142	-2.473214	1.590936
C	6.351317	-1.370577	0.358211
H	5.377390	-0.944431	-1.519039
C	6.180059	-1.824001	1.660064
H	4.794561	-2.576992	3.122448
H	7.331850	-1.068847	0.010573
H	7.031005	-1.875210	2.329153
C	-1.164378	-0.501187	-1.603179
C	-1.826762	0.750530	-1.493690

C	-0.464776	-0.841337	-2.881724
H	-1.606255	-1.336070	-1.076061
H	-1.441282	1.554489	-2.105804
C	-2.864365	1.041681	-0.656644
H	0.158051	-0.015307	-3.229321
H	0.149861	-1.735933	-2.786454
H	-1.214540	-1.040936	-3.656608
O	-3.398724	0.106909	0.151594
Si	-4.400727	-1.263456	0.119037
C	-4.251641	-2.137125	-1.526193
C	-6.177852	-0.737135	0.361029
C	-3.819970	-2.328037	1.577572
H	-5.093258	-2.827100	-1.641385
H	-4.303191	-1.424166	-2.354232
H	-3.331884	-2.714729	-1.633587
H	-6.790376	-1.617623	0.579248
H	-6.298552	-0.035101	1.188863
H	-6.584681	-0.275598	-0.541880
C	-4.562571	-3.673461	1.529689
H	-4.263154	-4.293756	2.382224
H	-5.647974	-3.550012	1.583401
H	-4.332612	-4.235875	0.620367
C	-4.138409	-1.626159	2.905828
H	-3.637021	-0.657565	2.985643
H	-5.211381	-1.461600	3.035547
H	-3.797735	-2.242853	3.745795
C	-2.310672	-2.590026	1.502803
H	-2.002936	-3.237880	2.331981
H	-2.023671	-3.093097	0.574791
H	-1.738350	-1.663106	1.579066
C	-3.460461	2.432904	-0.492553
C	-4.938265	2.413229	-0.913479
C	-3.363704	2.831936	0.989972
C	-2.729631	3.481786	-1.334390
H	-5.050568	2.082285	-1.949447
H	-5.529829	1.759472	-0.274773
H	-5.353890	3.421004	-0.834726
H	-2.322582	2.871410	1.321171
H	-3.802006	3.822863	1.132953
H	-3.898399	2.126733	1.626869
H	-3.181307	4.459965	-1.154322
H	-1.671101	3.550825	-1.074637
H	-2.807780	3.274273	-2.403967

TS-AD_Mes_1a

<u>SMD(DCE)-CAM-B3LYP/6-311+G(d,p)</u>

E = -2744.925309
Zero-point correction = 0.690118 (Hartree/Particle)
Thermal correction to Energy = 0.735605
Thermal correction to Enthalpy = 0.736549
Thermal correction to Gibbs Free Energy = 0.607942
Sum of electronic and zero-point Energies = -2744.235263

Sum of electronic and thermal Energies = -2744.189776
Sum of electronic and thermal Enthalpies = -2744.188832
Sum of electronic and thermal Free Energies = -2744.317439

C	-0.928913	-0.707348	0.022983
H	-0.348428	-0.238135	0.806160
H	-0.638364	-1.709369	-0.260049
C	-2.273258	-0.403202	-0.055355
S	-3.378299	-1.482514	-0.920124
O	-4.346637	-0.690858	-1.679561
O	-2.530365	-2.441702	-1.632437
S	-2.918322	1.030437	0.745748
O	-4.272413	0.724491	1.207424
O	-1.907675	1.451287	1.717710
C	-4.280841	-2.399264	0.319921
C	-5.586914	-2.040319	0.615835
C	-3.654253	-3.467925	0.948550
C	-6.282631	-2.775272	1.565209
H	-6.045909	-1.200685	0.112908
C	-4.358340	-4.189678	1.900264
H	-2.638065	-3.739489	0.692513
C	-5.669294	-3.843471	2.207290
H	-7.305046	-2.509587	1.804691
H	-3.883982	-5.026896	2.397448
H	-6.216343	-4.411539	2.950420
C	-3.044525	2.333074	-0.472587
C	-4.195352	2.430517	-1.244497
C	-1.993958	3.230105	-0.610224
C	-4.287066	3.450229	-2.179560
H	-4.998429	1.718514	-1.116548
C	-2.099602	4.245705	-1.550318
H	-1.117064	3.148226	0.018865
C	-3.241109	4.352796	-2.334304
H	-5.179174	3.541341	-2.787163
H	-1.290785	4.957129	-1.664706
H	-3.319921	5.147583	-3.066761
C	0.208168	0.246726	-1.465414
C	1.389819	-0.542883	-1.469417
C	-0.655211	0.248736	-2.689540
H	0.263440	1.185935	-0.925056
H	1.446789	-1.364398	-2.175631
C	2.446665	-0.391957	-0.622717
H	-0.805758	-0.759676	-3.078157
H	-1.629923	0.700286	-2.503154
H	-0.167609	0.839920	-3.473253
O	2.404537	0.545960	0.341343
Si	3.495297	1.764613	0.838494
C	3.733313	2.913173	-0.613969
C	5.148188	1.047700	1.331497
C	2.616701	2.585215	2.300289
H	4.363156	3.763462	-0.336971
H	4.234185	2.389449	-1.432960
H	2.787330	3.307474	-0.992385
H	5.809033	1.857947	1.654803

H	5.077653	0.327920	2.149050
H	5.631946	0.554238	0.485620
C	3.498544	3.733343	2.821867
H	3.008061	4.219751	3.673020
H	4.475485	3.382698	3.166079
H	3.663440	4.501450	2.060404
C	2.398727	1.565969	3.427739
H	1.759361	0.739873	3.106424
H	3.340652	1.147506	3.792093
H	1.904404	2.050616	4.277924
C	1.258347	3.154220	1.871374
H	0.771597	3.643337	2.723804
H	1.358147	3.904942	1.081557
H	0.581982	2.371912	1.522221
C	3.637800	-1.282615	-0.682209
C	3.926016	-2.119254	0.411880
C	4.455657	-1.310263	-1.822477
C	5.029470	-2.960374	0.345893
C	5.558407	-2.163111	-1.838375
C	5.865846	-2.990939	-0.766619
H	5.238200	-3.616570	1.184881
H	6.193254	-2.176997	-2.718424
C	3.059887	-2.146840	1.643470
H	3.297616	-1.319610	2.315881
H	1.998740	-2.069498	1.398323
H	3.215176	-3.075775	2.194290
C	4.188938	-0.461397	-3.039776
H	3.460283	-0.939264	-3.701442
H	3.793766	0.521827	-2.783938
H	5.107540	-0.325560	-3.613110
C	7.073404	-3.886884	-0.797249
H	7.927017	-3.406776	-0.308568
H	6.883824	-4.824740	-0.271110
H	7.369139	-4.120611	-1.821444

TS-AD_Mes_1b

SMD(DCE)-CAM-B3LYP/6-311+G(d,p)

E = -2744.923295
Zero-point correction = 0.689934 (Hartree/Particle)
Thermal correction to Energy = 0.735546
Thermal correction to Enthalpy = 0.736490
Thermal correction to Gibbs Free Energy = 0.607124
Sum of electronic and zero-point Energies = -2744.233329
Sum of electronic and thermal Energies = -2744.187718
Sum of electronic and thermal Enthalpies = -2744.186774
Sum of electronic and thermal Free Energies = -2744.316140

C	-0.848381	-0.278433	0.548225
H	-0.250831	0.616852	0.438420
H	-0.521090	-0.979954	1.303784
C	-2.210521	-0.195934	0.331834
S	-3.317821	-1.298598	1.164638
O	-2.515257	-2.001835	2.168167
O	-4.471053	-0.507503	1.597669

S	-2.868349	1.122928	-0.644276
O	-1.738186	1.639924	-1.420019
O	-4.058265	0.648591	-1.351913
C	-3.924377	-2.546055	0.034965
C	-3.336960	-3.803948	0.053528
C	-4.999796	-2.254851	-0.794394
C	-3.833011	-4.788630	-0.789144
H	-2.515182	-4.011276	0.725871
C	-5.484398	-3.247966	-1.632565
H	-5.443407	-1.269499	-0.786614
C	-4.901266	-4.509872	-1.632265
H	-3.386684	-5.775596	-0.781428
H	-6.323220	-3.035170	-2.284113
H	-5.286492	-5.281756	-2.288111
C	-3.383113	2.426116	0.464741
C	-2.434900	3.337492	0.911738
C	-4.715540	2.512459	0.841767
C	-2.836721	4.358509	1.760324
H	-1.404493	3.261938	0.589015
C	-5.104812	3.542023	1.686431
H	-5.430524	1.786688	0.480688
C	-4.168121	4.459328	2.146846
H	-2.109733	5.079235	2.114324
H	-6.142537	3.625751	1.985552
H	-4.477932	5.260277	2.807749
C	0.189163	-1.100190	-1.083983
C	1.390039	-1.553001	-0.469155
C	-0.715376	-2.106159	-1.721201
H	0.229562	-0.137069	-1.578652
H	1.451099	-2.596629	-0.178475
C	2.458905	-0.767254	-0.163564
H	-0.821552	-3.002572	-1.108359
H	-1.705644	-1.695520	-1.921635
H	-0.289729	-2.411366	-2.684340
O	2.419702	0.560398	-0.394124
Si	3.193003	1.573032	-1.528580
C	2.393276	1.258381	-3.186140
C	5.014107	1.173286	-1.614543
C	2.873617	3.330449	-0.895337
H	2.922666	1.799581	-3.975941
H	2.431754	0.195290	-3.440870
H	1.346839	1.571571	-3.208106
H	5.516142	1.893605	-2.268463
H	5.498892	1.216262	-0.637331
H	5.184267	0.179688	-2.036112
C	3.141241	4.321613	-2.040041
H	3.010923	5.348158	-1.678867
H	4.161426	4.242347	-2.428336
H	2.451817	4.178685	-2.876065
C	3.810248	3.661931	0.275085
H	3.683982	2.970627	1.112602
H	4.861645	3.637639	-0.022960
H	3.598384	4.670741	0.648099

C	1.417401	3.480176	-0.434298
H	1.226744	4.517471	-0.134427
H	0.704321	3.229934	-1.224225
H	1.203235	2.840342	0.424701
C	3.675080	-1.300740	0.511657
C	3.953199	-0.903471	1.832674
C	4.526696	-2.196331	-0.147555
C	5.084887	-1.404458	2.462344
C	3.032393	0.018562	2.587320
C	5.657647	-2.666644	0.520512
C	4.264176	-2.673775	-1.552724
C	5.955268	-2.283567	1.820891
H	5.289434	-1.107111	3.486286
H	3.074179	1.037255	2.198028
H	1.992597	-0.309676	2.514091
H	3.303394	0.048154	3.643271
H	6.322326	-3.351118	0.003230
H	3.521989	-3.476571	-1.565727
H	3.886383	-1.878916	-2.196755
H	5.179725	-3.066658	-1.997490
C	7.181333	-2.795151	2.525983
H	7.897438	-1.987681	2.703006
H	6.926867	-3.219192	3.500465
H	7.683858	-3.566453	1.940556

TS-AD_Mes_1c

SMD(DCE)-CAM-B3LYP/6-311+G(d,p)

E = -2744.921583
Zero-point correction = 0.689538 (Hartree/Particle)
Thermal correction to Energy = 0.735394
Thermal correction to Enthalpy = 0.736338
Thermal correction to Gibbs Free Energy = 0.604515
Sum of electronic and zero-point Energies = -2744.232028
Sum of electronic and thermal Energies = -2744.186172
Sum of electronic and thermal Enthalpies = -2744.185228
Sum of electronic and thermal Free Energies = -2744.317050

C	-0.893491	-0.746724	-0.121799
H	-0.382206	0.117284	0.282490
H	-0.659525	-1.698422	0.335141
C	-2.162764	-0.588379	-0.634690
S	-3.185776	-2.017347	-0.895255
O	-3.579839	-2.134595	-2.297038
O	-2.451596	-3.131265	-0.287021
S	-2.735756	1.018930	-1.091799
O	-1.612081	1.725012	-1.710465
O	-3.991769	0.873334	-1.824357
C	-4.666897	-1.758969	0.066361
C	-5.854907	-1.465665	-0.585809
C	-4.601928	-1.896749	1.447614
C	-7.008688	-1.305157	0.169543
H	-5.870020	-1.365529	-1.662426
C	-5.760535	-1.729755	2.190342
H	-3.664502	-2.135663	1.934590

C	-6.960632	-1.435635	1.551710
H	-7.945507	-1.079431	-0.325205
H	-5.727695	-1.835583	3.267793
H	-7.864510	-1.309541	2.136116
C	-3.104855	1.865923	0.440011
C	-2.098611	2.574507	1.083532
C	-4.394976	1.804984	0.949589
C	-2.394286	3.230461	2.269651
H	-1.106661	2.629152	0.655385
C	-4.677997	2.468985	2.134859
H	-5.166455	1.259219	0.422232
C	-3.680180	3.176130	2.794416
H	-1.620260	3.791599	2.778956
H	-5.682030	2.436631	2.539916
H	-3.907285	3.694605	3.718461
C	0.558142	-0.899498	-1.670775
C	1.663361	-1.469081	-0.980868
C	-0.102718	-1.694387	-2.752103
H	0.573370	0.173662	-1.820708
H	1.736553	-2.551489	-0.953709
C	2.630732	-0.778424	-0.317198
H	-0.249271	-2.737102	-2.462334
H	-1.064494	-1.271023	-3.041648
H	0.537020	-1.686775	-3.642510
O	2.594425	0.569289	-0.238289
Si	3.500200	1.780576	-1.029206
C	2.897350	1.866602	-2.794623
C	5.317540	1.352696	-0.998870
C	3.130932	3.371570	-0.067919
H	3.517911	2.553408	-3.377903
H	2.962197	0.886496	-3.275717
H	1.862109	2.208651	-2.864762
H	5.894819	2.194046	-1.395433
H	5.683382	1.141968	0.007773
H	5.534851	0.486802	-1.628653
C	3.596312	4.571719	-0.909142
H	3.434861	5.500650	-0.350450
H	4.662081	4.521250	-1.152313
H	3.041078	4.654130	-1.847026
C	3.881443	3.384010	1.271081
H	3.613253	2.532629	1.902227
H	4.965530	3.368304	1.132073
H	3.635712	4.295528	1.828527
C	1.625571	3.502442	0.195879
H	1.415375	4.451598	0.702922
H	1.042708	3.490622	-0.729182
H	1.259719	2.695662	0.834701
C	3.735116	-1.461256	0.413649
C	3.794840	-1.353773	1.815614
C	4.697673	-2.213057	-0.273838
C	4.824858	-1.988251	2.498105
C	2.745582	-0.604039	2.592865
C	5.720388	-2.824027	0.451780

C	4.671203	-2.390700	-1.770193
C	5.805326	-2.721510	1.833425
H	4.858231	-1.915025	3.580674
H	2.810960	0.471882	2.424384
H	1.738531	-0.912975	2.300734
H	2.858573	-0.787713	3.661996
H	6.471654	-3.394237	-0.085389
H	3.984708	-3.190622	-2.061232
H	4.350763	-1.487493	-2.289590
H	5.662527	-2.662135	-2.136313
C	6.931225	-3.365191	2.595074
H	7.688042	-2.624156	2.869525
H	6.573971	-3.821104	3.520961
H	7.423755	-4.136161	2.000399

TS-AD_Mes_1d

SMD(DCE)-CAM-B3LYP/6-311+G(d,p)

E = -2744.921835
Zero-point correction = 0.689369 (Hartree/Particle)
Thermal correction to Energy = 0.735243
Thermal correction to Enthalpy = 0.736188
Thermal correction to Gibbs Free Energy = 0.604143
Sum of electronic and zero-point Energies = -2744.232373
Sum of electronic and thermal Energies = -2744.186499
Sum of electronic and thermal Enthalpies = -2744.185555
Sum of electronic and thermal Free Energies = -2744.317599

C	0.669037	-0.527898	0.276737
H	0.172890	0.201671	-0.349221
H	0.396380	-1.563479	0.126988
C	1.967261	-0.270988	0.673161
S	3.003615	-1.591773	1.218756
O	3.945108	-1.096062	2.221246
O	2.121935	-2.712937	1.550159
S	2.505289	1.415593	0.776086
O	1.557114	2.174379	-0.046132
O	2.668052	1.813021	2.174313
C	3.965267	-2.088484	-0.207441
C	5.343661	-1.950738	-0.167587
C	3.317822	-2.643306	-1.305710
C	6.092483	-2.382539	-1.255387
H	5.820292	-1.518044	0.701633
C	4.075601	-3.064018	-2.386633
H	2.240374	-2.752101	-1.319450
C	5.460976	-2.934482	-2.360851
H	7.171032	-2.284662	-1.235052
H	3.585205	-3.499009	-3.248969
H	6.048707	-3.268222	-3.207852
C	4.103634	1.512049	-0.007877
C	4.169884	1.478451	-1.395714
C	5.235255	1.675742	0.776891
C	5.406119	1.610349	-2.007944
H	3.270543	1.361008	-1.987256
C	6.466902	1.810903	0.150201

H	5.148342	1.701238	1.854747
C	6.550749	1.778301	-1.235689
H	5.475328	1.588990	-3.088595
H	7.360490	1.944823	0.747591
H	7.514320	1.886372	-1.719607
C	-0.694542	-0.163647	1.836314
C	-1.826912	-0.907680	1.402393
C	0.001143	-0.573416	3.096905
H	-0.725018	0.903131	1.642104
H	-1.928104	-1.929309	1.753205
C	-2.775320	-0.457068	0.535644
H	0.173557	-1.650103	3.133701
H	0.955064	-0.058445	3.217382
H	-0.625745	-0.303067	3.954611
O	-2.662737	0.769728	-0.010405
Si	-3.663355	2.152645	0.017208
C	-3.706677	2.763265	1.781266
C	-5.387489	1.728129	-0.561021
C	-2.811830	3.380886	-1.147937
H	-4.285564	3.688889	1.855746
H	-4.179171	2.029514	2.440151
H	-2.704385	2.963334	2.166597
H	-6.018459	2.620839	-0.513484
H	-5.405842	1.361775	-1.590078
H	-5.848676	0.970034	0.075840
C	-3.770233	4.555356	-1.407445
H	-3.277103	5.302569	-2.039627
H	-4.679495	4.238206	-1.924874
H	-4.068653	5.060256	-0.483470
C	-2.473359	2.711127	-2.486679
H	-1.759104	1.894396	-2.360945
H	-3.361716	2.310342	-2.983457
H	-2.023066	3.444184	-3.166301
C	-1.522503	3.923603	-0.515574
H	-1.038530	4.625727	-1.204985
H	-1.721135	4.466149	0.412849
H	-0.799253	3.134640	-0.296782
C	-3.916169	-1.308846	0.094437
C	-3.990753	-1.710457	-1.251892
C	-4.899742	-1.718990	1.004684
C	-5.050488	-2.510094	-1.659919
C	-2.939488	-1.312031	-2.253433
C	-5.950652	-2.514686	0.548689
C	-4.873020	-1.317064	2.456986
C	-6.045226	-2.920926	-0.775458
H	-5.097177	-2.827019	-2.697247
H	-3.032116	-0.261814	-2.536104
H	-1.932053	-1.447044	-1.852969
H	-3.028250	-1.913132	-3.159186
H	-6.717200	-2.820136	1.253483
H	-4.140329	-1.899447	3.021220
H	-4.614584	-0.264810	2.585541
H	-5.849334	-1.485207	2.913969

C	-7.180751	-3.786350	-1.249033
H	-7.734059	-3.303308	-2.058617
H	-6.811949	-4.740536	-1.635052
H	-7.881568	-3.999113	-0.440519

TS-AD_Mes_2a

SMD(DCE)-CAM-B3LYP/6-311+G(d,p)

not determined => converged to **TS-AD_Mes_1a**

TS-AD_Mes_2b

SMD(DCE)-CAM-B3LYP/6-311+G(d,p)

E = -2744.923877
Zero-point correction = 0.689909 (Hartree/Particle)
Thermal correction to Energy = 0.735663
Thermal correction to Enthalpy = 0.736607
Thermal correction to Gibbs Free Energy = 0.605375
Sum of electronic and zero-point Energies = -2744.233967
Sum of electronic and thermal Energies = -2744.188214
Sum of electronic and thermal Enthalpies = -2744.187270
Sum of electronic and thermal Free Energies = -2744.318502

C	1.080190	0.778376	0.241733
H	1.154460	1.758916	0.692808
H	0.323463	0.650886	-0.518812
C	2.248426	0.057694	0.118239
S	2.346457	-1.310416	-0.993124
O	1.223690	-1.176758	-1.924451
O	3.710543	-1.359680	-1.520793
S	3.700911	0.568641	0.990323
O	3.262398	1.615954	1.917814
O	4.399121	-0.599222	1.529266
C	2.071335	-2.818282	-0.071125
C	0.833714	-3.440534	-0.160780
C	3.107255	-3.356908	0.682911
C	0.628716	-4.628205	0.528729
H	0.051945	-3.010997	-0.772300
C	2.887022	-4.541559	1.368753
H	4.061984	-2.852608	0.738334
C	1.650933	-5.174430	1.293641
H	-0.330007	-5.127948	0.461617
H	3.684953	-4.973421	1.960529
H	1.486958	-6.101935	1.829573
C	4.783459	1.344029	-0.201997
C	4.442077	2.594271	-0.701900
C	5.950520	0.699236	-0.579723
C	5.295509	3.208675	-1.605378
H	3.529059	3.085056	-0.388532
C	6.800576	1.328599	-1.478788
H	6.185756	-0.276320	-0.176894
C	6.472447	2.576733	-1.992024
H	5.043254	4.183612	-2.004290
H	7.718412	0.838840	-1.780574
H	7.137474	3.061737	-2.696828

C	-0.196986	0.130591	1.811617
C	-1.204013	-0.639525	1.172873
C	0.697915	-0.526844	2.816201
H	-0.457846	1.163632	2.013433
H	-1.126535	-1.720403	1.201687
C	-2.256747	-0.113014	0.483161
H	1.090406	-1.476656	2.447164
H	1.532981	0.115355	3.094438
H	0.125685	-0.738371	3.727036
O	-2.340517	1.219019	0.334429
Si	-3.592376	2.381157	0.336450
C	-4.554240	2.194312	1.925405
C	-4.726569	2.165534	-1.129461
C	-2.640596	4.017473	0.251431
H	-5.218199	3.052671	2.066225
H	-5.180604	1.299044	1.903914
H	-3.899183	2.131856	2.797558
H	-5.514078	2.924413	-1.088423
H	-4.207265	2.276731	-2.083590
H	-5.214635	1.188701	-1.117490
C	-3.641017	5.176249	0.113797
H	-3.102918	6.130787	0.091049
H	-4.223352	5.108233	-0.809046
H	-4.342369	5.218988	0.952365
C	-1.701388	4.021770	-0.963050
H	-0.958593	3.222408	-0.904667
H	-2.246848	3.905703	-1.903901
H	-1.161073	4.974298	-1.014583
C	-1.814632	4.221373	1.529316
H	-1.259072	5.164532	1.469766
H	-2.446069	4.269809	2.420806
H	-1.086330	3.419693	1.677171
C	-3.282397	-0.968546	-0.176746
C	-3.288977	-1.070008	-1.580269
C	-4.213515	-1.683018	0.587275
C	-4.242473	-1.873575	-2.190918
C	-5.161758	-2.467960	-0.069903
C	-5.195057	-2.574568	-1.453358
H	-4.240086	-1.959144	-3.273090
H	-5.891024	-3.011254	0.522640
C	-2.269171	-0.350802	-2.422795
H	-2.387781	0.732593	-2.361609
H	-1.250018	-0.583868	-2.104045
H	-2.367411	-0.639702	-3.469895
C	-4.216532	-1.648637	2.094030
H	-3.491088	-2.358204	2.502367
H	-3.961080	-0.665102	2.487703
H	-5.198796	-1.927901	2.478383
C	-6.234948	-3.411824	-2.145841
H	-6.989511	-2.779205	-2.622622
H	-5.788248	-4.028452	-2.929102
H	-6.747728	-4.069355	-1.442215

TS-AD_Mes_2c

SMD(DCE)-CAM-B3LYP/6-311+G(d,p)

E = -2744.920545
Zero-point correction = 0.689346 (Hartree/Particle)
Thermal correction to Energy = 0.735291
Thermal correction to Enthalpy = 0.736235
Thermal correction to Gibbs Free Energy = 0.604145
Sum of electronic and zero-point Energies = -2744.231199
Sum of electronic and thermal Energies = -2744.185254
Sum of electronic and thermal Enthalpies = -2744.184310
Sum of electronic and thermal Free Energies = -2744.316400

C	0.724670	0.750792	0.443142
H	0.733879	1.826160	0.563409
H	0.060220	0.349320	-0.308012
C	1.901159	0.078416	0.694279
S	1.977829	-1.655501	0.328512
O	1.937272	-2.435207	1.565747
O	0.926005	-1.890744	-0.666300
S	3.236033	0.894106	1.507750
O	2.684104	2.109189	2.112955
O	3.960853	-0.057181	2.348859
C	3.547522	-1.969331	-0.457379
C	4.544648	-2.616465	0.257175
C	3.712751	-1.611531	-1.790084
C	5.739821	-2.912200	-0.384235
H	4.381900	-2.886098	1.291832
C	4.912538	-1.908990	-2.416906
H	2.914384	-1.118969	-2.330813
C	5.921980	-2.559371	-1.715059
H	6.527326	-3.421302	0.157892
H	5.057005	-1.639475	-3.455929
H	6.856203	-2.794246	-2.211515
C	4.358374	1.421589	0.215968
C	3.905956	2.299137	-0.762685
C	5.673544	0.987177	0.253736
C	4.797153	2.744625	-1.725249
H	2.875513	2.632481	-0.776695
C	6.559079	1.446791	-0.713536
H	5.997311	0.304596	1.027985
C	6.122176	2.319730	-1.699628
H	4.458564	3.427384	-2.495000
H	7.590847	1.117725	-0.693725
H	6.815588	2.673981	-2.453195
C	-0.720350	0.516428	1.991806
C	-1.605973	-0.487742	1.518496
C	0.070695	0.257358	3.236782
H	-1.047886	1.538967	1.838954
H	-1.466419	-1.506765	1.862568
C	-2.612905	-0.269648	0.625748
H	0.559574	-0.718695	3.206214
H	0.825821	1.024841	3.402844
H	-0.604432	0.262219	4.100525
O	-2.762403	0.955085	0.090890

Si	-4.078511	1.957582	-0.328169
C	-5.140506	2.185814	1.190145
C	-5.087977	1.217811	-1.712599
C	-3.223854	3.562495	-0.863808
H	-5.861902	2.992841	1.029590
H	-5.710262	1.279867	1.411380
H	-4.546666	2.438526	2.071759
H	-5.909962	1.896307	-1.961356
H	-4.503544	1.055779	-2.620719
H	-5.530456	0.264138	-1.417476
C	-4.278694	4.543721	-1.399458
H	-3.797887	5.485702	-1.687162
H	-4.789018	4.154531	-2.284614
H	-5.039231	4.783252	-0.650418
C	-2.202510	3.273945	-1.973213
H	-1.416345	2.595119	-1.633669
H	-2.670819	2.831588	-2.857151
H	-1.721773	4.206637	-2.290981
C	-2.504150	4.206335	0.329593
H	-1.998688	5.125634	0.011403
H	-3.199652	4.475841	1.129188
H	-1.745748	3.543562	0.754506
C	-3.521908	-1.357800	0.167677
C	-3.390616	-1.853726	-1.143147
C	-4.483012	-1.895203	1.031699
C	-4.239542	-2.866136	-1.566880
C	-5.323442	-2.904890	0.558989
C	-5.219756	-3.401638	-0.731996
H	-4.130994	-3.253260	-2.575371
H	-6.076463	-3.311979	1.226163
C	-2.328678	-1.330729	-2.073344
H	-2.479000	-0.274883	-2.306257
H	-1.334966	-1.429997	-1.628678
H	-2.336401	-1.886789	-3.011615
C	-4.632886	-1.438081	2.459944
H	-3.943597	-1.978786	3.115084
H	-4.425000	-0.375430	2.581592
H	-5.644996	-1.633572	2.818061
C	-6.128800	-4.493670	-1.225221
H	-6.696940	-4.167142	-2.100326
H	-5.555035	-5.375236	-1.523507
H	-6.838390	-4.797405	-0.454310

TS-AD_Mes_2d

SMD(DCE)-CAM-B3LYP/6-311+G(d,p)

E = -2744.919603
Zero-point correction = 0.689533 (Hartree/Particle)
Thermal correction to Energy = 0.735496
Thermal correction to Enthalpy = 0.736440
Thermal correction to Gibbs Free Energy = 0.604813
Sum of electronic and zero-point Energies = -2744.230070
Sum of electronic and thermal Energies = -2744.184108
Sum of electronic and thermal Enthalpies = -2744.183163

Sum of electronic and thermal Free Energies = -2744.314790

C	-0.873964	0.838754	-0.519111
H	-0.933004	1.918163	-0.473955
H	-0.189090	0.361447	0.166981
C	-2.015074	0.164433	-0.892781
S	-2.174819	-1.561045	-0.561074
O	-3.235095	-2.112726	-1.402375
O	-0.829888	-2.136219	-0.617552
S	-3.324764	1.048366	-1.702029
O	-2.942666	2.462805	-1.622299
O	-3.605568	0.485920	-3.020660
C	-2.728679	-1.698299	1.136821
C	-4.039183	-2.075289	1.388415
C	-1.831497	-1.449104	2.169189
C	-4.459714	-2.204723	2.705935
H	-4.714204	-2.272586	0.566670
C	-2.265260	-1.575610	3.479476
H	-0.806071	-1.175007	1.957276
C	-3.577396	-1.952325	3.747244
H	-5.479312	-2.504821	2.914934
H	-1.575556	-1.387514	4.293317
H	-3.909903	-2.053953	4.773550
C	-4.787389	0.830270	-0.701546
C	-4.885001	1.515500	0.503546
C	-5.813547	0.024361	-1.170779
C	-6.041903	1.383138	1.255183
H	-4.075354	2.148317	0.845270
C	-6.969010	-0.096147	-0.409985
H	-5.706092	-0.493101	-2.114433
C	-7.081532	0.580042	0.797792
H	-6.134894	1.912292	2.195662
H	-7.781599	-0.718431	-0.764737
H	-7.985913	0.483767	1.386994
C	0.631570	0.984383	-2.041364
C	1.544642	-0.068168	-1.781258
C	-0.128172	0.986131	-3.330961
H	0.914558	1.956252	-1.652043
H	1.458346	-0.984260	-2.354248
C	2.523707	-0.023689	-0.832974
H	-0.587862	0.015790	-3.528707
H	-0.902986	1.752136	-3.347094
H	0.560577	1.198850	-4.157015
O	2.598500	1.043373	-0.018364
Si	3.832520	1.904854	0.788229
C	5.090153	2.462040	-0.473262
C	4.652651	0.854584	2.095055
C	2.888750	3.359194	1.555064
H	5.778175	3.183935	-0.022689
H	5.689144	1.621388	-0.831416
H	4.622545	2.940685	-1.336789
H	5.415120	1.449512	2.607294
H	3.948956	0.495284	2.849012
H	5.154320	-0.010517	1.656719

C	3.850757	4.179631	2.429030
H	3.324513	5.042851	2.852299
H	4.244394	3.595603	3.265333
H	4.700633	4.565393	1.858326
C	1.737099	2.843883	2.429620
H	1.009964	2.273260	1.846514
H	2.093565	2.204864	3.242673
H	1.206910	3.687965	2.886017
C	2.319390	4.264635	0.453889
H	1.760613	5.094402	0.902255
H	3.108231	4.699295	-0.166151
H	1.634593	3.723935	-0.204593
C	3.485778	-1.143996	-0.628738
C	3.318787	-1.999913	0.475037
C	4.532269	-1.355764	-1.533321
C	4.223068	-3.034793	0.667122
C	5.425243	-2.402323	-1.295743
C	5.291474	-3.247363	-0.203510
H	4.089728	-3.698057	1.516130
H	6.244017	-2.557636	-1.991007
C	2.155642	-1.832936	1.416048
H	2.154330	-0.848432	1.887781
H	1.209577	-1.942668	0.879469
H	2.187317	-2.585620	2.204891
C	4.711880	-0.512258	-2.768991
H	4.088240	-0.887134	-3.585943
H	4.437308	0.529429	-2.606827
H	5.748665	-0.545799	-3.107512
C	6.264742	-4.367471	0.042564
H	6.805782	-4.217028	0.980897
H	5.747392	-5.327280	0.118710
H	6.998437	-4.440126	-0.761604

TS-AD_Ph_1a

SMD(DCE)-CAM-B3LYP/6-311+G(d,p)

E = -2627.023676
Zero-point correction = 0.607680 (Hartree/Particle)
Thermal correction to Energy = 0.647991
Thermal correction to Enthalpy = 0.648936
Thermal correction to Gibbs Free Energy = 0.530769
Sum of electronic and zero-point Energies = -2626.415996
Sum of electronic and thermal Energies = -2626.375685
Sum of electronic and thermal Enthalpies = -2626.374740
Sum of electronic and thermal Free Energies = -2626.492907

C	-0.653987	-0.833935	-0.033454
H	0.035517	-0.513669	0.736341
H	-0.548786	-1.850991	-0.384893
C	-1.927545	-0.305153	-0.027930
S	-3.233155	-1.134766	-0.887613
O	-4.105347	-0.149662	-1.527286
O	-2.585989	-2.154481	-1.716457
S	-2.286964	1.165428	0.881560

O	-3.643302	1.049866	1.416961
O	-1.165658	1.365385	1.801999
C	-4.195580	-1.995580	0.347097
C	-5.435929	-1.497774	0.714804
C	-3.682558	-3.164339	0.895656
C	-6.183001	-2.194571	1.654684
H	-5.806304	-0.583004	0.272849
C	-4.435622	-3.846984	1.838382
H	-2.715895	-3.541071	0.586033
C	-5.683221	-3.362620	2.216112
H	-7.156133	-1.821357	1.949731
H	-4.049747	-4.760527	2.274031
H	-6.269109	-3.900827	2.951842
C	-2.270517	2.538831	-0.262132
C	-3.429816	2.864587	-0.953894
C	-1.095919	3.261902	-0.422607
C	-3.403235	3.938499	-1.831356
H	-4.331138	2.285385	-0.810261
C	-1.083373	4.333467	-1.304098
H	-0.214681	3.002983	0.149098
C	-2.233091	4.667422	-2.009297
H	-4.299948	4.206099	-2.376914
H	-0.175689	4.909984	-1.435298
H	-2.219459	5.505008	-2.696763
C	0.549985	0.007186	-1.537283
C	1.579926	-0.963903	-1.604762
C	-0.345074	0.204007	-2.720996
H	0.763476	0.896449	-0.955069
H	1.455165	-1.775558	-2.312394
C	2.658516	-1.023259	-0.766065
H	-0.662452	-0.746984	-3.151292
H	-1.232415	0.785591	-2.468320
H	0.197861	0.759118	-3.494660
O	2.816590	-0.100275	0.204015
C	3.630998	-2.132317	-0.791586
Si	3.871020	1.241120	0.308732
C	3.888678	-2.854347	-1.962494
C	4.306957	-2.488975	0.378906
C	3.336876	2.480780	-0.981838
C	5.625358	0.707209	-0.042811
C	3.650239	1.873783	2.078728
C	4.789233	-3.907624	-1.956552
H	3.397324	-2.579833	-2.887710
C	5.201765	-3.548526	0.383016
H	4.113872	-1.941069	1.291900
H	3.955254	3.381839	-0.925167
H	3.455980	2.069017	-1.988336
H	2.294642	2.783821	-0.863519
H	6.266744	1.591403	-0.108800
H	6.035327	0.053749	0.729755
H	5.697660	0.183786	-0.999760
C	5.448594	-4.260558	-0.784109
H	4.984268	-4.450511	-2.874038

H	5.709206	-3.818676	1.301802
H	6.152869	-5.084227	-0.782168
C	4.735542	2.925926	2.360570
H	4.599978	3.338769	3.366635
H	5.742286	2.502338	2.313977
H	4.692082	3.763828	1.658107
C	3.797639	0.721074	3.081886
H	3.012919	-0.028450	2.955255
H	4.764944	0.217863	2.993099
H	3.724036	1.107262	4.105206
C	2.270829	2.523472	2.255504
H	2.151956	2.868106	3.289413
H	2.147507	3.396015	1.607980
H	1.452155	1.829325	2.050573

TS-AD_Ph_1b

SMD(DCE)-CAM-B3LYP/6-311+G(d,p)

E = -2627.016119
Zero-point correction = 0.607439 (Hartree/Particle)
Thermal correction to Energy = 0.647875
Thermal correction to Enthalpy = 0.648819
Thermal correction to Gibbs Free Energy = 0.529235
Sum of electronic and zero-point Energies = -2626.414650
Sum of electronic and thermal Energies = -2626.374214
Sum of electronic and thermal Enthalpies = -2626.373270
Sum of electronic and thermal Free Energies = -2626.492854

C	0.479918	-0.231964	-0.600187
H	-0.174549	0.625568	-0.524133
H	0.212976	-0.974089	-1.339610
C	1.821121	-0.068139	-0.332647
S	3.017205	-1.144875	-1.069511
O	2.300334	-1.918200	-2.086536
O	4.157158	-0.319316	-1.472048
S	2.365756	1.330561	0.600945
O	1.181514	1.812192	1.316423
O	3.558723	0.968015	1.366835
C	3.613944	-2.325031	0.135853
C	3.105593	-3.616645	0.110568
C	4.605292	-1.948268	1.032846
C	3.595488	-4.548575	1.014800
H	2.349673	-3.889559	-0.613617
C	5.083681	-2.888992	1.932716
H	4.987564	-0.937364	1.030312
C	4.578948	-4.184305	1.925565
H	3.210858	-5.561165	1.002500
H	5.856156	-2.609261	2.638703
H	4.959286	-4.914847	2.629852
C	2.832917	2.609037	-0.557256
C	1.843638	3.435676	-1.074447
C	4.167590	2.759738	-0.904656
C	2.205660	4.436521	-1.963938
H	0.811135	3.310675	-0.774367
C	4.516498	3.768300	-1.791432

H	4.915005	2.098383	-0.489381
C	3.538784	4.601122	-2.321680
H	1.446087	5.091938	-2.372329
H	5.555076	3.901755	-2.068719
H	3.817573	5.385944	-3.014966
C	-0.611003	-1.091631	1.031882
C	-1.651279	-1.761896	0.338690
C	0.377739	-1.896107	1.812826
H	-0.822600	-0.111257	1.434680
H	-1.450135	-2.784413	0.046838
C	-2.841366	-1.227365	-0.067619
H	0.639017	-2.824298	1.302439
H	1.291940	-1.338003	2.019001
H	-0.063525	-2.163032	2.780573
O	-3.188082	0.034751	0.235254
Si	-3.709072	1.047502	1.494480
C	-2.714600	0.759437	3.050476
C	-5.502542	0.705258	1.892096
C	-3.475764	2.791798	0.790962
H	-3.221837	1.250313	3.886927
H	-2.654910	-0.305684	3.291297
H	-1.698981	1.155985	3.001943
H	-5.840213	1.422295	2.647396
H	-6.160434	0.800553	1.026571
H	-5.633311	-0.294462	2.314335
C	-3.691948	3.813891	1.918691
H	-3.590788	4.831064	1.523211
H	-4.689130	3.735886	2.362205
H	-2.957025	3.701024	2.720349
C	-4.492996	3.058520	-0.327711
H	-4.391500	2.344957	-1.150154
H	-5.523239	3.010503	0.034812
H	-4.337987	4.061641	-0.742029
C	-2.058241	2.958522	0.227480
H	-1.921293	3.985060	-0.133012
H	-1.285599	2.768562	0.976619
H	-1.883448	2.285451	-0.615522
C	-3.836556	-1.957475	-0.988562
C	-4.770295	-1.233031	-1.747189
C	-3.825129	-3.359770	-1.083530
C	-5.658332	-1.893125	-2.591719
H	-4.785056	-0.149393	-1.678693
C	-4.717643	-4.014664	-1.925376
H	-3.132493	-3.940983	-0.482447
C	-5.636767	-3.285318	-2.683642
H	-6.369125	-1.319388	-3.178811
H	-4.703524	-5.098813	-1.982654
H	-6.333274	-3.800186	-3.338356

TS-AD_Ph_1c

SMD(DCE)-CAM-B3LYP/6-311+G(d,p)

E = -2627.021272
Zero-point correction = 0.607538 (Hartree/Particle)

Thermal correction to Energy = 0.647875
Thermal correction to Enthalpy = 0.648819
Thermal correction to Gibbs Free Energy = 0.530785
Sum of electronic and zero-point Energies = -2626.413729
Sum of electronic and thermal Energies = -2626.373392
Sum of electronic and thermal Enthalpies = -2626.372448
Sum of electronic and thermal Free Energies = -2626.490482

C	-0.589124	-0.856389	-0.034417
H	0.013876	-0.042973	0.348780
H	-0.485508	-1.806979	0.470138
C	-1.812503	-0.573534	-0.597517
S	-2.992260	-1.881695	-0.828840
O	-3.370916	-2.006716	-2.234218
O	-2.406322	-3.049247	-0.162942
S	-2.181886	1.070366	-1.128194
O	-0.973783	1.613407	-1.751782
O	-3.435717	1.045341	-1.878283
C	-4.448890	-1.411433	0.089120
C	-5.578365	-0.994506	-0.598794
C	-4.427302	-1.508529	1.475135
C	-6.717147	-0.666687	0.124630
H	-5.560074	-0.928587	-1.677991
C	-5.569850	-1.174341	2.185605
H	-3.536876	-1.845783	1.990998
C	-6.711669	-0.755449	1.510934
H	-7.608982	-0.342792	-0.397913
H	-5.570451	-1.246743	3.266314
H	-7.603592	-0.499391	2.070721
C	-2.468363	2.011698	0.365068
C	-1.391935	2.612109	1.004671
C	-3.763600	2.126677	0.851418
C	-1.622223	3.339119	2.163585
H	-0.394441	2.526766	0.594402
C	-3.980391	2.859492	2.009771
H	-4.587953	1.660725	0.327543
C	-2.912795	3.460744	2.665153
H	-0.792781	3.817497	2.670094
H	-4.986684	2.963427	2.396855
H	-3.088227	4.032627	3.568711
C	0.876908	-1.257256	-1.524371
C	1.865063	-1.904926	-0.746031
C	0.161323	-2.031877	-2.585613
H	1.016699	-0.202894	-1.728425
H	1.786943	-2.981337	-0.645218
C	2.853844	-1.280291	-0.034787
H	-0.095264	-3.040136	-2.253857
H	-0.746997	-1.528029	-2.916546
H	0.815372	-2.128450	-3.460020
O	2.968201	0.064867	-0.052591
Si	3.996376	1.062674	-0.986840
C	3.342170	1.112754	-2.734669
C	5.714927	0.333340	-0.993455
C	3.919391	2.758120	-0.145769

H	4.034401	1.666800	-3.375925
H	3.255673	0.104478	-3.149089
H	2.363889	1.593234	-2.806680
H	6.382142	0.968668	-1.584407
H	6.141321	0.237316	0.006487
H	5.718516	-0.656385	-1.458767
C	4.589776	3.790658	-1.067035
H	4.586750	4.774970	-0.585260
H	5.632612	3.538679	-1.282381
H	4.063862	3.892367	-2.019921
C	4.660314	2.736231	1.198082
H	4.220268	2.019993	1.897011
H	5.718102	2.486391	1.079644
H	4.607796	3.725108	1.668355
C	2.460689	3.170450	0.091670
H	2.421866	4.169805	0.540809
H	1.886667	3.208368	-0.838058
H	1.956456	2.480767	0.772536
C	3.781864	-2.006427	0.853064
C	4.382339	-1.338882	1.924780
C	4.072600	-3.363009	0.663265
C	5.234729	-2.009416	2.789548
H	4.165532	-0.291454	2.085522
C	4.930394	-4.028022	1.524327
H	3.643372	-3.898598	-0.173925
C	5.513390	-3.355932	2.593313
H	5.682836	-1.477535	3.620764
H	5.151305	-5.075520	1.355860
H	6.183570	-3.878695	3.265566

TS-AD_Ph_1d

SMD(DCE)-CAM-B3LYP/6-311+G(d,p)

E = -2627.020448
Zero-point correction = 0.607252 (Hartree/Particle)
Thermal correction to Energy = 0.647830
Thermal correction to Enthalpy = 0.648774
Thermal correction to Gibbs Free Energy = 0.528865
Sum of electronic and zero-point Energies = -2626.413230
Sum of electronic and thermal Energies = -2626.372653
Sum of electronic and thermal Enthalpies = -2626.371708
Sum of electronic and thermal Free Energies = -2626.491617

C	0.322791	0.522799	-0.257305
H	-0.236485	-0.217355	0.298221
H	0.104701	1.559870	-0.046616
C	1.590806	0.211043	-0.682969
S	2.704944	1.501475	-1.149426
O	3.639298	1.001854	-2.155921
O	1.886763	2.679013	-1.445922
S	2.039304	-1.496070	-0.868837
O	1.010415	-2.246300	-0.142843
O	2.254180	-1.817045	-2.278987
C	3.660184	1.883630	0.315769
C	5.028987	1.667337	0.299832

C	3.019283	2.428887	1.422524
C	5.774566	2.008753	1.421510
H	5.501438	1.244786	-0.576688
C	3.773351	2.759166	2.536871
H	1.949732	2.599083	1.417438
C	5.149184	2.550190	2.535610
H	6.845841	1.848590	1.420644
H	3.287822	3.185752	3.406133
H	5.734305	2.813194	3.408939
C	3.588004	-1.723993	-0.015182
C	3.590777	-1.735997	1.374671
C	4.742725	-1.936652	-0.752994
C	4.785859	-1.966993	2.036984
H	2.674510	-1.576902	1.929654
C	5.932280	-2.171526	-0.076328
H	4.705247	-1.923734	-1.833992
C	5.952691	-2.186264	1.312069
H	4.805521	-1.981933	3.119836
H	6.842613	-2.344951	-0.637184
H	6.883519	-2.371190	1.835279
C	-1.094920	0.356839	-1.855097
C	-2.089656	1.248923	-1.400291
C	-0.336898	0.673619	-3.105554
H	-1.247170	-0.695659	-1.648120
H	-2.034367	2.273554	-1.749565
C	-3.055326	0.950830	-0.473445
H	-0.050804	1.725603	-3.150655
H	0.560020	0.060223	-3.201712
H	-0.970207	0.458682	-3.974166
O	-3.104951	-0.276263	0.084256
Si	-4.099205	-1.627842	-0.237791
C	-3.559992	-2.381712	-1.858258
C	-5.876455	-1.074416	-0.381071
C	-3.803203	-2.782565	1.232727
H	-4.231393	-3.201034	-2.132340
H	-3.605406	-1.644456	-2.664808
H	-2.544045	-2.780770	-1.819372
H	-6.513691	-1.935778	-0.603837
H	-6.252606	-0.605561	0.529720
H	-6.000931	-0.362973	-1.202370
C	-4.559709	-4.098013	0.984438
H	-4.409440	-4.778923	1.829978
H	-5.637521	-3.942238	0.880019
H	-4.205156	-4.611902	0.086648
C	-4.315990	-2.146652	2.532259
H	-3.791688	-1.215797	2.763651
H	-5.387171	-1.930755	2.491106
H	-4.152296	-2.831705	3.372363
C	-2.306018	-3.085564	1.379775
H	-2.144011	-3.767437	2.222861
H	-1.893475	-3.564902	0.488441
H	-1.725862	-2.179988	1.570877
C	-4.005363	1.951824	0.044629

C	-4.565857	1.786865	1.315389
C	-4.358153	3.084627	-0.699633
C	-5.437531	2.733277	1.833368
H	-4.302977	0.917307	1.902995
C	-5.234299	4.024642	-0.181568
H	-3.964584	3.223116	-1.698643
C	-5.775787	3.855860	1.088291
H	-5.853781	2.593561	2.824322
H	-5.502309	4.890527	-0.775702
H	-6.460632	4.592763	1.491127

TS-AD_Ph_2a

<u>SMD(DCE)-CAM-B3LYP/6-311+G(d,p)</u>

E = -2627.018954
Zero-point correction = 0.607372 (Hartree/Particle)
Thermal correction to Energy = 0.647905
Thermal correction to Enthalpy = 0.648849
Thermal correction to Gibbs Free Energy = 0.528182
Sum of electronic and zero-point Energies = -2626.411603
Sum of electronic and thermal Energies = -2626.371069
Sum of electronic and thermal Enthalpies = -2626.370125
Sum of electronic and thermal Free Energies = -2626.490792

C	-0.556086	-0.371610	-0.698420
H	-0.470737	-1.082729	-1.510454
H	0.218227	0.380131	-0.640924
C	-1.839850	-0.011412	-0.333442
S	-2.097643	1.449281	0.631788
O	-3.307094	1.288540	1.440278
O	-0.824752	1.713558	1.304332
S	-3.238260	-0.825580	-1.057697
O	-4.202657	0.214803	-1.420615
O	-2.703901	-1.699641	-2.105357
C	-2.379228	2.803228	-0.500612
C	-3.677040	3.215301	-0.764879
C	-1.280935	3.427347	-1.078845
C	-3.876133	4.279819	-1.632430
H	-4.512303	2.707940	-0.302934
C	-1.494166	4.485723	-1.949474
H	-0.275921	3.101905	-0.842629
C	-2.788907	4.910140	-2.225115
H	-4.883748	4.615087	-1.846155
H	-0.647620	4.983422	-2.406544
H	-2.950778	5.739224	-2.903851
C	-4.045110	-1.897167	0.129997
C	-4.878345	-1.350554	1.097767
C	-3.874579	-3.269454	0.007206
C	-5.537457	-2.203179	1.970889
H	-5.001215	-0.279569	1.170588
C	-4.543415	-4.111197	0.884835
H	-3.239208	-3.670485	-0.770896
C	-5.368829	-3.579066	1.866998
H	-6.188724	-1.789949	2.731486
H	-4.421415	-5.183853	0.796022

H	-5.888951	-4.239582	2.550764
C	0.460204	-1.630707	0.631353
C	1.219498	-0.762874	1.456311
C	-0.600954	-2.481036	1.258088
H	0.995265	-2.082477	-0.195239
H	0.789709	-0.456985	2.401603
C	2.416423	-0.210048	1.095379
H	-1.270429	-1.893225	1.889395
H	-1.192207	-3.004216	0.508405
H	-0.131698	-3.241381	1.892737
O	2.923774	-0.442496	-0.131816
C	3.152227	0.743582	1.945965
Si	4.167065	-1.462016	-0.701370
C	3.019128	0.734745	3.339179
C	3.992128	1.694661	1.359139
C	3.650979	-3.234328	-0.416958
C	5.746615	-1.130195	0.236352
C	4.291249	-1.020411	-2.537274
C	3.700182	1.656655	4.117866
H	2.395609	-0.008578	3.819800
C	4.666407	2.620740	2.140818
H	4.101161	1.715063	0.282866
H	4.463345	-3.911223	-0.698476
H	3.437027	-3.412532	0.641056
H	2.766612	-3.516872	-0.992134
H	6.521488	-1.830112	-0.091272
H	6.125511	-0.117183	0.090148
H	5.605052	-1.286453	1.309282
C	4.524148	2.605562	3.522607
H	3.591896	1.630925	5.195850
H	5.304201	3.358869	1.668502
H	5.054181	3.326805	4.133550
C	4.657605	0.460808	-2.705795
H	4.742401	0.705997	-3.770880
H	3.896532	1.118349	-2.277737
H	5.616153	0.702200	-2.238152
C	5.384042	-1.886019	-3.184350
H	5.162431	-2.954441	-3.107377
H	5.466206	-1.646843	-4.250689
H	6.366297	-1.713289	-2.735337
C	2.954652	-1.290026	-3.242224
H	3.035787	-1.040706	-4.306582
H	2.659820	-2.340991	-3.176301
H	2.145898	-0.685539	-2.823922

TS-AD_Ph_2b

<u>SMD(DCE)-CAM-B3LYP/6-311+G(d,p)</u>

E = -2627.021894
Zero-point correction = 0.607328 (Hartree/Particle)
Thermal correction to Energy = 0.647910
Thermal correction to Enthalpy = 0.648854
Thermal correction to Gibbs Free Energy = 0.527285
Sum of electronic and zero-point Energies = -2626.414566

Sum of electronic and thermal Energies = -2626.373983
Sum of electronic and thermal Enthalpies = -2626.373039
Sum of electronic and thermal Free Energies = -2626.494609

C	-0.635474	-0.759227	0.198764
H	-0.593000	-1.734747	0.665401
H	0.069514	-0.579293	-0.600752
C	-1.884089	-0.177746	0.096418
S	-2.169501	1.138620	-1.045843
O	-1.079455	1.093050	-2.022144
O	-3.552343	1.031088	-1.512075
S	-3.245631	-0.833767	1.020023
O	-2.662560	-1.787719	1.968091
O	-4.071105	0.257606	1.538726
C	-2.013026	2.692519	-0.173538
C	-0.868582	3.454015	-0.366731
C	-3.051594	3.127241	0.641734
C	-0.761164	4.678400	0.279651
H	-0.085539	3.100589	-1.023911
C	-2.928571	4.349934	1.283599
H	-3.932696	2.515086	0.776926
C	-1.786096	5.122635	1.104531
H	0.123136	5.286307	0.131470
H	-3.729603	4.701858	1.922278
H	-1.698410	6.079009	1.606499
C	-4.246039	-1.778879	-0.119697
C	-3.801176	-3.034364	-0.514148
C	-5.451317	-1.257075	-0.563054
C	-4.587168	-3.780775	-1.378766
H	-2.861870	-3.427375	-0.146010
C	-6.232495	-2.017789	-1.421719
H	-5.767214	-0.274049	-0.242555
C	-5.800254	-3.273031	-1.830503
H	-4.254562	-4.761922	-1.695006
H	-7.178928	-1.625826	-1.773745
H	-6.412594	-3.861136	-2.503885
C	0.590150	0.050682	1.685461
C	1.438149	0.968033	1.011264
C	-0.331197	0.568348	2.747971
H	1.010356	-0.931071	1.868736
H	1.177537	2.018463	1.046189
C	2.517634	0.610323	0.252239
H	-0.875268	1.454000	2.413068
H	-1.049264	-0.187934	3.062591
H	0.257229	0.855078	3.627001
O	2.803133	-0.691571	0.061655
Si	3.986086	-1.733347	0.715734
C	3.705717	-1.847494	2.558435
C	5.693863	-1.051200	0.396675
C	3.671352	-3.368325	-0.182998
H	4.501727	-2.436557	3.023747
H	3.729592	-0.854106	3.016131
H	2.751707	-2.314031	2.812722
H	6.440739	-1.709539	0.851048

H	5.924397	-0.965666	-0.666668
H	5.814757	-0.063495	0.849893
C	4.660583	-4.421524	0.341109
H	4.490315	-5.377473	-0.167093
H	5.700592	-4.136509	0.158144
H	4.543147	-4.597767	1.414313
C	3.876294	-3.192054	-1.694286
H	3.178645	-2.463625	-2.115647
H	4.892115	-2.867294	-1.935873
H	3.706912	-4.145588	-2.207859
C	2.237726	-3.852863	0.074457
H	2.067981	-4.805887	-0.439905
H	2.044408	-4.017636	1.138088
H	1.497575	-3.140791	-0.298846
C	3.340676	1.587432	-0.484262
C	3.982278	1.205818	-1.666017
C	3.484079	2.905980	-0.037537
C	4.731556	2.120945	-2.390148
C	4.240202	3.815874	-0.759533
C	4.864304	3.428598	-1.940209
H	5.212627	1.811749	-3.310769
H	4.349819	4.830507	-0.394802
H	3.021288	3.217460	0.890693
H	3.876402	0.190146	-2.024054
H	5.454044	4.142068	-2.503713

TS-AD_Ph_2c

SMD(DCE)-CAM-B3LYP/6-311+G(d,p)

E = -2627.018844
Zero-point correction = 0.607124 (Hartree/Particle)
Thermal correction to Energy = 0.647788
Thermal correction to Enthalpy = 0.648732
Thermal correction to Gibbs Free Energy = 0.527551
Sum of electronic and zero-point Energies = -2626.411719
Sum of electronic and thermal Energies = -2626.371056
Sum of electronic and thermal Enthalpies = -2626.370112
Sum of electronic and thermal Free Energies = -2626.491293

C	-0.281368	-0.656972	0.448372
H	-0.199443	-1.725809	0.598808
H	0.329165	-0.231494	-0.335297
C	-1.518599	-0.088578	0.682573
S	-1.757411	1.618415	0.262418
O	-1.813197	2.431791	1.477268
O	-0.717677	1.925660	-0.724060
S	-2.776746	-1.004140	1.514274
O	-2.116423	-2.141287	2.160774
O	-3.598296	-0.102529	2.319881
C	-3.337499	1.755266	-0.552666
C	-4.401211	2.331012	0.125910
C	-3.448605	1.336497	-1.873306
C	-5.609382	2.489727	-0.539368
H	-4.279299	2.652068	1.151500
C	-4.661710	1.496908	-2.524023

H	-2.600355	0.901498	-2.386900
C	-5.738062	2.073728	-1.858127
H	-6.448797	2.941987	-0.025475
H	-4.764921	1.177792	-3.553872
H	-6.682858	2.201578	-2.373261
C	-3.828654	-1.677254	0.231532
C	-3.286155	-2.553883	-0.701028
C	-5.175855	-1.353266	0.230215
C	-4.118533	-3.112154	-1.657403
H	-2.231618	-2.800226	-0.683111
C	-6.001358	-1.925699	-0.729878
H	-5.570175	-0.668847	0.969318
C	-5.474494	-2.799097	-1.670610
H	-3.709934	-3.796091	-2.391317
H	-7.057277	-1.684464	-0.740160
H	-6.121625	-3.241173	-2.418932
C	1.124322	-0.242077	1.941572
C	1.838190	0.886888	1.459787
C	0.345954	-0.110574	3.215531
H	1.593683	-1.204394	1.774482
H	1.531327	1.866083	1.807101
C	2.826324	0.831624	0.517832
H	-0.264292	0.795071	3.220203
H	-0.299978	-0.970996	3.385420
H	1.043893	-0.043800	4.057952
O	3.151490	-0.348109	-0.048600
C	3.497720	2.029999	-0.017864
Si	4.453974	-1.424315	0.182898
C	3.611533	3.203551	0.735881
C	4.020088	2.012746	-1.314290
C	4.325854	-2.140595	1.902940
C	6.076046	-0.515235	0.019592
C	4.196581	-2.720473	-1.172005
C	4.222651	4.326997	0.202388
H	3.237263	3.233755	1.751476
C	4.624460	3.141171	-1.848019
H	3.934051	1.113220	-1.909572
H	5.193056	-2.774639	2.110838
H	4.319693	-1.345684	2.654474
H	3.427739	-2.745816	2.043037
H	6.903376	-1.201961	0.223582
H	6.228026	-0.091769	-0.974694
H	6.144431	0.298196	0.747233
C	4.729224	4.302153	-1.092422
H	4.308924	5.225356	0.802345
H	5.012939	3.114274	-2.859469
H	5.205015	5.182614	-1.508133
C	4.267351	-2.063489	-2.557735
H	4.130061	-2.821388	-3.337825
H	3.486077	-1.310489	-2.689687
H	5.233619	-1.583579	-2.735951
C	5.301402	-3.783217	-1.060932
H	5.281176	-4.297983	-0.095989

H	5.166709	-4.543312	-1.838973
H	6.299090	-3.354957	-1.193623
C	2.828870	-3.398402	-1.008605
H	2.688631	-4.150405	-1.793842
H	2.736602	-3.910364	-0.046753
H	2.009216	-2.679843	-1.089752

TS-AD_Ph_2d

SMD(DCE)-CAM-B3LYP/6-311+G(d,p)

E = -2627.020143
Zero-point correction = 0.607129 (Hartree/Particle)
Thermal correction to Energy = 0.647760
Thermal correction to Enthalpy = 0.648704
Thermal correction to Gibbs Free Energy = 0.527898
Sum of electronic and zero-point Energies = -2626.413014
Sum of electronic and thermal Energies = -2626.372383
Sum of electronic and thermal Enthalpies = -2626.371438
Sum of electronic and thermal Free Energies = -2626.492244

C	0.749586	-0.744269	-0.312750
H	0.748629	-1.808236	-0.113150
H	0.085367	-0.149332	0.300354
C	1.946470	-0.192754	-0.716934
S	2.162282	1.559933	-0.777353
O	3.593652	1.832359	-0.909133
O	1.245407	2.134101	-1.763699
S	3.256239	-1.241465	-1.291099
O	2.724260	-2.605714	-1.210653
O	3.769604	-0.765895	-2.573814
C	1.632936	2.130393	0.832889
C	2.425406	1.842415	1.937638
C	0.461925	2.863311	0.943624
C	2.026439	2.300806	3.182938
H	3.341156	1.274211	1.827706
C	0.074315	3.319404	2.197670
H	-0.127766	3.080948	0.063013
C	0.852366	3.036747	3.311818
H	2.633736	2.086772	4.053926
H	-0.836387	3.896893	2.299198
H	0.546052	3.393927	4.287938
C	4.572545	-1.121279	-0.089679
C	4.456463	-1.831926	1.098833
C	5.690631	-0.353356	-0.377092
C	5.488140	-1.762443	2.022723
H	3.580083	-2.436833	1.294779
C	6.719220	-0.297827	0.553413
H	5.747498	0.190391	-1.309629
C	6.616763	-0.997199	1.749334
H	5.414335	-2.312899	2.952686
H	7.601170	0.294353	0.341726
H	7.422635	-0.949090	2.472310
C	-0.660936	-0.957732	-1.866103
C	-1.474168	0.198518	-1.760828
C	0.150196	-1.161488	-3.108755

H	-1.049115	-1.857500	-1.403881
H	-1.207226	1.054301	-2.367462
C	-2.511958	0.340068	-0.881076
H	0.676057	-0.249679	-3.397243
H	0.873288	-1.968511	-2.994270
H	-0.518398	-1.432972	-3.933763
O	-2.798334	-0.647499	-0.008879
C	-3.291502	1.584847	-0.748285
Si	-3.987063	-1.870663	0.010618
C	-3.400376	2.498087	-1.804163
C	-3.925453	1.883209	0.461747
C	-3.757544	-2.943923	-1.500084
C	-5.691219	-1.110056	-0.034064
C	-3.637063	-2.786978	1.629209
C	-4.114666	3.674973	-1.647610
H	-2.940573	2.280360	-2.760031
C	-4.634804	3.064981	0.616885
H	-3.846484	1.188867	1.287710
H	-4.566693	-3.677655	-1.563636
H	-3.795221	-2.342099	-2.412856
H	-2.811331	-3.488753	-1.493550
H	-6.445248	-1.902826	-0.060490
H	-5.897816	-0.479017	0.832164
H	-5.827278	-0.504403	-0.934214
C	-4.733252	3.965403	-0.436312
H	-4.195101	4.366431	-2.478214
H	-5.112283	3.283726	1.565017
H	-5.291295	4.886610	-0.316667
C	-3.807212	-1.839101	2.824885
H	-3.610125	-2.377396	3.759329
H	-3.111006	-0.997632	2.777141
H	-4.821703	-1.436150	2.888577
C	-4.628331	-3.953616	1.765139
H	-4.532660	-4.671605	0.945470
H	-4.439285	-4.496505	2.698246
H	-5.666373	-3.610059	1.792755
C	-2.205301	-3.340720	1.629881
H	-2.014363	-3.881185	2.564324
H	-2.035219	-4.041241	0.807683
H	-1.462941	-2.542193	1.553657
